# PEG3 Interacts with KAP1 through KRAB-A

**DOI:** 10.1371/journal.pone.0167541

**Published:** 2016-11-29

**Authors:** Hongzhi He, An Ye, Hana Kim, Joomyeong Kim

**Affiliations:** Department of Biological Sciences, Louisiana State University, Baton Rouge, Louisiana, United States of America; Universitat des Saarlandes, GERMANY

## Abstract

*Peg3* (Paternally Expressed Gene 3) is an imprinted gene that encodes a zinc finger DNA-binding protein. *Peg3* itself is localized in the middle of a KRAB-A (Kruppel-Associated Box) zinc finger gene cluster. The amino acid sequence encoded by its exon 7 also shows sequence similarity to that of KRAB-A, suggesting *Peg3* as a KRAB-containing zinc finger gene. As predicted, the PEG3 protein was co-immunoprecipitated with KAP1, a co-repressor that interacts with KRAB-A. A series of follow-up experiments further demonstrated that the exon 7 of PEG3 is indeed responsible for its physical interaction with KAP1. ChIP and promoter assays also indicated that PEG3 likely controls its downstream genes through the KAP1-mediated repression mechanism. Overall, the current study identifies PEG3 as a KRAB-containing zinc finger protein that interacts with the co-repressor protein KAP1.

## Introduction

*Peg3* (Paternally Expressed Gene 3) is an imprinted gene that encodes a zinc finger DNA-binding protein [1, 2]. As an imprinted gene, the genomic region surrounding the promoter of *Peg3* is usually methylated during oogenesis, thus the paternal allele is mainly expressed and functional in somatic cells [3–5]. Mutagenesis experiments indicated that this gene is involved in controlling the fetal growth rates and nurturing behaviors of placental mammals [6, 7]. Consistent with this, *Peg3* is highly expressed in embryos, placenta and neuronal cells [5, 8, 9]. In humans, *PEG3* has been often identified as one of the epigenetically affected genes in several cancers, including ovarian and breast cancers, thus regarded as a potential tumor suppressor [10–12]. At the molecular level, the protein encoded by this imprinted gene is known to bind to a large number of genomic targets as a DNA-binding transcription factor [1, 2]. Recent studies also revealed that this protein functions as a repressor controlling the transcriptional rates of its downstream genes [1, 2]. This has been further supported by the observation that the mutant embryos lacking PEG3 tend to de-repress several placenta-specific gene families in the brain [7]. However, the detailed mechanisms for the predicted repression are currently unknown.

*Peg3* is localized in the middle of a large zinc finger gene cluster in human chromosome 19q13.4/proximal mouse chromosome 7 [9]. This gene cluster spans a two megabase pair (bp) genomic distance, and contains more than 50 individual zinc finger genes [9]. In fact, its immediate neighbors are also another zinc finger genes with genomic imprinting, including *Zim1*, *Zim2*, *Zim3* and *Zfp264* [13–15]. The zinc finger genes in this cluster all share a similar protein domain structure, which consists of the KRAB-A (Kruppel-Associated Box A) domain at the N-terminus and the Cys2His2-Kruppel-type zinc finger domain at the C-terminus [16, 17]. The C2H2 zinc finger domain is responsible for DNA binding, whereas the KRAB-A domain is responsible for the physical interaction and subsequent recruitment of another protein called KAP1 (Kruppel-Associated Protein 1) [18]. KAP1 is a well-known co-repressor that interacts with several epigenetic modification proteins, including LSD1 (Lysine-Specific Demethylase 1), HDACs (Histone Deacetylases), SETDB1 (H3K9 histone methytransferase), and DNMT3A (*de novo* DNA methytransferase 3A) [19, 20]. Thus, the zinc finger genes containing the KRAB-A domain are thought to exert their repression functions through KAP1 [20]. Given the close localization to the other zinc finger genes, it has been believed that *Peg3* might have originated from a similar zinc finger gene during mammalian evolution, and that the repression roles played by PEG3 might be also mediated through similar mechanisms, which involve the co-repressor KAP1 [21].

In the current study, we performed a series of evolutionary and *in vitro* analyses to test whether PEG3 interacts with and exerts its repression roles through KAP1. According to the results, PEG3 indeed contains a divergent KRAB-A domain as one of 5’-side small exons, confirming the evolutionary origin of *Peg3* as a KRAB zinc finger gene. Furthermore, this divergent KRAB-A domain of PEG3 is still functional as a subdomain that interacts with the co-repressor KAP1. More detailed results are presented below.

## Results

### Identification of the KRAB-A domain within PEG3

**Fig 1:**
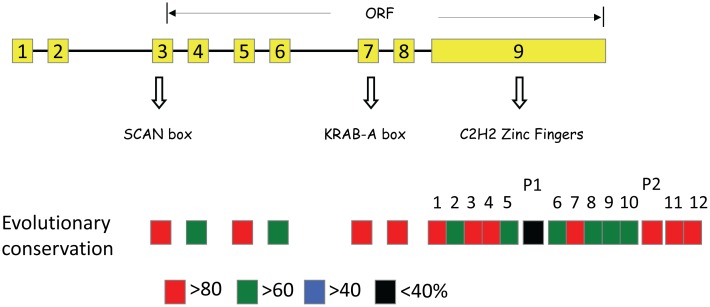
Evolutionary conservation of mammalian PEG3 proteins. The exon structure of mouse *Peg3* is presented with boxes indicating individual exons. The conservation level of each exon at the amino acid sequence level was calculated with Clustal Omega or Cobalt using the 10 representative PEG3 sequences, which were further summarized with a scheme involving boxes with different colors. The sequence of Exon9 was further divided into protein subdomains, including 12 zinc finger motifs and two proline-rich motifs (P1 and P2).

The amino acid sequence of mouse PEG3 was used as a probe to retrieve the protein sequences of PEG3 from the draft-stage genome sequences of the other mammals. This series of database searches successfully identified PEG3 sequences from the majority of 45 placental mammals, but none from the other vertebrates, such as opossum, platypus, avian, reptiles and fishes. This suggests that the *Peg3* locus may have been formed after the split of placental mammals from the other vertebrates. More than a half of these identified sequences are full-length sequences as compared to the ORF (Open Reading Frame) of mouse PEG3. Their exon structures are also very similar to that of the mouse *Peg3* locus: 9 exons are distributed throughout the 30-kb genomic region of each species (Fig 1). Ten of these sequences representing several eutherian lineages were selected and used for the evolutionary analyses of the current study (S1 File).

Multiple sequence alignment of the identified PEG3s derived the following observations (S2 File). First, twelve C2H2 zinc finger motifs (hereafter referred to as ZF) are found within the C-terminal region of the majority of the identified PEG3 sequences. These identified ZFs are all localized in the C-terminal 1,000 amino acid long region, which is encoded within the last exon, Exon9. The conservation levels of several ZF motifs (ZF1, 3, 4, 7, 11, 12) are much greater than those from the remaining ZF motifs. In fact, some of ZF motifs have been degenerated in a couple of individual species, including ZF2 for cow and horse. This indicated that individual ZF motifs might have been under the different levels of functional selection during evolution. Second, the alignments also identified several regions of the conserved domains that are localized between individual ZF motifs. These include two proline-rich motifs: the first one between ZF5 and 6 and the second one between ZF10 and 11. The first one appears to be rodent-specific: it is found only within mouse and rat. In contrast, the second one is found in all of the mammals examined so far, indicating high levels of functional constraints on this particular subdomain. The amino acid sequences of Exon3 also show high levels of sequence identity to a known motif termed the SCAN box, which is also associated with C2H2 Kruppel-type zinc finger proteins [16, 17]. Interestingly, Exon3 of rodent PEG3s do not have a proper ATG start codon that is located upstream of Exon3. Thus, it is unlikely that rodent PEG3s have this SCAN box as part of their ORFs. Consistent with this, the amino acid sequences of Exon3 of mouse and rat PEG3 show high levels of divergence as compared to those of the remaining mammals, suggesting that rodent PEG3s might have lost this subdomain.

**Fig 2:**
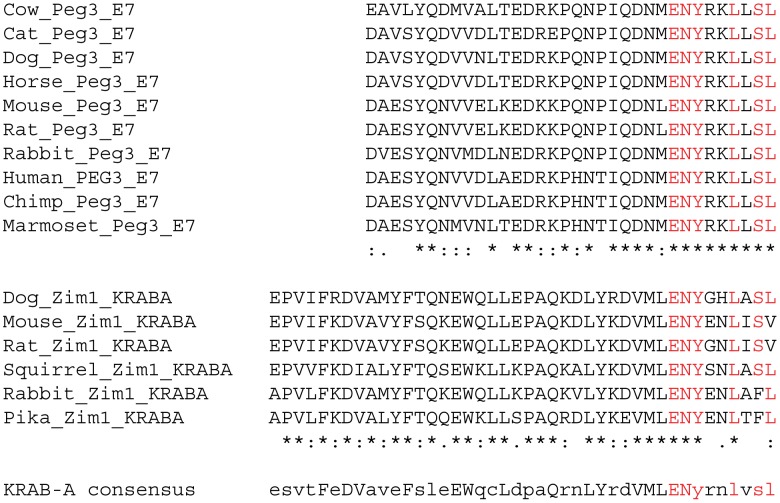
Identification of KRAB-A domain within PEG3. The amino acid sequences of Exon7 of mammalian PEG3 were aligned using Clustal Omega (upper panel). The amino acid sequences of the KRAB-A exon of mammalian ZIM1 were also similarly analyzed (lower panel). The 9-amino-acid-long region at the C-terminus of Exon7 of PEG3 shows sequence similarity to that of the KRAB-A region as indicated with red. The sequence on bottom indicates the consensus sequence of the KRAB-A domain.

The multiple sequence alignments also showed high levels of conservation in the amino acid sequences of Exon4 through 8. The sequences spanning from Exon4 through 6 showed on average 64.2, 84.8, 66.2%, respectively, between individual mammal sequences. In contrast, Exon7 and 8 show much higher levels of sequence conservation, on average 82.4 and 94.2% sequence identity, respectively. An independent database search using the sequence of Exon8 did not yield any hint regarding its potential connection to known domains, thus Exon8 is regarded as a novel motif unique to the PEG3 protein. However, a similar search with Exon7 derived an unexpected discovery that the 9 amino-acid-long motif found at the C terminus of Exon7 is very similar to that of KRAB-A domain, which has been compared with the KRAB-A domain of *Zim1* (Fig 2). Yet, this small motif is well conserved among all of the mammalian PEG3s, suggesting potential functional selection during evolution. Although the conserved region is relatively short, the observed similarity suggests that Exon7 might have been a KRAB-A domain as part of the ancestral locus of mammalian *Peg3*. Taken together, this series of evolutionary analyses identified full-length sequences of mammalian PEG3 and also a short motif within Exon7, which shows sequence similarity to the KRAB-A domain. Thus, it is most likely that the modern *Peg3* locus might have originated from an ancestral KRAB-A zinc finger gene during mammalian evolution.

### Identification of the potential interacting partners of PEG3

**Fig 3:**
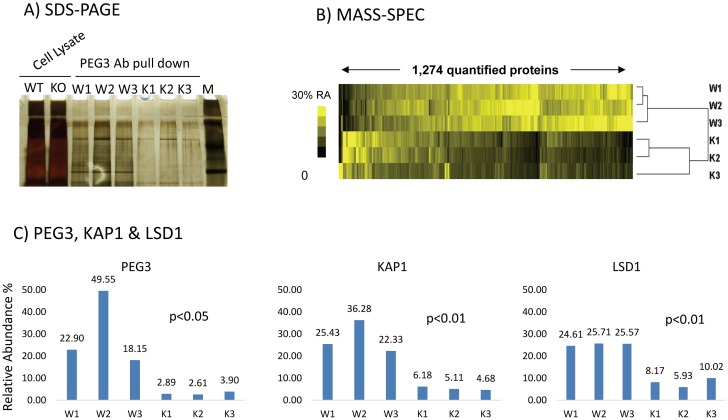
Identification of the potential interacting partners of PEG3. (**A**) The proteins extracts derived from a set of WT (W1-3) and KO (K1-3) MEF cells were immunoprecipitated with anti-PEG3 antibody. The quality of the isolated precipitates was first monitored through SDS-PAGE. The cell lysates (WT, KO) before the immunoprecipitation were also included along with size markers (M). (**B**) The isolated precipitates were further analyzed using a series of tandem mass spectrometry. A total of 1,274 proteins were successfully identified from each of the 6 tested samples. The relative abundance (RA) of each protein was summarized using a heat map showing different levels of abundance. (**C**) The relative abundance of three proteins in the WT and KO samples were summarized with graphs with statistical significances.

We performed a series of co-immunoprecipitation experiments to identify potential interacting partners of the PEG3 protein (Fig 3). This series of experiments utilized a set of MEF (Mouse Embryonic Fibroblast) cells, wild-type (WT) and knockout (KO), which were derived from the 14.5-dpc (days post coitum) embryos of the crossing between male heterozygotes for the *Peg3*^*CoKO*^ (conditional KO-ready) allele and female breeders [7]. The *Peg3*^*CoKO*^ allele is a mutant allele truncating the transcription of the *Peg3* locus. Since *Peg3* is expressed mainly from the paternal allele, the KO embryos and corresponding MEFs inheriting the mutant allele paternally are functionally null without any detectable levels of the PEG3 protein [7]. First, two sets of the protein extracts prepared from WT and KO MEFs were individually immunoprecipitated with the polyclonal antibody raised against the PEG3 protein (Fig 3A). The subsequent immonoprecipitates, three WT and KO samples, were further analyzed with tandem mass spectrometry (Fig 3B). According to the results, a set of 1,274 proteins was successfully identified from each of 6 samples (S3 File). Comparison of the relative abundance of each protein between the WT and KO sets further identified 302 proteins that were differentially represented between the two sets. The raw spectral counts of these proteins were greater than 3, and also their differential representation between the WT and KO sets were greater than 2.5. Inspection of these proteins with gene ontology analysis revealed that 212 proteins are localized in the cytoplasm whereas 64 proteins are in the nucleus of the cells. The remaining 26 proteins are considered to be mitochondria-associated proteins. More careful inspection of raw spectral counts also identified PEG3 as one of the most differentially represented proteins with the relative abundance ratio between WT and KO being 1 to 0.102 (Student’s t test, *p* < 0.05). This is consistent with the fact that the KO samples lack the PEG3 protein (Fig 3C). A group of 62 proteins also showed a similar pattern of differential representation with much lower levels in KO than in WT. Some of these proteins are most likely the potential interacting partners of PEG3 since the co-immunoprecipitation of these proteins with anti-PEG3 antibody were detected in WT, but not in KO. Some of the noteworthy proteins include ACLY (ATP Citrate Lyase), MEST (Mesoderm specific transcript), KAP1 and KDM1A (or LSD1). ACLY is the key enzyme producing cytosolic acetyl-CoA, thus important for lipogenesis and cholesterogenesis [22, 23]. On the other hand, KAP1 is a well-known co-repressor that interacts with numerous zinc finger proteins through their KRAB-A domains [20]. It is also relevant to note that KAP1 functions as a glue protein brining together various histone modification enzymes, such as LSD1. Thus, the detection of KAP1 and LSD1 is thought to be significant and also relevant to the functions of the PEG3 protein since PEG3 contains a divergent KRAB-A domain (Fig 2). Overall, this series of tandem mass spectrometry identified a large number of potential interacting partners for the PEG3 protein. In particular, the potential interaction with the two proteins, KAP1 and LSD1, is thought to be the most relevant to the predicted functions of PEG3, a DNA-binding protein with transcriptional repression.

### KAP1 as an interacting partner of PEG3

**Fig 4:**
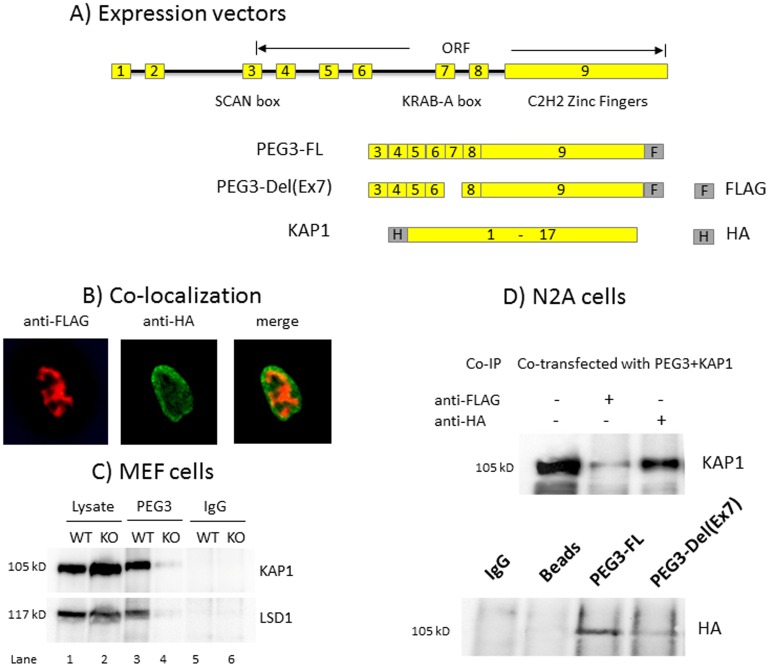
KAP1 as an interacting partner of PEG3. (**A**) A schematic representation indicates the exon structure and the ORF of mouse PEG3. The current study used three constructs expressing mouse PEG3 and human KAP1: PEG3-FL, the full-length PEG3 with the FLAG tag at the C-terminus; PEG3-Del(Ex7), the mutant version of mouse PEG3 lacking Exon7 with the FLAG tag at the C-terminus; KAP1, the full-length KAP1 with the HA tag at the N-terminus. (**B**) Co-localization experiments. As a control experiment, the cells were co-transfected with two constructs, PEG3-FL-FLAG and KAP1-HA constructs. The expression and subsequent localization within the nuclei were individually visualized with anti-FLAG (left) and anti-HA (middle) antibodies. These two images were later merged together (right). (**C**) The protein extracts derived from MEF cells were individually immunoprecipitated with anti-PEG3 antibodies, and further analyzed with western blotting using anti-KAP1 antibody (upper). A similar series of Co-IP experiments with anti-PEG3 antibody were repeated using the protein extracts from WT and KO MEFs, and later analyzed with western blotting using anti-LSD1 antibody (lower). (**D**) Neuro2a cells were co-transfected with the constructs expressing the full-length PEG3 and KAP1, immunoprecipitated with anti-FLAG and HA antibodies, and finally analyzed with western blotting using anti-KAP1 antibody (upper). A similar series of Co-IP experiments were repeated with two constructs expressing the full-length and mutant versions of PEG3. In this case, the immunoprecipitates were analyzed with western blotting using anti-HA antibody (lower).

The potential interaction between PEG3 and KAP1 was further tested using various *in vitro* experiments described below. First, we repeated an independent series of immunoprecipitation experiments with anti-PEG3 antibody using the protein extracts derived from MEF cells (Fig 4C). The subsequent immunoprecipitates were analyzed with western blotting using anti-KAP1 antibody. The results indicated that KAP1 was co-immunoprecipitated with anti-PEG3 antibody (the 3^rd^ lane on the upper panel of Fig 4C). In contrast, KAP1 was not detectable in the KO sample (the 4^th^ lane on the upper panel of Fig 4C), indicating that the precipitation and subsequent detection of KAP1 in WT was likely mediated through PEG3. We also repeated a similar series of experiments with anti-LSD1 antibody. The results also showed co-immunoprecipitation of LSD1 with anti-PEG3 antibody, supporting the possibility that PEG3 might also interact with LSD1 (the lower panel of Fig 4C). Second, this initial observation was further followed up with another series of co-immunoprecipitation experiments using various constructs expressing PEG3 and KAP1 (Fig 4A). The Neuro2A cells (N2A) were transfected with the two following expression constructs: the first one containing the full-length PEG3 with the FLAG tag at the C-terminus whereas the second one containing the full-length KAP1 with the HA tag at the N-terminus. The protein extracts from the N2A cells that had been transfected with these two constructs were individually immunoprecipitated with anti-FLAG and anti-HA antibodies. These immunoprecipitates were subsequently analyzed with western blotting using anti-KAP1 antibody (the upper panel on Fig 4D). The results indicated that PEG3 was indeed co-immunoprecipitated with KAP1. This series of co-transfection experiments were repeated again with another set of two constructs: the first one containing the full-length PEG3 whereas the second one containing the mutant version of PEG3 lacking Exon7. The immunoprecipitates with anti-FLAG antibody, which was designed to pull-down PEG3, detected high levels of KAP1 from the extracts transfected with the full-length PEG3, but very low levels of KAP1 from those with the mutant version of PEG3 (the lower panel on Fig 4D). This confirmed that the interaction between PEG3 and KAP1 is likely mediated through Exon7, a divergent KRAB-A domain. This further suggests that the divergent KRAB-A in PEG3 is still functional as a subdomain recruiting KAP1. As a control experiment, we also performed a series of co-localization experiments with two expression constructs, PEG3-FL-FLAG and KAP1-HA (Fig 4A). The immunostaning of the cells transfected with these two constructs indicated that two proteins are localized within the nuclei but excluded from the nucleolar areas, and further that some fraction of these two proteins are co-localized within the nuclei (Fig 4B and S4 File). Overall, this series of *in vitro* experiments concluded that PEG3 may interact with KAP1, and that this interaction may be mediated through Exon7.

### PEG3 binding to its targets with KAP1

**Fig 5:**
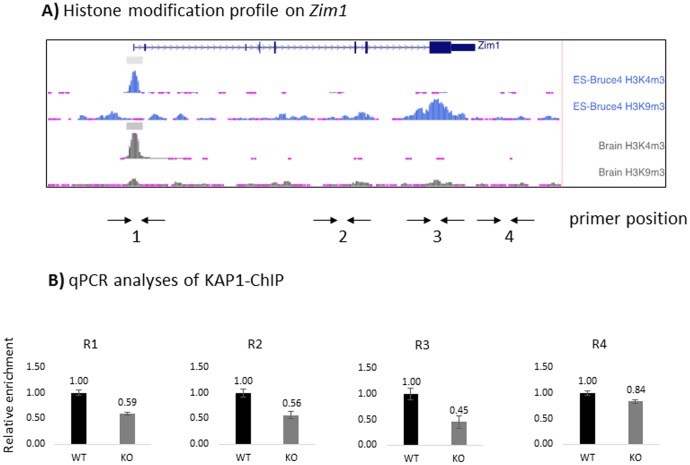
PEG3 binding to its targets with KAP1. The potential interaction of PEG3 and KAP1 was further tested using ChIP experiments. PEG3 is known to bind to the zinc finger exon of *Zim1*, thus the 4 regions covering the *Zim1* locus were used for this analysis (**A**). The chromatin prepared from WT and KO MEFs were precipitated with anti-KAP1 antibody, and the eluted DNA was subsequently analyzed using qPCR. The results were summarized with graphs with statistical significance (**B**).

The potential interaction between PEG3 and KAP1 was also tested using a series of ChIP experiments (Fig 5). According to the results from previous studies, PEG3 is known to bind to the zinc finger exon of the adjacent imprinted gene *Zim1* [24]. This binding of PEG3 is also known to be responsible for the transcriptional repression of *Zim1* [24]. However, the detailed mechanisms for this repression have not been well characterized so far, although the zinc finger exon of *Zim1*, the target region of PEG3, is known to be marked with the H3K9me3 (Fig 5A). Since KAP1 is a potential co-repressor for PEG3, we tested whether PEG3 binds to the zinc finger exon of *Zim1* along with KAP1. This series of experiments also utilized a similar set of MEF cells, WT and KO, as described above. The chromatin prepared from WT and KO MEFs was first immunoprecipitated with anti-KAP1 antibody, and the isolated DNA was later analyzed with quantitative PCR (Fig 5B). According to the results, the enrichment levels of several regions within *Zim1*, R1 through 3, were lower in KO than those from WT. This indicated that the binding of KAP1 to the *Zim1* locus was reduced in the KO cells lacking PEG3. In particular, the R3 region, the known target region of PEG3, showed the lowest levels among the four tested loci. As described above, this zinc finger exon is also known to be marked with H3K9me3, requiring the involvement of KAP1 (Fig 5A). Thus, this suggests that PEG3 might be the one recruiting KAP1 to the *Zim1* locus. This further implies that the transcriptional repression of *Zim1* by PEG3 may be mediated through KAP1.

### Exon7 for the repression function of PEG3

**Fig 6:**
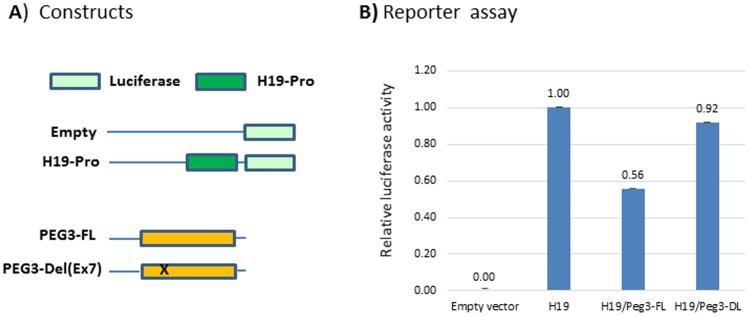
Exon7 for the repression role of PEG3. A series of reporter assays were performed to test potential roles played by Exon7 for the repression function of PEG3. This series of assays used two reporters: promoterless basic (Empty) and H19-promoter-containing reporters (H19-prom). The H19-prom construct was also co-transfected individually with the full-length and mutant versions of PEG3 (**A**). The graph summarizes the results derived from this series of reporter assays. The obtained values of the luciferase activity were first normalized with the amount of total proteins, and later compared to the value of the sample transfected with the H19-prom construct only without the PEG3 expression construct. This series of experiments were repeated with two independent trials (**B**).

We also tested potential roles played by Exon7 for the repression function of PEG3. According to the previous studies, a number of genomic targets are known to be bound by PEG3, one of which turned out to be the promoter region of another imprinted gene *H19* [Ye et al, unpublished]. Thus, we transfected Neuro2a and HEK293 cells with a series of luciferase constructs: the first one without any promoter (Empty vector), the second one with the promoter of *H19* (Fig 6A). As expected, the reporter activity of the H19-promoter construct was much higher than that of the Empty construct (Fig 6B). The H19-promoter construct was again co-transfected individually with the full-length PEG3 expression construct and also with the mutant version of PEG3 construct lacking Exon7. As expected, the full-length PEG3 reduced the promoter activity of *H19* by 44%, confirming the repressor role played by PEG3 in the transcription of *H19*. The mutant version of PEG3, on the other hand, did not show any difference compared to the levels observed from the cells without any co-transfection of PEG3. This suggests that the repression function of PEG3 is likely mediated through Exon7. A similar conclusion was also derived from the results from the experiment set using HEK293 cells (data not shown). However, the repressive effect by the full-length PEG3 was much milder than that from the Neuro2a set, reducing the promoter activity of *H19* by 21%. This is believed to be caused by the reduced activity of KAP1 in HEK293 cells, which has been transformed by the adenovirus E1B protein. Taken together, this series of *in vitro* promoter assays confirmed that Exon7 plays a critical role in the repression function of PEG3.

## Discussion

Through comparative genomic approaches, the current study has identified a divergent KRAB-A domain as part of the ORF of mammalian PEG3. A series of follow-up experiments further demonstrated that this divergent KRAB-A is indeed functional as a subdomain recruiting KAP1, and also suggest that this domain might be critical for the repression function of PEG3.

*Peg3* has been relatively well known as an imprinted gene, but the functional aspect of this gene, in particular the protein encoded by this gene, has not been well characterized so far. According to the results, several subdomains are well conserved among all mammals, including several zinc finger motifs (ZF1, 3, 4, 7, 11 and 12) and one proline-rich domain, consistent with the prediction that the PEG3 protein is likely a DNA-binding protein (Fig 1). It is interesting to note that the ZF motifs located in either N- or C-terminus are more conserved than the other remaining ZFs. This is consistent with the pattern observed from the other Kruppel-type zinc finger proteins [25, 26], and further suggests that those conserved ZF motifs are likely involved in DNA binding. In contrast, the ZF motifs located in the middle seem to have evolved very fast with minimum levels of functional constraints, thus these ZF motifs might have adapted to some functions other than DNA binding, including protein-protein interaction as seen in the other zinc finger proteins [27]. Besides the ZF motifs, three subdomains are also well conserved, including a SCAN domain in Exon3, a divergent KRAB-A domain in Exon7 and an unknown conserved domain in Exon8 (Figs 1 and 2). The identification of the two subdomains, SCAN and KRAB-A, is informative for pinpointing the evolutionary origin of the mammalian *Peg3* locus. The *Peg3* locus is surrounded by a large number of zinc finger genes, the majority of which contain KRAB-A domains at their N-terminus [9]. Some of these also have SCAN domains as part of their ORFs. Therefore, the detection of SCAN and KRAB-A within the ORF of PEG3 strongly supports the possibility that the *Peg3* locus might have originated as an early member of this large zinc finger gene family. Since non-eutherian mammals and the other vertebrates do not have similar sequences to *Peg3*, it is also likely that the formation of *Peg3* might have occurred right after the split of the eutherian from the other vertebrates during mammalian evolution. Overall, the results suggest that the mammalian *Peg3* locus may have originated from an ancestral zinc finger gene with SCAN and KRAB-A domains.

The PEG3 protein appears to contain a divergent KRAB-A domain, yet this domain has been shown to interact with KAP1 (Figs 3, 4 and 5). Although this domain is quite divergent, a series of unbiased proteomic analyses indicated that KAP1 is indeed one of the strongest candidates that were co-immunoprecipitated with PEG3 (Fig 3). Furthermore, the results from *in vitro* experiments also demonstrated that this divergent KRAB-A is responsible for the interaction between PEG3 and KAP1 (Fig 4). This further confirmed that this divergent KRAB-A is still functional as a subdomain recruiting KAP1. The potential interaction between PEG3 and KAP1 is also insightful for illuminating the unknown functions of the PEG3 protein. It has been known that PEG3 functions as a transcriptional repressor for its downstream targets [1, 2]. Since KAP1 is known to interact with various histone-modifying proteins, it is reasonable to predict that PEG3 might control its downstream targets through some of these histone-modifying proteins. Two of the well-known such partners are SETDB1 and LSD1, which are responsible for the methylation on H3K9 and the demethylation on H3K4me1 or 2, respectively [19, 28]. In fact, the co-immunoprecipitation experiments confirmed that PEG3 might also interact with LSD1 (Fig 4C). Therefore, it is possible that the functional outcome of the binding of PEG3 to its targets might manifest through some of these histone modifications. In that regard, it is relevant to point out the previous observation that several placenta-specific gene families known to be repressed with H3K9me3 are, in fact, de-repressed in the embryos lacking PEG3 [7]. This again supports the possibility that PEG3 might control its downstream targets through histone modifications. Overall, although premature at the moment, these lines of evidence strongly suggest that the repression function of PEG3 is likely mediated through KAP1.

The results from tandem mass spectrometry revealed that many proteins might interact with PEG3, including several nuclear proteins as well as various cytoplasmic proteins (Fig 3 and S3 File). As a potential DNA-binding protein, the interaction of PEG3 with nuclear proteins was expected. On the other hand, the potential interaction with cytoplasmic proteins was unexpected at first. However, it is also feasible since several previous studies have already reported that PEG3 is detected within the cytoplasm as part of the autophagosome in endothelial cells and also associated with the outside membrane of mitochondria upon the commencement of apoptosis [29, 30]. In that regard, some of potential interacting partners are noteworthy based on their known functions. In particular, the potential interaction of PEG3 with ACLY (ATP Citrate Lyase) is very intriguing in that this protein is a main enzyme controlling the rates of the lipogenesis [22, 23]. One of the well-known phenotypes observed from Peg3 KO models is that a large amount of adipocytes are known to be accumulated at the expense of muscle mass [31]. Although more work needs to be done in the near future, it is reasonable to predict that cytoplasmic PEG3 might be involved in controlling the lipogenesis through the physical interaction with ACLY. Besides ACLY, several other enzymes are also potential interacting partners, including HK2 (Hexokinase2). It is interesting to note that these proteins all seem to be involved in controlling glycolysis or gluconeogenesis. The potential connection with these enzymes appears to be consistent with the fact that PEG3 is involved in controlling energy metabolism. Overall, a list of potential interacting partners of PEG3 provides several new directions for the future study of the PEG3 protein, not only as a DNA-binding protein but also as a cytoplasmic protein controlling energy metabolism.

## Materials and Methods

### Ethics statement

All the experiments related to mice were performed in accordance with National Institutes of Health guidelines for care and use of animals, and also approved by the Louisiana State University Institutional Animal Care and Use Committee (IACUC), protocol #16–060.

### Database search and sequence analyses

A series of database searches were conducted using the BLAT program (http://genome.ucsc.edu/cgi-bin/hgBlat) to obtain PEG3-related sequences. Mouse PEG3 (GenBank accession No. NP_032843) was used as a query sequence to search sequence databases, including NCBI and the Genome Browser at University of California Santa Cruz (http://genome.ucsc.edu/). The 10 representative PEG3 sequences from different mammals are included as S1 File. Multiple sequence alignments were performed with Clustal Omega (http://www.ebi.ac.uk/Tools/msa/clustalo/) and also with Cobalt (http://www.ncbi.nlm.nih.gov/tools/cobalt/re_cobalt.cgi). The output from Cobalt using the 10 representative sequences is also included as S2 File.

### Plasmid construction

The ORF of mouse PEG3, corresponding to amino acid residue 1–1,571 (GenBank accession No. NP_032843), was amplified from the expression vector pCDNA-PEG3-FL [1] with the following two primers: BamHI-mPeg3-CDS-F (5’-ATATGGATTCATGTACCATCACGAAGACGACACC-3’) and EcoEV-mPeg3-CDS-R (5’-GAGTGATATCACCAGTGTGAGAATTCTGGTGTCT-3’). The amplified fragment was cloned into the pCMV-3T3 vector (Agilent Technologies), named pCMV-3T3-PEG3-FL. This construct, in short PEG3-FL, was use for the expression of the full-length PEG3 with the FLAG tag at the C-terminus. The exon 7 of PEG3 was deleted using a PCR scheme involving the two primers: BamHI-mPeg3-CDS-F and XbaI-mPeg3-CDS-R (5′-CTTCCTTTCTAGAGCTCTGCTTCTGG-3’). The added *Xba*I site was ligated to another *Xba*I site that is already included in the ORF of mouse PEG3, resulting in the deletion of the exon 7. The proper deletion and integrity of the final construct, named PEG3-Del(Ex7), was confirmed through sequencing. The current study also used the construct pKH3-TRIM28 that is designed to express human KAP1 with 3 HA tags at the N-terminus (Plasmid No. 45569, Addgene) [32].

#### Tissue culture and transfections

All cells were maintained in Dulbecco’s modified Eagle’s medium (Cat. No. 10566–016, Life Technologies) containing 10% fetal bovine serum (Cat. No. SH30088.03, Hyclone). Mouse embryonic fibroblast (MEF) cells were derived from 14.5-dpc embryos as described previously [24]. For Co-IP experiments, Neuro2a cells (CCL-131, ATCC) in 6-well plates were co-transfected with pKH3-TRIM28 (KAP1) combined with either the PEG3-FL or PEG3-Del(Ex7) constructs using the Lipofectmine 2000 method. The transfected cells were incubated for 48 hours in 2.5 ml of medium before the planned Co-IP experiments.

### Immunoprecipitation and immunoblotting

The immunoprecipitation for tandem mass spectrometry used the following procedure. Wild type and CoKO MEF cells in 100mm dish were collected by centrifugation, and the subsequent pellets were lysed in 1 ml of the Pierce IP Lysis Buffer (Cat. No. 87787, Thermo Scientific) containing 25 mM Tris-HCl pH 7.4, 150 mM NaCl, 1% NP-40, 1 mM EDTA, 5% glycerol. The prepared lysates were centrifuged at 4°C for 15 mins at 12,000xg to remove cell debris. Each cleared lysate from the wild type and CoKO cells was divided into 3 individual aliquots. For the pre-clearing step, 50 μl of the protein A/G agarose bead (Cat. No. 2042, Thermo Scientific) was added to each tube and incubated for 2 hours at 4°C with gentle agitation. The samples were centrifuged at 14,000xg at 4°C for 10 mins, and the subsequent supernatants were used for the planned immunoprecipitation with 5 μg of anti-PEG3 antibody (Cat. No. ab99252, Abcam). After overnight incubation at 4°C with gentle agitation, 50 μl of the protein A/G agarose bead was added to each tube, and incubated for additional 4 hours at 4°C. After the incubation, the samples were centrifuged again to remove the supernatants, and the remaining beads were washed three times with the lysis buffer. The proteins were eluted from the beads with 50 μl of 5% acetic acid for 3 mins at room temperature. The recovery and quality of the immunoprecipitated proteins were analyzed through SDS-PAGE followed by silver staining.

The immunoprecipitation for Co-IP experiments used the following procedure. Transfected Neuro2a cells were washed on ice with PBS, and lysed in 1 ml of the Pierce IP Lysis Buffer. The subsequent lysates were cleared with the centrifugation at 4°C for 15 mins at 21,000×g. The cleared lysates were then subject to the immunoprecipitation using 1 μg of the monoclonal anti-FLAG antibody (Cat. No. MA1-91878, Thermo Scientific) or anti-HA Epitope Tag antibody (Cat. No. 26183, Thermo Scientific), followed by 20 μl of the goat anti-mouse IgG beads to collect the immune complexes. After the wash with the lysis buffer, the beads were heated in the SDS sample buffer. The eluted protein samples were separated by SDS-PAGE, and transferred to Immun-Blot PVDF membrane (Bio-Rad). The membranes were blotted with the monoclonal antibodies against KAP1 (Cat. No. ab10484, Abcam), LSD1 (Cat. No. ab37165, Abcam), or HA Epitope Tag, and then incubated with the HRP-conjugated secondary antibody. The target proteins were visualized using North2South Chemiluminescent Substrate (Cat. No. 17295, Thermo Scientific) in the Chemidoc XRS documentation system (Bio-Rad)

### Double immunofluorescence staining for co-localization experiments

HeLa cells were transiently transfected with PEG3-FL-FLAG and KAP1-HA expression constructs. After 48 hour post-transfection, the cells were fixed in 4% paraformaldehyde for 15 minutes. The first primary antibody, anti-FLAG (Cat. No. MA1-91878, Thermo Scientific) was diluted 1:1000 in blocking solution (Cat. No. B40942, Invitrogen) overnight in 4°C with gentle rocking. After three washes in PBS, the cells were incubated at room temperature for 1 hour in HRP conjugated anti-mouse secondary antibody (Cat. No. B40942, Invitrogen) followed by 5 minute incubation in Alexa Fluor tyramide (Cat. No. B40942, Invitrogen). Subsequently, the cells were incubated in the second primary antibody, anti-HA tag antibody (Cat. No. 26183, Thermo Scientific), diluted 1:1000 in blocking solution overnight in 4°C with gentle rocking. The Alexa Fluor 488 (Cat. A11001, Invitrogen) were used as the subsequent secondary antibody at 1:200 dilution. The cells were imaged on Leica DM2500 microscope.

### Protein identification by LC-MS/MS

The immunoprecipitated proteins with the anti-PEG3 antibody were separated on an SDS gel, reduced with DTT, alkylated with iodoacetimide, and digested in-gel with trypsin. The digested peptides were extracted from the gel, labeled with the TMT (Tandem Mass Tag) reagent, and analyzed on an Orbitrap Fusion mass spectrometer (Thermo Fischer Scientific). In brief, the peptides were separated using a gradient of 3 to 23% acetonitrile in 0.125% formic acid over 180 minutes, detected (MS1) and quantified (MS3) in the Orbitrap. The peptides were further sequenced (MS2) in the ion trap. MS2 spectra were searched using the SEQUEST algorithm against the UniProt composite database derived from the mouse proteome containing its reversed complement and known contaminants. The peptide spectral matches were filtered to a 1% false discovery rate (FDR) using the target-decoy strategy combined with linear discriminant analysis. The proteins were filtered to a <1% FDR, and the proteins were quantified only from the peptides with the summed SN threshold of > = 100 and the isolation specificity of 0.5.

### Chromatin ImmunoPrecipitation (ChIP)

Chromatins were prepared from MEF according to the method previously described [24]. In brief, the homogenized samples were first cross-linked with 1% formaldehyde for 20 mins, and then lysed with the buffer containing protease inhibitor cocktail (Cat. No. 539131, Millipore). The released nuclei were fractionated with sonication to derive a pool of DNA fragments size-ranging from 300 to 500 bp in length. The prepared chromatin was immunoprecipitated with a commercial antibody against KAP1 (Cat. No. ab10484, Abcam). The immunoprecipitated DNA was dissolved in 50 μl of TE for PCR analyses.

### Promoter assays

The reporter assay used the following constructs. The modified version of the β-gal vector was used as a control vector monitoring transfection efficiency [33]. The vector designed to test promoter activity was modified from a commercial luciferase vector (PGL3, Promega). This modified vector is promoterless, thus no promoter activity without any inserted DNA (Empty vector). The promoter region of *H19*, 610 bp in length, was inserted into the *Not*I site of the modified luciferase vector (H19-prom). For the luciferase reporter assay, Neuro2a and HEK293 cells were cultured in DMEM medium with 10% fetal bovine serum, and plated in 6-well plates for transfection. For the control, the cells on one well were transfected with 1.0 μg of the β-gal vector and 1.0 μg of the Empty vector. For the luciferase assay, the cells on each well were also co-transfected with 1 μg of the β-gal vector, 1 μg of each luciferase vector and 0.5 μg of the expression vector containing the full-length or mutant versions of PEG3. Fresh complete media was added 6 hours after the transfection, and the total cell lysates were harvested 48 hours later in 300 μl of the cell lysis buffer according to the previously published protocol [33]. The luciferase assay was performed in triplicates.

## Supporting Information

S1 File This file contains amino acid sequences of 10 representative mammalian PEG3. (RTF)

S2 File This file contains the output of amino acid sequence alignment using 10 mammalian PEG3s. (PDF)

S3 File This file contains the output of tandem mass spectrometry, which has been derived from WT and KO MEFs. (PDF)

S4 File This file contains additional images that were derived from the immunostaining of the cells that were co-transfected with PEG3-FL-FLAG and KAP1-HA constructs. (TIF)
