# Supplementary material for: PEG3 Interacts with KAP1 through KRAB-A
Source: PLoS One. 2016 Nov 29;11(11):e0167541. doi: 10.1371/journal.pone.0167541 (PMC5127583; doi:10.1371/journal.pone.0167541)
Supplement: S1 File — (RTF) [file pone.0167541.s001.rtf]

>Mouse_Peg3MYHHEDDTNSDMNSDDDMSRSGRETPPPRPSHAFGSERDLERRGRSRDVEPRDRWPYTRNPRSRLPQRDLSLPVMSRPHFGLDRDDDRRSMDYESRSQDAESYQNVVELKEDKKPQNPIQDNLENYRKLLSLGVQLAEDDRHSHMTQGHSSRSKRTAYPSTSRGLKPMPEAKKPSHRRGICEDESSHGVIMEKFIKDVARNPKSGRARELNERPPPRFPRPNDNWKDSSSSRRESVIQERGYEGSAFRGGFRFNADLASRSRALERKRRYHFDSDERGSGHEHKSCVRKKPFECGAEMRQAMSMGNLNSPSFSESQSIDFGANPYVCDECGRQFSVISEFVEHQIMHTRENLYEYGESFIHSVAVNEVQKGQGGGKRFECKECGETFSRSAALAEHRQIHAREYLAECRDQEDEETIMPSPTFSELQKMYGKDKFYECKVCKETFLHSSALIEHQKIHGRGNSDDRDNERERERDRLRARAREQRERERERERERELGEPFLTCPNFNEFRKMYRKDKIYECKVCGESFLHLSSLREHQKIHTRGNPFENKSRMCEETFVPSQSLRRRQKTYREKLFDFNNARDALMGNSDSSEHQKNRSRRNFFEGRGFEKPFVESQKSHTITRPPENKDDDKPFTISVNPNDKLKFPIMENGSQGKSYERSVIHSLGSAEAQKSHGGLGFSKPRPVAESSTQSSSSIYYPRAHSGGNTYEGKEYKDSIIHSLPAPRPLKRHRANDHIQCDEGGESSIYIPDIINKGRKIPAREDAYEGSSSSNYHTPNVSRAEPPSLSGESHDSKQDVTFSVPSSSVREHQKARAKKKYIEPRNNETSVIHSLPFGELLAGHRRAKFFECQECGEAFARRSELIEHQKIHDRERPSGSRHYERSVIRSLAPSDPQTSYAQERFIQEQVRKFRAFGQRSTTSNNLSVQKIYAQETFNAEEPHDKETHGQKIHDKEPYGKEPSGKEPHGDEPQDKEPLDQEMRSEEPHDDKPHGQEPHDDKPHGQEPHDDKPHGQEPHGDEPHGQEPHGDEPHDKEPIDQEMRSEEPHSEESHGDEPHGEESHGQEKVEDATIQASVSEEHQKDDAGDAIYECQDCGLGFTDLNDLTSHQDTHSRKALVDSREYAHSEVHAHSVSEFEKKCSGEKLYECPKCGESFIHSSLLFEHQRVHEQDQLYSVKACDDAFIALLPVRPRRNCTVERNPAVSGSAIRCRQCGQGFIHSSALNEHMRQHRDNEIMEQSELSDEIFIQGLALTEYQGSETEEKLFECTICGECFFTAKQLGDHHTKVHKDEPYEYGPSYTHASFLTEPLRKHIPLYECKDCGQSFLDDTVIAERMVFHPEREGGSEIVAATAQEVEANVLIPQEVLRIQGSNAEAAEPEVEAAEPEVEAAEPEVEAAEPNGEAEGPDGEAAEPDGEAEQPNGEAEQPNGDADEPDGAGIEDPEERADEPEEDVEEPEGDADEPDGADIEDPEEEGEDQEIEVEEPYYNCHECAETFASSSAFGEHLKSHASVIIFEPANAPGECSGYIERASTSAGGAEQADDKYFKCDVCGQLFNDRLSLARHQNSHTG>Rat_Peg3MYHHEDDTNSDMNSDDDMSRSGRETPPPRPSHAFSSERDLERRGRSRDVEPRDRWPYTRNPRSRMPQRDLSLPVMSRPHFGLERDDDRRSMDYESRSQDAESYQNVVELKEDKKPQNPIQDNLENYRKLLSLGVQLAEDDRHSHMTQGHSSRSKRTAYPSTSRGLKPMPEAKKPSHRRGICEDESSHGVIMEKFIKDVSRNPRSGRARELNERPPPRFPRPNDNWKDSSSNKRESVIQERGYEGSSFRGGFRFNADLVSRSRALERKRRYHFDSEERGSGHEHKSCVRKKPFECGSEMRQAMSMGNLRNPSLSESQSVDFGANQYVCDECGRSFSVISEFVEHQIMHTRENLYEYGESFIHSVAVNEVQKGQGRGKRFECKECGETFSRSAALAEHRQIHAREYLAECRDQEDEETVMPSPTFSELQKMYGKDKFYECKVCKETFLHSSALIEHQKIHGRGNSDDRDNERERERDRLRARAREQREREREREREREREHEHGEPFLTCPNFNEFRKMYRKEKIYECKVCGESFLHLSSLREHQKIHTRGNPFENKSRVCEETFVPSQSLKRRQKTYREKLFDFNNARDALMGSSDPSEHQKNRSRKNFFEGRGFEKPFVESQKSHTITRPPENREDDKPFTISVNPNDKLKFSAMESSSQGKSYERSVIHSLGSPEAQKSHGALGFSKPKSVTESSTQTSSSINYRRTHSGGITYEGKEYKGSIIHSLPAPRPLKRHRVSDQIQCDEEGESSIYIPDIIKRRKIPAREDAYEGSSSSSYHTASVPRAEPPSVSGESRESKQDVTFSVPSSSVREHQKARAKKKYIEPRSNETSVIHSLPFGEFLAGHRRAKFFECQECGEAFARRSDLIEHQKIHDRERPSGSRHYERSVIRSLAPSDPQTSYAQERFIQEQVRKFRAFGQRSTTSNNLSVQKIYAQEKFNAEEPHDKETHGQKIHDKEPYGKEPSGQDPHGDEPQDKEPQDKEPQDKEPQDKEPQDKEPQDKEPQDKEPQDKQPQDKEPQDKEPLDQEMRSEEPHGDQPHGQEPHGDEPHDKEPVDQEMPSEEPQGEESHGQEKAEDITIETSVSEEPQKDDAGDAIYECQDCGLGFADLNDLTSHQDVHSRKSLVDSREYTHSEVHVHSVSEFEKKYSGEKLYECPKCGESFIHSSLLFEHQRVHEQDQTYSVKACDDGFIALLPARPRRNCTVERNPAVSGSAIRCRQCGQGFIHSSALNEHMRQHRDNEILEQNELADEIFIQGLALTEYQGSETEEKLFECTICGECFFTAKQLGDHHTKVHKDEPYEYGPSYTHASFLTEPLRKHIPLYECKDCGQSFLDDTVITERMVFHPEREGGSEIVAATAQEVEANVLIPQEVLRIQGSNAEAAEPEVEAAEPEVEAAEPEVEAAEPNGEAEGPDGEAAEPDGEAEQPNGEAEQPNGDADEPDGAGIEDPEERADEPEEDVEEPEGDADEPDGADIEDPEEEGEDQEIEVEEPYYNCHECAETFASSAAFGEHLKSHASVIIFEPANALGECSGYIERASTSAGGAEQADDKYFKCDVCGQLFNDRLSLARHQNSHTG>Rabbit_Peg3MYQPEDDNNSDIPSDDDMSRNGGETPPPRSEHSFSSGSGLERRGRSRNVEPRDCWSYPRNPRSRLLQRDLSLPVMAKTSFEMEREDDRDSMDYEPPSQDVESYQNVMDLNEDRKPQNPIQDNMENYRKLLSLGVQLAEDDGHSHMTQGHSSRSKRSAYPSTSRLKTIPEAKKSTHRRGICEDESSHGVIMEKFIKDVSRASKSGRARESTDRPQRFPRMSDESWKDVSFTKRESVIQERGYEGNEFRGGFRFNSNLVSRRRVLERKRRYHFDTDGKGSTWDQRARKKPFECGSEMRKALSMSSLSSISSSSFSGSQPADFGAMPYVCDECGRSFSVISEFVEHQIMHTRENLYEYGESFIHSVAVSEVQKSQAGGKRFECKECGETFNKSATLAEHRKIHAREYLAESNDQEYEEAFMPSPTFSELQKIYGKDKFYECKVCKETFLHSSALIEHQKTHGRGNFSDDKDNERERQREHERGEAFMPSSAFNEFQKMYGKEKIYECKVCGETFLHSSSLREHQKIHTRGNPFENKGKICEETFIPGQSFKRRQKTYTKEKLYDFTDGRDAFLSSDISEHQKIHSRKNLFESRGYEKPVIHNMPFTESQKSHTITRPPENEEEEKAFTISSNPSGNQKFPLRENVYERKPYERSVIHSLASTAAQRSYIAVGPSKLKVIAESTFQSSNVISYQKIRTGGNTYEGKEYKRSVIHSLAAPRPLKRHIVSDLGGCDERGESSIYISDLSNKRQKIPARENPYESDVNNSHEDSVLQSVSYVRPQKSLAGEGSSELKQDGEFSVPSSSVRQHQKARAKKKYIEHRSNETSVIHSLPFGELQRIRPRERLYECQECGESFARISDLTEHQKIHDREKPSGSKNYEQSVIRSLAPTDPQTSYAQEQYAEEQARNEFKFRQRFTNSNNLSTHQKIYAQEKSHGEEPRGEEPRGKEPHGEETHGEQTPEEMNPEEETHGQETPEEETRGQETPGKETRGQETPGEETRGQETPGEETRGQETAGQETRGQETPDEETRGQETPDETHGEETHGEKTPGEETRGQETPGEETHGEETVEDAVIQGSDLDEPQKDDPDNTIYECQDCGLGFVDLTDLTDHQNVHSTNTKCLVDSREYTHSVIHTHSISEYQREYTGEQLYECPKCGESFIHSSFLFEHQRVHEQDQLYALKGCDDGFISLLPVKPRRNRAAERNPALAGSAIRCLQCGQGFIHSSALNEHMRLHREDELLEQSEMAEEAIMSGLALTEFQGSESEEKLFECTICGECFFTASELGDHHIKVHKDEPYDYGPSFTHTSFLTEPLKGAIPFYECKDCGKSFVHSTVLTKHKELHPEEDDDDDDDDDSAQAVEANVIVPQEVLRIQGSNVEAAEPEVEAAEPEVEAAEPEVEAAEPNGEAEGPDGEAAEPNGEAEQPNGEAGEAEQPEGDADEPDGVGIEDPEERVEEPEGDADEPDGAGVEDPEEEGEDQEIEIEEPYYNCQECTETFTSSAAFGEHLKTHASVIVFEPADAFGECSGYIERASTSTSTSTSTSTSTSAGGADQADEKYFKCDVCGQLFNDRLSLARHQNTHTG>Human_PEG3MYQPEDDNNSDVTSDDDMTRNRRESSPPHSVHSFSPGLGRRGRSRDMEPRDRWSHTRNPRSRMPPRDLSLPVVAKTSFEMDREDDRDSRAYESRSQDAESYQNVVDLAEDRKPHNTIQDNMENYRKLLSLGVQLAEDDGHSHMTQGHSSRSKRSAYPSTSRGLKTMPEAKKSTHRRGICEDESSHGVIMEKFIKDVSRSSKSGRARESSDRSQRFPRMSDDNWKDISLNKRESVIQQRVYEGNAFRGGFRFNSTLVSRKRVLERKRRYHFDTDGKGSIHDQKGCPRKKPFECGSEMRKAMSVSSLSSLSSPSFTESQPIDFGAMPYVCDECGRSFSVISEFVEHQIMHTRENLYEYGESFIHSVAVSEVQKSQVGGKRFECKDCGETFNKSAALAEHRKIHARGYLVECKNQECEEAFMPSPTFSELQKIYGKDKFYECRVCKETFLHSSALIEHQKIHFGDDKDNEREHERERERERGETFRPSPALNEFQKMYGKEKMYECKVCGETFLHSSSLKEHQKIHTRGNPFENKGKVCEETFIPGQSLKRRQKTYNKEKLCDFTDGRDAFMQSSELSEHQKIHSRKNLFEGRGYEKSVIHSGPFTESQKSHTITRPLESDEDEKAFTISSNPYENQKIPTKENVYEAKSYERSVIHSLASVEAQKSHSVAGPSKPKVMAESTIQSFDAINHQRVRAGGNTSEGREYSRSVIHSLVASKPPRSHNGNELVESNEKGESSIYISDLNDKRQKIPARENPCEGGSKNRNYEDSVIQSVFRAKPQKSVPGEGSGEFKKDGEFSVPSSNVREYQKARAKKKYIEHRSNETSVIHSLPFGEQTFRPRGMLYECQECGECFAHSSDLTEHQKIHDREKPSGSRNYEWSVIRSLAPTDPQTSYAQEQYAKEQARNKCKDFRQFFATSEDLNTNQKIYDQEKSHGEESQGENTDGEETHSEETHGQETIEDPVIQGSDMEDPQKDDPDDKIYECEDCGLGFVDLTDLTDHQKVHSRKCLVDSREYTHSVIHTHSISEYQRDYTGEQLYECPKCGESFIHSSFLFEHQRIHEQDQLYSMKGCDDGFIALLPMKPRRNRAAERNPALAGSAIRCLLCGQGFIHSSALNEHMRLHREDDLLEQSQMAEEAIIPGLALTEFQRSQTEERLFECAVCGESFVNPAELADHVTVHKNEPYEYGSSYTHTSFLTEPLKGAIPFYECKDCGKSFIHSTVLTKHKELHLEEEEEDEAAAAAAAAAQEVEANVHVPQVVLRIQGLNVEAAEPEVEAAEPEVEAAEPEVEAAEPNGEAEGPDGEAAEPIGEAGQPNGEAEQPNGDADEPDGAGIEDPEERAEEPEGKAEEPEGDADEPDGVGIEDPEEGEDQEIQVEEPYYDCHECTETFTSSTAFSEHLKTHASMIIFEPANAFGECSGYIERASTSTGGANQADEKYFKCDVCGQLFNDRLSLARHQNTHTG>Chimp_Peg3MYQPEDDNNSDVTSDDDMTRNRRESSPPHSVHSFSPGLGRRGRSRDMEPRDRWSHTRNPRSRMPQRDLSLPVVAKTSFEMDRDDDRDSRAYESRSQDAESYQNVVDLAEDRKPHNTIQDNMENYRKLLSLGVQLAEDDGHSHMTQGHSSRSKRSAYPSTSRGLKTMPEAKKSTHRRGICEDESSHGVIMEKFIKDVSRSSKSGRARESSDRSQRFPRMSDDNWKDISLNKRESVIQQRVYEGNAFRGGFRFNSTLVSRKRVLERKRRYHFDTDGKGSIHDQKACPRKKPFECGSEMRKAMSMSSLSSLSSPSFTESQPIDFGAMPYVCDECGRSFSVISEFVEHQIMHTRENLYEYGESFIHSVAVSEVQKSQVGGKRFECKDCGETFNKSAALAEHRKIHARGYLVECKNQECEEAFMPSPTFSELQKIYGKDKFYECRVCKETFLHSSALIEHQKIHFGDDKDNEREHERERERGETFRPSPALNEFQKMYGKEKMYECKVCGETFLHSSSLKEHQKIHTRGNPFENKGKVCEETFIPGQSLKKRQKTYNKEKLYDFTDGRDAFMQSSELSEHQKIHSRKNLFEGRGYEKSVIHSGPFTESQKSHTITRPLESDEDEKAFTISSNPYENQKIPTKENVYEAKSYERSVIHSLASVEAQKSHSVAGPSKPKVMAESTIQSFDAINHQRVRAGGNTSEGREYSRSVIHSLVASKPPRSHNGNELVESNEKGESSIYISDLNDKRQKIPARENPCEGGSKNRNYEDSVIQSVSRAKPQKSVPGEGSGEFKKDGEFSVPSSNVREYQKARAKKKYIEHRSNETSVIHSLPFGEQTFRPRGMLYECQECGECFAHSSDLTEHQKIHDREKPSGSRNYEWSVIRSLAPTDPQTSYAQEQYAKEQAWNKCKEFRQFFATSEDLNTNQKIYDQEKSHGEESQGENTDGEETHSEETHGQETIEDPVIQGSDMEDPQKDDPDDKIYECEDCGLGFVDLTDLTDHQKVHSRKCLVDSREYTHSVIHTHSISEYQRDYTGEQLYECPKCGESFIHSSFLFEHQRIHEQDQLYSMKGCDDGFIALLPMKPRRNRAAERNPALAGSAIRCLLCGQGFIHSSALNEHMRLHREDDLLEQSQMAEEAIIPGLALTEFQRSQTEERLFECAVCGESFINPAELADHVTVHKNEPYEYGSSYTHTSFLTEPLKGAIPFYECKDCGKSFIHSTVLTKHKELHLEEEEEDEAAAAAAAAAQEVEANVHVPQVVLRIQGSNVEAAEPEVEAAEPEVEAAEPEVEAAEPNGEAEGPDGEAAEPIGEAGQPNGEAEQPNGDADEPDGAGIEDPEERAEEPEGKAEEPEGDADEPDGVGIEDPEEGEDQEIQVEEPYYDCHECTETFTSSTAFGEHLKTHASMIIFEPANAFGECSGYIERASTSTGGANQADEKYFKCDVCGQLFNDRLSLARHQNTHTG>Marmoset_Peg3MYQPEDDNDVTSDDEMTRNRGESPPPRSVYSFSPGLGRRGRSRDREPRGRWSYTRNPRSRMPQRDLSLPVMAKTSFEMERDDDRDSRAYESRSQDAESYQNMVNLTEDRKPHNTIQDNMENYRKLLSLGVQLAEDDGHSHMTQGHSSRSKRSAYPSTSRGLKTMPEAKKSTHRRGICEDESSHGVIMEKFIKDVSRSSKSGRARESSDRSQRFPRMSDDNWKDLSLNKRESVIQERVYEGNSFRGGFRFNSTLVSRKRILERKRRYHFDTDGKGSTHDQKACPRKKPFECGSEMRKAMSMSSLSSLSSPSFTESQPVDFGAMPYVCDECGRSFSVISGFVEHQIMHTRENLYEYGESFIHSVAVSEVQKSQIGGKRFECKDCGETFNKSAALAEHRKIHARDYLVECKDQAYEEAFMPSPTFSELQKIYGKDKFYECRVCKETFLHSSALIEHQKIHFGDGKDNDRERERERERERGEAFMPSSTLNEFQKMYGKEKMYECKVCGETFLHSSSLKEHQKIHARGNPFENKGKVCEETFIPGQSLKRRQKTYNKEKLYDFTDGRDAFMQSSELSEHQKIHSRKNIFEGRGYEKSVIHSMPFTESQKSHTITRPPESDEDEKPFTISSNPFENQIPTKENVYEGKSYERSVIHSLASVEAQKSHSVAGPSKPKVMAESTIQSFEAINHQRVRAGGNTSEGRGYSRSVIQRLVASKLPKSHNGNELVESNENGESSIYISDLNGKRLKIAARENPCEGGSKSHNYEDSVIQSVSHAKPQKSVSGKGSGEFKKDGEFSVPSSNVREYQKARAKKKYIEHRSNETSVIHSLPFGKQQTHRPRGMLYECQECGECFAHRSDLTEHQKIHDREKPSGSRNYEWSVIRSLAPTDPQTSYAQEQYAKEQARNKYKEFRQSFATNKDLNTHQKNYDQEKSHGKKSQGKKTHGEETHGKKSHGEKSNGEKTQDEEMHGEETHGQEIIEDPVIQGSDMEEPQKDDPDDTIYECEDCGLGFVDLTDLTDHQKVHRRKCLVDSREYTHSVSHTHSISEYQRNYTGEQLYECPKCGESFIHSSFLFEHQRIHEQDQLYSLKGCDDGFIALLPVKPRRNRAAERNPALAGSAIRCLLCGQGFIHSSALNEHMRLHREDDLLEQSQMAEEAIIPGLALAEFQRSQTEERLFECAVCGESFVNAAELADHVTVHKNEPYEYGSSYTHTSFLTEPLKGAMPFYECKDCGKSFIHSTVLTKHKELHLEEEDDDDDDEAAGAAAQEVEANVHVPQEVLRIQGSNVEAAEPEVEAAEPEVEAAEPEVEAAEPNGEAEGPDGEAAEPNGEAEQPNGEAEQPNGDADEPDGAGIEDPEERAEEPEGKAEEPEGDADEPDGAGIEDPEEGEDQEIQVEEPYYDCHECTETFTSSAAFGEHLKTHASMIIFEPANAFGECSGHIERASTSTGGADQADEKYFKCDVCGQLFSDRLSLARHQNTHTG>Dog_Peg3MYEPGDDNNSDLHSEDSMSRKGAESPPPRSASSFCGRGRSRDLESRDRWPYTRNPRSRLPQRDLSLPLMEKTSFAMERECNRDSMDYESRSQDAVSYQDVVNLTEDRKPQNPIQDNMENYRKLLSLGVQLAEDDGHSHMTQGHSSRSKRGAYPSTSRGLKTTPETKKLAHRRGICEDESSHGVIMEKFIKDVSRNSKSGRARESNDRSQRFPRRPENDWKGVSFNKRESVIQERGYEGNAFGGGFNMNSSLVSKKRVLERKRRYQFDTDGKGSVHEQKGYARKRPFECSEMRKAMSMSSLSAPSFTESHPFDFGAMPYVCDECGRSFSVISEFVEHQIMHTRENLYEYGESFIHSVAVSEVQKSQAGGKRFECKECGETFSKSTALAEHRKIHAREHLAECNDEEYEEPFMPSPTFSELQKIYGKDKFYECKVCKETFLHSSALIDHQKTHGRDDKDNERGEAFKPSLNELQKMYGKEKMYECKVCGETFHHSSSLKEHQKIHTRGNLFENKGKVCEETFIPGQSLKRHQKSYSKEKLYDFKDGGDAFRQSSDLSEHQKIHSRKNLYEGRGYEKSVIHSVPFTESQKSHTITRPPENEEDEKAFTISSNPDDNQKIPTKENACERKPYERSVIHSLAFAKAQKSCHSAVGPSKPQVIAESATQTSGVIEHQKVHAGENSEGKKYETSVIHTLAAFKPPKNCSGNEVVQCDEKGESSTYLSNLCDKQQKTPARETPYEGAKSNNQKDSVIQSVSRIEPQKSLPSQGSSESSIPSSNVREHQKARAKKKNIEHRNYETSVIHSLRFGEPQTFRPREKFYECPECGESFVRISDLTEHQKIHDRKKPSGSKNYERSVIRSLASTDPQTSYAEQQAQTSYAEHSNSRQMRYPEQPAQTSYTKQPAQASYTKQPAQASYAKQPAQASYSAHPVHLSYSEHPVRMSYTEQPGRVSYAQQPAQMNYTEEQAQTSFAEQQVHNKCKECGECFATLEELGAHQKIYAREEFHGRKLFGNSVIQGVGLDGPRLVESRPEEPRQEEPDEQDEPDEQDEPEDTIYGCKDCGLGFAHRADLKDHQKVHGREYLIDSREYTHSVIHTHSVSEYQKDYIGEQLYECPACGESFVHSSFLFEHQKIHEQDQFYGHRRYDEPFVQPLVINPRRPRAPQKNPPAGTSLQCHVCGQDFIHGSVLSEHMRIHTGEDLPEQGQRSEDAVSPGLALTEFQRSQTEEKHYECKTCGESFLNQADLREHMRIHEKDEPYDYGASFVHTSFLTEPPKRDSPFYECKDCGKSFIHNTVLNKHQKLHLEEEEEEGAQEVEANVLVPREVLRIQGSNVEAAEPEVEAAEPEVEAAEPNVEAAEPNGEAEGPDGEAAEPNGEAEQPNGEAEQPNGDADEPDGAGIEDPEERAEEPEGKAEEPEGDADEPDGAGIEDPEEEGEDQEIQVEEPYYDCRECGETFTSNSAYGEHLKTHARVIIFEPGNVYGESSHYTEHASTSTSDNDRADDKYFKCDVCGQLFSDRLSLARHQNTHTG>Cat_Peg3MYEPGDDNNSDLRSEDSMSRKGAESPPPRSASSFCGRGRSRDLGSRDRWPYPRNPRGRLPQRDLSLPLMEKTTFATERERNRDSMDYESRSQDAVSYQDVVDLTEDREPQNPIQDNMENYRKLLSLGVQLAEDDGHSHMTQGHSSRSKRSAYPSTSRGLKSMPETKKSTHRRGICEDESSHGVIMEKFIKDVSRNSKSGRARESNDRSQRFPRRPDSDWKEVSFNKRESVIQERGYEGNGFGGGFNFNSSLVSKKRVLERKRRYQFDTDGKGSAHEQKGYARKRPFECSEMRKAMSMSSLSAPSFTESQPLDFGAMPYVCDECGRSFSVISEFVEHQIMHTRENLYEYGESFIHSVAVSEVQKSQAGGKRFECKECGETFNKSAALAEHRKIHAREHLAECNDEEYEEPFMPSPTFSELQKIYGKDKFYECKVCKETFLHSSALIDHQKIHGRDDKDNERGEGFKPSPPPNDLPKTYGKEKMYECKVCGETFHHSSSLKEHQKIHTRGNLFESKGKVCEETFIPGQSLKRRQKTYPKEKLYDFTDGGDAFRQSSDLSEHQKIHSRKNLFEGRGYEKSVIHSVPFTESQKSHTITRPPEDDEDQKAFTVSSNPDDNQKVPPQENVYERKPYERSVIHSLAFAKAEKSHSAVGPSKPKVIAESTIQSSGVTEHQKAHAGENTSEGKKYERSVIHSVAAFKPPKSCNGNEVVECEEKGESSTSVSDRHDKQQKTPARENPNEGGKNNNYKDSVIQSVSHMESQKSPTSQGSSELKKDGESSTPTSNVREHQKARSKKKNIERRNYETSVIHSLRFGDHQTFRPREKFYECPECGESFVRSYDLTEHLKIHDRKKPSGSKNYERSVIRSLVSTDPQTSYAEQQPQTSYAGHSSQMRYSDQAAQTSYAKHPVQTSYSGMHMSYAVQPGHMSYTQQAAQTSYMVQPTQISYDEEQAQTSYAEQQVRNRCRECGECFATIGDLGAHQKIYAREEFHGRKLFGDTVIQGIGLEGPRPEEPRQNEPDEQDEQDEPEDAIYGCKDCGLGFADRADLKDHQKVHGREYLIDSREYTHSVIHTHSVSEYQKDYIGEQLYECPACGESFVHSSFLFEHQKIHEQDQFYGQRRYDEPFVQPLVINPRRPRAPQKNPTAGTSLQCHVCGQDFIHGSVLGEHMRIHTREDLPEQGQRSEDAVSPGLALTEFQRSQTEEKHYECKTCGETFLNQSDLREHMRIHEKDEPYDYGASFVHTSFLTEPPKRDSPFYECKDCGKSFIHNTVLTKHQKLHLEEEEEEGAQEVEANVLVPREVLRIQGSNVEAAEPEVEAAEPEVEAAEPNVEAAEPNGEAEGPDGEAAEPDGEAEQPNGEAEQPNGDADEPDGAGIEDPEERAEEPEGDADEPDGAGIEDPEEEGEDQEIQVEEPYYDCRECGETFASNSAYGEHLKTHARVIIFEPGNVYGESSRYTEHASTSTSDNDRADDKYFKCDVCGQLFSDRLSLARHQNTHTG>Horse_Peg3MYEPEDDDNSDVHSEDSMTRKVAESPPPRSVYSYGSVRARRRDLEPRDRWPYTRNPRGRLPQRDLSLPLMEKTTFITEREHNRDSMEYESRSQDAVSYQDVVDLTEDRKPQNPIQDNMENYRKLLSLGVQLAEDDGHSHMTQGHSSRSKRNAYPSTSRGLKTMPETKKSTHRRGICEDESSHGVIMEKFIKDVSRNSKSGRARESNDRSQRFPRRPDNGWKEVSFNKRESVIQERGYEGNAFGGGFNFNSHLVSRKRVLERKRRYHFDTDGQGSIHDQKGYPRKRPFECNDMRKAMSMSSLSSPSFTESQPLDFGAMPYVCDECGRPFSVISEFVEHQIMHTRENLYEYGESFIHSVAVSEVQKSQAGGKRFECKECGETFNKSAALAEHRKIHAREYFSECKDEEYEEPFMPSPTFSELQKIYGKDKFYECKVCKETFLHSSALIDHQKIHGRNDKDNDRGEAFKLSPTLSELQKMYGKEKMYECKVCGETFRHSSSLKEHQKIHTRGNLFEKKGKVCEETFIPGQSLKRRQKTYSKEKFYDFKDGGDAFRKSSDLNVHQKIHSRKHLYEGRGYEKSVFHGLSFTESQKSHTITRPPENEEDEKAFTISSNPDDNQTFPIKDNVSEGKPYERSVIHSLASAEAQKSHSAAGPNKLKVIAESVIQSSNVTEHQKVYAGENTSDRKKYERSVIHSLATFRPPKSCDGNELIECNEKAESSIYVSDLHDKQQKTPARQNPYEGDKNNSYKDSVIHSMSHTKPQKSLTGEESSEFKKDGESSVVNSNVREHQKARAKKKNIERRNYETSVIHSLSFGENQTFRPREKFYECPVCGESFVRNSDLTEHQKIHDRKKPSGSKNYERSVIRSLASTDPQTSYAEQPAQTSYAEQPAQMNPAEQPSETSYAEQQVRKKCKECGQSFATTEELRAHQKIYAREEFHGGNLFGGSVIQGVGLDGPQQGEPQQDKPDEQDELDEQDESEDTIYGCKDCGLGFADRADLKDHQKVHGREYLIDSCEYTHSVIHTHSVSEYQKDSIGDQLFECPACGESFVHSSFLFEHQKIHEQDQFFGHRRYDEPFMQPLIINPHRPRASQKNPPTGTSLQCCVCGRDFIHGSVLNEHMRIHTGEDLPEQGQRSEEAVSPGLALTEFQRSQTEEKHYECKTCGESFLNQSDLRDHMRIHEKDEPYDYGVTFLHTSFIAEPPKRDSPFYECKDCGKSFIHSTVLTKHQKLHLEEEEAAAAAQEVEANVLVPREVLRIQGSNVEAAEPEVEAAEPEVEAAEPNVEAAEPNGEAEGPDGEAAEPNGEAEQPNGEAEQPNVDADEPDGAGIEDPEERAEEPEGDADEPDGAGIEDPEEEGEDQEIQVEEPYYDCGECGETFASTSAYGEHLKTHARVIIFEPGNVYGESSHYTEHASTSTSDNDRADDKYFKCDVCGQLFNDRLSLARHQNTHTG>Cow_Peg3MYEPEDDNSSDTHSEGGMSRRAAESPPPRPALPCCSERERRRGRSRDMESRDRWPSVRSPRSRFHQRDLALPLAERAKEREHRRRDSLLDLDARSEEAVLYQDMVALTEDRKPQNPIQDNMENYRKLLSLGVQLAEDDGHSHMTQGHSARSKRSAYPSTSRGLKTAPETKKSAHRRGICEAESSHGVIMEKFIKDVARSSRSGRARESSERPHRLSRRAGGDWKEASFSRREAGASERGPEGGAFGGGGFSCGSDLVSKKRALERKRRYHFDAEGQGPVHDPRGGARKRPFECGGEARRAAKAAGASSLSAPPAAPSQPLDFGAMPYVCDECGRSFAVISEFVEHQIVHTRESLYEYGESFIHSAAVSEAQSRPEGARRSEGAQAAGLAEHRGGQAQEHLRGSGDEEQDEPFLPSPTFSELQKMYGKDKFYECKVCKETFLHSSALIEHQKIHSHEDREKERSTGAVRRTPMLGELQRACGKEKRYECKVCGETFHHSAALREHQKTHGRGSPSEGRARAFEETFIPGQSLKRRQKTYSKEKLYDFREGGDAFGRSSDFMEHQKIHSRKSYFDSRGYEKPLLHSMSMPGSQKSHTITRPPEDEDEEKAFTASSSPEDGQEARGYERSAYERAILHSLAAFRPPRGLREDGEPSTYLSGLRDPPQKTPAWESPYAGGRHSFFRSSVFYRASRPAPLDHLAGEGPSGWQRDGEASGPSSDGRQHQKARAKKKNIERKNYDASMMHSLHFGESQTFRPRERFYECLECGEFFVRSSELAEHQKIHNRKKLSGSKNYLRSVLRSLSSTDPQTSYQGQSVQMSYPQEAAQTSYAELAAQTSYAEEPAQTSYAVEPAQTSYAEEPAQTSYTEAPAEASYTEEPAQTSCIEEPAQTSYTNPAAETSYAEEPAQTSYTEAPAEASYTEEPAQTSCIEEPAQTSYTNPAAETSYTEEPAQTSYTEAPAEASGIEEPAQTNYTEESAEVSYTEEPSQTSCIEEPAQTSYTDPAAETSYTEEPAQTSYTQEPAQTSCTEEPAQTSCTEEPAQTSYTQEPAQTSYTKEPAEASYTEEPAQTSCIEEPAQTNYTKESAKASYTEEPAQTSYTDPAAETSYTEEPAQTNYTVESAEASYTEEPSQTSCIEEPAQTSYTDSAADTSCTEEPAQTSCTEEPAQTSYTQEPAQTSCTEEPAQTSCTEEPAQTSYTQEPAQTSCTEEPAQTSYTQEPAQTSCTEEPAQTSYTEEPAQTSYTEEPAQTSYTQEPAQTSCTEEPAQTSYTEEPAQTSYTEEPAQTSYTQEPAQTSYTEEPAQTSYTEEPAQTSYAQEPAQTSYAEEPAQTSYAEEPAQTSYAEEPAQTSYTQEPAQTNYTEEPAEASYTEEPAQTSYAEEPAQTSYPEEPAQTSYAEEPAQTSYAEEPAQTSYPEEPAQTSYTEEPAQTSYAKEPAQTSYPEEPAQTSYAEEPAQTSYAEEPAQTSYAEEPAQTSYSEEPAQTRYTGNELRSDMRKNQLRPDMPRNQLRPVMPRNQLRPDMPRNQPRPVILRNQLRPDMPRNQPRPVILRNQLRPDMLGNQLRPDMPGNQLRPDMLREPPAETSYAELVAQISYAELVTPTSYAELAAETGYFEPPAQTSYTEPAETNYADPAAQVSFDEPPAEASYADLAAEISYAELAAETSYADLAAQISYDEPPAETSYAELAAQISYSEPADQTSYAELAAQTSYSEPLAQTSYAELTSETSYCEQPVLNECKECGECFATVEDLGRHQKIYAREKFHDGKLFGEPVMQDLGLDGSPEEELEEQEEPEEPEDSIYGCKDCGLGFADRADLRDHQKVHGREYLVDSREYTHPAVHMPPVSEYQKDCLGEQLYECPACGESFVHSSFLFEHQKVHEQDQFYGHRRYEPFMQPLIVSPRRPQAPQKSAPAGVGPQCQVCGQDFIHASVLSEHARGHAGEGLPDQGQGGAGAAGPGPAPTEPQQDPGEEQRYECETCGESFPSQADLQEHMRVHEKGEPYDYGAAFVHTSFLTEPPKRDWPFYECKDCGKSFIHSTILTKHQKLHLQEEGAAAAAAATAQEAEANVLVPREVLRIQGSNVEAAEPEVEAAEPEVEAAEPEVEAAEPLGEAEGPEWEAAEPSGEAEQPHAEAEQPDMDADEPDGAGIEDPEERAEEPEGDDDEPDGAGIEDPEEEGEEQEIQVEEPYYDCGECGETFPSGAAYAEHLTAHASLVILEPAGLYGEGAGGPEGGRPDDELFKCDVCGQLFSDRLSLARHQNTHTG
