# Supplementary material for: PEG3 Interacts with KAP1 through KRAB-A
Source: PLoS One. 2016 Nov 29;11(11):e0167541. doi: 10.1371/journal.pone.0167541 (PMC5127583; doi:10.1371/journal.pone.0167541)
Supplement: S2 File — (PDF) [file pone.0167541.s002.pdf]

Supplemental data 2

```
         CLUSTAL W (1.83) multiple sequence alignment

Mouse    lcl|Query_10001      MYHHEDDTNSDMNSDDDMSRSGRETPPPRPSHAFGSERDLERRGRSRDVEPRDRWPYTRN
Rat      lcl|Query_10002      MYHHEDDTNSDMNSDDDMSRSGRETPPPRPSHAFSSERDLERRGRSRDVEPRDRWPYTRN
Rabbit   lcl|Query_10003      MYQPEDDNNSDIPSDDDMSRNGGETPPPRSEHSFSSGSGLERRGRSRNVEPRDCWSYPRN
Human    lcl|Query_10004      MYQPEDDNNSDVTSDDDMTRNRRESSPPHSVHSFSPGLG--RRGRSRDMEPRDRWSHTRN
Chimp    lcl|Query_10005      MYQPEDDNNSDVTSDDDMTRNRRESSPPHSVHSFSPGLG--RRGRSRDMEPRDRWSHTRN
Marmoset lcl|Query_10006      MYQPEDDN--DVTSDDEMTRNRGESPPPRSVYSFSPGLG--RRGRSRDREPRGRWSYTRN
Dog      lcl|Query_10007      MYEPGDDNNSDLHSEDSMSRKGAESPPPRSASSFC---G---RGRSRDLESRDRWPYTRN
Cat      lcl|Query_10008      MYEPGDDNNSDLRSEDSMSRKGAESPPPRSASSFC---G---RGRSRDLGSRDRWPYPRN
Horse    lcl|Query_10009      MYEPEDDDNSDVHSEDSMTRKVAESPPPRSVYSYG---S--VRARRRDLEPRDRWPYTRN
Cow      lcl|Query_10010      MYEPEDDNSSDTHSEGGMSRRAAESPPPRPALPCCSERE-RRRGRSRDMESRDRWPSVRS
                              **   **   *  *   * *   *  **              * * *    *  *   *

         lcl|Query_10001      PRSRLPQRDLSLPVMSRPHFGLDRDDDRRS--MDYESRSQDAESYQNVVELKEDKKPQNP
         lcl|Query_10002      PRSRMPQRDLSLPVMSRPHFGLERDDDRRS--MDYESRSQDAESYQNVVELKEDKKPQNP
         lcl|Query_10003      PRSRLLQRDLSLPVMAKTSFEMEREDDRDS--MDYEPPSQDVESYQNVMDLNEDRKPQNP
         lcl|Query_10004      PRSRMPPRDLSLPVVAKTSFEMDREDDRDS--RAYESRSQDAESYQNVVDLAEDRKPHNT
         lcl|Query_10005      PRSRMPQRDLSLPVVAKTSFEMDRDDDRDS--RAYESRSQDAESYQNVVDLAEDRKPHNT
         lcl|Query_10006      PRSRMPQRDLSLPVMAKTSFEMERDDDRDS--RAYESRSQDAESYQNMVNLTEDRKPHNT
         lcl|Query_10007      PRSRLPQRDLSLPLMEKTSFAMERECNRDS--MDYESRSQDAVSYQDVVNLTEDRKPQNP
         lcl|Query_10008      PRGRLPQRDLSLPLMEKTTFATERERNRDS--MDYESRSQDAVSYQDVVDLTEDREPQNP
         lcl|Query_10009      PRGRLPQRDLSLPLMEKTTFITEREHNRDS--MEYESRSQDAVSYQDVVDLTEDRKPQNP
         lcl|Query_10010      PRSRFHQRDLALPLAERAK---EREHRRRDSLLDLDARSEEAVLYQDMVALTEDRKPQNP
                              ** *   *** **          *   *          *     **    * **  * *

         lcl|Query_10001      IQDNLENYRKLLSLGVQLAEDDRHSHMTQGHSSRSKRTAYPSTSRGLKPMPEAKKPSHRR
         lcl|Query_10002      IQDNLENYRKLLSLGVQLAEDDRHSHMTQGHSSRSKRTAYPSTSRGLKPMPEAKKPSHRR
         lcl|Query_10003      IQDNMENYRKLLSLGVQLAEDDGHSHMTQGHSSRSKRSAYPSTSR-LKTIPEAKKSTHRR
         lcl|Query_10004      IQDNMENYRKLLSLGVQLAEDDGHSHMTQGHSSRSKRSAYPSTSRGLKTMPEAKKSTHRR
         lcl|Query_10005      IQDNMENYRKLLSLGVQLAEDDGHSHMTQGHSSRSKRSAYPSTSRGLKTMPEAKKSTHRR
         lcl|Query_10006      IQDNMENYRKLLSLGVQLAEDDGHSHMTQGHSSRSKRSAYPSTSRGLKTMPEAKKSTHRR
         lcl|Query_10007      IQDNMENYRKLLSLGVQLAEDDGHSHMTQGHSSRSKRGAYPSTSRGLKTTPETKKLAHRR
         lcl|Query_10008      IQDNMENYRKLLSLGVQLAEDDGHSHMTQGHSSRSKRSAYPSTSRGLKSMPETKKSTHRR
         lcl|Query_10009      IQDNMENYRKLLSLGVQLAEDDGHSHMTQGHSSRSKRNAYPSTSRGLKTMPETKKSTHRR
         lcl|Query_10010      IQDNMENYRKLLSLGVQLAEDDGHSHMTQGHSARSKRSAYPSTSRGLKTAPETKKSAHRR
                              **** ***************** ********* **** ******* **  ** **  ***

         lcl|Query_10001      GICEDESSHGVIMEKFIKDVARNPKSGRARELNERPPPRFPRPND-NWKDSSSSRRESVI
         lcl|Query_10002      GICEDESSHGVIMEKFIKDVSRNPRSGRARELNERPPPRFPRPND-NWKDSSSNKRESVI
         lcl|Query_10003      GICEDESSHGVIMEKFIKDVSRASKSGRARESTDRPQ-RFPRMSDESWKDVSFTKRESVI
         lcl|Query_10004      GICEDESSHGVIMEKFIKDVSRSSKSGRARESSDRSQ-RFPRMSDDNWKDISLNKRESVI
         lcl|Query_10005      GICEDESSHGVIMEKFIKDVSRSSKSGRARESSDRSQ-RFPRMSDDNWKDISLNKRESVI
         lcl|Query_10006      GICEDESSHGVIMEKFIKDVSRSSKSGRARESSDRSQ-RFPRMSDDNWKDLSLNKRESVI
         lcl|Query_10007      GICEDESSHGVIMEKFIKDVSRNSKSGRARESNDRSQ-RFPRRPENDWKGVSFNKRESVI
         lcl|Query_10008      GICEDESSHGVIMEKFIKDVSRNSKSGRARESNDRSQ-RFPRRPDSDWKEVSFNKRESVI
         lcl|Query_10009      GICEDESSHGVIMEKFIKDVSRNSKSGRARESNDRSQ-RFPRRPDNGWKEVSFNKRESVI
         lcl|Query_10010      GICEAESSHGVIMEKFIKDVARSSRSGRARESSERPH-RLSRRAGGDWKEASFSRREAGA
                              **** *************** *   ******   *   *  *     **  *   **

         lcl|Query_10001      QERGYEGSAFRGG-FRFNADLASRSRALERKRRYHFDSDERGSGHEHKSCVRKKPFECGA
         lcl|Query_10002      QERGYEGSSFRGG-FRFNADLVSRSRALERKRRYHFDSEERGSGHEHKSCVRKKPFECGS
         lcl|Query_10003      QERGYEGNEFRGG-FRFNSNLVSRRRVLERKRRYHFDTDGKGSTWDQRA--RKKPFECGS
         lcl|Query_10004      QQRVYEGNAFRGG-FRFNSTLVSRKRVLERKRRYHFDTDGKGSIHDQKGCPRKKPFECGS
         lcl|Query_10005      QQRVYEGNAFRGG-FRFNSTLVSRKRVLERKRRYHFDTDGKGSIHDQKACPRKKPFECGS
         lcl|Query_10006      QERVYEGNSFRGG-FRFNSTLVSRKRILERKRRYHFDTDGKGSTHDQKACPRKKPFECGS
         lcl|Query_10007      QERGYEGNAFGGG-FNMNSSLVSKKRVLERKRRYQFDTDGKGSVHEQKGYARKRPFEC-S
         lcl|Query_10008      QERGYEGNGFGGG-FNFNSSLVSKKRVLERKRRYQFDTDGKGSAHEQKGYARKRPFEC-S
         lcl|Query_10009      QERGYEGNAFGGG-FNFNSHLVSRKRVLERKRRYHFDTDGQGSIHDQKGYPRKRPFEC-N
         lcl|Query_10010      SERGPEGGAFGGGGFSCGSDLVSKKRALERKRRYHFDAEGQGPVHDPRGGARKRPFECGG
                                *  **   * ** *     * *  * ******* **    *         ** ****

         lcl|Query_10001      EMRQAMSMGNLNS---PSFSESQSIDFGANPYVCDECGRQFSVISEFVEHQIMHTRENLY
         lcl|Query_10002      EMRQAMSMGNLRN---PSLSESQSVDFGANQYVCDECGRSFSVISEFVEHQIMHTRENLY
         lcl|Query_10003      EMRKALSMSSLSSISSSSFSGSQPADFGAMPYVCDECGRSFSVISEFVEHQIMHTRENLY
         lcl|Query_10004      EMRKAMSVSSLSSLSSPSFTESQPIDFGAMPYVCDECGRSFSVISEFVEHQIMHTRENLY
         lcl|Query_10005      EMRKAMSMSSLSSLSSPSFTESQPIDFGAMPYVCDECGRSFSVISEFVEHQIMHTRENLY
         lcl|Query_10006      EMRKAMSMSSLSSLSSPSFTESQPVDFGAMPYVCDECGRSFSVISGFVEHQIMHTRENLY
         lcl|Query_10007      EMRKAMSMSSLSA---PSFTESHPFDFGAMPYVCDECGRSFSVISEFVEHQIMHTRENLY
         lcl|Query_10008      EMRKAMSMSSLSA---PSFTESQPLDFGAMPYVCDECGRSFSVISEFVEHQIMHTRENLY
```

```
lcl|Query_10009               DMRKAMSMSSLSS---PSFTESQPLDFGAMPYVCDECGRPFSVISEFVEHQIMHTRENLY
lcl|Query_10010               EARRAAKAAGASSLSAPPAAPSQPLDFGAMPYVCDECGRSFAVISEFVEHQIVHTRESLY
                                * *                *   ****  ******** * *** ****** **** **

lcl|Query_10001               EYGESFIHSVAVNEVQKGQGGGKRFECKECGETFSRSAALAEHRQIHAREYLAECRDQED
lcl|Query_10002               EYGESFIHSVAVNEVQKGQGRGKRFECKECGETFSRSAALAEHRQIHAREYLAECRDQED
lcl|Query_10003               EYGESFIHSVAVSEVQKSQAGGKRFECKECGETFNKSATLAEHRKIHAREYLAESNDQEY
lcl|Query_10004               EYGESFIHSVAVSEVQKSQVGGKRFECKDCGETFNKSAALAEHRKIHARGYLVECKNQEC
lcl|Query_10005               EYGESFIHSVAVSEVQKSQVGGKRFECKDCGETFNKSAALAEHRKIHARGYLVECKNQEC
lcl|Query_10006               EYGESFIHSVAVSEVQKSQIGGKRFECKDCGETFNKSAALAEHRKIHARDYLVECKDQAY
lcl|Query_10007               EYGESFIHSVAVSEVQKSQAGGKRFECKECGETFSKSTALAEHRKIHAREHLAECNDEEY
lcl|Query_10008               EYGESFIHSVAVSEVQKSQAGGKRFECKECGETFNKSAALAEHRKIHAREHLAECNDEEY
lcl|Query_10009               EYGESFIHSVAVSEVQKSQAGGKRFECKECGETFNKSAALAEHRKIHAREYFSECKDEEY
lcl|Query_10010               EYGESFIHSAAVSEAQSRPEGARRSEGAQ-------AAGLAEHRGGQAQEHLRGSGDEEQ
                              ********* ** * *       * *             *****   *

lcl|Query_10001               EETIMPSPTFSELQKMYGKDKFYECKVCKETFLHSSALIEHQKIHGRG-NSDDRDN--ER
lcl|Query_10002               EETVMPSPTFSELQKMYGKDKFYECKVCKETFLHSSALIEHQKIHGRG-NSDDRDN--ER
lcl|Query_10003               EEAFMPSPTFSELQKIYGKDKFYECKVCKETFLHSSALIEHQKTHGRGNFSDDKDN--ER
lcl|Query_10004               EEAFMPSPTFSELQKIYGKDKFYECRVCKETFLHSSALIEHQKIH----FGDDKDN--ER
lcl|Query_10005               EEAFMPSPTFSELQKIYGKDKFYECRVCKETFLHSSALIEHQKIH----FGDDKDN--ER
lcl|Query_10006               EEAFMPSPTFSELQKIYGKDKFYECRVCKETFLHSSALIEHQKIH----FGDGKDNDRER
lcl|Query_10007               EEPFMPSPTFSELQKIYGKDKFYECKVCKETFLHSSALIDHQKTHGR----DDKDN--ER
lcl|Query_10008               EEPFMPSPTFSELQKIYGKDKFYECKVCKETFLHSSALIDHQKIHGR----DDKDN--ER
lcl|Query_10009               EEPFMPSPTFSELQKIYGKDKFYECKVCKETFLHSSALIDHQKIHGR----NDKDN--DR
lcl|Query_10010               DEPFLPSPTFSELQKMYGKDKFYECKVCKETFLHSSALIEHQKIHSH----EDREK--ER
                               *   ********** ********* ************* *** *              *

lcl|Query_10001               ERERDRLRARAREQRERERERERERERL----GEPFLTCPNFNEFRKMYRKDKIYECKVCG
lcl|Query_10002               ERERDRLRARAREQRERERERERERERERHEHGEPFLTCPNFNEFRKMYRKEKIYECKVCG
lcl|Query_10003               ERQR---------------------EHER--GEAFMPSSAFNEFQKMYGKEKIYECKVCG
lcl|Query_10004               EHER---------------------ERERERGETFRPSPALNEFQKMYGKEKMYECKVCG
lcl|Query_10005               EHER---------------------ERER--GETFRPSPALNEFQKMYGKEKMYECKVCG
lcl|Query_10006               ERER---------------------ERER--GEAFMPSSTLNEFQKMYGKEKMYECKVCG
lcl|Query_10007               -------------------------------GEAFKPS--LNELQKMYGKEKMYECKVCG
lcl|Query_10008               -------------------------------GEGFKPSPPPNDLPKTYGKEKMYECKVCG
lcl|Query_10009               -------------------------------GEAFKLSPTLSELQKMYGKEKMYECKVCG
lcl|Query_10010               STGAVR----------------------R--------TPMLGELQRACGKEKRYECKVCG
                                                                               * * *******

lcl|Query_10001               ESFLHLSSLREHQKIHTRGNPFENKSRMCEETFVPSQSLRRRQKTY-REKLFDFNNARDA
lcl|Query_10002               ESFLHLSSLREHQKIHTRGNPFENKSRVCEETFVPSQSLKRRQKTY-REKLFDFNNARDA
lcl|Query_10003               ETFLHSSSLREHQKIHTRGNPFENKGKICEETFIPGQSFKRRQKTYTKEKLYDFTDGRDA
lcl|Query_10004               ETFLHSSSLKEHQKIHTRGNPFENKGKVCEETFIPGQSLKRRQKTYNKEKLCDFTDGRDA
lcl|Query_10005               ETFLHSSSLKEHQKIHTRGNPFENKGKVCEETFIPGQSLKKRQKTYNKEKLYDFTDGRDA
lcl|Query_10006               ETFLHSSSLKEHQKIHARGNPFENKGKVCEETFIPGQSLKRRQKTYNKEKLYDFTDGRDA
lcl|Query_10007               ETFHHSSSLKEHQKIHTRGNLFENKGKVCEETFIPGQSLKRHQKSYSKEKLYDFKDGGDA
lcl|Query_10008               ETFHHSSSLKEHQKIHTRGNLFESKGKVCEETFIPGQSLKRRQKTYPKEKLYDFTDGGDA
lcl|Query_10009               ETFRHSSSLKEHQKIHTRGNLFEKKGKVCEETFIPGQSLKRRQKTYSKEKFYDFKDGGDA
lcl|Query_10010               ETFHHSAALREHQKTHGRGSPSEGRARAFEETFIPGQSLKRRQKTYSKEKLYDFREGGDA
                              * * *   * **** * **   *      **** * **    ** *  **  **    **

lcl|Query_10001               LMGNSDSSEHQKNRSRRNFFEGRGFEK------PFVESQKSHTITRPPENKDDDKPFTIS
lcl|Query_10002               LMGSSDPSEHQKNRSRKNFFEGRGFEK------PFVESQKSHTITRPPENREDDKPFTIS
lcl|Query_10003               FL-SSDISEHQKIHSRKNLFESRGYEKPVIHNMPFTESQKSHTITRPPENEEEEKAFTIS
lcl|Query_10004               FMQSSELSEHQKIHSRKNLFEGRGYEKSVIHSGPFTESQKSHTITRPLESDEDEKAFTIS
lcl|Query_10005               FMQSSELSEHQKIHSRKNLFEGRGYEKSVIHSGPFTESQKSHTITRPLESDEDEKAFTIS
lcl|Query_10006               FMQSSELSEHQKIHSRKNIFEGRGYEKSVIHSMPFTESQKSHTITRPPESDEDEKPFTIS
lcl|Query_10007               FRQSSDLSEHQKIHSRKNLYEGRGYEKSVIHSVPFTESQKSHTITRPPENEEDEKAFTIS
lcl|Query_10008               FRQSSDLSEHQKIHSRKNLFEGRGYEKSVIHSVPFTESQKSHTITRPPEDDEDQKAFTVS
lcl|Query_10009               FRKSSDLNVHQKIHSRKHLYEGRGYEKSVFHGLSFTESQKSHTITRPPENEEDEKAFTIS
lcl|Query_10010               FGRSSDFMEHQKIHSRKSYFDSRGYEKPLLHSMSMPGSQKSHTITRPPEDEDEEKAFTAS
                                  *    ***  **      ** **          ********** *     * ** *

lcl|Query_10001               VNPNDKLKFPIMENGSQGKSYERSVIHSLGSAEAQKS-HGGLGFSKPRPVAESSTQSSSS
lcl|Query_10002               VNPNDKLKFSAMESSSQGKSYERSVIHSLGSPEAQKS-HGALGFSKPKSVTESSTQTSSS
lcl|Query_10003               SNPSGNQKFPLRENVYERKPYERSVIHSLASTAAQRS-YIAVGPSKLKVIAESTFQSSNV
lcl|Query_10004               SNPYENQKIPTKENVYEAKSYERSVIHSLASVEAQKS-HSVAGPSKPKVMAESTIQSFDA
lcl|Query_10005               SNPYENQKIPTKENVYEAKSYERSVIHSLASVEAQKS-HSVAGPSKPKVMAESTIQSFDA
lcl|Query_10006               SNPFENQ-IPTKENVYEGKSYERSVIHSLASVEAQKS-HSVAGPSKPKVMAESTIQSFEA
```

```
lcl|Query_10007              SNPDDNQKIPTKENACERKPYERSVIHSLAFAKAQKSCHSAVGPSKPQVIAESATQTSGV
lcl|Query_10008              SNPDDNQKVPPQENVYERKPYERSVIHSLAFAKAEKS-HSAVGPSKPKVIAESTIQSSGV
lcl|Query_10009              SNPDDNQTFPIKDNVSEGKPYERSVIHSLASAEAQKS-HSAAGPNKLKVIAESVIQSSNV
lcl|Query_10010              SSPEDGQ---------EARGYERSA-----------------------------------
                               *                 ****

lcl|Query_10001              IYYPRAHSGGNTYEGKEYKDSIIHSLPAPRPLKRHRANDHIQCDEGGESSIYIPDIINKG
lcl|Query_10002              INYRRTHSGGITYEGKEYKGSIIHSLPAPRPLKRHRVSDQIQCDEEGESSIYIPDII-KR
lcl|Query_10003              ISYQKIRTGGNTYEGKEYKRSVIHSLAAPRPLKRHIVSDLGGCDERGESSIYISDLSNKR
lcl|Query_10004              INHQRVRAGGNTSEGREYSRSVIHSLVASKPPRSHNGNELVESNEKGESSIYISDLNDKR
lcl|Query_10005              INHQRVRAGGNTSEGREYSRSVIHSLVASKPPRSHNGNELVESNEKGESSIYISDLNDKR
lcl|Query_10006              INHQRVRAGGNTSEGRGYSRSVIQRLVASKLPKSHNGNELVESNENGESSIYISDLNGKR
lcl|Query_10007              IEHQKVHAGEN-SEGKKYETSVIHTLAAFKPPKNCSGNEVVQCDEKGESSTYLSNLCDKQ
lcl|Query_10008              TEHQKAHAGENTSEGKKYERSVIHSVAAFKPPKSCNGNEVVECEEKGESSTSVSDRHDKQ
lcl|Query_10009              TEHQKVYAGENTSDRKKYERSVIHSLATFRPPKSCDGNELIECNEKAESSIYVSDLHDKQ
lcl|Query_10010              -----------------YERAILHSLAAFRPPRGLR--------EDGEPSTYLSGLRDPP
                                              *                          *  * *

lcl|Query_10001              RKIPAREDAYEG-SSSSNYHTP---NVSRAEPP-SLSGE-SHDSKQDVTFSVPSSSVREH
lcl|Query_10002              RKIPAREDAYEG-SSSSSYHTA---SVPRAEPP-SVSGE-SRESKQDVTFSVPSSSVREH
lcl|Query_10003              QKIPARENPYES-DVNNSHEDSVLQSVSYVRPQKSLAGEGSSELKQDGEFSVPSSSVRQH
lcl|Query_10004              QKIPARENPCEGGSKNRNYEDSVIQSVFRAKPQKSVPGEGSGEFKKDGEFSVPSSNVREY
lcl|Query_10005              QKIPARENPCEGGSKNRNYEDSVIQSVSRAKPQKSVPGEGSGEFKKDGEFSVPSSNVREY
lcl|Query_10006              LKIAARENPCEGGSKSHNYEDSVIQSVSHAKPQKSVSGKGSGEFKKDGEFSVPSSNVREY
lcl|Query_10007              QKTPARETPYEG-AKSNNQKDSVIQSVSRIEPQKSLPSQGSSE------SSIPSSNVREH
lcl|Query_10008              QKTPARENPNEG-GKNNNYKDSVIQSVSHMESQKSPTSQGSSELKKDGESSTPTSNVREH
lcl|Query_10009              QKTPARQNPYEG-DKNNSYKDSVIHSMSHTKPQKSLTGEESSEFKKDGESSVVNSNVREH
lcl|Query_10010              QKTPAWESPYAG-GRHSFFRSSVFYRASRPAPLDHLAGEGPSGWQRDGEASGPSSDGRQH
                              *  *                                             *   *  *

lcl|Query_10001              QKARAKKKYIEPRNNETSVIHSLPFGELLAGHRRAKFFECQECGEAFARRSELIEHQKIH
lcl|Query_10002              QKARAKKKYIEPRSNETSVIHSLPFGEFLAGHRRAKFFECQECGEAFARRSDLIEHQKIH
lcl|Query_10003              QKARAKKKYIEHRSNETSVIHSLPFGELQRIRPRERLYECQECGESFARISDLTEHQKIH
lcl|Query_10004              QKARAKKKYIEHRSNETSVIHSLPFG-EQTFRPRGMLYECQECGECFAHSSDLTEHQKIH
lcl|Query_10005              QKARAKKKYIEHRSNETSVIHSLPFG-EQTFRPRGMLYECQECGECFAHSSDLTEHQKIH
lcl|Query_10006              QKARAKKKYIEHRSNETSVIHSLPFGKQQTHRPRGMLYECQECGECFAHRSDLTEHQKIH
lcl|Query_10007              QKARAKKKNIEHRNYETSVIHSLRFGEPQTFRPREKFYECPECGESFVRISDLTEHQKIH
lcl|Query_10008              QKARSKKKNIERRNYETSVIHSLRFGDHQTFRPREKFYECPECGESFVRSYDLTEHLKIH
lcl|Query_10009              QKARAKKKNIERRNYETSVIHSLSFGENQTFRPREKFYECPVCGESFVRNSDLTEHQKIH
lcl|Query_10010              QKARAKKKNIERKNYDASMMHSLHFGESQTFRPRERFYECLECGEFFVRSSELAEHQKIH
                             **** *** **      *  *** **       *    **  *** *     * ** ***

lcl|Query_10001              DRERPSGSRHYERSVIRSLAPSDP------------------------------------
lcl|Query_10002              DRERPSGSRHYERSVIRSLAPSDP------------------------------------
lcl|Query_10003              DREKPSGSKNYEQSVIRSLAPTDP------------------------------------
lcl|Query_10004              DREKPSGSRNYEWSVIRSLAPTDP------------------------------------
lcl|Query_10005              DREKPSGSRNYEWSVIRSLAPTDP------------------------------------
lcl|Query_10006              DREKPSGSRNYEWSVIRSLAPTDP------------------------------------
lcl|Query_10007              DRKKPSGSKNYERSVIRSLASTDP------------------------------------
lcl|Query_10008              DRKKPSGSKNYERSVIRSLVSTDP------------------------------------
lcl|Query_10009              DRKKPSGSKNYERSVIRSLASTDP------------------------------------
lcl|Query_10010              NRKKLSGSKNYLRSVLRSLSSTDPQTSYQGQSVQMSYPQEAAQTSYAELAAQTSYAEEPA
                              *   ***  *  ** ***   **

lcl|Query_10001              ------------------------------------------------------------
lcl|Query_10002              ------------------------------------------------------------
lcl|Query_10003              ------------------------------------------------------------
lcl|Query_10004              ------------------------------------------------------------
lcl|Query_10005              ------------------------------------------------------------
lcl|Query_10006              ------------------------------------------------------------
lcl|Query_10007              ------------------------------------------------------------
lcl|Query_10008              ------------------------------------------------------------
lcl|Query_10009              ------------------------------------------------------------
lcl|Query_10010              QTSYAVEPAQTSYAEEPAQTSYTEAPAEASYTEEPAQTSCIEEPAQTSYTNPAAETSYAE


lcl|Query_10001              ------------------------------------------------------------
lcl|Query_10002              ------------------------------------------------------------
lcl|Query_10003              ------------------------------------------------------------
lcl|Query_10004              ------------------------------------------------------------
```

```
lcl|Query_10005          ------------------------------------------------------------
lcl|Query_10006          ------------------------------------------------------------
lcl|Query_10007          ------------------------------------------------------------
lcl|Query_10008          ------------------------------------------------------------
lcl|Query_10009          ------------------------------------------------------------
lcl|Query_10010          EPAQTSYTEAPAEASYTEEPAQTSCIEEPAQTSYTNPAAETSYTEEPAQTSYTEAPAEAS

lcl|Query_10001          ------------------------------------------------------------
lcl|Query_10002          ------------------------------------------------------------
lcl|Query_10003          ------------------------------------------------------------
lcl|Query_10004          ------------------------------------------------------------
lcl|Query_10005          ------------------------------------------------------------
lcl|Query_10006          ------------------------------------------------------------
lcl|Query_10007          ------------------------------------------------------------
lcl|Query_10008          ------------------------------------------------------------
lcl|Query_10009          ------------------------------------------------------------
lcl|Query_10010          GIEEPAQTNYTEESAEVSYTEEPSQTSCIEEPAQTSYTDPAAETSYTEEPAQTSYTQEPA

lcl|Query_10001          ------------------------------------------------------------
lcl|Query_10002          ------------------------------------------------------------
lcl|Query_10003          ------------------------------------------------------------
lcl|Query_10004          ------------------------------------------------------------
lcl|Query_10005          ------------------------------------------------------------
lcl|Query_10006          ------------------------------------------------------------
lcl|Query_10007          ------------------------------------------------------------
lcl|Query_10008          ------------------------------------------------------------
lcl|Query_10009          ------------------------------------------------------------
lcl|Query_10010          QTSCTEEPAQTSCTEEPAQTSYTQEPAQTSYTKEPAEASYTEEPAQTSCIEEPAQTNYTK

lcl|Query_10001          ------------------------------------------------------------
lcl|Query_10002          ------------------------------------------------------------
lcl|Query_10003          ------------------------------------------------------------
lcl|Query_10004          ------------------------------------------------------------
lcl|Query_10005          ------------------------------------------------------------
lcl|Query_10006          ------------------------------------------------------------
lcl|Query_10007          ------------------------------------------------------------
lcl|Query_10008          ------------------------------------------------------------
lcl|Query_10009          ------------------------------------------------------------
lcl|Query_10010          ESAKASYTEEPAQTSYTDPAAETSYTEEPAQTNYTVESAEASYTEEPSQTSCIEEPAQTS

lcl|Query_10001          ------------------------------------------------------------
lcl|Query_10002          ------------------------------------------------------------
lcl|Query_10003          ------------------------------------------------------------
lcl|Query_10004          ------------------------------------------------------------
lcl|Query_10005          ------------------------------------------------------------
lcl|Query_10006          ------------------------------------------------------------
lcl|Query_10007          ------------------------------------------------------------
lcl|Query_10008          ------------------------------------------------------------
lcl|Query_10009          ------------------------------------------------------------
lcl|Query_10010          YTDSAADTSCTEEPAQTSCTEEPAQTSYTQEPAQTSCTEEPAQTSCTEEPAQTSYTQEPA

lcl|Query_10001          ------------------------------------------------------------
lcl|Query_10002          ------------------------------------------------------------
lcl|Query_10003          ------------------------------------------------------------
lcl|Query_10004          ------------------------------------------------------------
lcl|Query_10005          ------------------------------------------------------------
lcl|Query_10006          ------------------------------------------------------------
lcl|Query_10007          ------------------------------------------------------------
lcl|Query_10008          ------------------------------------------------------------
lcl|Query_10009          ------------------------------------------------------------
lcl|Query_10010          QTSCTEEPAQTSYTQEPAQTSCTEEPAQTSYTEEPAQTSYTEEPAQTSYTQEPAQTSCTE

lcl|Query_10001          ------------------------------------------------------------
lcl|Query_10002          ------------------------------------------------------------
```

```
lcl|Query_10003          ------------------------------------------------------------
lcl|Query_10004          ------------------------------------------------------------
lcl|Query_10005          ------------------------------------------------------------
lcl|Query_10006          ------------------------------------------------------------
lcl|Query_10007          ------------------------------------------------------------
lcl|Query_10008          ------------------------------------------------------------
lcl|Query_10009          ------------------------------------------------------------
lcl|Query_10010          EPAQTSYTEEPAQTSYTEEPAQTSYTQEPAQTSYTEEPAQTSYTEEPAQTSYAQEPAQTS

lcl|Query_10001          ------------------------------------------------------------
lcl|Query_10002          ------------------------------------------------------------
lcl|Query_10003          ------------------------------------------------------------
lcl|Query_10004          ------------------------------------------------------------
lcl|Query_10005          ------------------------------------------------------------
lcl|Query_10006          ------------------------------------------------------------
lcl|Query_10007          ------------------------------------------------------------
lcl|Query_10008          ------------------------------------------------------------
lcl|Query_10009          ------------------------------------------------------------
lcl|Query_10010          YAEEPAQTSYAEEPAQTSYAEEPAQTSYTQEPAQTNYTEEPAEASYTEEPAQTSYAEEPA

lcl|Query_10001          ------------------------------------------------------------
lcl|Query_10002          ------------------------------------------------------------
lcl|Query_10003          ------------------------------------------------------------
lcl|Query_10004          ------------------------------------------------------------
lcl|Query_10005          ------------------------------------------------------------
lcl|Query_10006          ------------------------------------------------------------
lcl|Query_10007          ------------------------------------------------------------
lcl|Query_10008          ------------------------------------------------------------
lcl|Query_10009          ------------------------------------------------------------
lcl|Query_10010          QTSYPEEPAQTSYAEEPAQTSYAEEPAQTSYPEEPAQTSYTEEPAQTSYAKEPAQTSYPE

lcl|Query_10001          ------------------------------------------------------------
lcl|Query_10002          ------------------------------------------------------------
lcl|Query_10003          ------------------------------------------------------------
lcl|Query_10004          ------------------------------------------------------------
lcl|Query_10005          ------------------------------------------------------------
lcl|Query_10006          ------------------------------------------------------------
lcl|Query_10007          ------------------------------------------------------------
lcl|Query_10008          ------------------------------------------------------------
lcl|Query_10009          ------------------------------------------------------------
lcl|Query_10010          EPAQTSYAEEPAQTSYAEEPAQTSYAEEPAQTSYSEEPAQTRYTGNELRSDMRKNQLRPD

lcl|Query_10001          ------------------------------------------------------------
lcl|Query_10002          ------------------------------------------------------------
lcl|Query_10003          ------------------------------------------------------------
lcl|Query_10004          ------------------------------------------------------------
lcl|Query_10005          ------------------------------------------------------------
lcl|Query_10006          ------------------------------------------------------------
lcl|Query_10007          ------------------------------------------------------------
lcl|Query_10008          ------------------------------------------------------------
lcl|Query_10009          ------------------------------------------------------------
lcl|Query_10010          MPRNQLRPVMPRNQLRPDMPRNQPRPVILRNQLRPDMPRNQPRPVILRNQLRPDMLGNQL

lcl|Query_10001          ------------------------------------------------------------
lcl|Query_10002          ------------------------------------------------------------
lcl|Query_10003          ------------------------------------------------------------
lcl|Query_10004          ------------------------------------------------------------
lcl|Query_10005          ------------------------------------------------------------
lcl|Query_10006          ------------------------------------------------------------
lcl|Query_10007          ------------------------------------------------------------
lcl|Query_10008          ------------------------------------------------------------
lcl|Query_10009          ------------------------------------------------------------
lcl|Query_10010          RPDMPGNQLRPDMLREPPAETSYAELVAQISYAELVTPTSYAELAAETGYFEPPAQTSYT
```

```
lcl|Query_10001          ------------------------------------------------------------
lcl|Query_10002          ------------------------------------------------------------
lcl|Query_10003          ------------------------------------------------------------
lcl|Query_10004          ------------------------------------------------------------
lcl|Query_10005          ------------------------------------------------------------
lcl|Query_10006          ------------------------------------------------------------
lcl|Query_10007          ------------------------------------------------------------
lcl|Query_10008          ------------------------------------------------------------
lcl|Query_10009          ------------------------------------------------------------
lcl|Query_10010          EPAETNYADPAAQVSFDEPPAEASYADLAAEISYAELAAETSYADLAAQISYDEPPAETS


lcl|Query_10001          ---------------------------------QTSYAQER-------------------
lcl|Query_10002          ---------------------------------QTSYAQER-------------------
lcl|Query_10003          ---------------------------------QTSYAQEQ-------------------
lcl|Query_10004          ---------------------------------QTSYAQEQ-------------------
lcl|Query_10005          ---------------------------------QTSYAQEQ-------------------
lcl|Query_10006          ---------------------------------QTSYAQEQ-------------------
lcl|Query_10007          ---------------------------------QTSYAEQQAQTSYAEHSNSRQMRYPEQ
lcl|Query_10008          ---------------------------------QTSYAEQQPQTSYAGHSS--QMRYSDQ
lcl|Query_10009          ---------------------------------QTSYAEQPAQTSYAEQPA--QMNPAEQ
lcl|Query_10010          YAELAAQISYSEPADQTSYAELAAQTSYSEPLAQTSYAELT-------------------
                                                          *****

lcl|Query_10001          -----FIQEQVRKFRA-F-----------GQRST--------------------------
lcl|Query_10002          -----FIQEQVRKFRA-F-----------GQRST--------------------------
lcl|Query_10003          -----YAEEQARNEFK-F-----------RQRFT--------------------------
lcl|Query_10004          -----YAKEQARNKCKDF-----------RQFFA--------------------------
lcl|Query_10005          -----YAKEQAWNKCKEF-----------RQFFA--------------------------
lcl|Query_10006          -----YAKEQARNKYKEF-----------RQSFA--------------------------
lcl|Query_10007          PAQTSYTKQPAQASYTKQPAQASYAKQPAQASYSAHPVHLSYSEHPVRMSYTEQPGRVSY
lcl|Query_10008          AAQTSYAKHPVQTSYSGM-----------HMSYAVQPGH---------MSYTQQAAQTSY
lcl|Query_10009          PSETSYAEQQVRKKCKEC-----------GQSFA--------------------------
lcl|Query_10010          -SETSYCEQPVLNECKEC-----------GECFA--------------------------


lcl|Query_10001          -----------------------------------TSNNLSV-QKIYAQETFNAEEP---
lcl|Query_10002          -----------------------------------TSNNLSV-QKIYAQEKFNAEEP---
lcl|Query_10003          -----------------------------------NSNNLSTHQKIYAQEKSHGEEP---
lcl|Query_10004          -----------------------------------TSEDLNTNQKIYDQEKSHGEES---
lcl|Query_10005          -----------------------------------TSEDLNTNQKIYDQEKSHGEES---
lcl|Query_10006          -----------------------------------TNKDLNTHQKNYDQEKSHGKKS---
lcl|Query_10007          AQQPAQMNYTEEQAQTSFAEQQVHNKCKECGECFATLEELGAHQKIYAREEFHGRKLFGN
lcl|Query_10008          MVQPTQISYDEEQAQTSYAEQQVRNRCRECGECFATIGDLGAHQKIYAREEFHGRKLFGD
lcl|Query_10009          -----------------------------------TTEELRAHQKIYAREEFHGGNLFGG
lcl|Query_10010          -----------------------------------TVEDLGRHQKIYAREKFHDGKLFGE
                                                                *   ** *  *

lcl|Query_10001          ---HDKETHGQKIHD--------------------KEPYGKE-PSG----KEPHGDEPQD
lcl|Query_10002          ---HDKETHGQKIHD--------------------KEPYGKE-PSG----QDPHGDEPQD
lcl|Query_10003          ---RGEEPRGKEPHG--------------------EETHGEQTPEEMNPEEETHGQETPE
lcl|Query_10004          ---QGENTDGEETHS--------------------EETHGQETIED--PVIQGSDMEDPQ
lcl|Query_10005          ---QGENTDGEETHS--------------------EETHGQETIED--PVIQGSDMEDPQ
lcl|Query_10006          ---QGKKTHGEETHGKKSHGEKSNGEKTQDEEMHGEETHGQEIIED--PVIQGSDMEEPQ
lcl|Query_10007          SVIQGVGLDGPRLVESR---------------------PEEPRQEE--PDEQD---EPDE
lcl|Query_10008          TVIQGIGLEGPR--------------------------PEEPRQNE--PDEQ------DE
lcl|Query_10009          SVIQGVGLDGPQ--------------------------QGEPQQDK--PDEQD---ELDE
lcl|Query_10010          PVMQDLGLDG--------------------------------SPEE----------ELEE
                                  *

lcl|Query_10001          KEPLDQEMRSEEPHDDKPHGQEPHDDKPHGQEPHDDKPH---------------GQEPHG
lcl|Query_10002          KEPQDKEPQDKEPQDKEPQDKEPQDKEPQDKEPQDKQPQDKEPQDKEPLDQEMRSEEPHG
lcl|Query_10003          EETRGQETPGKETRGQETPGEETRGQETPGEETRGQETAGQETRGQETPDEETRGQE-TP
lcl|Query_10004          KD-------------------------------------DPDDKIYECEDCGLGFVD-LT
lcl|Query_10005          KD-------------------------------------DPDDKIYECEDCGLGFVD-LT
lcl|Query_10006          KD-------------------------------------DPDDTIYECEDCGLGFVD-LT
lcl|Query_10007          QD-------------------------------------EPEDTIYGCKDCGLGFAH-RA
lcl|Query_10008          QD-------------------------------------EPEDAIYGCKDCGLGFAD-RA
lcl|Query_10009          QD-------------------------------------ESEDTIYGCKDCGLGFAD-RA
lcl|Query_10010          QE----------------------------------EPEEPEDSIYGCKDCGLGFAD-RA
```

```
lcl|Query_10001               DEPHGQEPHGDEPHDKEPIDQEMRSEEPHSEESHGDEPHGEESHGQEKVEDATIQASVSE
lcl|Query_10002               DQPHGQEPHGDEPHDKEPVDQEMPSEEPQ----------GEESHGQEKAEDITIETSVSE
lcl|Query_10003               DETHGEETHGEKTPGEET---------------RGQETPGEETHGEETVEDAVIQGSDLD
lcl|Query_10004               DLTDHQKVHSRKCLVDSR----------------------EYTH-------SVIHTHSIS
lcl|Query_10005               DLTDHQKVHSRKCLVDSR----------------------EYTH-------SVIHTHSIS
lcl|Query_10006               DLTDHQKVHRRKCLVDSR----------------------EYTH-------SVSHTHSIS
lcl|Query_10007               DLKDHQKVHGREYLIDSR----------------------EYTH-------SVIHTHSVS
lcl|Query_10008               DLKDHQKVHGREYLIDSR----------------------EYTH-------SVIHTHSVS
lcl|Query_10009               DLKDHQKVHGREYLIDSC----------------------EYTH-------SVIHTHSVS
lcl|Query_10010               DLRDHQKVHGREYLVDSR----------------------EYTH-------PAVHMPPVS
                              *       *                               *  *

lcl|Query_10001               EHQKDDAGDAIYECQDCGLGFTDLNDLTSHQDTHSR--KALVDSREYAHSEVHAHSVSEF
lcl|Query_10002               EPQKDDAGDAIYECQDCGLGFADLNDLTSHQDVHSR--KSLVDSREYTHSEVHVHSVSEF
lcl|Query_10003               EPQKDDPDNTIYECQDCGLGFVDLTDLTDHQNVHSTNTKCLVDSREYTHSVIHTHSISEY
lcl|Query_10004               EYQRDYTGEQLYECPKCGESFIHSSFLFEHQRIHEQ------------------------
lcl|Query_10005               EYQRDYTGEQLYECPKCGESFIHSSFLFEHQRIHEQ------------------------
lcl|Query_10006               EYQRNYTGEQLYECPKCGESFIHSSFLFEHQRIHEQ------------------------
lcl|Query_10007               EYQKDYIGEQLYECPACGESFVHSSFLFEHQKIHEQ------------------------
lcl|Query_10008               EYQKDYIGEQLYECPACGESFVHSSFLFEHQKIHEQ------------------------
lcl|Query_10009               EYQKDSIGDQLFECPACGESFVHSSFLFEHQKIHEQ------------------------
lcl|Query_10010               EYQKDCLGEQLYECPACGESFVHSSFLFEHQKVHEQ------------------------
                              * *         **  **  *     *  **  *

lcl|Query_10001               EKKCSGEKLYECPKCGESFIHSSLLFEHQRVHEQDQLYSVKACDDAFIALLPVRPRRNCT
lcl|Query_10002               EKKYSGEKLYECPKCGESFIHSSLLFEHQRVHEQDQTYSVKACDDGFIALLPARPRRNCT
lcl|Query_10003               QREYTGEQLYECPKCGESFIHSSFLFEHQRVHEQDQLYALKGCDDGFISLLPVKPRRNRA
lcl|Query_10004               ------DQLY----------------------------SMKGCDDGFIALLPMKPRRNRA
lcl|Query_10005               ------DQLY----------------------------SMKGCDDGFIALLPMKPRRNRA
lcl|Query_10006               ------DQLY----------------------------SLKGCDDGFIALLPVKPRRNRA
lcl|Query_10007               ------DQFY----------------------------GHRRYDEPFVQPLVINPRRPRA
lcl|Query_10008               ------DQFY----------------------------GQRRYDEPFVQPLVINPRRPRA
lcl|Query_10009               ------DQFF----------------------------GHRRYDEPFMQPLIINPHRPRA
lcl|Query_10010               ------DQFYG----------------HRRY-------------EPFMQPLIVSPRRPQA
                                                                            *   *   * *

lcl|Query_10001               VERNPAVSGSAIRCRQCGQGFIHSSALNEHMRQHRDNEIMEQSELSDEIFIQGLALTEYQ
lcl|Query_10002               VERNPAVSGSAIRCRQCGQGFIHSSALNEHMRQHRDNEILEQNELADEIFIQGLALTEYQ
lcl|Query_10003               AERNPALAGSAIRCLQCGQGFIHSSALNEHMRLHREDELLEQSEMAEEAIMSGLALTEFQ
lcl|Query_10004               AERNPALAGSAIRCLLCGQGFIHSSALNEHMRLHREDDLLEQSQMAEEAIIPGLALTEFQ
lcl|Query_10005               AERNPALAGSAIRCLLCGQGFIHSSALNEHMRLHREDDLLEQSQMAEEAIIPGLALTEFQ
lcl|Query_10006               AERNPALAGSAIRCLLCGQGFIHSSALNEHMRLHREDDLLEQSQMAEEAIIPGLALAEFQ
lcl|Query_10007               PQKNPP-AGTSLQCHVCGQDFIHGSVLSEHMRIHTGEDLPEQGQRSEDAVSPGLALTEFQ
lcl|Query_10008               PQKNPT-AGTSLQCHVCGQDFIHGSVLGEHMRIHTREDLPEQGQRSEDAVSPGLALTEFQ
lcl|Query_10009               SQKNPP-TGTSLQCCVCGRDFIHGSVLNEHMRIHTGEDLPEQGQRSEEAVSPGLALTEFQ
lcl|Query_10010               PQKSAP-AGVGPQCQVCGQDFIHASVLSEHARGHAGEGLPDQGQGGAGAAGPGPAPTEPQ
                                      *    *  **  *** * * ** * *       *          * *  * *

lcl|Query_10001               GSETEEKLFECTICGECFFTAKQLGDHHTKVH-KDEPYEYGPSYTHASFLTEPLRKHIPL
lcl|Query_10002               GSETEEKLFECTICGECFFTAKQLGDHHTKVH-KDEPYEYGPSYTHASFLTEPLRKHIPL
lcl|Query_10003               GSESEEKLFECTICGECFFTASELGDHHIKVH-KDEPYDYGPSFTHTSFLTEPLKGAIPF
lcl|Query_10004               RSQTEERLFECAVCGESFVNPAELADH-VTVH-KNEPYEYGSSYTHTSFLTEPLKGAIPF
lcl|Query_10005               RSQTEERLFECAVCGESFINPAELADH-VTVH-KNEPYEYGSSYTHTSFLTEPLKGAIPF
lcl|Query_10006               RSQTEERLFECAVCGESFVNAAELADH-VTVH-KNEPYEYGSSYTHTSFLTEPLKGAMPF
lcl|Query_10007               RSQTEEKHYECKTCGESFLNQADLREH-MRIHEKDEPYDYGASFVHTSFLTEPPKRDSPF
lcl|Query_10008               RSQTEEKHYECKTCGETFLNQSDLREH-MRIHEKDEPYDYGASFVHTSFLTEPPKRDSPF
lcl|Query_10009               RSQTEEKHYECKTCGESFLNQSDLRDH-MRIHEKDEPYDYGVTFLHTSFIAEPPKRDSPF
lcl|Query_10010               QDPGEEQRYECETCGESFPSQADLQEH-MRVHEKGEPYDYGAAFVHTSFLTEPPKRDWPF
                                  **   **  *** *     *  *    * * *** **    * **  **     *

lcl|Query_10001               YECKDCGQSFLDDTVIAERMVFHPEREGGSE---IVAATAQEVEANVLIPQEVLRIQGSN
lcl|Query_10002               YECKDCGQSFLDDTVITERMVFHPEREGGSE---IVAATAQEVEANVLIPQEVLRIQGSN
lcl|Query_10003               YECKDCGKSFVHSTVLTKHKELHPEEDDDDDDD---DSAQAVEANVIVPQEVLRIQGSN
lcl|Query_10004               YECKDCGKSFIHSTVLTKHKELHLEEEEEDEAAAAAAAAAQEVEANVHVPQVVLRIQGLN
lcl|Query_10005               YECKDCGKSFIHSTVLTKHKELHLEEEEEDEAAAAAAAAAQEVEANVHVPQVVLRIQGSN
lcl|Query_10006               YECKDCGKSFIHSTVLTKHKELHLEEEDDDDDDEAAGAAAQEVEANVHVPQEVLRIQGSN
lcl|Query_10007               YECKDCGKSFIHNTVLNKHQKLHLEEEEE--------EGAQEVEANVLVPREVLRIQGSN
lcl|Query_10008               YECKDCGKSFIHNTVLTKHQKLHLEEEEE--------EGAQEVEANVLVPREVLRIQGSN
```

```
lcl|Query_10009          YECKDCGKSFIHSTVLTKHQKLHLEEEEA-------AAAAQEVEANVLVPREVLRIQGSN
lcl|Query_10010          YECKDCGKSFIHSTILTKHQKLHLQEEGAAA---AAAATAQEAEANVLVPREVLRIQGSN
                         ******* **   *        *                **  ****  *  ****** *

lcl|Query_10001          AEAAEPEVEAAEPEVEAAEPEVEAAEPNGEAEGPDGEAAEPDGEAEQPN---GEAEQPNG
lcl|Query_10002          AEAAEPEVEAAEPEVEAAEPEVEAAEPNGEAEGPDGEAAEPDGEAEQPN---GEAEQPNG
lcl|Query_10003          VEAAEPEVEAAEPEVEAAEPEVEAAEPNGEAEGPDGEAAEPNGEAEQPNGEAGEAEQPEG
lcl|Query_10004          VEAAEPEVEAAEPEVEAAEPEVEAAEPNGEAEGPDGEAAEPIGEAGQPN---GEAEQPNG
lcl|Query_10005          VEAAEPEVEAAEPEVEAAEPEVEAAEPNGEAEGPDGEAAEPIGEAGQPN---GEAEQPNG
lcl|Query_10006          VEAAEPEVEAAEPEVEAAEPEVEAAEPNGEAEGPDGEAAEPNGEAEQPN---GEAEQPNG
lcl|Query_10007          VEAAEPEVEAAEPEVEAAEPNVEAAEPNGEAEGPDGEAAEPNGEAEQPN---GEAEQPNG
lcl|Query_10008          VEAAEPEVEAAEPEVEAAEPNVEAAEPNGEAEGPDGEAAEPDGEAEQPN---GEAEQPNG
lcl|Query_10009          VEAAEPEVEAAEPEVEAAEPNVEAAEPNGEAEGPDGEAAEPNGEAEQPN---GEAEQPNV
lcl|Query_10010          VEAAEPEVEAAEPEVEAAEPEVEAAEPLGEAEGPEWEAAEPSGEAEQPH---AEAEQPDM
                          ******************* ****** ******  ***** *** **     *****

lcl|Query_10001          DADEPDGAGIEDPEERADEPEEDVEEPEG-------DADEPDGADIEDPEEEGEDQEIEV
lcl|Query_10002          DADEPDGAGIEDPEERADEPEEDVEEPEG-------DADEPDGADIEDPEEEGEDQEIEV
lcl|Query_10003          DADEPDGVGIEDPEER-------VEEPEG-------DADEPDGAGVEDPEEEGEDQEIEI
lcl|Query_10004          DADEPDGAGIEDPEER-------AEEPEGKAEEPEGDADEPDGVGIEDPEE-GEDQEIQV
lcl|Query_10005          DADEPDGAGIEDPEER-------AEEPEGKAEEPEGDADEPDGVGIEDPEE-GEDQEIQV
lcl|Query_10006          DADEPDGAGIEDPEER-------AEEPEGKAEEPEGDADEPDGAGIEDPEE-GEDQEIQV
lcl|Query_10007          DADEPDGAGIEDPEERAEEPEGKAEEPEG-------DADEPDGAGIEDPEEEGEDQEIQV
lcl|Query_10008          DADEPDGAGIEDPEER-------AEEPEG-------DADEPDGAGIEDPEEEGEDQEIQV
lcl|Query_10009          DADEPDGAGIEDPEER-------AEEPEG-------DADEPDGAGIEDPEEEGEDQEIQV
lcl|Query_10010          DADEPDGAGIEDPEER-------AEEPEG-------DDDEPDGAGIEDPEEEGEEQEIQV
                         ******* ********        *****       * *****   ***** ** ***

lcl|Query_10001          EEPYYNCHECAETFASSSAFGEHLKSHASVIIFEPANAPGECSGYIERASTSA-------
lcl|Query_10002          EEPYYNCHECAETFASSAAFGEHLKSHASVIIFEPANALGECSGYIERASTSA-------
lcl|Query_10003          EEPYYNCQECTETFTSSAAFGEHLKTHASVIVFEPADAFGECSGYIERASTSTSTSTSTS
lcl|Query_10004          EEPYYDCHECTETFTSSTAFSEHLKTHASMIIFEPANAFGECSGYIERASTST-------
lcl|Query_10005          EEPYYDCHECTETFTSSTAFGEHLKTHASMIIFEPANAFGECSGYIERASTST-------
lcl|Query_10006          EEPYYDCHECTETFTSSAAFGEHLKTHASMIIFEPANAFGECSGHIERASTST-------
lcl|Query_10007          EEPYYDCRECGETFTSNSAYGEHLKTHARVIIFEPGNVYGESSHYTEHASTST-------
lcl|Query_10008          EEPYYDCRECGETFASNSAYGEHLKTHARVIIFEPGNVYGESSRYTEHASTST-------
lcl|Query_10009          EEPYYDCGECGETFASTSAYGEHLKTHARVIIFEPGNVYGESSHYTEHASTST-------
lcl|Query_10010          EEPYYDCGECGETFPSGAAYAEHLTAHASLVILEPAGLYGEGAGGPE-------------
                         ***** * ** *** *  *  ***  **     **    **     *

lcl|Query_10001          -----GGAEQADDKYFKCDVCGQLFNDRLSLARHQNSHTGZ
lcl|Query_10002          -----GGAEQADDKYFKCDVCGQLFNDRLSLARHQNSHTG-
lcl|Query_10003          TSTSAGGADQADEKYFKCDVCGQLFNDRLSLARHQNTHTG-
lcl|Query_10004          -----GGANQADEKYFKCDVCGQLFNDRLSLARHQNTHTG-
lcl|Query_10005          -----GGANQADEKYFKCDVCGQLFNDRLSLARHQNTHTG-
lcl|Query_10006          -----GGADQADEKYFKCDVCGQLFSDRLSLARHQNTHTG-
lcl|Query_10007          -----SDNDRADDKYFKCDVCGQLFSDRLSLARHQNTHTG-
lcl|Query_10008          -----SDNDRADDKYFKCDVCGQLFSDRLSLARHQNTHTG-
lcl|Query_10009          -----SDNDRADDKYFKCDVCGQLFNDRLSLARHQNTHTG-
lcl|Query_10010          -------GGRPDDELFKCDVCGQLFSDRLSLARHQNTHTG-
                                    *   ********** ********** ***
```
