# Supplementary material for: PEG3 Interacts with KAP1 through KRAB-A
Source: PLoS One. 2016 Nov 29;11(11):e0167541. doi: 10.1371/journal.pone.0167541 (PMC5127583; doi:10.1371/journal.pone.0167541)
Supplement: S3 File — (PDF) [file pone.0167541.s003.pdf]

# Supplemental data 3

**Protein Quant tc-s104g**

RA = relative abundance

BH = Benjaminni-Hochberg

| | | | | Normalized %RA | | | | | | W | | K | | | T-test | |
|---|---|---|---|---|---|---|---|---|---|---|---|---|---|---|---|---|
| **Protein Id** | **Gene Symbol** | **Description** | **tified spectral** | **W1** | **W2** | **W3** | **K1** | **K2** | **K3** | **mean** | **stddev** | **mean** | **stddev** | **Ratio(K/W)** | **p value** | **adjusted p value** |
| sp\|Q8VDD5\|M | Myh9 | MYH9_MOUSE | 149 | 20.4775 | 8.18616 | 24.9216 | 13.5374 | 22.8612 | 10.0162 | 17.8618 | 8.6689 | 15.4716 | 6.6373 | 0.8662 | 7.24E-01 | 7.56E-01 |
| sp\|Q61879\|M | Myh10 | MYH10_MOUS | 128 | 12.0281 | 7.12137 | 15.389 | 19.2525 | 32.43 | 13.779 | 11.5128 | 4.1578 | 21.8205 | 9.5870 | 1.8953 | 1.63E-01 | 2.07E-01 |
| sp\|Q9QXS1\|PL | Plec | PLEC_MOUSE | 88 | 7.21056 | 5.5706 | 7.22533 | 24.0897 | 37.4553 | 18.4485 | 6.6688 | 0.9511 | 26.6645 | 9.7615 | 3.9984 | 2.42E-02 | 4.04E-02 |
| sp\|Q60847\|CC | Col12a1 | COCA1_MOUS | 85 | 1.30043 | 0.527731 | 0.395907 | 31.2333 | 51.2418 | 15.3008 | 0.7414 | 0.4886 | 32.5920 | 18.0090 | 43.9626 | 3.76E-02 | 5.76E-02 |
| sp\|Q9JHU4\|DY | Dync1h1 | DYHC1_MOUS | 69 | 21.4033 | 25.9809 | 18.1946 | 13.408 | 11.2104 | 9.80287 | 21.8596 | 3.9132 | 11.4738 | 1.8169 | 0.5249 | 1.40E-02 | 2.62E-02 |
| sp\|Q8BTM8\|F | Flna | FLNA_MOUSE | 69 | 10.2686 | 12.0175 | 9.09222 | 25.4954 | 27.442 | 15.6843 | 10.4594 | 1.4719 | 22.8739 | 6.3020 | 2.1869 | 2.93E-02 | 4.71E-02 |
| tr\|E9Q616\|E9 | Ahnak | E9Q616_MOU | 67 | 6.51335 | 6.44125 | 4.4865 | 36.9867 | 25.566 | 20.0063 | 5.8137 | 1.1500 | 27.5197 | 8.6571 | 4.7336 | 1.26E-02 | 2.40E-02 |
| sp\|Q80X90\|FL | Flnb | FLNB_MOUSE | 53 | 10.766 | 11.0453 | 10.4352 | 25.0006 | 26.4748 | 16.2782 | 10.7488 | 0.3054 | 22.5845 | 5.5110 | 2.1011 | 2.06E-02 | 3.55E-02 |
| sp\|P07901\|HS | Hsp90aa1 | HS90A_MOUS | 50 | 26.8888 | 26.5723 | 30.4978 | 6.88255 | 5.04736 | 4.11124 | 27.9863 | 2.1808 | 5.3471 | 1.4098 | 0.1911 | 1.12E-04 | 1.65E-03 |
| sp\|P14873\|MA | Map1b | MAP1B_MOUS | 50 | 30.2468 | 32.2255 | 22.5025 | 4.93687 | 5.13438 | 4.95399 | 28.3249 | 5.1385 | 5.0084 | 0.1094 | 0.1768 | 1.42E-03 | 5.48E-03 |
| sp\|Q6ZQ93\|UI | Usp34 | UBP34_MOUS | 50 | 29.4671 | 39.334 | 21.4563 | 2.81611 | 3.07653 | 3.84997 | 30.0858 | 8.9549 | 3.2475 | 0.5377 | 0.1079 | 6.60E-03 | 1.50E-02 |
| sp\|P20029\|GR | Hspa5 | GRP78_MOUS | 47 | 20.4612 | 20.9796 | 17.3223 | 17.3111 | 12.9904 | 10.9353 | 19.5877 | 1.9789 | 13.7456 | 3.2543 | 0.7017 | 5.66E-02 | 8.18E-02 |
| sp\|P20152\|VII | Vim | VIME_MOUSE | 41 | 10.1784 | 6.79169 | 8.05252 | 23.8134 | 33.8513 | 17.3127 | 8.3409 | 1.7117 | 24.9925 | 8.3321 | 2.9964 | 2.75E-02 | 4.46E-02 |
| tr\|Q3V117\|Q3 | Acly | Q3V117_MOU | 39 | 28.7588 | 29.242 | 30.5295 | 4.32965 | 3.42846 | 3.71162 | 29.5101 | 0.9153 | 3.8232 | 0.4608 | 0.1296 | 1.68E-06 | 5.68E-04 |
| sp\|P08113\|EN | Hsp90b1 | ENPL_MOUSE | 39 | 23.0732 | 21.7568 | 25.6045 | 12.8248 | 9.04683 | 7.69389 | 23.4782 | 1.9556 | 9.8552 | 2.6593 | 0.4198 | 2.03E-03 | 6.63E-03 |
| sp\|Q3URU2\|P | Peg3 | PEG3_MOUSE | 39 | 22.8982 | 49.548 | 18.1531 | 2.88961 | 2.61243 | 3.89874 | 30.1998 | 16.9232 | 3.1336 | 0.6770 | 0.1038 | 5.04E-02 | 7.41E-02 |
| sp\|P19096\|FA | Fasn | FAS_MOUSE F | 38 | 26.2429 | 25.5558 | 26.9213 | 8.57827 | 6.65389 | 6.04783 | 26.2400 | 0.6828 | 7.0933 | 1.3212 | 0.2703 | 2.39E-05 | 1.11E-03 |
| sp\|P38647\|GR | Hspa9 | GRP75_MOUS | 38 | 22.9219 | 23.2644 | 19.2155 | 13.6688 | 10.5893 | 10.3401 | 21.8006 | 2.2453 | 11.5327 | 1.8541 | 0.5290 | 3.64E-03 | 9.78E-03 |
| tr\|E9QA15\|E9 | Cald1 | E9QA15_MOU | 36 | 6.1384 | 2.24483 | 4.48834 | 28.8798 | 41.0257 | 17.2229 | 4.2905 | 1.9543 | 29.0428 | 11.9022 | 6.7691 | 2.37E-02 | 3.97E-02 |
| sp\|P48678\|LM | Lmna | LMNA_MOUSE | 36 | 11.3179 | 10.622 | 12.7694 | 19.6422 | 22.8797 | 22.7688 | 11.5698 | 1.0956 | 21.7636 | 1.8380 | 1.8811 | 1.18E-03 | 5.07E-03 |
| sp\|P58252\|EF | Eef2 | EF2_MOUSE E | 35 | 23.6475 | 21.9863 | 28.1718 | 10.3534 | 8.0648 | 7.77612 | 24.6019 | 3.2013 | 8.7314 | 1.4121 | 0.3549 | 1.42E-03 | 5.48E-03 |
| sp\|P63038\|CH | Hspd1 | CH60_MOUSE | 35 | 26.843 | 25.2458 | 26.9814 | 9.14204 | 6.33502 | 5.45271 | 26.3567 | 0.9646 | 6.9766 | 1.9265 | 0.2647 | 9.91E-05 | 1.63E-03 |
| sp\|P60710\|AC | Actb | ACTB_MOUSE | 34 | 11.8375 | 6.94084 | 14.9702 | 20.575 | 30.0173 | 15.659 | 11.2495 | 4.0468 | 22.0838 | 7.2971 | 1.9631 | 8.77E-02 | 1.21E-01 |
| sp\|P09405\|NU | Ncl | NUCL_MOUSE | 34 | 35.0372 | 29.4454 | 21.2761 | 5.3558 | 4.55784 | 4.32763 | 28.5862 | 6.9207 | 4.7471 | 0.5396 | 0.1661 | 4.01E-03 | 1.04E-02 |
| sp\|P63017\|HS | Hspa8 | HSP7C_MOUS | 33 | 24.8556 | 26.9021 | 27.4847 | 7.9767 | 6.66476 | 6.11611 | 26.4141 | 1.3808 | 6.9192 | 0.9560 | 0.2620 | 3.61E-05 | 1.17E-03 |
| sp\|P27773\|PD | Pdia3 | PDIA3_MOUSE | 33 | 23.2075 | 17.5945 | 22.1556 | 16.6431 | 11.1752 | 9.2241 | 20.9859 | 2.9837 | 12.3475 | 3.8459 | 0.5884 | 3.72E-02 | 5.70E-02 |
| sp\|P26039\|TLI | Tln1 | TLN1_MOUSE | 33 | 15.0517 | 16.3567 | 17.7096 | 20.785 | 16.9515 | 13.1455 | 16.3727 | 1.3290 | 16.9607 | 3.8198 | 1.0359 | 8.14E-01 | 8.40E-01 |
| sp\|Q8CGC7\|SY | Eprs | SYEP_MOUSE | 32 | 23.4363 | 30.6237 | 22.8818 | 9.32911 | 7.83629 | 5.89279 | 25.6473 | 4.3186 | 7.6861 | 1.7231 | 0.2997 | 2.60E-03 | 7.74E-03 |
| sp\|P16546\|SP | Sptan1 | SPTN1_MOUSI | 31 | 14.9268 | 16.7339 | 13.3756 | 20.98 | 20.4597 | 13.5241 | 15.0121 | 1.6808 | 18.3213 | 4.1626 | 1.2204 | 2.71E-01 | 3.27E-01 |
| sp\|Q01853\|TE | Vcp | TERA_MOUSE | 31 | 19.9514 | 22.2397 | 26.0155 | 13.453 | 9.57996 | 8.76031 | 22.7355 | 3.0623 | 10.5978 | 2.5064 | 0.4661 | 6.04E-03 | 1.41E-02 |
| sp\|O35218\|CF | Cpsf2 | CPSF2_MOUSE | 30 | 21.2873 | 45.6116 | 18.741 | 4.62706 | 4.55966 | 5.17343 | 28.5466 | 14.8334 | 4.7867 | 0.3366 | 0.1677 | 5.01E-02 | 7.39E-02 |
| sp\|P17182\|EN | Eno1 | ENOA_MOUSE | 30 | 25.4693 | 25.7187 | 28.1515 | 9.2152 | 6.07316 | 5.37217 | 26.4465 | 1.4818 | 6.8868 | 2.0467 | 0.2604 | 1.79E-04 | 1.97E-03 |
| sp\|P11276\|FIN | Fn1 | FINC_MOUSE | 28 | 2.13862 | 1.11397 | 1.33264 | 27.6434 | 47.4186 | 20.3528 | 1.5284 | 0.5396 | 31.8049 | 14.0046 | 20.8092 | 2.01E-02 | 3.49E-02 |
| sp\|P15331\|PE | Prph | PERI_MOUSE | 28 | 27.0825 | 21.5261 | 25.405 | 8.88943 | 10.2012 | 6.89589 | 24.6712 | 2.8500 | 8.6622 | 1.6643 | 0.3511 | 1.10E-03 | 4.89E-03 |
| sp\|P68372\|TB | Tubb4b | TBB4B_MOUSI | 28 | 24.0416 | 28.6063 | 29.9907 | 6.78869 | 4.95364 | 5.61911 | 27.5462 | 3.1130 | 5.7871 | 0.9290 | 0.2101 | 3.15E-04 | 2.57E-03 |
| sp\|P05213\|TB | Tuba1b | TBA1B_MOUSI | 27 | 22.6729 | 29.374 | 28.2415 | 7.75827 | 5.96097 | 5.99235 | 26.7628 | 3.5869 | 6.5705 | 1.0287 | 0.2455 | 7.22E-04 | 4.03E-03 |
| sp\|P11499\|HS | Hsp90ab1 | HS90B_MOUSI | 26 | 25.9002 | 31.2881 | 29.2298 | 5.73143 | 4.32491 | 3.52559 | 28.8060 | 2.7188 | 4.5273 | 1.1168 | 0.1572 | 1.39E-04 | 1.82E-03 |
| sp\|P52480-2\|I | Pkm | KPYM_MOUSE | 26 | 18.701 | 18.3518 | 26.4877 | 14.6427 | 10.8666 | 10.9503 | 21.1802 | 4.5998 | 12.1532 | 2.1564 | 0.5738 | 3.70E-02 | 5.69E-02 |
| sp\|P05064\|AL | Aldoa | ALDOA_MOUS | 25 | 22.9996 | 20.8503 | 32.5775 | 9.51241 | 7.2113 | 6.84884 | 25.4758 | 6.2434 | 7.8575 | 1.4446 | 0.3084 | 8.89E-03 | 1.86E-02 |
| sp\|P23116\|EIF | Eif3a | EIF3A_MOUSE | 25 | 23.0399 | 29.6721 | 22.1911 | 9.97394 | 7.9115 | 7.21146 | 24.9677 | 4.0962 | 8.3656 | 1.4361 | 0.3351 | 2.69E-03 | 7.94E-03 |
| sp\|Q9JKF1\|IQ | Iqgap1 | IQGA1_MOUSI | 25 | 15.091 | 14.056 | 16.2019 | 20.1421 | 21.3903 | 13.1187 | 15.1163 | 1.0732 | 18.2170 | 4.4592 | 1.2051 | 3.07E-01 | 3.67E-01 |
| sp\|P70398\|US | Usp9x | USP9X_MOUSI | 25 | 24.1582 | 32.3966 | 20.4475 | 8.40893 | 7.19477 | 7.394 | 25.6674 | 6.1158 | 7.6659 | 0.6511 | 0.2987 | 7.13E-03 | 1.59E-02 |
| sp\|Q64727\|VI | Vcl | VINC_MOUSE | 25 | 13.8885 | 12.2708 | 17.6569 | 25.4408 | 18.2495 | 12.4934 | 14.6054 | 2.7637 | 18.7279 | 6.4869 | 1.2823 | 3.69E-01 | 4.28E-01 |
| sp\|P10126\|EF | Eef1a1 | EF1A1_MOUSE | 24 | 23.6864 | 22.6293 | 24.3887 | 12.5135 | 9.08668 | 7.69545 | 23.5681 | 0.8856 | 9.7652 | 2.4797 | 0.4143 | 8.16E-04 | 4.29E-03 |
| sp\|Q99KI0\|AC | Aco2 | ACON_MOUSE | 23 | 25.9711 | 20.8774 | 32.6966 | 7.77234 | 6.17882 | 6.50375 | 26.5150 | 5.9283 | 6.8183 | 0.8420 | 0.2571 | 4.69E-03 | 1.17E-02 |
| sp\|P14824\|AN | Anxa6 | ANXA6_MOUS | 23 | 20.2618 | 15.413 | 25.3317 | 16.2032 | 11.8678 | 10.9226 | 20.3355 | 4.9598 | 12.9979 | 2.8158 | 0.6392 | 8.98E-02 | 1.24E-01 |
| sp\|P80313\|TC | Cct7 | TCPH_MOUSE | 23 | 26.4964 | 29.3352 | 27.4207 | 6.55746 | 5.38081 | 4.8094 | 27.7508 | 1.4479 | 5.5826 | 0.8913 | 0.2012 | 2.28E-05 | 1.11E-03 |
| sp\|P42932\|TC | Cct8 | TCPQ_MOUSE | 23 | 27.9141 | 28.2807 | 28.8178 | 5.85105 | 4.60215 | 4.53423 | 28.3375 | 0.4545 | 4.9958 | 0.7414 | 0.1763 | 1.28E-06 | 5.68E-04 |
| sp\|Q61316\|HS | Hspa4 | HSP74_MOUSI | 23 | 25.8322 | 25.3167 | 27.2558 | 9.10073 | 6.23061 | 6.2639 | 26.1349 | 1.0044 | 7.1984 | 1.6475 | 0.2754 | 7.02E-05 | 1.51E-03 |
| sp\|Q60864\|ST | Stip1 | STIP1_MOUSE | 23 | 31.4839 | 19.5392 | 23.9916 | 8.31802 | 6.93399 | 9.73325 | 25.0049 | 6.0365 | 8.3284 | 1.3997 | 0.3331 | 9.58E-03 | 1.95E-02 |
| sp\|P57780\|AC | Actn4 | ACTN4_MOUS | 22 | 13.353 | 11.9261 | 16.8514 | 23.9557 | 19.7119 | 14.2019 | 14.0435 | 2.5342 | 19.2898 | 4.8906 | 1.3736 | 1.74E-01 | 2.20E-01 |
| sp\|Q68FD5\|CL | Cltc | CLH1_MOUSE | 22 | 16.8262 | 19.5347 | 15.9957 | 19.1073 | 16.2269 | 12.3093 | 17.4522 | 1.8507 | 15.8812 | 3.4122 | 0.9100 | 5.22E-01 | 5.68E-01 |
| sp\|P03975\|IGI | Iap | IGEB_MOUSE | 22 | 28.7545 | 35.2778 | 24.7917 | 3.63456 | 3.62637 | 3.91509 | 29.6080 | 5.2949 | 3.7253 | 0.1644 | 0.1258 | 1.07E-03 | 4.81E-03 |
| sp\|Q6PB66\|LP | Lrpprc | LPPRC_MOUSI | 22 | 25.7472 | 26.2231 | 28.2001 | 7.04541 | 6.00338 | 6.78079 | 26.7235 | 1.3008 | 6.6099 | 0.5416 | 0.2473 | 1.59E-05 | 1.03E-03 |
| sp\|P29341\|PA | Pabpc1 | PABP1_MOUS | 22 | 18.9566 | 23.7077 | 18.0688 | 13.2751 | 15.0948 | 10.8971 | 20.2444 | 3.0320 | 13.0890 | 2.1050 | 0.6466 | 2.84E-02 | 4.57E-02 |
| sp\|P08003\|PD | Pdia4 | PDIA4_MOUSE | 22 | 23.8897 | 19.1497 | 24.6152 | 14.8139 | 9.34718 | 8.18426 | 22.5515 | 2.9683 | 10.7818 | 3.5400 | 0.4781 | 1.16E-02 | 2.24E-02 |
| sp\|Q6A4J8\|UE | Usp7 | UBP7_MOUSE | 22 | 23.4105 | 40.156 | 18.3338 | 6.08157 | 5.46468 | 6.5535 | 27.3001 | 11.4192 | 6.0333 | 0.5460 | 0.2210 | 3.22E-02 | 5.09E-02 |
| tr\|E9PWE8\|E9 | Dpysl3 | E9PWE8_MOU | 21 | 24.0596 | 32.0418 | 24.8638 | 6.82521 | 6.49768 | 5.71196 | 26.9884 | 4.3948 | 6.3450 | 0.5721 | 0.2351 | 1.28E-03 | 5.27E-03 |

| | | | | | | | | | | | | | | | | |
|---|---|---|---|---|---|---|---|---|---|---|---|---|---|---|---|---|
| sp\|Q8VHX6\|FL | Flnc | FLNC_MOUSE | 21 | 17.0366 | 20.8721 | 15.7178 | 19.163 | 16.1087 | 11.1018 | 17.8755 | 2.6776 | 15.4578 | 4.0698 | 0.8647 | 4.38E-01 | 4.90E-01 |
| sp\|Q02053\|UE | Uba1 | UBA1_MOUSE | 21 | 23.6241 | 21.5649 | 30.6554 | 10.5514 | 6.80997 | 6.79418 | 25.2815 | 4.7665 | 8.0519 | 2.1647 | 0.3185 | 4.68E-03 | 1.17E-02 |
| sp\|Q9WTQ5\|A | Akap12 | AKA12_MOUS | 20 | 25.805 | 23.7405 | 16.3246 | 14.2177 | 10.483 | 9.42923 | 21.9567 | 4.9856 | 11.3766 | 2.5162 | 0.5181 | 3.05E-02 | 4.86E-02 |
| sp\|Q03265\|AT | Atp5a1 | ATPA_MOUSE | 20 | 20.3188 | 21.4868 | 20.6126 | 14.6237 | 11.748 | 11.2101 | 20.8061 | 0.6076 | 12.5273 | 1.8354 | 0.6021 | 1.76E-03 | 6.03E-03 |
| sp\|P56480\|AT | Atp5b | ATPB_MOUSE | 20 | 22.3712 | 24.1786 | 26.928 | 11.1802 | 8.06933 | 7.27264 | 24.4926 | 2.2946 | 8.8407 | 2.0648 | 0.3610 | 9.27E-04 | 4.58E-03 |
| sp\|Q78PY7\|SN | Snd1 | SND1_MOUSE | 20 | 25.2385 | 24.8478 | 25.4018 | 10.0831 | 8.14135 | 6.28738 | 25.1627 | 0.2847 | 8.1706 | 1.8980 | 0.3247 | 1.05E-04 | 1.63E-03 |
| sp\|Q62261\|SP | Sptbn1 | SPTB2_MOUSE | 20 | 15.5147 | 17.4845 | 13.5837 | 19.5268 | 21.3016 | 12.5887 | 15.5276 | 1.9504 | 17.8057 | 4.6044 | 1.1467 | 4.74E-01 | 5.24E-01 |
| sp\|P14211\|CA | Calr | CALR_MOUSE | 19 | 28.3023 | 16.1576 | 25.961 | 13.438 | 8.56671 | 7.57436 | 23.4736 | 6.4431 | 9.8597 | 3.1384 | 0.4200 | 3.02E-02 | 4.82E-02 |
| sp\|P80315\|TC | Cct4 | TCPD_MOUSE | 19 | 25.7356 | 28.0214 | 26.7933 | 7.97537 | 6.091 | 5.3833 | 26.8501 | 1.1440 | 6.4832 | 1.3398 | 0.2415 | 3.67E-05 | 1.17E-03 |
| sp\|P80316\|TC | Cct5 | TCPE_MOUSE | 19 | 26.7683 | 29.0553 | 27.1111 | 7.11134 | 5.16123 | 4.7928 | 27.6449 | 1.2334 | 5.6885 | 1.2459 | 0.2058 | 2.67E-05 | 1.13E-03 |
| sp\|Q922B2\|SY | Dars | SYDC_MOUSE | 19 | 26.3608 | 27.5001 | 24.2117 | 8.70624 | 6.90315 | 6.31809 | 26.0242 | 1.6698 | 7.3092 | 1.2448 | 0.2809 | 9.95E-05 | 1.63E-03 |
| sp\|Q6NZJ6\|IF | Eif4g1 | IF4G1_MOUSE | 19 | 22.7585 | 24.7724 | 21.9898 | 9.82375 | 10.6071 | 10.0485 | 23.1736 | 1.4370 | 10.1598 | 0.4034 | 0.4384 | 1.12E-04 | 1.65E-03 |
| sp\|Q8VEK3\|HI | Hnrnpu | HNRPU_MOUS | 19 | 21.0424 | 29.1306 | 16.0048 | 13.0746 | 12.6127 | 8.13489 | 22.0593 | 6.6217 | 11.2741 | 2.7284 | 0.5111 | 5.95E-02 | 8.58E-02 |
| sp\|P09103\|PD | P4hb | PDIA1_MOUSE | 19 | 16.8503 | 12.5993 | 15.4324 | 25.2297 | 17.1776 | 12.7107 | 14.9607 | 2.1644 | 18.3727 | 6.3445 | 1.2281 | 4.28E-01 | 4.81E-01 |
| sp\|P40142\|TK | Tkt | TKT_MOUSE T | 19 | 23.9134 | 20.9763 | 27.8924 | 11.5736 | 8.2338 | 7.41046 | 24.2607 | 3.4711 | 9.0726 | 2.2047 | 0.3740 | 3.07E-03 | 8.70E-03 |
| sp\|P80314\|TC | Cct2 | TCPB_MOUSE | 18 | 27.1464 | 28.9375 | 28.4806 | 6.11374 | 4.85416 | 4.46757 | 28.1882 | 0.9307 | 5.1452 | 0.8608 | 0.1825 | 6.07E-06 | 8.20E-04 |
| sp\|Q9QXK7\|CI | Cpsf3 | CPSF3_MOUSE | 18 | 22.4163 | 46.2188 | 17.996 | 4.47441 | 3.9721 | 4.92242 | 28.8770 | 15.1802 | 4.4563 | 0.4754 | 0.1543 | 4.96E-02 | 7.32E-02 |
| sp\|Q9CZD3\|SY | Gars | SYG_MOUSE G | 18 | 27.3673 | 24.5479 | 30.6788 | 6.79202 | 4.93367 | 5.68028 | 27.5313 | 3.0687 | 5.8020 | 0.9351 | 0.2107 | 3.02E-04 | 2.52E-03 |
| sp\|P17710\|HX | Hk1 | HXK1_MOUSE | 18 | 22.3986 | 29.6682 | 24.8231 | 8.7389 | 7.14844 | 7.22278 | 25.6300 | 3.7014 | 7.7034 | 0.8976 | 0.3006 | 1.23E-03 | 5.17E-03 |
| sp\|P17156\|HS | Hspa2 | HSP72_MOUSI | 18 | 24.5759 | 26.8098 | 23.5769 | 10.2402 | 7.97555 | 6.82166 | 24.9875 | 1.6553 | 8.3458 | 1.7391 | 0.3340 | 2.76E-04 | 2.44E-03 |
| sp\|Q9JKR6\|HY | Hyou1 | HYOU1_MOUS | 18 | 25.3578 | 22.1726 | 29.845 | 9.38297 | 7.28575 | 5.95586 | 25.7918 | 3.8546 | 7.5415 | 1.7278 | 0.2924 | 1.71E-03 | 5.98E-03 |
| sp\|P62908\|RS | Rps3 | RS3_MOUSE 4 | 18 | 21.9073 | 25.4501 | 18.9856 | 12.5588 | 12.5567 | 8.54155 | 22.1143 | 3.2372 | 11.2190 | 2.3188 | 0.5073 | 9.04E-03 | 1.87E-02 |
| sp\|Q64514-2\| | Tpp2 | TPP2_MOUSE | 18 | 24.9419 | 23.3358 | 26.4951 | 9.19402 | 8.3601 | 7.67306 | 24.9243 | 1.5797 | 8.4091 | 0.7617 | 0.3374 | 8.27E-05 | 1.60E-03 |
| sp\|Q6ZQ38\|CA | Cand1 | CAND1_MOUS | 17 | 23.6106 | 24.2102 | 25.4153 | 10.6049 | 7.71171 | 8.44727 | 24.4120 | 0.9191 | 8.9213 | 1.5037 | 0.3654 | 1.09E-04 | 1.64E-03 |
| sp\|P80318\|TC | Cct3 | TCPG_MOUSE | 17 | 25.4245 | 29.3302 | 28.3592 | 7.18357 | 4.64242 | 5.06005 | 27.7046 | 2.0335 | 5.6287 | 1.3627 | 0.2032 | 9.81E-05 | 1.63E-03 |
| sp\|Q3U1J4\|DD | Ddb1 | DDB1_MOUSE | 17 | 23.9597 | 26.7355 | 24.1708 | 9.65355 | 7.39143 | 8.08899 | 24.9553 | 1.5453 | 8.3780 | 1.1584 | 0.3357 | 1.19E-04 | 1.73E-03 |
| sp\|P60843\|IF | Eif4a1 | IF4A1_MOUSE | 17 | 19.2602 | 18.4618 | 23.051 | 13.6544 | 15.2426 | 10.3299 | 20.2577 | 2.4518 | 13.0756 | 2.5070 | 0.6455 | 2.39E-02 | 3.99E-02 |
| sp\|P05202\|AA | Got2 | AATM_MOUSE | 17 | 26.0277 | 25.6215 | 28.0651 | 7.25425 | 6.86965 | 6.16178 | 26.5714 | 1.3094 | 6.7619 | 0.5541 | 0.2545 | 1.75E-05 | 1.03E-03 |
| sp\|Q8BU30\|SY | Iars | SYIC_MOUSE I | 17 | 23.9919 | 27.1761 | 22.883 | 10.3214 | 8.13006 | 7.49751 | 24.6837 | 2.2286 | 8.6497 | 1.4819 | 0.3504 | 4.87E-04 | 3.26E-03 |
| sp\|P08249\|MI | Mdh2 | MDHM_MOUS | 17 | 22.0175 | 21.8444 | 27.1706 | 13.2139 | 8.54979 | 7.20381 | 23.6775 | 3.0264 | 9.6558 | 3.1540 | 0.4078 | 5.14E-03 | 1.26E-02 |
| sp\|Q3V3R1\|C1 | Mthfd1l | C1TM_MOUSE | 17 | 27.4385 | 25.9695 | 29.6152 | 5.70017 | 5.72886 | 5.54778 | 27.6744 | 1.8343 | 5.6589 | 0.0973 | 0.2045 | 3.18E-05 | 1.17E-03 |
| sp\|O35685\|NU | Nudc | NUDC_MOUSE | 17 | 35.9103 | 20.2194 | 28.8812 | 5.08675 | 5.52746 | 4.37483 | 28.3370 | 7.8596 | 4.9963 | 0.5816 | 0.1763 | 6.84E-03 | 1.54E-02 |
| sp\|P11983\|TC | Tcp1 | TCPA_MOUSE | 17 | 26.2396 | 31.7941 | 25.6934 | 6.70469 | 4.98057 | 4.58761 | 27.9090 | 3.3756 | 5.4243 | 1.1261 | 0.1944 | 3.96E-04 | 2.89E-03 |
| sp\|Q9Z1Q9\|SY | Vars | SYVC_MOUSE | 17 | 25.2099 | 31.394 | 22.5013 | 7.88655 | 6.30655 | 6.70167 | 26.3684 | 4.5581 | 6.9649 | 0.8222 | 0.2641 | 1.92E-03 | 6.41E-03 |
| sp\|B2RQC6\|PY | Cad | PYR1_MOUSE | 16 | 24.8621 | 31.3943 | 22.2854 | 8.05822 | 6.91112 | 6.48887 | 26.1806 | 4.6954 | 7.1527 | 0.8121 | 0.2732 | 2.29E-03 | 7.09E-03 |
| sp\|Q61656\|DD | Ddx5 | DDX5_MOUSE | 16 | 18.5388 | 22.8215 | 14.5768 | 15.1892 | 18.7368 | 10.1369 | 18.6457 | 4.1234 | 14.6876 | 4.3218 | 0.7877 | 3.15E-01 | 3.75E-01 |
| sp\|P26040\|EZ | Ezr | EZRI_MOUSE E | 16 | 15.9998 | 15.9983 | 18.3172 | 21.5408 | 15.6913 | 12.4526 | 16.7718 | 1.3384 | 16.5616 | 4.6062 | 0.9875 | 9.43E-01 | 9.54E-01 |
| sp\|Q8VDJ3\|VI | Hdlbp | VIGLN_MOUSE | 16 | 15.6859 | 16.8917 | 17.3279 | 21.1017 | 18.5579 | 10.4348 | 16.6352 | 0.8505 | 16.6981 | 5.5713 | 1.0038 | 9.85E-01 | 9.87E-01 |
| sp\|Q8CAQ8\|IN | Immt | IMMT_MOUSE | 16 | 23.2929 | 30.5331 | 19.4185 | 10.5426 | 8.86868 | 7.34417 | 24.4148 | 5.6416 | 8.9185 | 1.5998 | 0.3653 | 1.02E-02 | 2.05E-02 |
| sp\|Q8CGK3\|LC | Lonp1 | LONM_MOUSI | 16 | 25.1654 | 26.1926 | 27.8628 | 8.13917 | 6.47842 | 6.16164 | 26.4069 | 1.3614 | 6.9264 | 1.0622 | 0.2623 | 4.04E-05 | 1.19E-03 |
| sp\|Q91YQ5\|RI | Rpn1 | RPN1_MOUSE | 16 | 23.7313 | 28.1354 | 22.3175 | 11.0863 | 7.77566 | 6.95389 | 24.7281 | 3.0343 | 8.6053 | 2.1876 | 0.3480 | 1.72E-03 | 5.98E-03 |
| sp\|Q8VIJ6\|SFF | Sfpq | SFPQ_MOUSE | 16 | 12.0127 | 13.7055 | 11.4579 | 20.9635 | 34.3555 | 7.50491 | 12.3920 | 1.1708 | 20.9413 | 13.4253 | 1.6899 | 3.34E-01 | 3.95E-01 |
| sp\|Q8BGQ7\|S | Aars | SYAC_MOUSE | 15 | 22.8147 | 23.8967 | 27.5118 | 9.94646 | 9.01205 | 6.81825 | 24.7411 | 2.4598 | 8.5923 | 1.6058 | 0.3473 | 6.79E-04 | 3.87E-03 |
| sp\|Q7TPR4\|AC | Actn1 | ACTN1_MOUS | 15 | 11.184 | 10.3627 | 13.6803 | 24.6433 | 24.7388 | 15.3909 | 11.7423 | 1.7278 | 21.5910 | 5.3697 | 1.8387 | 3.90E-02 | 5.95E-02 |
| sp\|Q9DBR0\|AI | Akap8 | AKAP8_MOUS | 15 | 22.4611 | 41.2197 | 14.6109 | 8.21347 | 6.53432 | 6.9606 | 26.0972 | 13.6720 | 7.2361 | 0.8728 | 0.2773 | 7.56E-02 | 1.06E-01 |
| sp\|O55143\|AT | Atp2a2 | AT2A2_MOUSI | 15 | 22.7909 | 22.5664 | 21.6526 | 12.5556 | 10.9653 | 9.46914 | 22.3366 | 0.6029 | 10.9967 | 1.5435 | 0.4923 | 2.90E-04 | 2.46E-03 |
| sp\|P35564\|CA | Canx | CALX_MOUSE | 15 | 21.0609 | 26.9117 | 23.4595 | 13.0321 | 9.03308 | 6.50259 | 23.8107 | 2.9412 | 9.5226 | 3.2922 | 0.3999 | 4.97E-03 | 1.22E-02 |
| sp\|P80317\|TC | Cct6a | TCPZ_MOUSE | 15 | 25.4412 | 24.7214 | 25.1316 | 8.14888 | 6.66196 | 9.89489 | 25.0981 | 0.3611 | 8.2352 | 1.6182 | 0.3281 | 6.10E-05 | 1.41E-03 |
| sp\|Q8CIE6\|CO | Copa | COPA_MOUSE | 15 | 18.1885 | 22.4816 | 17.8018 | 17.3177 | 14.1279 | 10.0826 | 19.4906 | 2.5975 | 13.8427 | 3.6260 | 0.7102 | 9.34E-02 | 1.28E-01 |
| sp\|O08553\|DF | Dpysl2 | DPYL2_MOUSE | 15 | 21.1193 | 22.6244 | 22.0253 | 13.9055 | 10.9993 | 9.32626 | 21.9230 | 0.7577 | 11.4104 | 2.3171 | 0.5205 | 1.72E-03 | 5.98E-03 |
| sp\|Q922D8\|C1 | Mthfd1 | C1TC_MOUSE | 15 | 22.3495 | 22.4371 | 24.8668 | 10.6656 | 11.4228 | 8.25813 | 23.2178 | 1.4287 | 10.1155 | 1.6525 | 0.4357 | 4.85E-04 | 3.26E-03 |
| sp\|Q9EQK5\|M | Mvp | MVP_MOUSE I | 15 | 9.79148 | 15.0178 | 7.94275 | 27.7244 | 32.2553 | 7.26828 | 10.9173 | 3.6694 | 22.4160 | 13.3125 | 2.0532 | 2.23E-01 | 2.75E-01 |
| sp\|Q11011\|PS | Npepps | PSA_MOUSE P | 15 | 19.9031 | 22.4503 | 24.0841 | 12.4837 | 9.94583 | 11.133 | 22.1458 | 2.1071 | 11.1875 | 1.2698 | 0.5052 | 1.52E-03 | 5.72E-03 |
| sp\|Q9D0I9\|SY | Rars | SYRC_MOUSE | 15 | 24.2755 | 27.7836 | 24.341 | 8.58211 | 7.98744 | 7.03041 | 25.4667 | 2.0068 | 7.8667 | 0.7829 | 0.3089 | 1.45E-04 | 1.84E-03 |
| sp\|Q62318\|TII | Trim28 | TIF1B_MOUSE | 15 | 25.4329 | 36.2784 | 22.3305 | 6.17642 | 5.10532 | 4.67648 | 28.0139 | 7.3234 | 5.3194 | 0.7725 | 0.1899 | 5.93E-03 | 1.39E-02 |
| sp\|Q6P5F9\|XP | Xpo1 | XPO1_MOUSE | 15 | 27.6051 | 27.2611 | 29.6952 | 5.496 | 4.66235 | 5.28025 | 28.1871 | 1.3173 | 5.1462 | 0.4327 | 0.1826 | 8.67E-06 | 8.92E-04 |
| sp\|Q61024\|AS | Asns | ASNS_MOUSE | 14 | 28.8742 | 26.6555 | 31.1022 | 5.25298 | 4.01886 | 4.09623 | 28.8773 | 2.2234 | 4.4560 | 0.6913 | 0.1543 | 5.40E-05 | 1.38E-03 |
| sp\|Q9CWJ9\|PI | Atic | PUR9_MOUSE | 14 | 25.473 | 20.2029 | 29.3996 | 10.9746 | 8.38563 | 5.56421 | 25.0252 | 4.6147 | 8.3081 | 2.7060 | 0.3320 | 5.64E-03 | 1.35E-02 |
| sp\|Q3USJ8\|FC | Fchsd2 | FCSD2_MOUSI | 14 | 18.7589 | 33.0243 | 14.0264 | 12.4237 | 11.0701 | 10.6967 | 21.9365 | 9.8895 | 11.3968 | 0.9087 | 0.5195 | 1.40E-01 | 1.82E-01 |
| tr\|E9PVA8\|E9I | Gcn1l1 | E9PVA8_MOU | 14 | 22.2698 | 26.0012 | 22.7561 | 10.5281 | 9.36628 | 9.07857 | 23.6757 | 2.0286 | 9.6577 | 0.7674 | 0.4079 | 3.63E-04 | 2.74E-03 |
| sp\|O08528\|HX | Hk2 | HXK2_MOUSE | 14 | 25.9862 | 30.5032 | 27.0032 | 5.92319 | 4.88234 | 5.70192 | 27.8309 | 2.3695 | 5.5025 | 0.5483 | 0.1977 | 9.14E-05 | 1.63E-03 |
| sp\|P61979-2\|I | Hnrnpk | HNRPK_MOUS | 14 | 20.7901 | 21.9481 | 18.8222 | 14.9354 | 13.8509 | 9.65334 | 20.5201 | 1.5803 | 12.8132 | 2.7897 | 0.6244 | 1.41E-02 | 2.63E-02 |
| sp\|P09411\|PG | Pgk1 | PGK1_MOUSE | 14 | 21.2707 | 18.7912 | 28.3522 | 13.6324 | 9.09506 | 8.85847 | 22.8047 | 4.9617 | 10.5286 | 2.6905 | 0.4617 | 1.97E-02 | 3.44E-02 |

| | | | | | | | | | | | | | | | | |
|---|---|---|---|---|---|---|---|---|---|---|---|---|---|---|---|---|
| sp\|Q61753\|SE | Phgdh | SERA_MOUSE | 14 | 25.1882 | 25.5238 | 28.0282 | 7.79925 | 7.49759 | 5.96307 | 26.2467 | 1.5519 | 7.0866 | 0.9847 | 0.2700 | 5.53E-05 | 1.38E-03 |
| sp\|Q9D0R2\|S\ | Tars | SYTC_MOUSE | 14 | 25.4807 | 23.6935 | 27.5138 | 9.60225 | 7.20883 | 6.50095 | 25.5627 | 1.9115 | 7.7707 | 1.6252 | 0.3040 | 2.52E-04 | 2.32E-03 |
| sp\|Q91WQ3\|S | Yars | SYYC_MOUSE | 14 | 28.5059 | 21.8973 | 30.4182 | 7.48429 | 5.76638 | 5.92795 | 26.9405 | 4.4709 | 6.3929 | 0.9486 | 0.2373 | 1.47E-03 | 5.60E-03 |
| sp\|Q9D8N0\|EI | Eef1g | EF1G_MOUSE | 13 | 23.2081 | 27.8347 | 22.7266 | 10.9434 | 8.42758 | 6.85959 | 24.5898 | 2.8205 | 8.7435 | 2.0602 | 0.3556 | 1.42E-03 | 5.48E-03 |
| sp\|Q8BHN3\|G | Ganab | GANAB_MOUS | 13 | 21.9523 | 21.3281 | 24.0557 | 13.7622 | 9.67934 | 9.22243 | 22.4454 | 1.4291 | 10.8880 | 2.4996 | 0.4851 | 2.25E-03 | 7.03E-03 |
| sp\|P68040\|GB | Gnb2l1 | GBLP_MOUSE | 13 | 21.8327 | 25.8942 | 24.3062 | 10.2256 | 9.13571 | 8.60561 | 24.0110 | 2.0468 | 9.3223 | 0.8260 | 0.3883 | 3.23E-04 | 2.60E-03 |
| sp\|Q61699\|HS | Hsph1 | HS105_MOUSI | 13 | 27.8195 | 29.5825 | 26.6171 | 6.9942 | 4.743 | 4.24365 | 28.0064 | 1.4915 | 5.3270 | 1.4653 | 0.1902 | 4.73E-05 | 1.33E-03 |
| sp\|Q99MN1\|S | Kars | SYK_MOUSE Ly | 13 | 26.5783 | 31.174 | 25.6443 | 6.85382 | 4.48994 | 5.25973 | 27.7989 | 2.9600 | 5.5345 | 1.2057 | 0.1991 | 2.71E-04 | 2.42E-03 |
| sp\|Q9QUR6\|P | Prep | PPCE_MOUSE | 13 | 24.5697 | 19.4611 | 31.7726 | 7.029 | 7.19579 | 9.97174 | 25.2678 | 6.1854 | 8.0655 | 1.6529 | 0.3192 | 9.63E-03 | 1.96E-02 |
| sp\|P14148\|RL | Rpl7 | RL7_MOUSE 6 | 13 | 16.5889 | 19.3124 | 12.8044 | 18.6118 | 20.9847 | 11.6978 | 16.2352 | 3.2684 | 17.0981 | 4.8249 | 1.0531 | 8.10E-01 | 8.38E-01 |
| sp\|Q99PL5\|RR | Rrbp1 | RRBP1_MOUSI | 13 | 10.8528 | 9.83293 | 6.69686 | 27.2307 | 30.0729 | 15.3138 | 9.1275 | 2.1659 | 24.2058 | 7.8307 | 2.6520 | 3.25E-02 | 5.11E-02 |
| sp\|Q6P5E4\|U | Uggt1 | UGGG1_MOUS | 13 | 21.5994 | 24.8149 | 24.5381 | 11.3123 | 9.59604 | 8.13921 | 23.6508 | 1.7819 | 9.6825 | 1.5883 | 0.4094 | 5.33E-04 | 3.43E-03 |
| sp\|P47738\|AL | Aldh2 | ALDH2_MOUS | 12 | 21.3686 | 21.6239 | 24.6863 | 12.9054 | 9.72799 | 9.68779 | 22.5596 | 1.8462 | 10.7737 | 1.8462 | 0.4776 | 1.44E-03 | 5.53E-03 |
| sp\|Q8BMK4\|C | Ckap4 | CKAP4_MOUS | 12 | 11.496 | 13.2821 | 9.1036 | 28.8362 | 22.5842 | 14.6979 | 11.2939 | 2.0966 | 22.0394 | 7.0849 | 1.9514 | 6.54E-02 | 9.33E-02 |
| sp\|Q6ZQ08\|C | Cnot1 | CNOT1_MOUS | 12 | 22.9494 | 29.0619 | 22.0647 | 9.18397 | 8.13508 | 8.60499 | 24.6920 | 3.8102 | 8.6413 | 0.5254 | 0.3500 | 1.94E-03 | 6.47E-03 |
| sp\|Q8R1B4\|EI | Eif3c | EIF3C_MOUSE | 12 | 24.2239 | 29.3964 | 22.1157 | 9.39769 | 7.72649 | 7.13985 | 25.2453 | 3.7463 | 8.0880 | 1.1715 | 0.3204 | 1.63E-03 | 5.93E-03 |
| sp\|Q8QZY1\|EI | Eif3l | EIF3L_MOUSE | 12 | 24.1883 | 27.5006 | 22.7178 | 10.4446 | 7.24211 | 7.90648 | 24.8022 | 2.4498 | 8.5311 | 1.6901 | 0.3440 | 6.94E-04 | 3.91E-03 |
| sp\|Q6A0A9\|F1 | FAM120A | F120A_MOUSI | 12 | 6.97046 | 9.20095 | 5.25571 | 28.7802 | 26.8107 | 22.982 | 7.1424 | 1.9782 | 26.1910 | 2.9484 | 3.6670 | 7.46E-04 | 4.08E-03 |
| sp\|P97807\|FU | Fh | FUMH_MOUSI | 12 | 24.7676 | 24.5738 | 27.6713 | 9.21293 | 7.19847 | 6.57594 | 25.6709 | 1.7351 | 7.6624 | 1.3784 | 0.2985 | 1.48E-04 | 1.85E-03 |
| sp\|P16858\|G3 | Gapdh | G3P_MOUSE G | 12 | 19.0939 | 20.2301 | 23.488 | 16.7689 | 11.2143 | 9.20491 | 20.9373 | 2.2808 | 12.3960 | 3.9180 | 0.5921 | 3.10E-02 | 4.92E-02 |
| sp\|O88569\|RC | Hnrnpa2b1 | ROA2_MOUSE | 12 | 19.5457 | 18.2354 | 17.5448 | 17.6428 | 15.1798 | 11.8514 | 18.4420 | 1.0163 | 14.8913 | 2.9065 | 0.8075 | 1.16E-01 | 1.55E-01 |
| sp\|P70168\|IM | Kpnb1 | IMB1_MOUSE | 12 | 21.1873 | 23.4014 | 25.6633 | 12.8297 | 8.72748 | 8.19074 | 23.4173 | 2.2380 | 9.9160 | 2.5376 | 0.4234 | 2.30E-03 | 7.09E-03 |
| sp\|Q8BMJ2\|S\ | Lars | SYLC_MOUSE | 12 | 22.3186 | 22.3307 | 23.0105 | 13.093 | 9.84425 | 9.40284 | 22.5533 | 0.3960 | 10.7800 | 2.0152 | 0.4780 | 5.78E-04 | 3.53E-03 |
| sp\|Q9WU78\|F | Pdcd6ip | PDC6I_MOUSE | 12 | 18.7084 | 19.3175 | 21.2886 | 15.5472 | 13.1597 | 11.9786 | 19.7715 | 1.3487 | 13.5618 | 1.8180 | 0.6859 | 8.96E-03 | 1.86E-02 |
| sp\|P97351\|RS | Rps3a | RS3A_MOUSE | 12 | 18.0085 | 22.5645 | 14.0478 | 17.0393 | 19.0899 | 9.25016 | 18.2069 | 4.2618 | 15.1265 | 5.1913 | 0.8308 | 4.71E-01 | 5.22E-01 |
| tr\|Q9CZN7\|Q | Shmt2 | Q9CZN7_MOU | 12 | 24.795 | 25.4563 | 28.5264 | 7.71022 | 6.56332 | 6.94876 | 26.2592 | 1.9911 | 7.0741 | 0.5836 | 0.2694 | 8.89E-05 | 1.63E-03 |
| sp\|P48962\|AD | Slc25a4 | ADT1_MOUSE | 12 | 24.9293 | 26.8288 | 23.7903 | 9.1441 | 8.76722 | 6.54029 | 25.1828 | 1.5350 | 8.1505 | 1.4072 | 0.3237 | 1.44E-04 | 1.84E-03 |
| sp\|P10852\|4F | Slc3a2 | 4F2_MOUSE 4 | 12 | 28.6508 | 28.8544 | 28.4157 | 5.46078 | 4.51294 | 4.10536 | 28.6403 | 0.2195 | 4.6930 | 0.6954 | 0.1639 | 5.72E-07 | 5.68E-04 |
| sp\|Q8CG48\|SI | Smc2 | SMC2_MOUSE | 12 | 25.3495 | 30.4004 | 26.1112 | 5.31727 | 6.22637 | 6.59524 | 27.2870 | 2.7230 | 6.0463 | 0.6577 | 0.2216 | 1.94E-04 | 2.08E-03 |
| sp\|Q9Z110\|P5 | Aldh18a1 | P5CS_MOUSE | 11 | 24.4132 | 22.916 | 25.0418 | 10.5974 | 8.76828 | 8.26323 | 24.1237 | 1.0921 | 9.2096 | 1.2281 | 0.3818 | 9.57E-05 | 1.63E-03 |
| sp\|Q8VDN2\|A | Atp1a1 | AT1A1_MOUSI | 11 | 19.7268 | 22.205 | 19.8451 | 16.703 | 12.067 | 9.45308 | 20.5923 | 1.3979 | 12.7410 | 3.6717 | 0.6187 | 2.58E-02 | 4.26E-02 |
| sp\|Q91VR5\|DI | Ddx1 | DDX1_MOUSE | 11 | 13.5702 | 16.6601 | 12.8126 | 19.1307 | 24.0721 | 13.7543 | 14.3476 | 2.0382 | 18.9857 | 5.1604 | 1.3233 | 2.21E-01 | 2.73E-01 |
| sp\|Q501J6\|DI | Ddx17 | DDX17_MOUS | 11 | 15.4187 | 19.362 | 13.5604 | 19.1063 | 21.358 | 11.1947 | 16.1137 | 2.9626 | 17.2197 | 5.3379 | 1.0686 | 7.69E-01 | 7.98E-01 |
| sp\|Q62167\|DI | Ddx3x | DDX3X_MOUS | 11 | 14.5337 | 18.1643 | 12.0766 | 19.8538 | 22.5975 | 12.7741 | 14.9249 | 3.0626 | 18.4085 | 5.0687 | 1.2334 | 3.66E-01 | 4.26E-01 |
| sp\|Q8BMF4\|O | Dlat | ODP2_MOUSE | 11 | 12.4012 | 21.3452 | 8.6901 | 17.8837 | 18.3913 | 21.2885 | 14.1455 | 6.5054 | 19.1878 | 1.8368 | 1.3565 | 2.66E-01 | 3.22E-01 |
| sp\|P06745\|G6 | Gpi | G6PI_MOUSE | 11 | 26.1739 | 21.704 | 33.9192 | 7.10662 | 5.06721 | 6.02916 | 27.2657 | 6.1804 | 6.0677 | 1.0203 | 0.2225 | 4.23E-03 | 1.08E-02 |
| sp\|P62806\|H4 | Hist1h4a | H4_MOUSE Hi | 11 | 5.11541 | 7.99662 | 4.51708 | 31.5248 | 26.1994 | 24.6467 | 5.8764 | 1.8604 | 27.4570 | 3.6074 | 4.6724 | 7.72E-04 | 4.17E-03 |
| sp\|Q3U0V1\|FI | Khsrp | FUBP2_MOUS | 11 | 31.1086 | 23.8978 | 22.5871 | 8.91607 | 6.23797 | 7.25249 | 25.8645 | 4.5886 | 7.4688 | 1.3521 | 0.2888 | 2.64E-03 | 7.84E-03 |
| sp\|P06151\|LD | Ldha | LDHA_MOUSE | 11 | 26.6929 | 23.6834 | 32.5661 | 8.15901 | 4.8214 | 4.07714 | 27.6475 | 4.5176 | 5.6859 | 2.1739 | 0.2057 | 1.62E-03 | 5.92E-03 |
| sp\|P14733\|LN | Lmnb1 | LMNB1_MOUS | 11 | 19.9957 | 21.7759 | 18.5287 | 14.0553 | 11.7517 | 13.8928 | 20.1001 | 1.6261 | 13.2333 | 1.2856 | 0.6584 | 4.57E-03 | 1.15E-02 |
| sp\|P17742\|PP | Ppia | PPIA_MOUSE | 11 | 24.74 | 21.7046 | 24.9405 | 12.1601 | 8.71568 | 7.73911 | 23.7950 | 1.8131 | 9.5383 | 2.3225 | 0.4009 | 1.11E-03 | 4.90E-03 |
| sp\|Q76MZ3\|2 | Ppp2r1a | 2AAA_MOUSE | 11 | 22.3565 | 22.4995 | 26.1943 | 11.1291 | 7.71447 | 10.1061 | 23.6834 | 2.1756 | 9.6499 | 1.7524 | 0.4075 | 9.61E-04 | 4.62E-03 |
| sp\|P35700\|PR | Prdx1 | PRDX1_MOUS | 11 | 22.6676 | 21.4673 | 20.0306 | 15.1809 | 10.1746 | 10.479 | 21.3885 | 1.3203 | 11.9448 | 2.8066 | 0.5585 | 6.20E-03 | 1.43E-02 |
| sp\|P62192\|PR | Psmc1 | PRS4_MOUSE | 11 | 23.4511 | 26.8852 | 22.3349 | 11.1264 | 8.70934 | 7.49305 | 24.2237 | 2.3715 | 9.1096 | 1.8494 | 0.3761 | 9.59E-04 | 4.62E-03 |
| sp\|P47911\|RL | Rpl6 | RL6_MOUSE 6 | 11 | 14.6997 | 20.3393 | 12.027 | 19.553 | 22.8883 | 10.4927 | 15.6887 | 4.2435 | 17.6447 | 6.4144 | 1.1247 | 6.82E-01 | 7.18E-01 |
| sp\|P62270\|RS | Rps18 | RS18_MOUSE | 11 | 18.4822 | 20.9448 | 13.6723 | 18.5144 | 16.8232 | 11.5631 | 17.6998 | 3.6988 | 15.6336 | 3.6251 | 0.8833 | 5.28E-01 | 5.73E-01 |
| sp\|Q9WVA4\|T | Tagln2 | TAGL2_MOUSI | 11 | 28.9633 | 20.9318 | 24.7707 | 10.8479 | 7.05013 | 7.43613 | 24.8886 | 4.0170 | 8.4447 | 2.0901 | 0.3393 | 3.26E-03 | 9.09E-03 |
| sp\|P21107\|TP | Tpm3 | TPM3_MOUSE | 11 | 14.0939 | 6.16334 | 12.1164 | 23.5826 | 27.874 | 16.1698 | 10.7912 | 4.1280 | 22.5421 | 5.9211 | 2.0889 | 4.78E-02 | 7.10E-02 |
| sp\|Q9EPU0\|RI | Upf1 | RENT1_MOUSI | 11 | 10.2596 | 12.2196 | 8.58528 | 24.2241 | 25.3941 | 19.3174 | 10.3548 | 1.8190 | 22.9785 | 3.2242 | 2.2191 | 4.11E-03 | 1.06E-02 |
| sp\|Q9QUJ7\|A | Acsl4 | ACSL4_MOUSE | 10 | 25.7554 | 25.7078 | 26.8634 | 8.01137 | 6.40846 | 7.25353 | 26.1089 | 0.6539 | 7.2245 | 0.8019 | 0.2767 | 5.97E-06 | 8.20E-04 |
| sp\|P50516\|VA | Atp6v1a | VATA_MOUSE | 10 | 21.6349 | 23.3708 | 24.9363 | 11.4201 | 10.0734 | 8.5643 | 23.3140 | 1.6514 | 10.0193 | 1.4287 | 0.4298 | 4.57E-04 | 3.14E-03 |
| sp\|Q9CZU6\|CI | Cs | CISY_MOUSE | 10 | 24.8278 | 22.1459 | 28.3676 | 9.76478 | 7.71674 | 7.17721 | 25.1138 | 3.1207 | 8.2196 | 1.3651 | 0.3273 | 1.01E-03 | 4.73E-03 |
| sp\|Q9ERK4\|XF | Cse1l | XPO2_MOUSE | 10 | 26.4532 | 27.7349 | 28.2369 | 5.63636 | 4.68378 | 7.25485 | 27.4750 | 0.9198 | 5.8583 | 1.2998 | 0.2132 | 1.94E-05 | 1.09E-03 |
| sp\|Q00612\|G6 | G6pdx | G6PD1_MOUS | 10 | 25.678 | 21.5053 | 27.5582 | 9.89885 | 7.28668 | 8.07302 | 24.9138 | 3.0980 | 8.4195 | 1.3401 | 0.3379 | 1.07E-03 | 4.81E-03 |
| sp\|Q8BMS1\|E | Hadha | ECHA_MOUSE | 10 | 19.012 | 20.283 | 19.5947 | 17.7643 | 12.9868 | 10.3592 | 19.6299 | 0.6362 | 13.7034 | 3.7542 | 0.6981 | 5.43E-02 | 7.91E-02 |
| sp\|Q9D0E1\|HI | Hnrnpm | HNRPM_MOU | 10 | 15.3796 | 21.3469 | 12.5503 | 20.1332 | 17.9058 | 12.6843 | 16.4256 | 4.4906 | 16.9078 | 3.8234 | 1.0294 | 8.94E-01 | 9.10E-01 |
| sp\|O88844\|ID | Idh1 | IDHC_MOUSE | 10 | 22.2083 | 20.5321 | 24.5882 | 12.9249 | 9.70536 | 10.0412 | 22.4429 | 2.0382 | 10.8905 | 1.7698 | 0.4853 | 1.77E-03 | 6.03E-03 |
| sp\|P27546\|M | Map4 | MAP4_MOUSE | 10 | 17.0162 | 14.8454 | 10.7438 | 26.2375 | 19.1764 | 11.9807 | 14.2018 | 3.1853 | 19.1315 | 7.1285 | 1.3471 | 3.36E-01 | 3.97E-01 |
| sp\|P49718\|M | Mcm5 | MCM5_MOUS | 10 | 22.5354 | 28.3688 | 19.9481 | 10.5104 | 10.2869 | 8.3504 | 23.6174 | 4.3134 | 9.7159 | 1.1878 | 0.4114 | 5.76E-03 | 1.37E-02 |
| sp\|P26041\|M | Msn | MOES_MOUSE | 10 | 10.5379 | 10.3771 | 9.83596 | 30.246 | 23.6503 | 15.3528 | 10.2503 | 0.3677 | 23.0830 | 7.4628 | 2.2519 | 4.10E-02 | 6.20E-02 |
| tr\|E9QPE7\|E9 | Myh11 | E9QPE7_MOU | 10 | 3.12065 | 2.01497 | 1.94019 | 30.7512 | 45.5482 | 16.6247 | 2.3586 | 0.6610 | 30.9747 | 14.4630 | 13.1326 | 2.67E-02 | 4.35E-02 |
| tr\|Q921K2\|Q9 | Parp1 | Q921K2_MOU | 10 | 16.6364 | 19.3142 | 17.2251 | 15.0218 | 16.1762 | 15.6263 | 17.7252 | 1.4072 | 15.6081 | 0.5774 | 0.8806 | 7.35E-02 | 1.03E-01 |
| sp\|P35486\|OI | Pdha1 | ODPA_MOUSE | 10 | 18.2831 | 33.2104 | 15.1335 | 10.3701 | 9.17162 | 13.8314 | 22.2090 | 9.6568 | 11.1244 | 2.4197 | 0.5009 | 1.26E-01 | 1.67E-01 |

| | | | | | | | | | | | | | | | | | |
|---|---|---|---|---|---|---|---|---|---|---|---|---|---|---|---|---|---|
| sp\|P67778\|PH | Phb | PHB_MOUSE F | 10 | 22.5536 | 26.1491 | 22.4015 | 11.3483 | 7.57613 | 9.97143 | 23.7014 | 2.1211 | 9.6320 | 1.9089 | 0.4064 | 1.03E-03 | 4.76E-03 |
| sp\|Q99K85\|SE | Psat1 | SERC_MOUSE | 10 | 27.5994 | 21.8855 | 32.9902 | 7.58613 | 5.18608 | 4.75272 | 27.4917 | 5.5531 | 5.8416 | 1.5262 | 0.2125 | 2.87E-03 | 8.25E-03 |
| sp\|Q8VDM4\|F | Psmd2 | PSMD2_MOUS | 10 | 24.9623 | 27.6579 | 24.5095 | 9.61479 | 6.7798 | 6.47568 | 25.7099 | 1.7021 | 7.6234 | 1.7313 | 0.2965 | 2.08E-04 | 2.11E-03 |
| sp\|P14869\|RL | Rplp0 | RLA0_MOUSE | 10 | 19.3183 | 21.8791 | 16.7379 | 15.3629 | 16.675 | 10.0267 | 19.3118 | 2.5706 | 14.0215 | 3.5213 | 0.7261 | 1.03E-01 | 1.40E-01 |
| sp\|P62281\|RS | Rps11 | RS11_MOUSE | 10 | 17.1518 | 20.913 | 14.5669 | 16.7208 | 19.5825 | 11.0649 | 17.5439 | 3.1912 | 15.7894 | 4.3345 | 0.9000 | 6.03E-01 | 6.43E-01 |
| sp\|P19324\|SE | Serpinh1 | SERPH_MOUS | 10 | 1.2814 | 1.06244 | 0.568101 | 39.5509 | 40.7848 | 16.7524 | 0.9706 | 0.3654 | 32.3627 | 13.5330 | 33.3414 | 1.59E-02 | 2.88E-02 |
| sp\|P58771-2\|T | Tpm1 | TPM1_MOUSE | 10 | 6.25048 | 2.00575 | 4.78161 | 29.7193 | 40.6182 | 16.6246 | 4.3459 | 2.1556 | 28.9874 | 12.0135 | 6.6700 | 2.50E-02 | 4.14E-02 |
| sp\|Q9CQN1\|T | Trap1 | TRAP1_MOUS | 10 | 22.2196 | 22.7817 | 23.8857 | 10.9938 | 10.7274 | 9.39189 | 22.9623 | 0.8476 | 10.3710 | 0.8584 | 0.4517 | 5.50E-05 | 1.38E-03 |
| sp\|P63101\|14 | Ywhaz | 1433Z_MOUSE | 10 | 25.0854 | 21.9209 | 29.8252 | 9.80966 | 6.26184 | 7.09699 | 25.6105 | 3.9782 | 7.7228 | 1.8549 | 0.3015 | 2.12E-03 | 6.83E-03 |
| sp\|P50247\|SA | Ahcy | SAHH_MOUS | 9 | 22.6173 | 18.2081 | 26.0353 | 11.8784 | 8.65584 | 12.6052 | 22.2869 | 3.9240 | 11.0465 | 2.1020 | 0.4956 | 1.19E-02 | 2.29E-02 |
| sp\|P48036\|AN | Anxa5 | ANXA5_MOUS | 9 | 12.7013 | 13.4059 | 14.6374 | 26.399 | 18.3851 | 14.4713 | 13.5815 | 0.9799 | 19.7518 | 6.0802 | 1.4543 | 1.58E-01 | 2.02E-01 |
| sp\|Q9D8L5\|C | Ccdc91 | CCD91_MOUS | 9 | 21.653 | 30.7793 | 12.6064 | 12.2408 | 9.80682 | 12.9136 | 21.6796 | 9.0865 | 11.6537 | 1.6345 | 0.5375 | 1.33E-01 | 1.75E-01 |
| sp\|P11087\|CC | Col1a1 | CO1A1_MOUS | 9 | 1.9416 | 0.821149 | 1.0585 | 40.7465 | 40.1069 | 15.3254 | 1.2737 | 0.5904 | 32.0596 | 14.4958 | 25.1695 | 2.13E-02 | 3.65E-02 |
| sp\|Q9JIF7\|CO | Copb1 | COPB_MOUSE | 9 | 19.8182 | 23.5462 | 21.8794 | 13.6222 | 11.3205 | 9.81345 | 21.7479 | 1.8675 | 11.5854 | 1.9181 | 0.5327 | 2.77E-03 | 8.10E-03 |
| sp\|Q60598\|SR | Cttn | SRC8_MOUSE | 9 | 24.1828 | 14.3184 | 17.0632 | 17.5639 | 15.3972 | 11.4745 | 18.5215 | 5.0913 | 14.8119 | 3.0866 | 0.7997 | 3.41E-01 | 4.01E-01 |
| sp\|Q8JZQ9\|EI | Eif3b | EIF3B_MOUSE | 9 | 21.962 | 24.2982 | 24.6751 | 9.47439 | 10.295 | 9.2953 | 23.6451 | 1.4697 | 9.6882 | 0.5331 | 0.4097 | 1.02E-04 | 1.63E-03 |
| sp\|Q9WUA2\|S | Farsb | SYFB_MOUSE | 9 | 24.256 | 25.1602 | 28.1271 | 9.33366 | 7.06191 | 6.06112 | 25.8478 | 2.0251 | 7.4856 | 1.6769 | 0.2896 | 2.68E-04 | 2.41E-03 |
| sp\|Q91WJ8\|FU | Fubp1 | FUBP1_MOUS | 9 | 36.7197 | 22.5069 | 25.2191 | 6.05405 | 4.58074 | 4.91942 | 28.1486 | 7.5457 | 5.1847 | 0.7717 | 0.1842 | 6.32E-03 | 1.45E-02 |
| sp\|P13020\|GE | Gsn | GELS_MOUSE | 9 | 9.77077 | 11.7899 | 12.382 | 26.9242 | 21.0958 | 18.0374 | 11.3142 | 1.3691 | 22.0191 | 4.5148 | 1.9461 | 1.71E-02 | 3.06E-02 |
| sp\|P15864\|H1 | Hist1h1c | H12_MOUSE H | 9 | 7.01918 | 10.9357 | 3.92355 | 35.824 | 25.9599 | 16.3375 | 7.2928 | 3.5141 | 26.0405 | 9.7435 | 3.5707 | 3.50E-02 | 5.43E-02 |
| sp\|P48722\|HS | Hspa4l | HS74L_MOUS | 9 | 26.1049 | 28.7154 | 29.3828 | 5.04381 | 4.56175 | 6.19127 | 28.0677 | 1.7323 | 5.2656 | 0.8371 | 0.1876 | 3.33E-05 | 1.17E-03 |
| sp\|P97311\|M | Mcm6 | MCM6_MOUS | 9 | 18.9726 | 19.8173 | 20.3213 | 13.4696 | 18.4183 | 9.00088 | 19.7037 | 0.6815 | 13.6296 | 4.7107 | 0.6917 | 9.16E-02 | 1.26E-01 |
| sp\|Q99K48\|N | Nono | NONO_MOUS | 9 | 12.1628 | 12.359 | 10.7406 | 22.6413 | 31.4762 | 10.62 | 11.7541 | 0.8832 | 21.5792 | 10.4686 | 1.8359 | 1.81E-01 | 2.27E-01 |
| sp\|Q60597\|O | Ogdh | ODO1_MOUSE | 9 | 24.1347 | 26.7687 | 23.1937 | 9.54818 | 8.57468 | 7.7801 | 24.6990 | 1.8531 | 8.6343 | 0.8855 | 0.3496 | 1.72E-04 | 1.97E-03 |
| sp\|P50580\|PA | Pa2g4 | PA2G4_MOUS | 9 | 25.6509 | 22.2267 | 26.979 | 10.6011 | 8.33246 | 6.20994 | 24.9522 | 2.4520 | 8.3812 | 2.1960 | 0.3359 | 9.53E-04 | 4.62E-03 |
| sp\|P57722\|PC | Pcbp3 | PCBP3_MOUS | 9 | 22.6884 | 21.4248 | 23.2234 | 7.29328 | 6.28962 | 19.0805 | 22.4455 | 0.9236 | 10.8878 | 7.1128 | 0.4851 | 4.93E-02 | 7.28E-02 |
| sp\|Q9D051\|O | Pdhb | ODPB_MOUSE | 9 | 19.0019 | 32.2733 | 14.4536 | 10.143 | 10.4061 | 13.7222 | 21.9096 | 9.2589 | 11.4238 | 1.9948 | 0.5214 | 1.28E-01 | 1.69E-01 |
| sp\|Q922R8\|PD | Pdia6 | PDIA6_MOUS | 9 | 20.9895 | 20.036 | 23.6838 | 15.7815 | 11.3509 | 8.15839 | 21.5698 | 1.8919 | 11.7636 | 3.8283 | 0.5454 | 1.64E-02 | 2.94E-02 |
| sp\|Q9DCD0\|6 | Pgd | 6PGD_MOUSE | 9 | 25.5677 | 22.1344 | 28.634 | 10.5084 | 6.6907 | 6.46476 | 25.4454 | 3.2515 | 7.8880 | 2.2722 | 0.3100 | 1.56E-03 | 5.79E-03 |
| sp\|O08709\|PR | Prdx6 | PRDX6_MOUS | 9 | 24.1107 | 19.8249 | 26.2288 | 10.0073 | 7.51177 | 12.3165 | 23.3881 | 3.2625 | 9.9452 | 2.4030 | 0.4252 | 4.55E-03 | 1.15E-02 |
| sp\|Q8CIG8\|AN | Prmt5 | ANM5_MOUS | 9 | 21.4865 | 32.2791 | 20.9147 | 9.18506 | 7.47908 | 8.6555 | 24.8934 | 6.4026 | 8.4399 | 0.8732 | 0.3390 | 1.16E-02 | 2.24E-02 |
| sp\|P14685\|PS | Psmd3 | PSMD3_MOUS | 9 | 22.9925 | 26.5726 | 24.4257 | 10.4854 | 7.50053 | 8.02323 | 24.6636 | 1.8019 | 8.6697 | 1.5940 | 0.3515 | 3.25E-04 | 2.60E-03 |
| sp\|Q8BK67\|R | Rcc2 | RCC2_MOUSE | 9 | 28.2997 | 30.6553 | 27.529 | 4.53004 | 4.64192 | 4.34408 | 28.8280 | 1.6287 | 4.5053 | 0.1504 | 0.1563 | 1.35E-05 | 9.60E-04 |
| sp\|P47962\|RL | Rpl5 | RL5_MOUSE 6 | 9 | 22.5938 | 22.7659 | 23.4056 | 11.7325 | 10.9998 | 8.50244 | 22.9218 | 0.4278 | 10.4116 | 1.6935 | 0.4542 | 2.43E-04 | 2.29E-03 |
| sp\|P12970\|RL | Rpl7a | RL7A_MOUSE | 9 | 15.5388 | 20.1153 | 12.0765 | 19.4938 | 22.5579 | 10.2177 | 15.9102 | 4.0322 | 17.4231 | 6.4254 | 1.0951 | 7.47E-01 | 7.77E-01 |
| sp\|P62918\|RL | Rpl8 | RL8_MOUSE 6 | 9 | 17.3669 | 23.6369 | 14.1965 | 16.4549 | 18.5088 | 9.83598 | 18.4001 | 4.8043 | 14.9332 | 4.5322 | 0.8116 | 4.15E-01 | 4.68E-01 |
| sp\|P62301\|RS | Rps13 | RS13_MOUSE | 9 | 14.0108 | 19.8436 | 11.2457 | 20.3368 | 21.2166 | 13.3465 | 15.0334 | 4.3892 | 18.3000 | 4.3123 | 1.2173 | 4.10E-01 | 4.64E-01 |
| sp\|Q9CZX8\|RS | Rps19 | RS19_MOUSE | 9 | 20.9157 | 22.2289 | 12.1856 | 19.2988 | 16.4177 | 8.95322 | 18.4434 | 5.4590 | 14.8899 | 5.3393 | 0.8073 | 4.65E-01 | 5.16E-01 |
| sp\|P25444\|RS | Rps2 | RS2_MOUSE 4 | 9 | 17.0725 | 21.2052 | 13.7519 | 17.3904 | 19.2944 | 11.2855 | 17.3432 | 3.7340 | 15.9901 | 4.1840 | 0.9220 | 6.97E-01 | 7.32E-01 |
| sp\|P62702\|RS | Rps4x | RS4X_MOUSE | 9 | 16.428 | 19.529 | 11.7514 | 19.3168 | 21.8597 | 11.1151 | 15.9028 | 3.9153 | 17.4305 | 5.6152 | 1.0961 | 7.19E-01 | 7.52E-01 |
| sp\|Q921M3\|S | Sf3b3 | SF3B3_MOUS | 9 | 21.9154 | 24.9511 | 20.4228 | 13.2543 | 10.2972 | 9.15903 | 22.4298 | 2.3076 | 10.9035 | 2.1139 | 0.4861 | 3.10E-03 | 8.77E-03 |
| sp\|Q9CW03\|S | Smc3 | SMC3_MOUSE | 9 | 20.5456 | 23.3689 | 22.8302 | 7.69468 | 9.91005 | 15.6506 | 22.2482 | 1.4989 | 11.0851 | 4.1061 | 0.4982 | 1.15E-02 | 2.24E-02 |
| sp\|Q99L47\|F1 | St13 | F10A1_MOUS | 9 | 31.8163 | 23.201 | 24.4315 | 8.16646 | 6.178 | 6.20665 | 26.4829 | 4.6596 | 6.8504 | 1.1399 | 0.2587 | 2.09E-03 | 6.77E-03 |
| sp\|P17751\|TP | Tpi1 | TPIS_MOUSE T | 9 | 23.6944 | 22.0269 | 25.9266 | 10.6415 | 8.18405 | 9.52648 | 23.8826 | 1.9567 | 9.4507 | 1.2305 | 0.3957 | 4.15E-04 | 2.95E-03 |
| sp\|F6ZDS4\|TP | Tpr | TPR_MOUSE N | 9 | 27.0976 | 26.7686 | 15.6876 | 12.424 | 9.44264 | 8.57963 | 23.1846 | 6.4947 | 10.1488 | 2.0171 | 0.4377 | 2.94E-02 | 4.71E-02 |
| sp\|Q62465\|V | Vat1 | VAT1_MOUSE | 9 | 21.6361 | 21.7377 | 25.0102 | 13.2799 | 10.5913 | 7.7448 | 22.7947 | 1.9194 | 10.5387 | 2.7679 | 0.4623 | 3.24E-03 | 9.06E-03 |
| sp\|O88342\|W | Wdr1 | WDR1_MOUS | 9 | 17.3884 | 16.5623 | 18.1764 | 19.604 | 16.1422 | 12.1267 | 17.3757 | 0.8071 | 15.9576 | 3.7421 | 0.9184 | 5.56E-01 | 5.98E-01 |
| sp\|Q80UG5\|S | 9-Sep | SEPT9_MOUSE | 8 | 23.0446 | 25.6769 | 21.9347 | 10.6441 | 8.13897 | 10.5608 | 23.5521 | 1.9220 | 9.7813 | 1.4229 | 0.4153 | 5.68E-04 | 3.52E-03 |
| sp\|P07356\|AN | Anxa2 | ANXA2_MOUS | 8 | 8.20701 | 7.92184 | 10.7793 | 30.5157 | 23.3495 | 19.2266 | 8.9694 | 1.5739 | 24.3639 | 5.7125 | 2.7163 | 1.08E-02 | 2.14E-02 |
| sp\|P17426\|AP | Ap2a1 | AP2A1_MOUS | 8 | 18.8312 | 19.2021 | 16.917 | 16.7075 | 16.8244 | 11.5177 | 18.3168 | 1.2263 | 15.0165 | 3.0306 | 0.8198 | 1.55E-01 | 1.99E-01 |
| sp\|Q9JLV1\|BA | Bag3 | BAG3_MOUSE | 8 | 30.6412 | 30.9126 | 20.2336 | 7.10863 | 5.55024 | 5.55379 | 27.2625 | 6.0887 | 6.0709 | 0.8987 | 0.2227 | 3.97E-03 | 1.03E-02 |
| sp\|P97427\|DP | Crmp1 | DPYL1_MOUS | 8 | 27.1446 | 34.9897 | 24.8946 | 4.38244 | 4.29025 | 4.29842 | 29.0096 | 5.2997 | 4.3237 | 0.0510 | 0.1490 | 1.28E-03 | 5.27E-03 |
| sp\|O08788\|D | Dctn1 | DCTN1_MOUS | 8 | 22.2205 | 24.8459 | 23.1537 | 10.23 | 9.41617 | 10.1336 | 23.4067 | 1.3309 | 9.9266 | 0.4447 | 0.4241 | 7.64E-05 | 1.54E-03 |
| sp\|Q9JIK5\|DD | Ddx21 | DDX21_MOUS | 8 | 26.8486 | 24.3502 | 20.5299 | 10.1006 | 10.8268 | 7.34383 | 23.9096 | 3.1823 | 9.4237 | 1.8375 | 0.3941 | 2.41E-03 | 7.35E-03 |
| sp\|Q8VDW0\|D | Ddx39a | DX39A_MOUS | 8 | 25.7199 | 21.8799 | 28.2112 | 9.94044 | 7.08299 | 7.16558 | 25.2703 | 3.1895 | 8.0630 | 1.6264 | 0.3191 | 1.14E-03 | 4.97E-03 |
| sp\|P57776-3\|E | Eef1d | EF1D_MOUSE | 8 | 24.0396 | 30.1007 | 20.6762 | 10.4816 | 7.4811 | 7.22089 | 24.9388 | 4.7762 | 8.3945 | 1.8121 | 0.3366 | 4.96E-03 | 1.22E-02 |
| sp\|Q8BGD9\|IF | Eif4b | IF4B_MOUSE E | 8 | 28.7948 | 23.5581 | 19.7886 | 10.8782 | 8.31977 | 8.66049 | 24.0472 | 4.5230 | 9.2862 | 1.3892 | 0.3862 | 5.68E-03 | 1.36E-02 |
| sp\|Q61553\|FS | Fscn1 | FSCN1_MOUS | 8 | 20.6502 | 15.436 | 25.9523 | 14.0491 | 14.0447 | 9.86768 | 20.6795 | 5.2582 | 12.6538 | 2.4129 | 0.6119 | 7.41E-02 | 1.04E-01 |
| tr\|E9PZF0\|E9P | Gm20390 | E9PZF0_MOUS | 8 | 21.7139 | 16.4449 | 23.3456 | 16.003 | 10.5981 | 11.8945 | 20.5015 | 3.6066 | 12.8319 | 2.8217 | 0.6259 | 4.41E-02 | 6.61E-02 |
| sp\|P28798\|GR | Grn | GRN_MOUSE | 8 | 28.7844 | 27.7132 | 14.7336 | 11.809 | 7.37503 | 9.58478 | 23.7437 | 7.8214 | 9.5896 | 2.2170 | 0.4039 | 3.93E-02 | 5.98E-02 |
| sp\|P10853\|H2 | Hist1h2bf | H2B1F_MOUS | 8 | 5.51453 | 5.53879 | 3.68181 | 32.8591 | 29.8375 | 22.5683 | 4.9117 | 1.0652 | 28.4216 | 5.2895 | 5.7865 | 1.65E-03 | 5.94E-03 |
| sp\|P14152\|M | Mdh1 | MDHC_MOUS | 8 | 24.4494 | 19.6033 | 31.0218 | 8.79957 | 6.60673 | 9.51928 | 25.0248 | 5.7310 | 8.3085 | 1.5171 | 0.3320 | 8.14E-03 | 1.75E-02 |
| sp\|P18155\|M | Mthfd2 | MTDC_MOUSE | 8 | 30.1757 | 30.4164 | 28.2111 | 3.62185 | 3.53821 | 4.03668 | 29.6011 | 1.2097 | 3.7322 | 0.2669 | 0.1261 | 3.49E-06 | 7.86E-04 |

| | | | | | | | | | | | | | | | | |
|---|---|---|---|---|---|---|---|---|---|---|---|---|---|---|---|---|
| sp\|Q8BP47\|SY | Nars | SYNC_MOUSE | 8 | 23.4133 | 24.3944 | 26.2695 | 9.22472 | 7.83028 | 8.86779 | 24.6924 | 1.4512 | 8.6409 | 0.7244 | 0.3499 | 6.80E-05 | 1.49E-03 |
| sp\|Q61937\|NI | Npm1 | NPM_MOUSE | 8 | 31.4837 | 23.2991 | 20.3827 | 9.9245 | 8.12118 | 6.78891 | 25.0552 | 5.7551 | 8.2782 | 1.5737 | 0.3304 | 8.22E-03 | 1.76E-02 |
| sp\|P29758\|OA | Oat | OAT_MOUSE ( | 8 | 24.1021 | 22.9695 | 24.8636 | 9.48062 | 8.88961 | 9.69461 | 23.9784 | 0.9531 | 9.3549 | 0.4170 | 0.3901 | 1.69E-05 | 1.03E-03 |
| sp\|Q9DBC7\|K/ | Prkar1a | KAP0_MOUSE | 8 | 31.6641 | 24.7728 | 32.1194 | 3.79882 | 3.06088 | 4.58398 | 29.5188 | 4.1164 | 3.8146 | 0.7617 | 0.1292 | 4.43E-04 | 3.07E-03 |
| sp\|Q9JIF0\|ANI | Prmt1 | ANM1_MOUSI | 8 | 29.1326 | 31.7278 | 24.0276 | 4.73236 | 4.46967 | 5.90999 | 28.2960 | 3.9177 | 5.0373 | 0.7671 | 0.1780 | 5.43E-04 | 3.44E-03 |
| sp\|Q99PV0\|PI | Prpf8 | PRP8_MOUSE | 8 | 19.1819 | 24.3217 | 15.768 | 15.3082 | 15.7485 | 9.67182 | 19.7572 | 4.3058 | 13.5762 | 3.3884 | 0.6872 | 1.22E-01 | 1.62E-01 |
| sp\|Q3TXS7\|PS | Psmd1 | PSMD1_MOUS | 8 | 22.943 | 25.9823 | 21.647 | 11.8339 | 9.32114 | 8.27273 | 23.5241 | 2.2253 | 9.8093 | 1.8301 | 0.4170 | 1.18E-03 | 5.07E-03 |
| sp\|Q9D8E6\|RI | Rpl4 | RL4_MOUSE 6 | 8 | 15.9601 | 18.6017 | 12.8039 | 18.8834 | 22.1507 | 11.6003 | 15.7886 | 2.9027 | 17.5448 | 5.4011 | 1.1112 | 6.46E-01 | 6.85E-01 |
| sp\|Q8VEM8\|N | Slc25a3 | MPCP_MOUSE | 8 | 23.1726 | 25.5564 | 21.4735 | 12.8683 | 8.7259 | 8.20329 | 23.4008 | 2.0510 | 9.9325 | 2.5559 | 0.4245 | 2.06E-03 | 6.72E-03 |
| sp\|Q6P4T2\|U! | Snrnp200 | U520_MOUSE | 8 | 15.3105 | 21.7815 | 13.0319 | 18.2242 | 22.4055 | 9.24638 | 16.7080 | 4.5391 | 16.6254 | 6.7237 | 0.9951 | 9.87E-01 | 9.88E-01 |
| sp\|Q62351\|TF | Tfrc | TFR1_MOUSE | 8 | 27.4707 | 28.5748 | 27.2112 | 5.4322 | 5.23012 | 6.08089 | 27.7522 | 0.7241 | 5.5811 | 0.4445 | 0.2011 | 1.43E-06 | 5.68E-04 |
| sp\|Q60932\|VI | Vdac1 | VDAC1_MOUS | 8 | 24.3661 | 22.4488 | 26.0898 | 11.5819 | 8.44782 | 7.06558 | 24.3016 | 1.8214 | 9.0318 | 2.3141 | 0.3717 | 8.51E-04 | 4.41E-03 |
| sp\|Q60930\|VI | Vdac2 | VDAC2_MOUS | 8 | 20.9002 | 20.548 | 22.1908 | 14.7915 | 11.5832 | 9.9864 | 21.2130 | 0.8649 | 12.1204 | 2.4472 | 0.5714 | 3.73E-03 | 9.86E-03 |
| sp\|Q5XJY5\|CO | Arcn1 | COPD_MOUSE | 7 | 18.2612 | 18.5879 | 21.1627 | 17.3819 | 14.0748 | 10.5314 | 19.3373 | 1.5893 | 13.9960 | 3.4259 | 0.7238 | 7.05E-02 | 9.99E-02 |
| sp\|Q9CXW3\|C | Cacybp | CYBP_MOUSE | 7 | 32.0282 | 25.0905 | 27.8298 | 5.75376 | 4.93751 | 4.3602 | 28.3162 | 3.4943 | 5.0172 | 0.7002 | 0.1772 | 3.47E-04 | 2.70E-03 |
| sp\|P18760\|CC | Cfl1 | COF1_MOUSE | 7 | 19.8559 | 18.33 | 15.8252 | 20.7417 | 14.1541 | 11.093 | 18.0037 | 2.0351 | 15.3296 | 4.9306 | 0.8515 | 4.34E-01 | 4.86E-01 |
| sp\|O54734\|OS | Ddost | OST48_MOUSI | 7 | 21.1559 | 25.0661 | 22.4753 | 12.5875 | 10.1802 | 8.53503 | 22.8991 | 1.9893 | 10.4342 | 2.0381 | 0.4557 | 1.62E-03 | 5.92E-03 |
| sp\|Q9Z2W0\|D | Dnpep | DNPEP_MOUS | 7 | 21.7891 | 24.3535 | 25.4282 | 13.2386 | 8.12754 | 7.06303 | 23.8569 | 1.8697 | 9.4764 | 3.3014 | 0.3972 | 2.79E-03 | 8.11E-03 |
| sp\|Q9Z0N1\|IF | Eif2s3x | IF2G_MOUSE I | 7 | 19.1892 | 21.0603 | 16.3889 | 14.1117 | 17.9276 | 11.3222 | 18.8795 | 2.3511 | 14.4538 | 3.3160 | 0.7656 | 1.32E-01 | 1.74E-01 |
| sp\|P60229\|EIF | Eif3e | EIF3E_MOUSE | 7 | 22.5226 | 27.0545 | 21.0967 | 10.5907 | 9.23734 | 9.49806 | 23.5579 | 3.1109 | 9.7754 | 0.7180 | 0.4150 | 1.71E-03 | 5.98E-03 |
| sp\|P70372\|EL/ | Elavl1 | ELAV1_MOUSI | 7 | 16.2186 | 20.2189 | 13.6882 | 18.2599 | 17.5545 | 14.0599 | 16.7086 | 3.2928 | 16.6248 | 2.2491 | 0.9950 | 9.73E-01 | 9.78E-01 |
| sp\|Q8C0C7\|SY | Farsa | SYFA_MOUSE | 7 | 24.3994 | 31.8417 | 22.7279 | 8.15649 | 7.09852 | 5.77598 | 26.3230 | 4.8519 | 7.0103 | 1.1927 | 0.2663 | 2.59E-03 | 7.74E-03 |
| sp\|Q920E5\|FP | Fdps | FPPS_MOUSE | 7 | 29.0177 | 24.8809 | 30.5143 | 5.129 | 4.35563 | 6.10248 | 28.1376 | 2.9180 | 5.1957 | 0.8753 | 0.1847 | 1.99E-04 | 2.09E-03 |
| sp\|P30416\|FK | Fkbp4 | FKBP4_MOUSI | 7 | 29.0503 | 23.4447 | 32.555 | 5.59871 | 4.29036 | 5.06094 | 28.3500 | 4.5953 | 4.9833 | 0.6576 | 0.1758 | 9.53E-04 | 4.62E-03 |
| sp\|D3Z7P3\|GL | Gls | GLSK_MOUSE | 7 | 23.4922 | 23.6115 | 27.0918 | 10.2166 | 8.44327 | 7.14457 | 24.7318 | 2.0447 | 8.6015 | 1.5421 | 0.3478 | 4.01E-04 | 2.89E-03 |
| sp\|Q3THK7\|GI | Gmps | GUAA_MOUSE | 7 | 26.1972 | 31.8168 | 26.9937 | 4.89463 | 3.86779 | 6.22983 | 28.3359 | 3.0407 | 4.9974 | 1.1844 | 0.1764 | 2.44E-04 | 2.29E-03 |
| sp\|P54071\|IDI | Idh2 | IDHP_MOUSE | 7 | 22.1143 | 21.8905 | 28.8865 | 10.9194 | 8.22762 | 7.96156 | 24.2971 | 3.9761 | 9.0362 | 1.6363 | 0.3719 | 3.55E-03 | 9.63E-03 |
| sp\|Q8BKC5\|IP | Ipo5 | IPO5_MOUSE I | 7 | 24.7154 | 28.3351 | 28.7696 | 7.08701 | 5.22072 | 5.87216 | 27.2734 | 2.2259 | 6.0600 | 0.9472 | 0.2222 | 1.10E-04 | 1.64E-03 |
| sp\|Q505F5\|LR | Lrrc47 | LRC47_MOUSI | 7 | 16.7643 | 16.2301 | 18.4695 | 15.5066 | 21.2632 | 11.7662 | 17.1546 | 1.1696 | 16.1787 | 4.7840 | 0.9431 | 7.49E-01 | 7.78E-01 |
| sp\|Q8K310\|M | Matr3 | MATR3_MOUS | 7 | 15.2839 | 20.7753 | 12.5249 | 19.9499 | 18.4344 | 13.0315 | 16.1947 | 4.1999 | 17.1386 | 3.6367 | 1.0583 | 7.83E-01 | 8.11E-01 |
| sp\|P46460\|NS | Nsf | NSF_MOUSE V | 7 | 26.2594 | 26.1437 | 27.1003 | 8.15106 | 5.01017 | 7.3354 | 26.5011 | 0.5221 | 6.8322 | 1.6298 | 0.2578 | 3.76E-05 | 1.17E-03 |
| sp\|Q1HFZ0\|NS | Nsun2 | NSUN2_MOUS | 7 | 24.0106 | 29.0181 | 29.0413 | 6.56684 | 5.09645 | 6.26665 | 27.3567 | 2.8978 | 5.9766 | 0.7769 | 0.2185 | 2.48E-04 | 2.29E-03 |
| sp\|Q99LX0\|PA | Park7 | PARK7_MOUS | 7 | 28.7068 | 22.506 | 25.78 | 8.85875 | 5.70694 | 8.4416 | 25.6643 | 3.1020 | 7.6691 | 1.7120 | 0.2988 | 9.21E-04 | 4.58E-03 |
| sp\|Q8BH04\|P( | Pck2 | PCKGM_MOUS | 7 | 14.3173 | 10.9436 | 15.3134 | 24.3401 | 17.9673 | 17.1184 | 13.5248 | 2.2902 | 19.8086 | 3.9473 | 1.4646 | 7.56E-02 | 1.06E-01 |
| sp\|P62962\|PR | Pfn1 | PROF1_MOUS | 7 | 17.3206 | 15.8403 | 21.1122 | 20.7719 | 13.8936 | 11.0613 | 18.0910 | 2.7191 | 15.2423 | 4.9938 | 0.8425 | 4.34E-01 | 4.86E-01 |
| sp\|O35129\|PH | Phb2 | PHB2_MOUSE | 7 | 21.5399 | 28.9225 | 21.4213 | 12.1913 | 7.71264 | 8.21234 | 23.9612 | 4.2970 | 9.3721 | 2.4543 | 0.3911 | 6.95E-03 | 1.55E-02 |
| sp\|P37040\|NC | Por | NCPR_MOUSE | 7 | 22.9989 | 19.3821 | 29.3392 | 10.5774 | 8.96481 | 8.73759 | 23.9067 | 5.0402 | 9.4266 | 1.0031 | 0.3943 | 8.16E-03 | 1.75E-02 |
| sp\|O08807\|PR | Prdx4 | PRDX4_MOUS | 7 | 22.9691 | 21.7025 | 21.4558 | 13.4628 | 8.8178 | 11.592 | 22.0425 | 0.8119 | 11.2909 | 2.3371 | 0.5122 | 1.67E-03 | 5.94E-03 |
| sp\|Q9R1P4\|PS | Psma1 | PSA1_MOUSE | 7 | 21.5328 | 25.5836 | 22.8751 | 10.4552 | 8.70227 | 10.8511 | 23.3305 | 2.0634 | 10.0029 | 1.1436 | 0.4287 | 6.11E-04 | 3.64E-03 |
| sp\|O70435\|PS | Psma3 | PSA3_MOUSE | 7 | 18.757 | 20.8383 | 17.9955 | 19.1651 | 12.6806 | 10.5636 | 19.1969 | 1.4716 | 14.1364 | 4.4817 | 0.7364 | 1.37E-01 | 1.78E-01 |
| sp\|P46471\|PR | Psmc2 | PRS7_MOUSE | 7 | 26.3116 | 22.636 | 26.6519 | 9.22396 | 7.86629 | 7.31021 | 25.1998 | 2.2269 | 8.1335 | 0.9845 | 0.3228 | 2.64E-04 | 2.40E-03 |
| sp\|Q8BG32\|PS | Psmd11 | PSD11_MOUSI | 7 | 23.4667 | 27.5883 | 23.3207 | 10.3069 | 7.85338 | 7.46404 | 24.7919 | 2.4229 | 8.5414 | 1.5413 | 0.3445 | 6.07E-04 | 3.63E-03 |
| tr\|Q5XJF6\|Q5 | Rpl10a | Q5XJF6_MOUS | 7 | 14.6207 | 19.6501 | 11.2723 | 20.8779 | 22.807 | 10.772 | 15.1810 | 4.2169 | 18.1523 | 6.4639 | 1.1957 | 5.41E-01 | 5.84E-01 |
| sp\|P27659\|RI | Rpl3 | RL3_MOUSE 6 | 7 | 14.9098 | 20.0282 | 11.999 | 19.2394 | 23.3641 | 10.4595 | 15.6457 | 4.0649 | 17.6877 | 6.5908 | 1.1305 | 6.72E-01 | 7.09E-01 |
| sp\|Q6ZWN5\|R | Rps9 | RS9_MOUSE 4 | 7 | 18.6586 | 17.9531 | 15.9637 | 15.9184 | 19.4108 | 12.0953 | 17.5251 | 1.3975 | 15.8082 | 3.6590 | 0.9020 | 4.90E-01 | 5.39E-01 |
| sp\|Q99P72\|RT | Rtn4 | RTN4_MOUSE | 7 | 22.8113 | 17.9715 | 22.9725 | 16.6325 | 9.75146 | 9.86064 | 21.2518 | 2.8419 | 12.0815 | 3.9416 | 0.5685 | 3.08E-02 | 4.91E-02 |
| sp\|P26638\|SY | Sars | SYSC_MOUSE | 7 | 28.0895 | 23.6545 | 32.6047 | 5.96674 | 4.70438 | 4.98016 | 28.1162 | 4.4752 | 5.2171 | 0.6637 | 0.1856 | 9.33E-04 | 4.58E-03 |
| sp\|Q3UPL0\|SC | Sec31a | SC31A_MOUSI | 7 | 19.0518 | 18.1853 | 19.457 | 18.3315 | 13.3307 | 11.6437 | 18.8980 | 0.6496 | 14.4353 | 3.4780 | 0.7639 | 9.43E-02 | 1.29E-01 |
| sp\|Q8K4Z5\|SF | Sf3a1 | SF3A1_MOUSI | 7 | 18.5379 | 21.6463 | 17.5696 | 16.2635 | 12.7042 | 13.2785 | 19.2513 | 2.1299 | 14.0821 | 1.9109 | 0.7315 | 3.52E-02 | 5.45E-02 |
| tr\|G5E866\|G5 | Sf3b1 | G5E866_MOU | 7 | 21.313 | 27.2253 | 18.8706 | 13.2428 | 11.2234 | 8.12494 | 22.4696 | 4.2958 | 10.8637 | 2.5778 | 0.4835 | 1.60E-02 | 2.88E-02 |
| tr\|Q3UJB0\|Q3 | Sf3b2 | Q3UJB0_MOU | 7 | 26.5968 | 21.946 | 18.2403 | 14.2837 | 10.56 | 8.37326 | 22.2610 | 4.1871 | 11.0723 | 2.9883 | 0.4974 | 1.97E-02 | 3.44E-02 |
| sp\|P54227\|STI | Stmn1 | STMN1_MOUS | 7 | 30.0498 | 25.6257 | 25.7051 | 6.61827 | 5.33908 | 6.662 | 27.1269 | 2.5316 | 6.2065 | 0.7515 | 0.2288 | 1.63E-04 | 1.94E-03 |
| sp\|Q93092\|TA | Taldo1 | TALDO_MOUS | 7 | 23.1189 | 19.6944 | 26.7743 | 13.9264 | 8.14833 | 8.33767 | 23.1959 | 3.5406 | 10.1375 | 3.2827 | 0.4370 | 9.42E-03 | 1.92E-02 |
| sp\|Q61029\|LA | Tmpo | LAP2B_MOUSI | 7 | 18.1029 | 21.243 | 15.2571 | 15.6556 | 17.3922 | 12.3493 | 18.2010 | 2.9942 | 15.1324 | 2.5618 | 0.8314 | 2.49E-01 | 3.04E-01 |
| sp\|Q9CZW5\|T | Tomm70a | TOM70_MOUS | 7 | 24.976 | 25.0744 | 27.0856 | 9.37823 | 6.5783 | 6.90745 | 25.7120 | 1.1906 | 7.6213 | 1.5304 | 0.2964 | 8.58E-05 | 1.63E-03 |
| sp\|Q9R0P9\|U( | Uchl1 | UCHL1_MOUS | 7 | 29.9375 | 28.6816 | 30.9442 | 2.75837 | 3.0741 | 4.60423 | 29.8544 | 1.1336 | 3.4789 | 0.9873 | 0.1165 | 6.98E-06 | 8.58E-04 |
| sp\|Q60931\|VI | Vdac3 | VDAC3_MOUS | 7 | 26.5583 | 22.7761 | 30.0253 | 8.95772 | 4.60054 | 7.08209 | 26.4532 | 3.6257 | 6.8801 | 2.1856 | 0.2601 | 1.32E-03 | 5.35E-03 |
| sp\|Q9EQH3\|V | Vps35 | VPS35_MOUSI | 7 | 23.0679 | 18.4868 | 26.4732 | 11.6592 | 8.86809 | 11.4448 | 22.6760 | 4.0076 | 10.6574 | 1.5533 | 0.4700 | 8.38E-03 | 1.78E-02 |
| sp\|P32921\|SY | Wars | SYWC_MOUSE | 7 | 25.3613 | 24.1341 | 32.073 | 6.68045 | 4.87716 | 6.87396 | 27.1895 | 4.2735 | 6.1439 | 1.1013 | 0.2260 | 1.17E-03 | 5.07E-03 |
| sp\|P61222\|AB | Abce1 | ABCE1_MOUSI | 6 | 24.2426 | 25.8838 | 26.2041 | 8.98114 | 8.30047 | 6.3878 | 25.4435 | 1.0523 | 7.8898 | 1.3446 | 0.3101 | 5.84E-05 | 1.39E-03 |
| sp\|Q8QZT1\|TI | Acat1 | THIL_MOUSE / | 6 | 22.1414 | 20.2095 | 30.1002 | 11.1534 | 8.60446 | 7.79102 | 24.1504 | 5.2425 | 9.1830 | 1.7543 | 0.3802 | 9.38E-03 | 1.92E-02 |
| sp\|P61164\|AC | Actr1a | ACTZ_MOUSE | 6 | 20.5783 | 30.7748 | 20.3258 | 11.3332 | 8.21421 | 8.77369 | 23.8930 | 5.9612 | 9.4404 | 1.6629 | 0.3951 | 1.55E-02 | 2.82E-02 |
| sp\|P61161\|AR | Actr2 | ARP2_MOUSE | 6 | 20.0268 | 17.4301 | 21.541 | 15.995 | 13.4173 | 11.5898 | 19.6660 | 2.0791 | 13.6674 | 2.2132 | 0.6950 | 2.67E-02 | 4.35E-02 |

| | | | | | | | | | | | | | | | | |
|---|---|---|---|---|---|---|---|---|---|---|---|---|---|---|---|---|
| sp\|P45376\|AL | Akr1b1 | ALDR_MOUSE | 6 | 21.0495 | 22.4545 | 24.4726 | 10.7977 | 10.9635 | 10.2622 | 22.6589 | 1.7207 | 10.6745 | 0.3665 | 0.4711 | 2.95E-04 | 2.48E-03 |
| sp\|P61205\|AR | Arf3 | ARF3_MOUSE | 6 | 23.3566 | 18.1503 | 27.4206 | 10.3707 | 9.43794 | 11.2639 | 22.9758 | 4.6469 | 10.3575 | 0.9131 | 0.4508 | 9.92E-03 | 2.00E-02 |
| sp\|Q99PT1\|GI | Arhgdia | GDIR1_MOUSI | 6 | 16.6415 | 15.3673 | 20.295 | 22.1654 | 13.3669 | 12.1639 | 17.4346 | 2.5578 | 15.8987 | 5.4603 | 0.9119 | 6.82E-01 | 7.18E-01 |
| sp\|Q9CVB6\|AI | Arpc2 | ARPC2_MOUS | 6 | 18.07 | 11.4946 | 21.0421 | 16.4868 | 13.3436 | 19.5628 | 16.8689 | 4.8858 | 16.4644 | 3.1097 | 0.9760 | 9.10E-01 | 9.25E-01 |
| sp\|Q7TQH0\|A | Atxn2l | ATX2L_MOUSE | 6 | 19.4794 | 21.2519 | 16.5144 | 13.9813 | 18.5012 | 10.2718 | 19.0819 | 2.3936 | 14.2514 | 4.1213 | 0.7469 | 1.54E-01 | 1.98E-01 |
| sp\|Q9CQC6\|B | Bzw1 | BZW1_MOUSE | 6 | 24.2737 | 20.85 | 27.04 | 10.7841 | 7.7689 | 9.28327 | 24.0546 | 3.1008 | 9.2788 | 1.5076 | 0.3857 | 1.76E-03 | 6.03E-03 |
| sp\|Q91VK1\|B | Bzw2 | BZW2_MOUSE | 6 | 27.6725 | 24.3224 | 30.9974 | 4.76587 | 5.34488 | 6.89689 | 27.6641 | 3.3375 | 5.6692 | 1.1019 | 0.2049 | 4.11E-04 | 2.94E-03 |
| sp\|P40124\|CA | Cap1 | CAP1_MOUSE | 6 | 10.8242 | 7.03455 | 10.5953 | 24.4379 | 20.3043 | 26.8039 | 9.4847 | 2.1250 | 23.8487 | 3.2896 | 2.5144 | 3.15E-03 | 8.85E-03 |
| sp\|P47757-2\|( | Capzb | CAPZB_MOUSI | 6 | 17.7201 | 13.6632 | 20.0985 | 15.7234 | 18.139 | 14.6558 | 17.1606 | 3.2539 | 16.1727 | 1.7845 | 0.9424 | 6.69E-01 | 7.06E-01 |
| sp\|O88207\|CC | Col5a1 | CO5A1_MOUS | 6 | 0.970373 | 0.258828 | 0.317628 | 35.5371 | 48.9439 | 13.9722 | 0.5156 | 0.3949 | 32.8177 | 17.6437 | 63.6484 | 3.38E-02 | 5.29E-02 |
| sp\|O55029\|CC | Copb2 | COPB2_MOUS | 6 | 19.0937 | 23.2668 | 17.6398 | 17.662 | 11.4794 | 10.8583 | 20.0001 | 2.9210 | 13.3332 | 3.7617 | 0.6667 | 7.24E-02 | 1.02E-01 |
| sp\|Q9WUM4\| | Coro1c | COR1C_MOUS | 6 | 18.2458 | 9.97181 | 19.257 | 18.0264 | 23.114 | 11.3851 | 15.8249 | 5.0941 | 17.5085 | 5.8816 | 1.1064 | 7.27E-01 | 7.58E-01 |
| sp\|P18242\|CA | Ctsd | CATD_MOUSE | 6 | 23.7679 | 17.3719 | 21.9522 | 15.6558 | 10.62 | 10.6321 | 21.0307 | 3.2961 | 12.3026 | 2.9039 | 0.5850 | 2.63E-02 | 4.31E-02 |
| sp\|Q9D4H8\|C | Cul2 | CUL2_MOUSE | 6 | 17.6109 | 27.8114 | 14.3141 | 17.0883 | 13.408 | 9.76721 | 19.9121 | 7.0368 | 13.4212 | 3.6606 | 0.6740 | 2.29E-01 | 2.82E-01 |
| sp\|Q9DCN2\|N | Cyb5r3 | NB5R3_MOUS | 6 | 24.3626 | 22.29 | 26.3705 | 11.6016 | 7.90192 | 7.47342 | 24.3410 | 2.0403 | 8.9923 | 2.2698 | 0.3694 | 9.57E-04 | 4.62E-03 |
| sp\|Q99KJ8\|DC | Dctn2 | DCTN2_MOUS | 6 | 23.6926 | 27.1436 | 17.6207 | 12.3106 | 9.20654 | 10.026 | 22.8190 | 4.8212 | 10.5144 | 1.6086 | 0.4608 | 1.38E-02 | 2.57E-02 |
| tr\|E9QNN1\|E9 | Dhx9 | E9QNN1_MOU | 6 | 17.5286 | 25.0733 | 18.9439 | 13.3068 | 15.3954 | 9.75201 | 20.5153 | 4.0103 | 12.8181 | 2.8533 | 0.6248 | 5.36E-02 | 7.82E-02 |
| sp\|Q9D2G2\|O | Dlst | ODO2_MOUSE | 6 | 22.8496 | 28.141 | 17.4743 | 13.1095 | 9.71024 | 8.71531 | 22.8216 | 5.3334 | 10.5117 | 2.3041 | 0.4606 | 2.14E-02 | 3.67E-02 |
| sp\|O08810\|U! | Eftud2 | U5S1_MOUSE | 6 | 22.0241 | 24.5929 | 19.9207 | 12.6893 | 12.0988 | 8.67425 | 22.1792 | 2.3400 | 11.1541 | 2.1678 | 0.5029 | 3.91E-03 | 1.02E-02 |
| sp\|Q6ZWX6\|IF | Eif2s1 | IF2A_MOUSE I | 6 | 21.4168 | 23.5765 | 22.1242 | 12.5309 | 11.118 | 9.23353 | 22.3725 | 1.1011 | 10.9608 | 1.6543 | 0.4899 | 5.74E-04 | 3.52E-03 |
| sp\|Q99L45\|IF | Eif2s2 | IF2B_MOUSE I | 6 | 20.3307 | 23.587 | 17.2212 | 14.9136 | 14.6411 | 9.30653 | 20.3796 | 3.1832 | 12.9537 | 3.1615 | 0.6356 | 4.56E-02 | 6.82E-02 |
| sp\|P97855\|G3 | G3bp1 | G3BP1_MOUS | 6 | 19.8348 | 20.1421 | 16.4751 | 14.7085 | 17.4932 | 11.3464 | 18.8173 | 2.0342 | 14.5160 | 3.0779 | 0.7714 | 1.14E-01 | 1.52E-01 |
| sp\|Q61598\|GI | Gdi2 | GDIB_MOUSE | 6 | 20.1426 | 16.7931 | 27.3901 | 14.0321 | 9.62455 | 12.0176 | 21.4419 | 5.4167 | 11.8914 | 2.2065 | 0.5546 | 4.74E-02 | 7.06E-02 |
| sp\|P26443\|DH | Glud1 | DHE3_MOUSE | 6 | 15.5675 | 18.2408 | 16.9423 | 21.7769 | 14.9817 | 12.4908 | 16.9169 | 1.3368 | 16.4165 | 4.8064 | 0.9704 | 8.71E-01 | 8.91E-01 |
| sp\|Q8R050\|EF | Gspt1 | ERF3A_MOUSI | 6 | 27.2577 | 22.3584 | 27.0951 | 9.97686 | 7.09674 | 6.21516 | 25.5704 | 2.7829 | 7.7629 | 1.9673 | 0.3036 | 8.26E-04 | 4.32E-03 |
| sp\|P43276\|H1 | Hist1h1b | H15_MOUSE H | 6 | 14.3328 | 18.858 | 8.86994 | 24.4071 | 21.8781 | 11.6541 | 14.0202 | 5.0014 | 19.3131 | 6.7523 | 1.3775 | 3.37E-01 | 3.98E-01 |
| sp\|P63158\|HN | Hmgb1 | HMGB1_MOU | 6 | 25.2083 | 21.6813 | 24.1143 | 13.3299 | 8.23127 | 7.435 | 23.6680 | 1.8054 | 9.6654 | 3.1984 | 0.4084 | 2.73E-03 | 8.00E-03 |
| sp\|P49312\|RC | Hnrnpa1 | ROA1_MOUSE | 6 | 25.6803 | 21.4978 | 21.6317 | 11.6196 | 10.3014 | 9.26908 | 22.9366 | 2.3771 | 10.3967 | 1.1782 | 0.4533 | 1.21E-03 | 5.12E-03 |
| sp\|Q64433\|CH | Hspe1 | CH10_MOUSE | 6 | 22.8132 | 21.7913 | 20.7967 | 15.5443 | 9.29561 | 9.75886 | 21.8004 | 1.0083 | 11.5329 | 3.4817 | 0.5290 | 8.01E-03 | 1.74E-02 |
| sp\|Q9EPL8\|IP( | Ipo7 | IPO7_MOUSE I | 6 | 26.8968 | 27.2867 | 30.9412 | 4.54512 | 4.86966 | 5.46044 | 28.3749 | 2.2310 | 4.9584 | 0.4641 | 0.1747 | 5.86E-05 | 1.39E-03 |
| sp\|P09055\|ITE | Itgb1 | ITB1_MOUSE I | 6 | 21.9724 | 19.8162 | 21.5759 | 16.205 | 10.2487 | 10.1818 | 21.1215 | 1.1477 | 12.2118 | 3.4583 | 0.5782 | 1.33E-02 | 2.51E-02 |
| sp\|Q61768\|KII | Kif5b | KINH_MOUSE | 6 | 16.7876 | 21.2185 | 17.2406 | 19.1667 | 14.2957 | 11.2909 | 18.4156 | 2.4380 | 14.9178 | 3.9746 | 0.8101 | 2.64E-01 | 3.20E-01 |
| sp\|Q9CPY7\|AI | Lap3 | AMPL_MOUSE | 6 | 23.5024 | 22.9469 | 29.8526 | 6.50224 | 7.0663 | 10.1295 | 25.4340 | 3.8367 | 7.8993 | 1.9519 | 0.3106 | 2.13E-03 | 6.83E-03 |
| sp\|P16045\|LE( | Lgals1 | LEG1_MOUSE | 6 | 26.2704 | 17.7037 | 20.4257 | 17.5944 | 10.0677 | 7.93816 | 21.4666 | 4.3772 | 11.8668 | 5.0733 | 0.5528 | 6.81E-02 | 9.70E-02 |
| sp\|P63085\|MI | Mapk1 | MK01_MOUSE | 6 | 20.3976 | 21.3888 | 22.3001 | 13.8763 | 11.8058 | 10.2314 | 21.3622 | 0.9515 | 11.9712 | 1.8281 | 0.5604 | 1.39E-03 | 5.48E-03 |
| sp\|Q3THS6\|M | Mat2a | METK2_MOUS | 6 | 26.6031 | 25.8987 | 28.3509 | 6.3717 | 6.50977 | 6.2659 | 26.9509 | 1.2626 | 6.3825 | 0.1223 | 0.2368 | 9.56E-06 | 8.92E-04 |
| sp\|P97310\|M( | Mcm2 | MCM2_MOUS | 6 | 27.2714 | 27.4754 | 32.5424 | 3.94428 | 3.33073 | 5.43575 | 29.0964 | 2.9861 | 4.2369 | 1.0826 | 0.1456 | 1.71E-04 | 1.97E-03 |
| sp\|P25206\|M( | Mcm3 | MCM3_MOUS | 6 | 25.2332 | 31.795 | 24.6908 | 5.88041 | 5.86046 | 6.54006 | 27.2397 | 3.9543 | 6.0936 | 0.3867 | 0.2237 | 7.70E-04 | 4.17E-03 |
| sp\|P49717\|M( | Mcm4 | MCM4_MOUS | 6 | 24.5054 | 25.6858 | 27.1562 | 9.12555 | 7.00635 | 6.52067 | 25.7825 | 1.3280 | 7.5509 | 1.3852 | 0.2929 | 7.98E-05 | 1.56E-03 |
| sp\|Q7TPV4\|M | Mybbp1a | MBB1A_MOU! | 6 | 24.4584 | 25.1657 | 20.087 | 10.9784 | 11.9087 | 7.4018 | 23.2370 | 2.7508 | 10.0963 | 2.3794 | 0.4345 | 3.33E-03 | 9.23E-03 |
| sp\|Q9WTI7\|M | Myo1c | MYO1C_MOU! | 6 | 4.92751 | 1.58037 | 4.48817 | 25.3333 | 43.7243 | 19.9464 | 3.6654 | 1.8190 | 29.6680 | 12.4675 | 8.0942 | 2.33E-02 | 3.93E-02 |
| sp\|P17918\|PC | Pcna | PCNA_MOUSE | 6 | 21.5094 | 20.7672 | 22.308 | 12.7666 | 10.4873 | 12.1615 | 21.5282 | 0.7706 | 11.8051 | 1.1807 | 0.5484 | 2.81E-04 | 2.45E-03 |
| sp\|Q8BKZ9\|OI | Pdhx | ODPX_MOUSE | 6 | 12.8804 | 22.8116 | 10.0289 | 17.4185 | 16.9761 | 19.8846 | 15.2403 | 6.7102 | 18.0931 | 1.5672 | 1.1872 | 5.13E-01 | 5.60E-01 |
| sp\|Q9DBJ1\|PC | Pgam1 | PGAM1_MOU! | 6 | 18.5937 | 18.9972 | 28.4316 | 13.8843 | 10.188 | 9.90515 | 22.0075 | 5.5671 | 11.3258 | 2.2202 | 0.5146 | 3.67E-02 | 5.64E-02 |
| sp\|Q61171\|PF | Prdx2 | PRDX2_MOUS | 6 | 25.4575 | 21.1596 | 24.8683 | 11.3327 | 7.15774 | 10.0241 | 23.8285 | 2.3300 | 9.5048 | 2.1354 | 0.3989 | 1.42E-03 | 5.48E-03 |
| sp\|Q99KP6\|PF | Prpf19 | PRP19_MOUSI | 6 | 22.1119 | 35.8086 | 18.287 | 8.73109 | 8.979 | 6.08245 | 25.4025 | 9.2126 | 7.9308 | 1.6056 | 0.3122 | 3.18E-02 | 5.03E-02 |
| sp\|O88685\|PF | Psmc3 | PRS6A_MOUSI | 6 | 25.3146 | 24.8629 | 24.483 | 9.98381 | 7.5068 | 7.84895 | 24.8868 | 0.4163 | 8.4465 | 1.3423 | 0.3394 | 3.50E-05 | 1.17E-03 |
| sp\|P54775\|PR | Psmc4 | PRS6B_MOUSI | 6 | 23.9257 | 29.0623 | 24.7354 | 8.08382 | 7.4259 | 6.76688 | 25.9078 | 2.7617 | 7.4255 | 0.6585 | 0.2866 | 3.53E-04 | 2.70E-03 |
| sp\|P62334\|PR | Psmc6 | PRS10_MOUSI | 6 | 21.0107 | 26.3208 | 23.0003 | 12.0562 | 10.2034 | 7.40861 | 23.4439 | 2.6827 | 9.8894 | 2.3397 | 0.4218 | 2.74E-03 | 8.02E-03 |
| sp\|Q8CI94\|PY | Pygb | PYGB_MOUSE | 6 | 17.6995 | 15.6529 | 21.4463 | 17.5449 | 11.4943 | 16.162 | 18.2662 | 2.9380 | 15.0671 | 3.1704 | 0.8249 | 2.69E-01 | 3.25E-01 |
| sp\|P46638\|RB | Rab11b | RB11B_MOUSI | 6 | 24.3167 | 19.3226 | 27.2372 | 11.9637 | 8.63395 | 8.52577 | 23.6255 | 4.0023 | 9.7078 | 1.9544 | 0.4109 | 5.65E-03 | 1.35E-02 |
| sp\|P46061\|RA | Rangap1 | RAGP1_MOUS | 6 | 26.1936 | 24.4149 | 31.7975 | 4.906 | 6.07753 | 6.61052 | 27.4687 | 3.8529 | 5.8647 | 0.8720 | 0.2135 | 6.93E-04 | 3.91E-03 |
| sp\|P62900\|RL | Rpl31 | RL31_MOUSE | 6 | 22.1931 | 20.8813 | 13.0122 | 16.7011 | 16.9271 | 10.2851 | 18.6955 | 4.9654 | 14.6378 | 3.7712 | 0.7830 | 3.23E-01 | 3.83E-01 |
| sp\|Q9DBG6\|R | Rpn2 | RPN2_MOUSE | 6 | 21.2631 | 21.4511 | 23.9275 | 14.2366 | 9.05756 | 10.0642 | 22.2139 | 1.4870 | 11.1195 | 2.7460 | 0.5006 | 3.54E-03 | 9.62E-03 |
| sp\|P62245\|RS | Rps15a | RS15A_MOUSI | 6 | 17.4711 | 21.3871 | 14.9077 | 17.9072 | 17.2184 | 11.1085 | 17.9220 | 3.2631 | 15.4114 | 3.7423 | 0.8599 | 4.31E-01 | 4.84E-01 |
| sp\|P14131\|RS | Rps16 | RS16_MOUSE | 6 | 18.002 | 22.9676 | 14.6158 | 15.5758 | 18.1176 | 10.7212 | 18.5285 | 4.2007 | 14.8049 | 3.7580 | 0.7990 | 3.16E-01 | 3.77E-01 |
| sp\|P18653\|KS | Rps6ka1 | KS6A1_MOUSI | 6 | 24.3528 | 30.3836 | 23.1974 | 6.5169 | 7.30817 | 8.24111 | 25.9779 | 3.8589 | 7.3554 | 0.8631 | 0.2831 | 1.23E-03 | 5.17E-03 |
| sp\|P62242\|RS | Rps8 | RS8_MOUSE 4 | 6 | 16.9317 | 20.4457 | 12.9347 | 18.2204 | 20.921 | 10.5465 | 16.7707 | 3.7581 | 16.5626 | 5.3823 | 0.9876 | 9.59E-01 | 9.66E-01 |
| sp\|Q63918\|SI | Sdpr | SDPR_MOUSE | 6 | 36.9238 | 31.9182 | 23.5105 | 1.34873 | 2.17426 | 4.12458 | 30.7842 | 6.7782 | 2.5492 | 1.4254 | 0.0828 | 2.12E-03 | 6.83E-03 |
| sp\|O70456\|14 | Sfn | 1433S_MOUSE | 6 | 21.6973 | 21.4137 | 25.1087 | 12.5677 | 9.44139 | 9.77129 | 22.7399 | 2.0563 | 10.5935 | 1.7177 | 0.4659 | 1.42E-03 | 5.48E-03 |
| sp\|P51881\|AI | Slc25a5 | ADT2_MOUSE | 6 | 24.4641 | 29.2251 | 22.4779 | 8.77504 | 7.77431 | 7.28355 | 25.3890 | 3.4674 | 7.9443 | 0.7601 | 0.3129 | 1.04E-03 | 4.76E-03 |
| sp\|Q8CG47\|SI | Smc4 | SMC4_MOUSE | 6 | 26.6021 | 33.4236 | 24.2554 | 4.67157 | 4.8895 | 6.15776 | 28.0937 | 4.7626 | 5.2396 | 0.8026 | 0.1865 | 1.21E-03 | 5.11E-03 |
| sp\|Q7TMK9-2 | Syncrip | HNRPQ_MOU! | 6 | 14.5902 | 18.1737 | 12.3352 | 19.6117 | 21.9898 | 13.2993 | 15.0330 | 2.9443 | 18.3003 | 4.4912 | 1.2173 | 3.51E-01 | 4.11E-01 |

| | | | | | | | | | | | | | | | | |
|---|---|---|---|---|---|---|---|---|---|---|---|---|---|---|---|---|
| sp\|P21107-2\|T | Tpm3 | TPM3_MOUSE | 6 | 23.7106 | 11.8641 | 22.8805 | 15.7989 | 15.0014 | 10.7444 | 19.4851 | 6.6130 | 13.8482 | 2.7174 | 0.7107 | 2.44E-01 | 2.98E-01 |
| sp\|Q8BFR5\|EF | Tufm | EFTU_MOUSE | 6 | 22.6252 | 24.8522 | 22.7896 | 12.3431 | 8.9403 | 8.44955 | 23.4223 | 1.2410 | 9.9110 | 2.1205 | 0.4231 | 6.78E-04 | 3.87E-03 |
| sp\|Q9DB77\|Q | Uqcrc2 | QCR2_MOUSE | 6 | 24.0011 | 26.9682 | 24.538 | 9.32189 | 8.56141 | 6.60937 | 25.1691 | 1.5810 | 8.1642 | 1.3992 | 0.3244 | 1.53E-04 | 1.88E-03 |
| sp\|P56399\|UB | Usp5 | UBP5_MOUSE | 6 | 20.9778 | 18.637 | 27.5078 | 11.6483 | 10.9215 | 10.3076 | 22.3742 | 4.5973 | 10.9591 | 0.6711 | 0.4898 | 1.31E-02 | 2.47E-02 |
| sp\|Q9WV55\|V | Vapa | VAPA_MOUSE | 6 | 24.1143 | 38.6705 | 20.6495 | 6.69497 | 5.29448 | 4.57623 | 27.8114 | 9.5625 | 5.5219 | 1.0775 | 0.1985 | 1.60E-02 | 2.88E-02 |
| sp\|Q62523\|ZY | Zyx | ZYX_MOUSE Z | 6 | 12.8002 | 10.5876 | 9.70277 | 38.5131 | 16.7436 | 11.6527 | 11.0302 | 1.5954 | 22.3031 | 14.2672 | 2.0220 | 2.45E-01 | 3.00E-01 |
| sp\|P42208\|SE | 2-Sep | SEPT2_MOUSE | 5 | 15.1733 | 16.4318 | 16.09 | 23.0944 | 15.1163 | 14.0942 | 15.8984 | 0.6508 | 17.4350 | 4.9278 | 1.0967 | 6.21E-01 | 6.60E-01 |
| sp\|O55131\|SE | 7-Sep | SEPT7_MOUSE | 5 | 16.0454 | 16.4942 | 13.4814 | 24.0772 | 16.7683 | 13.1335 | 15.3403 | 1.6254 | 17.9930 | 5.5737 | 1.1729 | 4.73E-01 | 5.23E-01 |
| sp\|P62737\|AC | Acta2 | ACTA_MOUSE | 5 | 6.22685 | 3.07811 | 6.85192 | 22.31 | 39.9121 | 21.621 | 5.3856 | 2.0227 | 27.9477 | 10.3672 | 5.1893 | 2.08E-02 | 3.59E-02 |
| sp\|Q68FL4\|SA | Ahcyl2 | SAHH3_MOUS | 5 | 24.1975 | 25.6596 | 23.2651 | 10.8053 | 8.74961 | 7.32294 | 24.3741 | 1.2070 | 8.9593 | 1.7506 | 0.3676 | 2.32E-04 | 2.27E-03 |
| sp\|Q8BK64\|AH | Ahsa1 | AHSA1_MOUS | 5 | 21.1321 | 27.4405 | 20.6133 | 14.1815 | 9.60307 | 7.02955 | 23.0620 | 3.8008 | 10.2714 | 3.6225 | 0.4454 | 1.35E-02 | 2.53E-02 |
| sp\|P10107\|AN | Anxa1 | ANXA1_MOUS | 5 | 1.84699 | 1.35534 | 1.15179 | 44.8774 | 28.1627 | 22.6059 | 1.4514 | 0.3574 | 31.8820 | 11.5922 | 21.9668 | 1.05E-02 | 2.08E-02 |
| sp\|O35643\|AP | Ap1b1 | AP1B1_MOUS | 5 | 19.6351 | 20.7029 | 19.152 | 16.0577 | 14.4514 | 10.0008 | 19.8300 | 0.7936 | 13.5033 | 3.1378 | 0.6810 | 2.76E-02 | 4.47E-02 |
| sp\|Q60865\|CA | Caprin1 | CAPR1_MOUS | 5 | 19.2859 | 19.8479 | 12.9129 | 18.442 | 18.279 | 11.2322 | 17.3489 | 3.8520 | 15.9844 | 4.1163 | 0.9213 | 6.97E-01 | 7.32E-01 |
| sp\|Q9ER72\|SY | Cars | SYCC_MOUSE | 5 | 24.252 | 25.4121 | 27.6802 | 9.25079 | 6.82742 | 6.57743 | 25.7814 | 1.7437 | 7.5519 | 1.4766 | 0.2929 | 1.59E-04 | 1.93E-03 |
| sp\|Q9QZE5\|CO | Copg1 | COPG1_MOUS | 5 | 18.3731 | 21.7656 | 18.5822 | 17.5094 | 11.9108 | 11.859 | 19.5736 | 1.9012 | 13.7597 | 3.2474 | 0.7030 | 5.55E-02 | 8.04E-02 |
| sp\|Q8VE73\|CU | Cul7 | CUL7_MOUSE | 5 | 14.6559 | 25.3268 | 14.3796 | 3.8064 | 3.81846 | 38.0129 | 18.1208 | 6.2421 | 15.2126 | 19.7457 | 0.8395 | 8.20E-01 | 8.45E-01 |
| sp\|Q9QXS6\|DI | Dbn1 | DREB_MOUSE | 5 | 18.0253 | 6.15654 | 20.2243 | 16.3733 | 24.2104 | 15.0102 | 14.8020 | 7.5675 | 18.5313 | 4.9652 | 1.2519 | 5.15E-01 | 5.62E-01 |
| sp\|Q8K1M6\|D | Dnm1l | DNM1L_MOUS | 5 | 23.1448 | 22.2505 | 25.3414 | 9.50181 | 8.39082 | 11.3706 | 23.5789 | 1.5905 | 9.7544 | 1.5059 | 0.4137 | 3.98E-04 | 2.89E-03 |
| sp\|Q9D8Y0\|EF | Efhd2 | EFHD2_MOUS | 5 | 25.8073 | 8.01626 | 25.5887 | 11.3557 | 14.0296 | 15.2025 | 19.8041 | 10.2091 | 13.5293 | 1.9716 | 0.6832 | 3.55E-01 | 4.14E-01 |
| sp\|O70194\|EI | Eif3d | EIF3D_MOUSE | 5 | 25.4331 | 28.1026 | 24.3929 | 7.24136 | 7.01681 | 7.81325 | 25.9762 | 1.9136 | 7.3571 | 0.4106 | 0.2832 | 7.94E-05 | 1.56E-03 |
| sp\|Q9DCH4\|EI | Eif3f | EIF3F_MOUSE | 5 | 24.627 | 26.9636 | 25.6677 | 7.44035 | 6.84834 | 8.45305 | 25.7528 | 1.1706 | 7.5806 | 0.8115 | 0.2944 | 2.48E-05 | 1.11E-03 |
| sp\|Q91WK2\|E | Eif3h | EIF3H_MOUSE | 5 | 14.3714 | 14.3303 | 15.048 | 20.4715 | 21.8094 | 13.9694 | 14.5832 | 0.4030 | 18.7501 | 4.1939 | 1.2857 | 1.62E-01 | 2.07E-01 |
| sp\|Q62448\|IF | Eif4g2 | IF4G2_MOUSE | 5 | 26.1078 | 23.9433 | 28.9041 | 6.38748 | 7.06581 | 7.59143 | 26.3184 | 2.4871 | 7.0149 | 0.6036 | 0.2665 | 1.98E-04 | 2.09E-03 |
| sp\|Q9JJ28\|FLI | Flii | FLII_MOUSE P | 5 | 11.2842 | 5.82992 | 13.3149 | 22.351 | 28.0189 | 19.2011 | 10.1430 | 3.8708 | 23.1903 | 4.4684 | 2.2863 | 1.87E-02 | 3.31E-02 |
| sp\|Q61584\|FX | Fxr1 | FXR1_MOUSE | 5 | 13.2878 | 17.7253 | 10.8526 | 17.384 | 28.0804 | 12.6699 | 13.9552 | 3.4846 | 19.3781 | 7.8964 | 1.3886 | 3.38E-01 | 3.98E-01 |
| sp\|Q64737\|PU | Gart | PUR2_MOUSE | 5 | 25.3788 | 24.8659 | 25.4177 | 8.9462 | 6.59696 | 8.79447 | 25.2208 | 0.3080 | 8.1125 | 1.3147 | 0.3217 | 2.55E-05 | 1.11E-03 |
| sp\|Q61543\|GS | Glg1 | GSLG1_MOUS | 5 | 17.9863 | 17.5644 | 18.852 | 17.2508 | 17.4411 | 10.9054 | 18.1342 | 0.6564 | 15.1991 | 3.7197 | 0.8381 | 2.50E-01 | 3.04E-01 |
| sp\|Q99JY0\|EC | Hadhb | ECHB_MOUSE | 5 | 21.4846 | 18.3902 | 21.5677 | 16.1554 | 10.991 | 11.4112 | 20.4808 | 1.8110 | 12.8525 | 2.8681 | 0.6275 | 1.76E-02 | 3.13E-02 |
| sp\|P22752\|H2 | Hist1h2ab | H2A1_MOUSE | 5 | 5.14019 | 8.06244 | 3.65331 | 36.0688 | 30.844 | 16.2313 | 5.6186 | 2.2432 | 27.7147 | 10.2823 | 4.9326 | 2.20E-02 | 3.76E-02 |
| sp\|P30681\|HM | Hmgb2 | HMGB2_MOU | 5 | 30.4975 | 23.7331 | 23.609 | 10.3746 | 6.59293 | 5.19292 | 25.9465 | 3.9417 | 7.3868 | 2.6805 | 0.2847 | 2.52E-03 | 7.60E-03 |
| sp\|Q99020\|RO | Hnrnpab | ROAA_MOUSE | 5 | 29.6146 | 26.8149 | 21.0182 | 8.54698 | 7.32526 | 6.68009 | 25.8159 | 4.3844 | 7.5174 | 0.9482 | 0.2912 | 2.12E-03 | 6.83E-03 |
| sp\|O35737\|HN | Hnrnph1 | HNRH1_MOUS | 5 | 17.7087 | 22.6303 | 19.2837 | 15.2559 | 13.8807 | 11.2407 | 19.8742 | 2.5134 | 13.4591 | 2.0405 | 0.6772 | 2.65E-02 | 4.33E-02 |
| sp\|Q8R081\|HN | Hnrnpl | HNRPL_MOUS | 5 | 16.7662 | 20.404 | 15.1067 | 17.6673 | 17.4291 | 12.6267 | 17.4256 | 2.7095 | 15.9077 | 2.8439 | 0.9129 | 5.40E-01 | 5.84E-01 |
| tr\|Q8VHM5\|Q | Hnrnpr | Q8VHM5_MOU | 5 | 18.1786 | 25.7045 | 19.6207 | 14.5433 | 11.9252 | 10.0276 | 21.1679 | 3.9944 | 12.1654 | 2.2674 | 0.5747 | 2.74E-02 | 4.44E-02 |
| sp\|O70503\|DH | Hsd17b12 | DHB12_MOUS | 5 | 19.5889 | 24.2915 | 19.1588 | 14.6956 | 12.0193 | 10.246 | 21.0131 | 2.8473 | 12.3203 | 2.2400 | 0.5863 | 1.42E-02 | 2.63E-02 |
| sp\|Q9JHR7\|ID | Ide | IDE_MOUSE In | 5 | 19.2076 | 18.0241 | 27.5231 | 9.0449 | 7.22868 | 18.9717 | 21.5849 | 5.1765 | 11.7484 | 6.3211 | 0.5443 | 1.05E-01 | 1.42E-01 |
| sp\|Q9D6R2\|ID | Idh3a | IDH3A_MOUS | 5 | 25.5813 | 25.6268 | 27.706 | 8.27632 | 6.33225 | 6.47729 | 26.3047 | 1.2138 | 7.0286 | 1.0830 | 0.2672 | 3.33E-05 | 1.17E-03 |
| sp\|Q91YE6\|IP | Ipo9 | IPO9_MOUSE | 5 | 23.2348 | 26.3518 | 23.8552 | 8.22287 | 7.17843 | 11.1569 | 24.4806 | 1.6499 | 8.8527 | 2.0627 | 0.3616 | 5.11E-04 | 3.35E-03 |
| sp\|Q61765\|K1 | Krt31 | K1H1_MOUSE | 5 | 2.07823 | 2.73815 | 0.663252 | 0.893579 | 0.771544 | 92.8552 | 1.8265 | 1.0601 | 31.5068 | 53.1293 | 17.2494 | 3.88E-01 | 4.46E-01 |
| sp\|Q9ERE2\|KR | Krt81 | KRT81_MOUS | 5 | 2.17185 | 3.89699 | 0.786432 | 0.46804 | 0.441652 | 92.235 | 2.2851 | 1.5584 | 31.0482 | 52.9893 | 13.5873 | 4.01E-01 | 4.56E-01 |
| sp\|P24527\|LK | Lta4h | LKHA4_MOUS | 5 | 24.8859 | 18.613 | 29.7518 | 9.86732 | 8.74586 | 8.13606 | 24.4169 | 5.5842 | 8.9164 | 0.8781 | 0.3652 | 8.97E-03 | 1.86E-02 |
| sp\|Q68FL6\|SY | Mars | SYMC_MOUSE | 5 | 22.7701 | 26.9624 | 22.1739 | 12.0124 | 8.63939 | 7.44184 | 23.9688 | 2.6096 | 9.3645 | 2.3700 | 0.3907 | 2.00E-03 | 6.57E-03 |
| sp\|Q61881\|M | Mcm7 | MCM7_MOUS | 5 | 21.2787 | 22.0287 | 21.2032 | 8.83709 | 11.6281 | 15.0242 | 21.5035 | 0.4564 | 11.8298 | 3.0985 | 0.5501 | 5.89E-03 | 1.38E-02 |
| sp\|Q80UM7\|M | Mogs | MOGS_MOUS | 5 | 21.87 | 23.2442 | 22.4938 | 9.17456 | 11.8306 | 11.3869 | 22.5360 | 0.6881 | 10.7974 | 1.4228 | 0.4791 | 2.10E-04 | 2.12E-03 |
| sp\|Q3THE2\|M | Myl12b | ML12B_MOUS | 5 | 19.3176 | 10.5943 | 20.0152 | 18.2568 | 21.2897 | 10.5265 | 16.6424 | 5.2494 | 16.6910 | 5.5498 | 1.0029 | 9.92E-01 | 9.92E-01 |
| sp\|Q60605\|M | Myl6 | MYL6_MOUSE | 5 | 19.4217 | 10.6757 | 20.5981 | 17.7909 | 21.7979 | 9.71566 | 16.8985 | 5.4211 | 16.4348 | 6.1542 | 0.9726 | 9.27E-01 | 9.39E-01 |
| sp\|P28656\|NP | Nap1l1 | NP1L1_MOUS | 5 | 17.1203 | 26.6962 | 33.9209 | 8.56208 | 6.02898 | 7.67151 | 25.9125 | 8.4277 | 7.4209 | 1.2850 | 0.2864 | 1.98E-02 | 3.46E-02 |
| sp\|Q9CZ30\|OL | Ola1 | OLA1_MOUSE | 5 | 23.8474 | 23.9036 | 28.4222 | 9.68724 | 7.12686 | 7.01274 | 25.3911 | 2.6252 | 7.9423 | 1.5123 | 0.3128 | 5.67E-04 | 3.52E-03 |
| sp\|Q8CI51\|PD | Pdlim5 | PDLI5_MOUSE | 5 | 15.5774 | 17.2379 | 14.7144 | 21.4613 | 17.8409 | 13.1681 | 15.8432 | 1.2826 | 17.4901 | 4.1577 | 1.1039 | 5.48E-01 | 5.90E-01 |
| sp\|Q9R0E2\|PL | Plod1 | PLOD1_MOUS | 5 | 21.5578 | 22.7744 | 23.2692 | 10.6604 | 9.06684 | 12.6714 | 22.5338 | 0.8807 | 10.7995 | 1.8063 | 0.4793 | 5.38E-04 | 3.44E-03 |
| sp\|P24369\|PP | Ppib | PPIB_MOUSE F | 5 | 11.0375 | 4.82236 | 6.89498 | 26.7579 | 28.0431 | 22.4442 | 7.5849 | 3.1645 | 25.7484 | 2.9328 | 3.3947 | 1.88E-03 | 6.32E-03 |
| sp\|O08795\|GL | Prkcsh | GLU2B_MOUS | 5 | 28.824 | 21.5417 | 20.5831 | 12.0847 | 8.19 | 8.77648 | 23.6496 | 4.5067 | 9.6837 | 2.0999 | 0.4095 | 8.25E-03 | 1.77E-02 |
| sp\|Q3TLH4\|PR | Prrc2c | PRC2C_MOUS | 5 | 22.8657 | 21.3332 | 13.7033 | 15.9085 | 16.4118 | 9.77751 | 19.3007 | 4.9077 | 14.0326 | 3.6936 | 0.7271 | 2.12E-01 | 2.62E-01 |
| sp\|Q9Z2U1\|PS | Psma5 | PSA5_MOUSE | 5 | 22.5457 | 25.3411 | 27.8385 | 8.76747 | 5.71756 | 9.78965 | 25.2418 | 2.6478 | 8.0916 | 2.1185 | 0.3206 | 9.36E-04 | 4.58E-03 |
| sp\|Q9Z2U0\|PS | Psma7 | PSA7_MOUSE | 5 | 27.333 | 21.9572 | 25.4451 | 9.18045 | 6.68449 | 9.39981 | 24.9118 | 2.7273 | 8.4216 | 1.5084 | 0.3381 | 7.87E-04 | 4.24E-03 |
| sp\|Q9WVJ2\|PS | Psmd13 | PSD13_MOUS | 5 | 21.6797 | 23.4814 | 22.3138 | 13.3865 | 9.243 | 9.89548 | 22.4916 | 0.9139 | 10.8417 | 2.2279 | 0.4820 | 1.11E-03 | 4.90E-03 |
| sp\|P62821\|RA | Rab1A | RAB1A_MOUS | 5 | 21.1699 | 19.7013 | 22.7723 | 14.542 | 10.8239 | 10.9905 | 21.2145 | 1.5360 | 12.1188 | 2.1002 | 0.5713 | 3.76E-03 | 9.91E-03 |
| sp\|P26043\|RA | Rdx | RADI_MOUSE | 5 | 22.3836 | 23.4579 | 21.3743 | 13.0633 | 11.0827 | 8.63832 | 22.4053 | 1.0420 | 10.9281 | 2.2165 | 0.4877 | 1.25E-03 | 5.21E-03 |
| sp\|Q6ZWV3\|R | Rpl10 | RL10_MOUSE | 5 | 19.0538 | 21.3526 | 16.8331 | 16.0161 | 15.8703 | 10.8739 | 19.0798 | 2.2599 | 14.2534 | 2.9277 | 0.7470 | 8.67E-02 | 1.20E-01 |
| sp\|P35979\|RL | Rpl12 | RL12_MOUSE | 5 | 17.741 | 24.1977 | 15.4177 | 17.417 | 16.525 | 8.70157 | 19.1188 | 4.5493 | 14.2145 | 4.7951 | 0.7435 | 2.68E-01 | 3.25E-01 |
| sp\|P47963\|RL | Rpl13 | RL13_MOUSE | 5 | 17.7493 | 25.6434 | 13.1979 | 16.5306 | 18.0483 | 8.83053 | 18.8635 | 6.2971 | 14.4698 | 4.9424 | 0.7671 | 3.96E-01 | 4.52E-01 |
| sp\|Q9CPR4\|RL | Rpl17 | RL17_MOUSE | 5 | 13.698 | 16.8043 | 11.0476 | 31.1716 | 17.7909 | 9.4877 | 13.8500 | 2.8814 | 19.4834 | 10.9406 | 1.4067 | 4.37E-01 | 4.88E-01 |

| | | | | | | | | | | | | | | | | |
|---|---|---|---|---|---|---|---|---|---|---|---|---|---|---|---|---|
| sp\|P84099\|RL | Rpl19 | RL19_MOUSE | 5 | 18.6252 | 21.9807 | 13.1436 | 17.6437 | 18.7646 | 9.84218 | 17.9165 | 4.4610 | 15.4168 | 4.8602 | 0.8605 | 5.47E-01 | 5.90E-01 |
| sp\|P61255\|RL | Rpl26 | RL26_MOUSE | 5 | 19.7243 | 18.8127 | 15.0706 | 16.8646 | 17.1956 | 12.3321 | 17.8692 | 2.4661 | 15.4641 | 2.7174 | 0.8654 | 3.20E-01 | 3.80E-01 |
| sp\|P62754\|RS | Rps6 | RS6_MOUSE 4 | 5 | 18.7586 | 21.0376 | 13.6263 | 17.7641 | 17.7673 | 11.0462 | 17.8075 | 3.7961 | 15.5259 | 3.8795 | 0.8719 | 5.07E-01 | 5.55E-01 |
| sp\|P62082\|RS | Rps7 | RS7_MOUSE 4 | 5 | 18.7323 | 20.217 | 14.8692 | 18.7579 | 16.8206 | 10.603 | 17.9395 | 2.7606 | 15.3938 | 4.2606 | 0.8581 | 4.34E-01 | 4.86E-01 |
| sp\|P14206\|RS | Rpsa | RSSA_MOUSE | 5 | 24.5529 | 22.836 | 29.1481 | 8.66932 | 7.07385 | 7.71992 | 25.5123 | 3.2636 | 7.8210 | 0.8025 | 0.3066 | 8.03E-04 | 4.27E-03 |
| sp\|Q8K2B3\|SD | Sdha | SDHA_MOUSE | 5 | 24.5411 | 24.573 | 27.2122 | 9.13895 | 6.61794 | 7.91684 | 25.4421 | 1.5330 | 7.8912 | 1.2607 | 0.3102 | 1.06E-04 | 1.63E-03 |
| sp\|Q9CY58\|PA | Serbp1 | PAIRB_MOUSE | 5 | 29.837 | 23.4782 | 20.8324 | 10.665 | 8.67026 | 6.51722 | 24.7159 | 4.6281 | 8.6175 | 2.0744 | 0.3487 | 5.34E-03 | 1.30E-02 |
| sp\|Q64674\|SP | Srm | SPEE_MOUSE | 5 | 29.3623 | 25.1026 | 32.0663 | 4.3476 | 4.48016 | 4.641 | 28.8437 | 3.5107 | 4.4896 | 0.1469 | 0.1557 | 2.76E-04 | 2.44E-03 |
| sp\|P32067\|LA | Ssb | LA_MOUSE Lu | 5 | 27.305 | 24.4681 | 25.9997 | 7.6303 | 7.11069 | 7.48617 | 25.9243 | 1.4200 | 7.4091 | 0.2683 | 0.2858 | 2.44E-05 | 1.11E-03 |
| sp\|Q08943\|SS | Ssrp1 | SSRP1_MOUSE | 5 | 20.2784 | 15.8044 | 16.3355 | 15.2985 | 18.7995 | 13.4839 | 17.4728 | 2.4442 | 15.8606 | 2.7020 | 0.9077 | 4.86E-01 | 5.35E-01 |
| sp\|Q920B9\|SP | Supt16h | SP16H_MOUSE | 5 | 17.5223 | 21.9125 | 16.8004 | 14.9798 | 17.5163 | 11.2688 | 18.7451 | 2.7667 | 14.5883 | 3.1421 | 0.7782 | 1.61E-01 | 2.05E-01 |
| sp\|Q8C1A5\|TH | Thop1 | THOP1_MOUS | 5 | 25.6354 | 22.3015 | 30.8749 | 7.89473 | 6.38827 | 6.90525 | 26.2706 | 4.3219 | 7.0628 | 0.7655 | 0.2688 | 1.62E-03 | 5.92E-03 |
| sp\|P58774\|TP | Tpm2 | TPM2_MOUSE | 5 | 14.9277 | 5.40879 | 11.8829 | 24.0494 | 25.0989 | 18.6323 | 10.7398 | 4.8613 | 22.5935 | 3.4704 | 2.1037 | 2.64E-02 | 4.32E-02 |
| sp\|Q6IRU2\|TP | Tpm4 | TPM4_MOUSE | 5 | 14.1361 | 8.47948 | 14.9171 | 24.176 | 22.9398 | 15.3516 | 12.5109 | 3.5131 | 20.8225 | 4.7781 | 1.6643 | 7.22E-02 | 1.02E-01 |
| sp\|Q62191\|RC | Trim21 | RO52_MOUSE | 5 | 23.4799 | 44.3934 | 17.0248 | 5.03547 | 4.97229 | 5.09407 | 28.2994 | 14.3067 | 5.0339 | 0.0609 | 0.1779 | 4.80E-02 | 7.12E-02 |
| sp\|Q922F4\|TB | Tubb6 | TBB6_MOUSE | 5 | 23.6677 | 27.9613 | 29.8795 | 5.93619 | 6.73218 | 5.82316 | 27.1695 | 3.1807 | 6.1638 | 0.4954 | 0.2269 | 3.49E-04 | 2.70E-03 |
| sp\|P10639\|TH | Txn | THIO_MOUSE | 5 | 20.0399 | 29.0598 | 19.179 | 11.5426 | 6.9421 | 13.2366 | 22.7596 | 5.4731 | 10.5738 | 3.2572 | 0.4646 | 2.95E-02 | 4.73E-02 |
| sp\|Q9Z1F9\|SA | Uba2 | SAE2_MOUSE | 5 | 23.2565 | 19.3527 | 29.1894 | 8.47398 | 7.42277 | 12.3046 | 23.9329 | 4.9531 | 9.4005 | 2.5694 | 0.3928 | 1.07E-02 | 2.13E-02 |
| sp\|P0CG49\|UE | Ubb | UBB_MOUSE F | 5 | 19.0124 | 26.2979 | 21.5576 | 12.3046 | 10.6817 | 10.1459 | 22.2893 | 3.6975 | 11.0441 | 1.1240 | 0.4955 | 7.28E-03 | 1.61E-02 |
| sp\|Q9CZ13\|Q | Uqcrc1 | QCR1_MOUSE | 5 | 24.3651 | 27.7727 | 28.4428 | 7.72717 | 6.18796 | 5.50421 | 26.8602 | 2.1866 | 6.4731 | 1.1386 | 0.2410 | 1.38E-04 | 1.82E-03 |
| sp\|P52479\|UE | Usp10 | UBP10_MOUS | 5 | 20.3753 | 32.4278 | 21.1935 | 9.21023 | 7.89473 | 8.89851 | 24.6655 | 6.7348 | 8.6678 | 0.6874 | 0.3514 | 1.49E-02 | 2.74E-02 |
| sp\|Q9QY76\|V | Vapb | VAPB_MOUSE | 5 | 23.91 | 39.3613 | 19.6679 | 6.41656 | 5.78556 | 4.85873 | 27.6464 | 10.3647 | 5.6870 | 0.7836 | 0.2057 | 2.16E-02 | 3.69E-02 |
| sp\|Q91V12\|B | Acot7 | BACH_MOUSE | 4 | 20.357 | 21.8767 | 32.7477 | 8.63141 | 6.99092 | 9.39626 | 24.9938 | 6.7579 | 8.3395 | 1.2289 | 0.3337 | 1.37E-02 | 2.56E-02 |
| sp\|Q99JY9\|AR | Actr3 | ARP3_MOUSE | 4 | 17.7063 | 16.2926 | 21.9667 | 16.9915 | 13.4343 | 13.6086 | 18.6552 | 2.9537 | 14.6781 | 2.0053 | 0.7868 | 1.26E-01 | 1.67E-01 |
| sp\|P31230\|AI | Aimp1 | AIMP1_MOUS | 4 | 26.8551 | 29.0817 | 23.8389 | 8.30861 | 5.27066 | 6.64499 | 26.5919 | 2.6313 | 6.7414 | 1.5213 | 0.2535 | 3.48E-04 | 2.70E-03 |
| sp\|Q9JLJ2\|AL | Aldh9a1 | AL9A1_MOUSI | 4 | 27.3164 | 23.8665 | 28.9387 | 7.63393 | 5.53421 | 6.71037 | 26.7072 | 2.5904 | 6.6262 | 1.0524 | 0.2481 | 2.40E-04 | 2.29E-03 |
| sp\|P84091\|AP | Ap2m1 | AP2M1_MOUS | 4 | 15.2946 | 18.76 | 11.5579 | 17.9124 | 20.2454 | 16.2297 | 15.2042 | 3.6019 | 18.1292 | 2.0166 | 1.1924 | 2.87E-01 | 3.45E-01 |
| sp\|Q9WV32\|A | Arpc1b | ARC1B_MOUS | 4 | 17.3568 | 13.2357 | 19.5356 | 19.1112 | 17.7449 | 13.0159 | 16.7094 | 3.1995 | 16.6240 | 3.1985 | 0.9949 | 9.75E-01 | 9.79E-01 |
| sp\|P59999\|AR | Arpc4 | ARPC4_MOUS | 4 | 18.4907 | 15.1997 | 22.996 | 16.5818 | 13.11 | 13.6216 | 18.8955 | 3.9139 | 14.4378 | 1.8743 | 0.7641 | 1.50E-01 | 1.94E-01 |
| sp\|P50518\|VA | Atp6v1e1 | VATE1_MOUSI | 4 | 23.2402 | 20.2751 | 25.9796 | 12.3147 | 8.02795 | 10.1625 | 23.1650 | 2.8530 | 10.1684 | 2.1434 | 0.4390 | 3.23E-03 | 9.05E-03 |
| sp\|Q91XV3\|B | Basp1 | BASP1_MOUSI | 4 | 28.834 | 20.7026 | 20.8054 | 12.7758 | 7.94566 | 8.93654 | 23.4473 | 4.6653 | 9.8860 | 2.5512 | 0.4216 | 1.15E-02 | 2.24E-02 |
| sp\|P24288\|BC | Bcat1 | BCAT1_MOUSI | 4 | 27.15 | 26.7516 | 33.4234 | 4.27654 | 3.62942 | 4.76904 | 29.1083 | 3.7423 | 4.2250 | 0.5716 | 0.1451 | 3.39E-04 | 2.68E-03 |
| tr\|E9QA16\|E9 | Cald1 | E9QA16_MOU | 4 | 4.8775 | 1.0555 | 3.27142 | 30.5393 | 41.4251 | 18.8311 | 3.0681 | 1.9191 | 30.2652 | 11.2995 | 9.8643 | 1.47E-02 | 2.72E-02 |
| sp\|Q6A068\|CD | Cdc5l | CDC5L_MOUSI | 4 | 21.4351 | 29.6884 | 18.2844 | 10.7393 | 11.9571 | 7.89567 | 23.1360 | 5.8892 | 10.1974 | 2.0842 | 0.4408 | 2.30E-02 | 3.90E-02 |
| sp\|P11440\|CD | Cdk1 | CDK1_MOUSE | 4 | 20.8773 | 21.9736 | 21.3385 | 13.0301 | 11.9323 | 10.8482 | 21.3965 | 0.5504 | 11.9369 | 1.0910 | 0.5579 | 1.79E-04 | 1.97E-03 |
| sp\|Q9Z1Q5\|CL | Clic1 | CLIC1_MOUSE | 4 | 21.2772 | 18.429 | 26.9508 | 15.3314 | 9.57355 | 8.43794 | 22.2190 | 4.3383 | 11.1143 | 3.6960 | 0.5002 | 2.79E-02 | 4.51E-02 |
| sp\|Q9CQI6\|CC | Cotl1 | COTL1_MOUSI | 4 | 23.8791 | 19.8323 | 26.6219 | 12.8051 | 9.65895 | 7.20267 | 23.4444 | 3.4156 | 9.8889 | 2.8083 | 0.4218 | 6.05E-03 | 1.41E-02 |
| sp\|Q91W50\|C | Csde1 | CSDE1_MOUSI | 4 | 18.8462 | 23.906 | 14.9926 | 16.7643 | 15.157 | 10.334 | 19.2483 | 4.4703 | 14.0851 | 3.3465 | 0.7318 | 1.85E-01 | 2.32E-01 |
| sp\|Q8BK63\|K | Csnk1a1 | KC1A_MOUSE | 4 | 20.8603 | 31.1806 | 17.8401 | 9.25799 | 9.58084 | 11.2801 | 23.2937 | 6.9952 | 10.0396 | 1.0863 | 0.4310 | 3.16E-02 | 5.00E-02 |
| sp\|P53395\|OD | Dbt | ODB2_MOUSE | 4 | 17.9818 | 21.3898 | 15.3221 | 11.0096 | 18.1109 | 16.1858 | 18.2312 | 3.0415 | 15.1021 | 3.6726 | 0.8284 | 3.19E-01 | 3.80E-01 |
| sp\|O35286\|DH | Dhx15 | DHX15_MOUS | 4 | 21.4595 | 24.2345 | 18.3645 | 13.0206 | 12.9519 | 9.96891 | 21.3528 | 2.9365 | 11.9805 | 1.7424 | 0.5611 | 8.94E-03 | 1.86E-02 |
| sp\|O08749\|DL | Dld | DLDH_MOUSE | 4 | 21.8323 | 22.1276 | 24.3455 | 11.8437 | 10.1097 | 9.74115 | 22.7685 | 1.3737 | 10.5649 | 1.1227 | 0.4640 | 2.84E-04 | 2.45E-03 |
| sp\|Q6NZB0\|DI | Dnajc8 | DNJC8_MOUSI | 4 | 34.8353 | 22.4109 | 25.6776 | 7.07557 | 4.36136 | 5.63931 | 27.6413 | 6.4408 | 5.6921 | 1.3579 | 0.2059 | 4.46E-03 | 1.13E-02 |
| sp\|P39054\|DY | Dnm2 | DYN2_MOUSE | 4 | 18.9157 | 21.2078 | 20.5168 | 16.027 | 12.1921 | 11.1406 | 20.2134 | 1.1758 | 13.1199 | 2.5719 | 0.6491 | 1.22E-02 | 2.34E-02 |
| sp\|Q8R1Q8\|D | Dync1li1 | DC1L1_MOUSI | 4 | 21.1398 | 23.1467 | 20.3393 | 12.8738 | 10.4663 | 12.0341 | 21.5419 | 1.4463 | 11.7914 | 1.2220 | 0.5474 | 8.73E-04 | 4.47E-03 |
| sp\|Q9Z1D1\|EI | Eif3g | EIF3G_MOUSE | 4 | 30.9293 | 25.9328 | 23.5025 | 7.92089 | 5.37119 | 6.34338 | 26.7882 | 3.7866 | 6.5452 | 1.2868 | 0.2443 | 9.33E-04 | 4.58E-03 |
| sp\|Q6DYE8\|EN | Enpp3 | ENPP3_MOUSI | 4 | 31.4797 | 27.2511 | 32.2281 | 1.79882 | 2.70498 | 4.53734 | 30.3196 | 2.6836 | 3.0137 | 1.3951 | 0.0994 | 9.77E-05 | 1.63E-03 |
| sp\|Q9D379\|H | Ephx1 | HYEP_MOUSE | 4 | 11.59 | 12.793 | 13.137 | 31.8868 | 17.7965 | 12.7967 | 12.5067 | 0.8123 | 20.8267 | 9.8992 | 1.6652 | 2.20E-01 | 2.72E-01 |
| sp\|Q8R180\|EF | Ero1l | ERO1A_MOUS | 4 | 20.4803 | 17.8837 | 30.5968 | 10.6496 | 9.19203 | 11.1976 | 22.9869 | 6.7170 | 10.3464 | 1.0366 | 0.4501 | 3.22E-02 | 5.09E-02 |
| sp\|Q8BWY3\|E | Etf1 | ERF1_MOUSE | 4 | 24.612 | 20.8353 | 26.2458 | 11.1445 | 8.60714 | 8.55536 | 23.8977 | 2.7751 | 9.4357 | 1.4801 | 0.3948 | 1.35E-03 | 5.43E-03 |
| sp\|P35922\|FM | Fmr1 | FMR1_MOUSE | 4 | 7.71326 | 8.43914 | 6.20553 | 26.3222 | 33.3753 | 17.9447 | 7.4526 | 1.1394 | 25.8807 | 7.7248 | 3.4727 | 1.50E-02 | 2.74E-02 |
| sp\|P56959\|FU | Fus | FUS_MOUSE R | 4 | 20.9294 | 16.8185 | 17.0695 | 16.4379 | 18.804 | 9.94065 | 18.2725 | 2.3044 | 15.0609 | 4.5893 | 0.8242 | 3.40E-01 | 4.00E-01 |
| sp\|P97379\|G3 | G3bp2 | G3BP2_MOUS | 4 | 13.8376 | 15.5736 | 11.1332 | 17.6598 | 27.197 | 14.5987 | 13.5148 | 2.2377 | 19.8185 | 6.5707 | 1.4664 | 1.91E-01 | 2.39E-01 |
| sp\|P50396\|GD | Gdi1 | GDIA_MOUSE | 4 | 21.9852 | 19.3685 | 26.6115 | 13.056 | 9.00841 | 9.97037 | 22.6551 | 3.6677 | 10.6783 | 2.1146 | 0.4713 | 8.04E-03 | 1.74E-02 |
| sp\|Q8K0D5\|EF | Gfm1 | EFGM_MOUSE | 4 | 29.2119 | 24.7428 | 35.1047 | 3.26065 | 3.05798 | 4.62197 | 29.6865 | 5.1972 | 3.6469 | 0.8505 | 0.1228 | 1.02E-03 | 4.76E-03 |
| sp\|P02301\|H3 | H3f3c | H3C_MOUSE H | 4 | 5.3595 | 9.81344 | 4.24853 | 33.5449 | 26.1368 | 20.8968 | 6.4738 | 2.9451 | 26.8595 | 6.3549 | 4.1489 | 7.28E-03 | 1.61E-02 |
| sp\|Q61035\|SY | Hars | SYHC_MOUSE | 4 | 20.3975 | 17.8078 | 25.0006 | 10.7588 | 14.102 | 11.9332 | 21.0686 | 3.6431 | 12.2647 | 1.6961 | 0.5821 | 1.92E-02 | 3.37E-02 |
| sp\|P51660\|DH | Hsd17b4 | DHB4_MOUSE | 4 | 16.1903 | 17.977 | 20.9285 | 15.2927 | 13.5216 | 16.0898 | 18.3653 | 2.3928 | 14.9680 | 1.3145 | 0.8150 | 9.74E-02 | 1.33E-01 |
| sp\|P52293\|IM | Kpna2 | IMA1_MOUSE | 4 | 22.4183 | 24.6065 | 22.9852 | 12.1682 | 10.9883 | 6.83353 | 23.3367 | 1.1357 | 9.9967 | 2.8022 | 0.4284 | 1.58E-03 | 5.83E-03 |
| sp\|Q99M74\|K | Krt82 | KRT82_MOUSE | 4 | 1.54007 | 4.33052 | 0.305286 | 0.233674 | 0.664365 | 92.9261 | 2.0586 | 2.0621 | 31.2747 | 53.3921 | 15.1920 | 3.97E-01 | 4.53E-01 |
| sp\|Q9Z2I0\|LET | Letm1 | LETM1_MOUS | 4 | 23.1847 | 23.5065 | 27.427 | 9.85349 | 5.97664 | 10.0517 | 24.7061 | 2.3619 | 8.6273 | 2.2977 | 0.3492 | 1.07E-03 | 4.82E-03 |
| sp\|Q9ERG0\|LI | Lima1 | LIMA1_MOUSI | 4 | 10.1156 | 3.94304 | 8.54377 | 26.6197 | 32.8501 | 17.9278 | 7.5341 | 3.2077 | 25.7992 | 7.4949 | 3.4243 | 1.78E-02 | 3.17E-02 |
| sp\|Q922Q8\|LF | Lrrc59 | LRC59_MOUSE | 4 | 21.1513 | 18.9785 | 19.112 | 16.0671 | 15.7081 | 8.98313 | 19.7473 | 1.2178 | 13.5861 | 3.9903 | 0.6880 | 6.28E-02 | 9.00E-02 |

| | | | | | | | | | | | | | | | | |
|---|---|---|---|---|---|---|---|---|---|---|---|---|---|---|---|---|
| sp\|Q6PGB6\|N. | Naa50 | NAA50_MOUS | 4 | 26.0633 | 23.5554 | 23.925 | 11.8471 | 7.99462 | 6.61471 | 24.5146 | 1.3539 | 8.8188 | 2.7118 | 0.3597 | 8.55E-04 | 4.41E-03 |
| sp\|Q6GQT9\|N | Nomo1 | NOMO1_MOU | 4 | 20.123 | 25.1333 | 20.5175 | 12.6246 | 8.40651 | 13.1952 | 21.9246 | 2.7858 | 11.4088 | 2.6156 | 0.5204 | 8.86E-03 | 1.86E-02 |
| sp\|Q7TQI3\|OT | Otub1 | OTUB1_MOUS | 4 | 20.252 | 16.5666 | 27.0298 | 11.2928 | 12.1746 | 12.6842 | 21.2828 | 5.3072 | 12.0505 | 0.7039 | 0.5662 | 4.05E-02 | 6.14E-02 |
| sp\|Q9D0K2\|SC | Oxct1 | SCOT1_MOUSI | 4 | 24.0601 | 22.1472 | 27.464 | 12.3948 | 7.16925 | 6.76467 | 24.5571 | 2.6930 | 8.7762 | 3.1403 | 0.3574 | 2.72E-03 | 8.00E-03 |
| sp\|P60335\|PC | Pcbp1 | PCBP1_MOUSI | 4 | 24.8954 | 24.5707 | 26.5161 | 9.71991 | 6.41948 | 7.87844 | 25.3274 | 1.0422 | 8.0059 | 1.6539 | 0.3161 | 1.05E-04 | 1.63E-03 |
| sp\|P23506\|PII | Pcmt1 | PIMT_MOUSE | 4 | 30.3653 | 30.9436 | 23.16 | 4.46047 | 4.5906 | 6.48006 | 28.1563 | 4.3366 | 5.1770 | 1.1303 | 0.1839 | 8.88E-04 | 4.49E-03 |
| sp\|Q5SUR0\|PU | Pfas | PUR4_MOUSE | 4 | 23.2623 | 25.381 | 34.8991 | 5.7928 | 4.85171 | 5.81303 | 27.8475 | 6.1981 | 5.4858 | 0.5493 | 0.1970 | 3.39E-03 | 9.35E-03 |
| sp\|Q9WUA3\|I | Pfkp | K6PP_MOUSE | 4 | 20.6145 | 27.6308 | 22.3669 | 11.2878 | 8.67169 | 9.42836 | 23.5374 | 3.6517 | 9.7960 | 1.3462 | 0.4162 | 3.62E-03 | 9.78E-03 |
| sp\|Q9D819\|IP | Ppa1 | IPYR_MOUSE I | 4 | 19.2534 | 20.482 | 16.5183 | 16.8552 | 13.2108 | 13.6802 | 18.7512 | 2.0290 | 14.5821 | 1.9825 | 0.7777 | 6.36E-02 | 9.09E-02 |
| sp\|P62137\|PP | Ppp1ca | PP1A_MOUSE | 4 | 19.9424 | 16.5691 | 19.8421 | 14.5114 | 14.349 | 14.786 | 18.7845 | 1.9193 | 14.5488 | 0.2209 | 0.7745 | 1.91E-02 | 3.37E-02 |
| sp\|Q9R1P0\|PS | Psma4 | PSA4_MOUSE | 4 | 23.7466 | 25.3408 | 23.9107 | 11.063 | 7.49601 | 8.44288 | 24.3327 | 0.8769 | 9.0006 | 1.8477 | 0.3699 | 2.03E-04 | 2.09E-03 |
| sp\|Q9QUM9\|I | Psma6 | PSA6_MOUSE | 4 | 22.8483 | 26.8038 | 22.2965 | 9.71585 | 7.3458 | 10.9897 | 23.9829 | 2.4585 | 9.3505 | 1.8492 | 0.3899 | 1.18E-03 | 5.07E-03 |
| sp\|O55234\|PS | Psmb5 | PSB5_MOUSE | 4 | 21.8054 | 23.6243 | 20.8308 | 12.838 | 9.87852 | 11.0231 | 22.0868 | 1.4179 | 11.2465 | 1.4923 | 0.5092 | 8.02E-04 | 4.27E-03 |
| sp\|Q60692\|PS | Psmb6 | PSB6_MOUSE | 4 | 22.2953 | 28.2256 | 24.5395 | 10.588 | 6.79247 | 7.55911 | 25.0201 | 2.9942 | 8.3132 | 2.0070 | 0.3323 | 1.31E-03 | 5.33E-03 |
| sp\|P62196\|PR | Psmc5 | PRS8_MOUSE | 4 | 24.6677 | 28.5508 | 26.0065 | 8.3554 | 6.39489 | 6.02477 | 26.4083 | 1.9725 | 6.9250 | 1.2525 | 0.2622 | 1.34E-04 | 1.82E-03 |
| sp\|P97371\|PS | Psme1 | PSME1_MOUS | 4 | 27.4684 | 18.0383 | 27.8244 | 11.4922 | 7.28467 | 7.89205 | 24.4437 | 5.5501 | 8.8896 | 2.2743 | 0.3637 | 1.09E-02 | 2.15E-02 |
| sp\|P17225\|PT | Ptbp1 | PTBP1_MOUSI | 4 | 16.1306 | 20.0584 | 14.5063 | 17.7829 | 18.8002 | 12.7216 | 16.8984 | 2.8546 | 16.4349 | 3.2558 | 0.9726 | 8.62E-01 | 8.84E-01 |
| sp\|Q3UEB3\|PU | Puf60 | PUF60_MOUSI | 4 | 19.3517 | 23.2666 | 17.7841 | 15.4166 | 13.4523 | 10.7287 | 20.1341 | 2.8238 | 13.1992 | 2.3542 | 0.6556 | 3.09E-02 | 4.91E-02 |
| sp\|Q64012\|RA | Raly | RALY_MOUSE | 4 | 15.3126 | 25.4907 | 14.7749 | 15.3365 | 16.3598 | 12.7256 | 18.5261 | 6.0375 | 14.8073 | 1.8740 | 0.7993 | 3.66E-01 | 4.26E-01 |
| sp\|Q91VM5\|R | Rbmxl1 | RMXL1_MOUS | 4 | 16.7815 | 17.4252 | 11.0955 | 19.2295 | 22.5709 | 12.8974 | 15.1007 | 3.4835 | 18.2326 | 4.9132 | 1.2074 | 4.19E-01 | 4.72E-01 |
| sp\|P19253\|RL | Rpl13a | RL13A_MOUSI | 4 | 15.2935 | 19.7782 | 12.6278 | 19.4851 | 21.4786 | 11.3368 | 15.8998 | 3.6136 | 17.4335 | 5.3732 | 1.0965 | 7.03E-01 | 7.37E-01 |
| sp\|P35980\|RL | Rpl18 | RL18_MOUSE | 4 | 15.1542 | 19.94 | 12.9429 | 18.709 | 22.724 | 10.5298 | 16.0124 | 3.5766 | 17.3209 | 6.2145 | 1.0817 | 7.68E-01 | 7.97E-01 |
| sp\|P62717\|RL | Rpl18a | RL18A_MOUSI | 4 | 18.0979 | 18.2628 | 13.4985 | 18.8198 | 20.5742 | 10.7468 | 16.6197 | 2.7043 | 16.7136 | 5.2413 | 1.0056 | 9.79E-01 | 9.82E-01 |
| sp\|P62751\|RL | Rpl23a | RL23A_MOUSI | 4 | 19.5238 | 19.992 | 11.608 | 21.0809 | 18.3134 | 9.48188 | 17.0413 | 4.7112 | 16.2921 | 6.0579 | 0.9560 | 8.74E-01 | 8.93E-01 |
| sp\|P41105\|RL | Rpl28 | RL28_MOUSE | 4 | 16.2147 | 20.1351 | 12.6734 | 21.1521 | 19.3856 | 10.4391 | 16.3411 | 3.7325 | 16.9923 | 5.7435 | 1.0399 | 8.77E-01 | 8.94E-01 |
| sp\|P62889\|RL | Rpl30 | RL30_MOUSE | 4 | 15.1991 | 20.1999 | 14.464 | 18.6367 | 18.7418 | 12.7586 | 16.6210 | 3.1211 | 16.7124 | 3.4245 | 1.0055 | 9.74E-01 | 9.79E-01 |
| sp\|Q9D1R9\|RI | Rpl34 | RL34_MOUSE | 4 | 17.5991 | 22.0993 | 14.5949 | 18.48 | 17.3262 | 9.90058 | 18.0978 | 3.7770 | 15.2356 | 4.6561 | 0.8418 | 4.55E-01 | 5.06E-01 |
| sp\|P51410\|RL | Rpl9 | RL9_MOUSE 6 | 4 | 16.0702 | 20.0208 | 13.2689 | 19.348 | 20.3854 | 10.9067 | 16.4533 | 3.3922 | 16.8800 | 5.1990 | 1.0259 | 9.11E-01 | 9.26E-01 |
| sp\|P63325\|RS | Rps10 | RS10_MOUSE | 4 | 25.5151 | 20.4486 | 22.0032 | 13.6873 | 11.267 | 7.07877 | 22.6556 | 2.5955 | 10.6777 | 3.3434 | 0.4713 | 8.04E-03 | 1.74E-02 |
| sp\|P63276\|RS | Rps17 | RS17_MOUSE | 4 | 20.5944 | 21.4593 | 16.0159 | 15.8887 | 15.2783 | 10.7634 | 19.3565 | 2.9252 | 13.9768 | 2.7996 | 0.7221 | 8.28E-02 | 1.15E-01 |
| sp\|P62852\|RS | Rps25 | RS25_MOUSE | 4 | 20.9636 | 22.3007 | 14.4544 | 14.7872 | 17.0921 | 10.4019 | 19.2396 | 4.1977 | 14.0937 | 3.3986 | 0.7325 | 1.74E-01 | 2.20E-01 |
| sp\|P07742\|RII | Rrm1 | RIR1_MOUSE I | 4 | 27.957 | 25.3155 | 32.908 | 3.62906 | 4.59059 | 5.59988 | 28.7268 | 3.8543 | 4.6065 | 0.9855 | 0.1604 | 4.65E-04 | 3.17E-03 |
| sp\|Q99LF4\|RT | Rtcb | RTCB_MOUSE | 4 | 14.9194 | 19.0891 | 15.1987 | 17.1684 | 20.6374 | 12.9871 | 16.4024 | 2.3309 | 16.9310 | 3.8307 | 1.0322 | 8.48E-01 | 8.71E-01 |
| sp\|Q9WTM5\|I | Ruvbl2 | RUVB2_MOUS | 4 | 22.8273 | 26.7964 | 23.9906 | 9.89697 | 9.23765 | 7.25108 | 24.5381 | 2.0404 | 8.7952 | 1.3773 | 0.3584 | 3.78E-04 | 2.84E-03 |
| sp\|Q9R1T2\|SA | Sae1 | SAE1_MOUSE | 4 | 18.3768 | 19.4232 | 16.9327 | 16.8914 | 11.9547 | 16.4212 | 18.2442 | 1.2505 | 15.0891 | 2.7246 | 0.8271 | 1.42E-01 | 1.85E-01 |
| sp\|Q01405\|SC | Sec23a | SC23A_MOUSI | 4 | 16.5452 | 17.6438 | 17.4953 | 19.6071 | 14.9938 | 13.7148 | 17.2281 | 0.5961 | 16.1052 | 3.0994 | 0.9348 | 5.71E-01 | 6.12E-01 |
| sp\|Q9EQU5\|SI | Set | SET_MOUSE P | 4 | 27.3039 | 16.9155 | 23.2779 | 15.1653 | 9.49891 | 7.83856 | 22.4991 | 5.2378 | 10.8343 | 3.8416 | 0.4815 | 3.59E-02 | 5.54E-02 |
| sp\|Q8BH59\|CI | Slc25a12 | CMC1_MOUSE | 4 | 24.6663 | 31.7137 | 25.612 | 6.67988 | 5.12889 | 6.19932 | 27.3307 | 3.8252 | 6.0027 | 0.7940 | 0.2196 | 6.98E-04 | 3.91E-03 |
| sp\|P97496\|SM | Smarcc1 | SMRC1_MOUS | 4 | 21.9949 | 23.0729 | 23.8923 | 12.948 | 9.42212 | 8.66976 | 22.9867 | 0.9516 | 10.3466 | 2.2840 | 0.4501 | 9.01E-04 | 4.52E-03 |
| sp\|P08228\|SO | Sod1 | SODC_MOUSE | 4 | 29.395 | 15.0221 | 26.2878 | 13.2935 | 8.04495 | 7.95663 | 23.5683 | 7.5625 | 9.7650 | 3.0561 | 0.4143 | 4.28E-02 | 6.44E-02 |
| sp\|P62869\|EL | Tceb2 | ELOB_MOUSE | 4 | 22.2766 | 24.4331 | 14.9644 | 15.0673 | 14.9633 | 8.29527 | 20.5580 | 4.9628 | 12.7753 | 3.8802 | 0.6214 | 9.91E-02 | 1.34E-01 |
| sp\|Q9JHJ0\|TM | Tmod3 | TMOD3_MOU | 4 | 16.3487 | 5.96384 | 14.8228 | 20.7281 | 26.6791 | 15.4575 | 12.3784 | 5.6074 | 20.9549 | 5.6142 | 1.6929 | 1.35E-01 | 1.76E-01 |
| sp\|Q7TMM9\|T | Tubb2a | TBB2A_MOUSI | 4 | 21.5299 | 24.3858 | 22.8806 | 10.4122 | 8.0925 | 12.699 | 22.9321 | 1.4286 | 10.4012 | 2.3033 | 0.4536 | 1.32E-03 | 5.35E-03 |
| sp\|Q9ERD7\|TE | Tubb3 | TBB3_MOUSE | 4 | 28.516 | 28.2878 | 29.8311 | 4.47384 | 3.52837 | 5.36288 | 28.8783 | 0.8330 | 4.4550 | 0.9174 | 0.1543 | 4.39E-06 | 8.20E-04 |
| sp\|Q9JMH6\|TI | Txnrd1 | TRXR1_MOUSI | 4 | 25.2591 | 28.0196 | 25.7715 | 8.00469 | 6.37298 | 6.57212 | 26.3501 | 1.4684 | 6.9833 | 0.8902 | 0.2650 | 4.05E-05 | 1.19E-03 |
| sp\|P68037\|UB | Ube2l3 | UB2L3_MOUSI | 4 | 25.1474 | 16.7664 | 20.79 | 15.2949 | 9.35732 | 12.6439 | 20.9013 | 4.1916 | 12.4320 | 2.9745 | 0.5948 | 4.62E-02 | 6.90E-02 |
| sp\|Q9Z1Z0\|US | Uso1 | USO1_MOUSE | 4 | 20.2963 | 23.6452 | 22.4868 | 12.4661 | 10.7386 | 10.3671 | 22.1428 | 1.7008 | 11.1906 | 1.1201 | 0.5054 | 7.39E-04 | 4.08E-03 |
| sp\|Q80TB8\|VA | Vat1l | VAT1L_MOUSI | 4 | 30.897 | 25.6693 | 35.6798 | 2.40325 | 2.32798 | 3.02275 | 30.7487 | 5.0069 | 2.5847 | 0.3813 | 0.0841 | 6.29E-04 | 3.71E-03 |
| sp\|P46467\|VP | Vps4b | VPS4B_MOUSI | 4 | 22.2684 | 24.6984 | 25.3349 | 11.5654 | 8.82707 | 7.30583 | 24.1006 | 1.6183 | 9.2328 | 2.1586 | 0.3831 | 6.73E-04 | 3.87E-03 |
| sp\|P62259\|14 | Ywhae | 1433E_MOUSE | 4 | 17.2587 | 15.7726 | 24.7871 | 16.4596 | 11.1839 | 14.478 | 19.2728 | 4.8330 | 14.0605 | 2.6328 | 0.7296 | 1.76E-01 | 2.22E-01 |
| sp\|P68510\|14 | Ywhah | 1433F_MOUSE | 4 | 20.8456 | 17.8671 | 21.4676 | 12.7696 | 10.6583 | 16.3918 | 20.0601 | 1.9245 | 13.2732 | 2.8997 | 0.6617 | 2.78E-02 | 4.50E-02 |
| sp\|Q5SWU9\|A | Acaca | ACACA_MOUS | 3 | 19.3995 | 26.4435 | 21.1335 | 10.2947 | 8.42485 | 14.304 | 22.3255 | 3.6702 | 11.0079 | 3.0038 | 0.4931 | 1.45E-02 | 2.67E-02 |
| sp\|P50544\|AC | Acadvl | ACADV_MOUS | 3 | 16.8823 | 19.6163 | 19.3772 | 13.3762 | 11.1888 | 19.5593 | 18.6253 | 1.5142 | 14.7081 | 4.3413 | 0.7897 | 2.14E-01 | 2.65E-01 |
| sp\|Q9JIX8\|ACI | Acin1 | ACINU_MOUSI | 3 | 17.8862 | 15.2342 | 12.0638 | 13.9396 | 27.7805 | 13.0956 | 15.0614 | 2.9150 | 18.2719 | 8.2455 | 1.2132 | 5.59E-01 | 6.01E-01 |
| sp\|Q9WTP6\|K | Ak2 | KAD2_MOUSE | 3 | 21.2384 | 22.784 | 23.8252 | 9.43448 | 5.17253 | 17.5453 | 22.6159 | 1.3016 | 10.7174 | 6.2854 | 0.4739 | 3.26E-02 | 5.12E-02 |
| sp\|P21300\|AL | Akr1b7 | ALD1_MOUSE | 3 | 22.389 | 23.0164 | 29.1751 | 11.3099 | 7.79383 | 6.31575 | 24.8602 | 3.7500 | 8.4732 | 2.5654 | 0.3408 | 3.35E-03 | 9.27E-03 |
| sp\|Q8K009\|AL | Aldh1l2 | AL1L2_MOUSE | 3 | 24.2 | 25.118 | 27.0891 | 10.5933 | 7.10953 | 5.89006 | 25.4690 | 1.4762 | 7.8643 | 2.4408 | 0.3088 | 4.34E-04 | 3.05E-03 |
| sp\|O35381\|AN | Anp32a | AN32A_MOUS | 3 | 32.6035 | 18.877 | 25.182 | 9.19917 | 7.01248 | 7.12579 | 25.5542 | 6.8708 | 7.7791 | 1.2311 | 0.3044 | 1.16E-02 | 2.24E-02 |
| sp\|P17427\|AP | Ap2a2 | AP2A2_MOUS | 3 | 16.742 | 17.5908 | 22.0456 | 17.496 | 16.0261 | 10.0996 | 18.7928 | 2.8488 | 14.5406 | 3.9156 | 0.7737 | 2.03E-01 | 2.53E-01 |
| sp\|Q9DBG3\|A | Ap2b1 | AP2B1_MOUSI | 3 | 16.6954 | 19.1216 | 14.8494 | 18.6396 | 18.2695 | 12.4245 | 16.8888 | 2.1427 | 16.4445 | 3.4864 | 0.9737 | 8.60E-01 | 8.83E-01 |
| sp\|P28352\|AP | Apex1 | APEX1_MOUSI | 3 | 19.3234 | 18.7674 | 28.8488 | 9.67296 | 8.73704 | 14.6504 | 22.3132 | 5.6668 | 11.0201 | 3.1785 | 0.4939 | 3.95E-02 | 6.01E-02 |
| sp\|O35841\|AF | Api5 | API5_MOUSE | 3 | 24.6932 | 20.0852 | 27.6159 | 11.7792 | 8.12829 | 7.69819 | 24.1314 | 3.7966 | 9.2019 | 2.2423 | 0.3813 | 4.22E-03 | 1.08E-02 |
| sp\|P08030\|AP | Aprt | APT_MOUSE A | 3 | 29.3763 | 22.507 | 36.7488 | 3.38877 | 3.46524 | 4.51391 | 29.5440 | 7.1224 | 3.7893 | 0.6287 | 0.1283 | 3.36E-03 | 9.29E-03 |

| | | | | | | | | | | | | | | | |
|---|---|---|---|---|---|---|---|---|---|---|---|---|---|---|---|
| sp\|P16460\|AS | Ass1 | ASSY_MOUSE | 3 | 21.1249 | 22.5551 | 20.5905 | 15.1473 | 9.60898 | 10.9732 | 21.4235 | 1.0158 | 11.9098 | 2.8855 | 0.5559 | 5.74E-03 | 1.36E-02 |
| sp\|Q925I1\|AT | Atad3 | ATAD3_MOUS | 3 | 21.5288 | 27.673 | 18.0635 | 10.6385 | 9.16206 | 12.9341 | 22.4218 | 4.8666 | 10.9116 | 1.9008 | 0.4867 | 1.88E-02 | 3.32E-02 |
| tr\|G5E829\|G5 | Atp2b1 | G5E829_MOU | 3 | 21.6281 | 28.0791 | 28.7494 | 7.32843 | 6.40091 | 7.81406 | 26.1522 | 3.9323 | 7.1811 | 0.7180 | 0.2746 | 1.19E-03 | 5.09E-03 |
| sp\|Q91VR2\|A | Atp5c1 | ATPG_MOUSE | 3 | 22.1312 | 25.0503 | 18.5082 | 13.4942 | 12.0582 | 8.75794 | 21.8966 | 3.2774 | 11.4368 | 2.4285 | 0.5223 | 1.13E-02 | 2.22E-02 |
| sp\|Q9DB20\|A | Atp5o | ATPO_MOUSE | 3 | 18.9437 | 19.2498 | 17.691 | 20.3567 | 14.2007 | 9.55814 | 18.6282 | 0.8259 | 14.7052 | 5.4169 | 0.7894 | 2.83E-01 | 3.41E-01 |
| sp\|P62814\|VA | Atp6v1b2 | VATB2_MOUS | 3 | 20.8289 | 23.0653 | 24.6113 | 13.0045 | 8.8399 | 9.65016 | 22.8352 | 1.9017 | 10.4982 | 2.2080 | 0.4597 | 1.84E-03 | 6.20E-03 |
| sp\|Q9Z1G3\|V | Atp6v1c1 | VATC1_MOUS | 3 | 23.1567 | 24.2558 | 25.3485 | 9.43631 | 10.5673 | 7.23544 | 24.2537 | 1.0959 | 9.0797 | 1.6943 | 0.3744 | 2.01E-04 | 2.09E-03 |
| sp\|Q61335\|B | Bcap31 | BAP31_MOUS | 3 | 23.1176 | 19.094 | 22.7241 | 15.0048 | 10.4404 | 9.61916 | 21.6452 | 2.2182 | 11.6881 | 2.9015 | 0.5400 | 9.16E-03 | 1.88E-02 |
| sp\|Q8R016\|BL | Blmh | BLMH_MOUSE | 3 | 13.7346 | 11.4393 | 13.2491 | 19.4077 | 17.668 | 24.5013 | 12.8077 | 1.2096 | 20.5257 | 3.5512 | 1.6026 | 2.35E-02 | 3.95E-02 |
| sp\|P18572\|BA | Bsg | BASI_MOUSE | 3 | 28.2968 | 29.6829 | 32.0901 | 2.66523 | 3.27353 | 3.99149 | 30.0233 | 1.9194 | 3.3101 | 0.6639 | 0.1103 | 2.20E-05 | 1.11E-03 |
| sp\|O35350\|C | Capn1 | CAN1_MOUSE | 3 | 16.4967 | 17.0388 | 22.4721 | 13.0264 | 14.1441 | 16.8219 | 18.6692 | 3.3045 | 14.6641 | 1.9505 | 0.7855 | 1.45E-01 | 1.88E-01 |
| sp\|P47754\|CA | Capza2 | CAZA2_MOUS | 3 | 18.6995 | 15.47 | 23.8664 | 11.7157 | 18.242 | 12.0064 | 19.3453 | 4.2353 | 13.9880 | 3.6869 | 0.7231 | 1.74E-01 | 2.20E-01 |
| sp\|P48758\|CB | Cbr1 | CBR1_MOUSE | 3 | 7.65965 | 7.91785 | 7.13576 | 32.3968 | 25.5064 | 19.3835 | 7.5711 | 0.3985 | 25.7622 | 6.5104 | 3.4027 | 8.46E-03 | 1.79E-02 |
| sp\|Q8CH18\|C | Ccar1 | CCAR1_MOUS | 3 | 27.7252 | 29.0598 | 25.4603 | 7.00837 | 4.99647 | 5.74994 | 27.4151 | 1.8197 | 5.9183 | 1.0165 | 0.2159 | 5.77E-05 | 1.39E-03 |
| sp\|Q9QXK3\|C | Copg2 | COPG2_MOUS | 3 | 21.0528 | 27.7439 | 20.5615 | 12.3672 | 10.5033 | 7.77133 | 23.1194 | 4.0125 | 10.2139 | 2.3116 | 0.4418 | 8.48E-03 | 1.80E-02 |
| sp\|O88712\|CT | Ctbp1 | CTBP1_MOUS | 3 | 20.5875 | 24.5507 | 21.2486 | 13.315 | 10.1352 | 10.1631 | 22.1289 | 2.1232 | 11.2044 | 1.8279 | 0.5063 | 2.51E-03 | 7.57E-03 |
| sp\|Q9WTX6\|C | Cul1 | CUL1_MOUSE | 3 | 18.9675 | 24.0491 | 19.9172 | 13.2579 | 12.6656 | 11.1427 | 20.9779 | 2.7018 | 12.3554 | 1.0912 | 0.5890 | 6.86E-03 | 1.54E-02 |
| sp\|Q9QYJ3\|D | Dnajb1 | DNJB1_MOUS | 3 | 25.3874 | 19.7736 | 27.6746 | 9.91934 | 8.90115 | 8.34396 | 24.2785 | 4.0655 | 9.0548 | 0.7989 | 0.3730 | 3.13E-03 | 8.83E-03 |
| sp\|Q9QYI3\|D | Dnajc7 | DNJC7_MOUS | 3 | 26.9619 | 27.5005 | 28.3898 | 4.95529 | 6.30349 | 5.88895 | 27.6174 | 0.7211 | 5.7159 | 0.6906 | 0.2070 | 2.87E-06 | 7.74E-04 |
| sp\|Q99KK7\|D | Dpp3 | DPP3_MOUSE | 3 | 22.113 | 19.4421 | 27.8234 | 11.7787 | 8.57987 | 10.2629 | 23.1262 | 4.2815 | 10.2072 | 1.6001 | 0.4414 | 8.07E-03 | 1.74E-02 |
| sp\|O88487\|D | Dync1i2 | DC1I2_MOUSE | 3 | 19.224 | 20.1021 | 17.8416 | 18.5293 | 11.8456 | 12.4573 | 19.0559 | 1.1396 | 14.2774 | 3.6949 | 0.7492 | 9.90E-02 | 1.34E-01 |
| sp\|P62631\|E | Eef1a2 | EF1A2_MOUS | 3 | 34.9218 | 27.4736 | 28.1271 | 3.41942 | 2.59093 | 3.46707 | 30.1742 | 4.1245 | 3.1591 | 0.4927 | 0.1047 | 3.54E-04 | 2.70E-03 |
| sp\|Q9WVK4\|E | Ehd1 | EHD1_MOUSE | 3 | 16.9402 | 15.3605 | 20.0671 | 17.5733 | 15.0706 | 14.9883 | 17.4559 | 2.3953 | 15.8774 | 1.4693 | 0.9096 | 3.86E-01 | 4.44E-01 |
| sp\|Q80XI3\|IF | Eif4g3 | IF4G3_MOUSE | 3 | 19.9871 | 25.1338 | 20.5532 | 6.3531 | 13.8423 | 14.1305 | 21.8914 | 2.8223 | 11.4420 | 4.4094 | 0.5227 | 2.59E-02 | 4.27E-02 |
| sp\|P63242\|IF | Eif5a | IF5A1_MOUSE | 3 | 24.0918 | 22.0423 | 23.1357 | 13.4742 | 8.87996 | 8.37599 | 23.0899 | 1.0255 | 10.2434 | 2.8093 | 0.4436 | 1.74E-03 | 6.01E-03 |
| sp\|P21995\|E | Emb | EMB_MOUSE | 3 | 34.9736 | 25.4358 | 30.8298 | 2.15875 | 3.0562 | 3.54595 | 30.4131 | 4.7825 | 2.9203 | 0.7035 | 0.0960 | 5.96E-04 | 3.60E-03 |
| sp\|O70318\|E4 | Epb41l2 | E41L2_MOUSE | 3 | 17.3443 | 16.946 | 18.6493 | 21.5813 | 14.6419 | 10.8372 | 17.6465 | 0.8910 | 15.6868 | 5.4477 | 0.8889 | 5.72E-01 | 6.12E-01 |
| sp\|Q9DCW4\|E | Etfb | ETFB_MOUSE | 3 | 20.8035 | 22.7123 | 32.9291 | 8.77234 | 7.71653 | 7.06619 | 25.4816 | 6.5199 | 7.8517 | 0.8611 | 0.3081 | 9.71E-03 | 1.97E-02 |
| sp\|Q922J9\|FA | Far1 | FACR1_MOUS | 3 | 21.82 | 29.5431 | 24.6334 | 4.62183 | 4.10066 | 15.2811 | 25.3322 | 3.9087 | 8.0012 | 6.3100 | 0.3159 | 1.55E-02 | 2.82E-02 |
| sp\|P35550\|FB | Fbl | FBRL_MOUSE | 3 | 16.9746 | 18.4769 | 15.0576 | 15.8983 | 17.6245 | 15.9681 | 16.8364 | 1.7138 | 16.4970 | 0.9771 | 0.9798 | 7.81E-01 | 8.09E-01 |
| sp\|Q62446\|FK | Fkbp3 | FKBP3_MOUS | 3 | 28.4428 | 21.1955 | 24.849 | 7.41355 | 8.59404 | 9.50512 | 24.8291 | 3.6237 | 8.5042 | 1.0487 | 0.3425 | 1.69E-03 | 5.96E-03 |
| sp\|P63094\|G | Gnas | GNAS2_MOUS | 3 | 18.1396 | 19.1134 | 18.9266 | 18.7405 | 15.0138 | 10.0661 | 18.7265 | 0.5168 | 14.6068 | 4.3515 | 0.7800 | 1.79E-01 | 2.25E-01 |
| sp\|P62874\|G | Gnb1 | GBB1_MOUSE | 3 | 21.6625 | 21.9927 | 22.0481 | 14.1454 | 11.4423 | 8.70901 | 21.9011 | 0.2085 | 11.4322 | 2.7182 | 0.5220 | 2.65E-03 | 7.86E-03 |
| sp\|Q99LP6\|G | Grpel1 | GRPE1_MOUS | 3 | 25.9179 | 21.3255 | 21.4201 | 12.0996 | 8.77814 | 10.4587 | 22.8878 | 2.6245 | 10.4455 | 1.6608 | 0.4564 | 2.27E-03 | 7.06E-03 |
| sp\|P19157\|GS | Gstp1 | GSTP1_MOUS | 3 | 21.5448 | 29.3189 | 28.6516 | 9.47822 | 6.76996 | 4.2365 | 26.5051 | 4.3087 | 6.8282 | 2.6213 | 0.2576 | 2.50E-03 | 7.57E-03 |
| sp\|Q61191\|H | Hcfc1 | HCFC1_MOUS | 3 | 23.4823 | 32.1755 | 22.3141 | 10.1606 | 6.49644 | 5.37106 | 25.9906 | 5.3880 | 7.3427 | 2.5044 | 0.2825 | 5.56E-03 | 1.34E-02 |
| sp\|P43274\|H1 | Hist1h1e | H14_MOUSE | 3 | 8.47786 | 13.9781 | 5.32921 | 37.6674 | 24.8405 | 9.70689 | 9.2617 | 4.3774 | 24.0716 | 13.9961 | 2.5990 | 1.55E-01 | 1.99E-01 |
| sp\|Q8BG05\|R | Hnrnpa3 | ROA3_MOUSE | 3 | 22.6562 | 16.9482 | 17.7709 | 11.2999 | 16.9332 | 14.3915 | 19.1251 | 3.0856 | 14.2082 | 2.8211 | 0.7429 | 1.11E-01 | 1.50E-01 |
| sp\|Q9Z204\|H | Hnrnpc | HNRPC_MOUS | 3 | 16.7062 | 20.1123 | 14.4034 | 22.6098 | 14.7873 | 11.381 | 17.0740 | 2.8722 | 16.2594 | 5.7573 | 0.9523 | 8.37E-01 | 8.61E-01 |
| sp\|Q60668\|H | Hnrnpd | HNRPD_MOUS | 3 | 20.628 | 22.8677 | 15.3843 | 17.9129 | 13.2036 | 10.0035 | 19.6267 | 3.8409 | 13.7067 | 3.9786 | 0.6984 | 1.37E-01 | 1.79E-01 |
| sp\|Q9Z2X1\|H | Hnrnpf | HNRPF_MOUS | 3 | 18.5945 | 21.3115 | 14.6594 | 17.7874 | 15.7275 | 11.9196 | 18.1885 | 3.3446 | 15.1448 | 2.9770 | 0.8327 | 3.04E-01 | 3.64E-01 |
| sp\|P17879\|HS | Hspa1b | HS71B_MOUS | 3 | 11.2423 | 17.4201 | 17.0766 | 20.2975 | 14.5244 | 19.439 | 15.2463 | 3.4718 | 18.0870 | 3.1150 | 1.1863 | 3.51E-01 | 4.11E-01 |
| sp\|Q8VI75\|IP | Ipo4 | IPO4_MOUSE | 3 | 22.5194 | 23.3725 | 24.3687 | 13.7013 | 6.24287 | 9.79533 | 23.4202 | 0.9256 | 9.9132 | 3.7306 | 0.4233 | 3.68E-03 | 9.82E-03 |
| sp\|Q6ZQ88\|K | Kdm1a | KDM1A_MOU | 3 | 24.614 | 25.7053 | 25.5654 | 8.17095 | 5.92587 | 10.0185 | 25.2949 | 0.5938 | 8.0384 | 2.0495 | 0.3178 | 1.51E-04 | 1.87E-03 |
| sp\|Q61792\|LA | Lasp1 | LASP1_MOUSE | 3 | 19.8202 | 16.6026 | 13.2223 | 21.5404 | 15.9933 | 12.8212 | 16.5484 | 3.2993 | 16.7850 | 4.4132 | 1.0143 | 9.44E-01 | 9.55E-01 |
| sp\|Q3U9G9\|L | Lbr | LBR_MOUSE | 3 | 23.8525 | 29.6718 | 22.5681 | 8.66709 | 7.65938 | 7.58118 | 25.3641 | 3.7854 | 7.9692 | 0.6056 | 0.3142 | 1.42E-03 | 5.48E-03 |
| sp\|Q9D0F3\|L | Lman1 | LMAN1_MOUS | 3 | 19.1249 | 22.0402 | 20.0364 | 16.4007 | 11.4189 | 10.979 | 20.4005 | 1.4914 | 12.9329 | 3.0113 | 0.6339 | 1.83E-02 | 3.25E-02 |
| sp\|Q91WK0\|L | Lrrfip2 | LRRF2_MOUSE | 3 | 22.794 | 13.5372 | 21.3563 | 13.8881 | 17.2135 | 11.2109 | 19.2292 | 4.9815 | 14.1042 | 3.0071 | 0.7335 | 2.02E-01 | 2.52E-01 |
| sp\|Q7TNC4\|L | Luc7l2 | LC7L2_MOUSE | 3 | 22.078 | 23.2313 | 17.5672 | 13.5407 | 14.6345 | 8.94831 | 20.9588 | 2.9933 | 12.3745 | 3.0172 | 0.5904 | 2.49E-02 | 4.14E-02 |
| sp\|P97820\|M | Map4k4 | M4K4_MOUSE | 3 | 26.3893 | 28.5725 | 26.2651 | 7.83674 | 6.3266 | 4.60981 | 27.0756 | 1.2978 | 6.2577 | 1.6146 | 0.2311 | 6.39E-05 | 1.44E-03 |
| sp\|P28667\|M | Marcksl1 | MRP_MOUSE | 3 | 32.7245 | 27.9935 | 26.504 | 4.50653 | 3.57041 | 4.70107 | 29.0740 | 3.2480 | 4.2593 | 0.6045 | 0.1465 | 2.01E-04 | 2.09E-03 |
| sp\|Q07646\|M | Mest | MEST_MOUSE | 3 | 23.0745 | 34.9058 | 30.8514 | 1.38809 | 4.27206 | 5.50816 | 29.6106 | 6.0125 | 3.7228 | 2.1142 | 0.1257 | 2.15E-03 | 6.84E-03 |
| sp\|Q8BI84\|M | Mia3 | MIA3_MOUSE | 3 | 27.1916 | 33.0209 | 22.3214 | 6.1107 | 4.9243 | 6.43118 | 27.5113 | 5.3569 | 5.8221 | 0.7938 | 0.2116 | 2.27E-03 | 7.06E-03 |
| sp\|Q9QZQ1\|A | Mllt4 | AFAD_MOUSE | 3 | 28.3111 | 27.6309 | 32.9785 | 2.29828 | 4.32063 | 4.46063 | 29.6402 | 2.9110 | 3.6932 | 1.2100 | 0.1246 | 1.41E-04 | 1.83E-03 |
| sp\|Q9CQ65\|M | Mtap | MTAP_MOUSE | 3 | 20.9098 | 22.7802 | 21.864 | 10.6975 | 10.8471 | 12.9014 | 21.8513 | 0.9353 | 11.4820 | 1.2315 | 0.5255 | 3.14E-04 | 2.57E-03 |
| sp\|Q6URW6\|M | Myh14 | MYH14_MOUS | 3 | 12.3107 | 9.45242 | 18.7414 | 17.0968 | 33.1417 | 9.257 | 13.5015 | 4.7576 | 19.8318 | 12.1750 | 1.4689 | 4.49E-01 | 5.00E-01 |
| sp\|P70670\|N | Naca | NACAM_MOU | 3 | 32.8148 | 22.6835 | 25.9521 | 7.60608 | 5.42651 | 5.517 | 27.1501 | 5.1708 | 6.1832 | 1.2331 | 0.2277 | 2.40E-03 | 7.35E-03 |
| tr\|D3YVV7\|D3 | Nova2 | D3YVV7_MOU | 3 | 34.1077 | 24.0741 | 36.0262 | 1.96489 | 0 | 3.82715 | 31.4027 | 6.4188 | 1.9307 | 1.9138 | 0.0615 | 1.59E-03 | 5.87E-03 |
| tr\|B9EJ54\|B9E | Nup205 | B9EJ54_MOUS | 3 | 21.4737 | 27.3139 | 24.2153 | 10.8788 | 7.73958 | 8.37873 | 24.3343 | 2.9219 | 8.9990 | 1.6590 | 0.3698 | 1.39E-03 | 5.48E-03 |
| sp\|Q8BJ71\|N | Nup93 | NUP93_MOUS | 3 | 23.2147 | 22.9756 | 24.9095 | 8.72329 | 10.0334 | 10.1435 | 23.6999 | 1.0543 | 9.6334 | 0.7901 | 0.4065 | 5.03E-05 | 1.33E-03 |
| sp\|Q60715\|P4 | P4ha1 | P4HA1_MOUS | 3 | 11.3506 | 11.5263 | 12.1348 | 26.2308 | 23.2467 | 15.5109 | 11.6706 | 0.4115 | 21.6628 | 5.5327 | 1.8562 | 3.55E-02 | 5.49E-02 |
| sp\|Q9WVE8\|P | Pacsin2 | PACN2_MOUS | 3 | 26.367 | 20.5291 | 26.3214 | 11.3586 | 6.91863 | 8.50529 | 24.4058 | 3.3574 | 8.9275 | 2.2499 | 0.3658 | 2.68E-03 | 7.92E-03 |
| sp\|P63005\|LIS | Pafah1b1 | LIS1_MOUSE | 3 | 19.9715 | 15.7663 | 23.3666 | 19.111 | 10.5829 | 11.2018 | 19.7015 | 3.8073 | 13.6319 | 4.7551 | 0.6919 | 1.59E-01 | 2.04E-01 |

| | | | | | | | | | | | | | | | | |
|---|---|---|---|---|---|---|---|---|---|---|---|---|---|---|---|---|
| sp\|Q3UHX2\|H | Pdap1 | HAP28_MOUS | 3 | 26.0001 | 19.6368 | 20.8215 | 6.08692 | 10.8641 | 16.5907 | 22.1528 | 3.3841 | 11.1806 | 5.2590 | 0.5047 | 3.84E-02 | 5.88E-02 |
| sp\|P70296\|PE | Pebp1 | PEBP1_MOUSI | 3 | 26.5134 | 25.3862 | 24.4825 | 10.4184 | 6.40387 | 6.7956 | 25.4607 | 1.0175 | 7.8726 | 2.2134 | 0.3092 | 2.35E-04 | 2.29E-03 |
| sp\|P47857\|K6 | Pfkm | K6PF_MOUSE | 3 | 25.7857 | 24.2806 | 30.9544 | 6.82545 | 5.69619 | 6.45774 | 27.0069 | 3.5005 | 6.3265 | 0.5760 | 0.2343 | 5.41E-04 | 3.44E-03 |
| sp\|Q8K411\|PF | Pitrm1 | PREP_MOUSE | 3 | 25.8315 | 26.6613 | 31.655 | 5.5588 | 4.15621 | 6.13714 | 28.0493 | 3.1501 | 5.2841 | 1.0186 | 0.1884 | 2.85E-04 | 2.45E-03 |
| sp\|P27612\|PL | Plaa | PLAP_MOUSE | 3 | 18.543 | 21.0484 | 21.9429 | 15.8188 | 12.1488 | 10.4981 | 20.5114 | 1.7624 | 12.8219 | 2.7235 | 0.6251 | 1.48E-02 | 2.72E-02 |
| sp\|Q61074\|PF | Ppm1g | PPM1G_MOUS | 3 | 22.7463 | 23.2828 | 25.0681 | 9.08088 | 9.78804 | 10.034 | 23.6991 | 1.2156 | 9.6343 | 0.4948 | 0.4065 | 4.96E-05 | 1.33E-03 |
| sp\|Q6P1F6\|2A | Ppp2r2a | 2ABA_MOUSE | 3 | 21.2636 | 24.8504 | 24.8897 | 10.2659 | 8.66133 | 10.0691 | 23.6679 | 2.0823 | 9.6654 | 0.8751 | 0.4084 | 4.26E-04 | 3.02E-03 |
| sp\|P58389\|PT | Ppp2r4 | PTPA_MOUSE | 3 | 24.0209 | 17.5784 | 27.5903 | 12.3386 | 9.99519 | 8.47659 | 23.0632 | 5.0742 | 10.2701 | 1.9456 | 0.4453 | 1.51E-02 | 2.76E-02 |
| tr\|Q91V89\|Q9 | Ppp2r5d | Q91V89_MOU | 3 | 26.0874 | 19.2751 | 27.8718 | 9.23866 | 9.72192 | 7.80522 | 24.4114 | 4.5368 | 8.9219 | 0.9968 | 0.3655 | 4.46E-03 | 1.13E-02 |
| sp\|P05132\|KA | Prkaca | KAPCA_MOUS | 3 | 21.3064 | 21.4636 | 21.5859 | 13.7624 | 12.2278 | 9.65393 | 21.4520 | 0.1401 | 11.8814 | 2.0760 | 0.5539 | 1.35E-03 | 5.43E-03 |
| sp\|Q91YR7\|PF | Prpf6 | PRP6_MOUSE | 3 | 17.2627 | 25.5491 | 17.1902 | 11.5327 | 16.0234 | 12.4418 | 20.0007 | 4.8052 | 13.3326 | 2.3742 | 0.6666 | 9.75E-02 | 1.33E-01 |
| sp\|O09061\|PS | Psmb1 | PSB1_MOUSE | 3 | 21.9221 | 19.9119 | 25.4211 | 8.78841 | 8.70287 | 15.2536 | 22.4184 | 2.7879 | 10.9150 | 3.7576 | 0.4869 | 1.31E-02 | 2.47E-02 |
| sp\|P70195\|PS | Psmb7 | PSB7_MOUSE | 3 | 24.127 | 20.4142 | 27.2638 | 9.57496 | 7.32853 | 11.2915 | 23.9350 | 3.4288 | 9.3983 | 1.9874 | 0.3927 | 3.15E-03 | 8.85E-03 |
| sp\|Q9D8W5\|F | Psmd12 | PSD12_MOUSI | 3 | 24.1261 | 26.7534 | 22.5119 | 11.2559 | 8.18949 | 7.16327 | 24.4638 | 2.1408 | 8.8696 | 2.1294 | 0.3626 | 8.64E-04 | 4.44E-03 |
| sp\|Q99JI4\|PSN | Psmd6 | PSMD6_MOUS | 3 | 23.7206 | 24.0461 | 21.3436 | 12.5694 | 9.22736 | 9.09295 | 23.0368 | 1.4753 | 10.2966 | 1.9695 | 0.4470 | 8.56E-04 | 4.41E-03 |
| sp\|Q8R326\|PS | Pspc1 | PSPC1_MOUSI | 3 | 21.9936 | 18.1285 | 18.8993 | 15.2514 | 17.0361 | 8.69108 | 19.6738 | 2.0456 | 13.6595 | 4.3944 | 0.6943 | 9.81E-02 | 1.34E-01 |
| sp\|Q99LS3\|SE | Psph | SERB_MOUSE | 3 | 27.2126 | 19.4961 | 31.2756 | 8.30216 | 6.73329 | 6.98024 | 25.9948 | 5.9834 | 7.3386 | 0.8436 | 0.2823 | 5.90E-03 | 1.38E-02 |
| sp\|P61027\|RA | Rab10 | RAB10_MOUS | 3 | 20.8229 | 18.2014 | 22.1675 | 15.8607 | 11.3292 | 11.6184 | 20.3973 | 2.0170 | 12.9361 | 2.5369 | 0.6342 | 1.63E-02 | 2.93E-02 |
| sp\|P35279\|RA | Rab6a | RAB6A_MOUS | 3 | 21.2794 | 23.2485 | 23.5026 | 12.1826 | 9.57155 | 10.2153 | 22.6768 | 1.2169 | 10.6565 | 1.3603 | 0.4699 | 3.37E-04 | 2.68E-03 |
| sp\|P51150\|RA | Rab7a | RAB7A_MOUS | 3 | 21.1465 | 18.6375 | 24.9384 | 14.172 | 10.0099 | 11.0958 | 21.5741 | 3.1721 | 11.7592 | 2.1589 | 0.5451 | 1.14E-02 | 2.23E-02 |
| sp\|P62827\|RA | Ran | RAN_MOUSE ( | 3 | 25.791 | 21.1603 | 25.7973 | 13.075 | 7.53401 | 6.64238 | 24.2495 | 2.6754 | 9.0838 | 3.4851 | 0.3746 | 3.93E-03 | 1.03E-02 |
| sp\|P34022\|RA | Ranbp1 | RANG_MOUSE | 3 | 35.0922 | 24.3374 | 26.9859 | 5.42493 | 3.77284 | 4.38677 | 28.8052 | 5.6035 | 4.5282 | 0.8351 | 0.1572 | 1.76E-03 | 6.03E-03 |
| sp\|P62835\|RA | Rap1a | RAP1A_MOUS | 3 | 22.867 | 20.9628 | 26.5464 | 11.7116 | 8.78252 | 9.12976 | 23.4587 | 2.8384 | 9.8746 | 1.6003 | 0.4209 | 1.95E-03 | 6.48E-03 |
| sp\|Q8VH51\|RI | Rbm39 | RBM39_MOUS | 3 | 17.1887 | 20.6037 | 15.8761 | 14.1609 | 12.8474 | 19.3232 | 17.8895 | 2.4405 | 15.4438 | 3.4232 | 0.8633 | 3.71E-01 | 4.30E-01 |
| sp\|Q62159\|RH | Rhoc | RHOC_MOUSE | 3 | 14.8867 | 16.3006 | 18.1469 | 22.4673 | 14.6408 | 13.5578 | 16.4447 | 1.6349 | 16.8886 | 4.8615 | 1.0270 | 8.88E-01 | 9.05E-01 |
| sp\|Q9D706\|RI | Rpap3 | RPAP3_MOUS | 3 | 23.4073 | 27.7085 | 30.5588 | 5.99689 | 6.23013 | 6.09833 | 27.2249 | 3.6002 | 6.1085 | 0.1169 | 0.2244 | 5.30E-04 | 3.42E-03 |
| sp\|Q9CZM2\|R | Rpl15 | RL15_MOUSE | 3 | 16.5416 | 19.2359 | 16.6363 | 16.1272 | 19.0603 | 12.3988 | 17.4713 | 1.5290 | 15.8621 | 3.3387 | 0.9079 | 4.90E-01 | 5.39E-01 |
| sp\|O09167\|RL | Rpl21 | RL21_MOUSE | 3 | 14.7494 | 17.6673 | 10.329 | 22.8142 | 23.674 | 10.766 | 14.2486 | 3.6947 | 19.0847 | 7.2170 | 1.3394 | 3.60E-01 | 4.20E-01 |
| sp\|P67984\|RL | Rpl22 | RL22_MOUSE | 3 | 22.5 | 20.3785 | 17.9409 | 14.8534 | 14.1946 | 10.1326 | 20.2731 | 2.2814 | 13.0602 | 2.5567 | 0.6442 | 2.18E-02 | 3.73E-02 |
| sp\|P62830\|RL | Rpl23 | RL23_MOUSE | 3 | 17.7102 | 23.1357 | 14.3109 | 16.3203 | 17.899 | 10.624 | 18.3856 | 4.4510 | 14.9478 | 3.8268 | 0.8130 | 3.68E-01 | 4.27E-01 |
| sp\|Q8BP67\|RL | Rpl24 | RL24_MOUSE | 3 | 18.3041 | 18.3878 | 13.6898 | 19.8622 | 19.4886 | 10.2675 | 16.7939 | 2.6886 | 16.5394 | 5.4349 | 0.9848 | 9.46E-01 | 9.55E-01 |
| sp\|P61358\|RL | Rpl27 | RL27_MOUSE | 3 | 21.5981 | 23.8657 | 13.3093 | 16.9895 | 15.7453 | 8.49212 | 19.5910 | 5.5570 | 13.7423 | 4.5892 | 0.7015 | 2.33E-01 | 2.86E-01 |
| sp\|P14115\|RL | Rpl27a | RL27A_MOUSI | 3 | 20.3512 | 17.1862 | 14.0031 | 18.6767 | 17.6463 | 12.1365 | 17.1802 | 3.1741 | 16.1532 | 3.5165 | 0.9402 | 7.26E-01 | 7.58E-01 |
| sp\|P47915\|RL | Rpl29 | RL29_MOUSE | 3 | 17.6898 | 20.9929 | 12.4723 | 19.9036 | 19.5207 | 9.42062 | 17.0517 | 4.2960 | 16.2816 | 5.9449 | 0.9548 | 8.65E-01 | 8.86E-01 |
| tr\|Q6ZWZ6\|Q | Rps12 | Q6ZWZ6_MOL | 3 | 25.4322 | 26.7033 | 20.2807 | 12.3473 | 8.56121 | 6.6753 | 24.1387 | 3.4011 | 9.1946 | 2.8886 | 0.3809 | 4.39E-03 | 1.12E-02 |
| sp\|P62264\|RS | Rps14 | RS14_MOUSE | 3 | 18.4555 | 20.2366 | 14.0609 | 17.3501 | 18.9897 | 10.9073 | 17.5843 | 3.1787 | 15.7490 | 4.2725 | 0.8956 | 5.83E-01 | 6.23E-01 |
| sp\|P60867\|RS | Rps20 | RS20_MOUSE | 3 | 17.688 | 26.1785 | 18.1315 | 14.4549 | 14.2326 | 9.31446 | 20.6660 | 4.7791 | 12.6673 | 2.9058 | 0.6130 | 6.84E-02 | 9.72E-02 |
| sp\|P62267\|RS | Rps23 | RS23_MOUSE | 3 | 16.9382 | 21.7362 | 14.0567 | 18.7635 | 18.2379 | 10.2675 | 17.5770 | 3.8794 | 15.7563 | 4.7607 | 0.8964 | 6.35E-01 | 6.74E-01 |
| sp\|P97461\|RS | Rps5 | RS5_MOUSE 4 | 3 | 19.6994 | 22.6348 | 19.1838 | 16.1941 | 12.6294 | 9.6586 | 20.5060 | 1.8615 | 12.8274 | 3.2722 | 0.6255 | 2.42E-02 | 4.04E-02 |
| sp\|P70122\|SB | Sbds | SBDS_MOUSE | 3 | 23.0897 | 21.5706 | 23.1485 | 13.9325 | 9.38669 | 8.87201 | 22.6029 | 0.8945 | 10.7304 | 2.7850 | 0.4747 | 2.16E-03 | 6.84E-03 |
| sp\|P61620\|S6 | Sec61a1 | S61A1_MOUSI | 3 | 26.0251 | 22.9395 | 23.1854 | 10.1627 | 9.00632 | 8.68099 | 24.0500 | 1.7149 | 9.2833 | 0.7787 | 0.3860 | 1.70E-04 | 1.97E-03 |
| sp\|Q64213\|SF | Sf1 | SF01_MOUSE | 3 | 22.7625 | 22.7455 | 21.2436 | 9.92109 | 11.8908 | 11.4366 | 22.2505 | 0.8721 | 11.0828 | 1.0314 | 0.4981 | 1.38E-04 | 1.82E-03 |
| sp\|Q8BMD8\|S | Slc25a24 | SCMC1_MOUS | 3 | 17.4205 | 20.4266 | 19.36 | 17.8219 | 11.6576 | 13.3134 | 19.0690 | 1.5240 | 14.2643 | 3.1903 | 0.7480 | 7.82E-02 | 1.09E-01 |
| sp\|P27048\|RS | Snrpb | RSMB_MOUSE | 3 | 23.2636 | 21.8022 | 20.6737 | 12.6132 | 11.6309 | 10.0164 | 21.9132 | 1.2985 | 11.4202 | 1.3112 | 0.5212 | 5.96E-04 | 3.60E-03 |
| sp\|P09671\|SO | Sod2 | SODM_MOUSI | 3 | 27.7648 | 19.4412 | 25.2387 | 12.5779 | 8.02909 | 6.94827 | 24.1482 | 4.2676 | 9.1851 | 2.9875 | 0.3804 | 7.62E-03 | 1.67E-02 |
| sp\|Q9DBG7\|SI | Srpr | SRPR_MOUSE | 3 | 22.6848 | 20.616 | 25.4406 | 12.6887 | 9.0107 | 9.55924 | 22.9138 | 2.4204 | 10.4195 | 1.9842 | 0.4547 | 2.30E-03 | 7.09E-03 |
| sp\|Q99MR6\|S | Srrt | SRRT_MOUSE | 3 | 19.7208 | 24.5966 | 20.9194 | 14.4234 | 12.5785 | 7.76143 | 21.7456 | 2.5407 | 11.5878 | 3.4397 | 0.5329 | 1.47E-02 | 2.71E-02 |
| sp\|Q9Z1Z2\|ST | Strap | STRAP_MOUSI | 3 | 14.0461 | 14.6836 | 30.0148 | 7.58332 | 6.10223 | 27.5699 | 19.5815 | 9.0411 | 13.7518 | 11.9897 | 0.7023 | 5.38E-01 | 5.83E-01 |
| sp\|Q9WUM5\| | Suclg1 | SUCA_MOUSE | 3 | 20.0637 | 23.1827 | 18.511 | 16.4864 | 12.7571 | 8.99913 | 20.5858 | 2.3792 | 12.7475 | 3.7436 | 0.6192 | 3.76E-02 | 5.76E-02 |
| sp\|Q80X82\|SY | Sympk | SYMPK_MOUS | 3 | 25.5033 | 27.9563 | 22.3841 | 5.12269 | 10.2497 | 8.78397 | 25.2812 | 2.7927 | 8.0521 | 2.6407 | 0.3185 | 1.48E-03 | 5.61E-03 |
| sp\|Q9CY27\|TE | Tecr | TECR_MOUSE | 3 | 20.6467 | 24.0841 | 20.7216 | 12.9378 | 11 | 10.6097 | 21.8175 | 1.9633 | 11.5158 | 1.2468 | 0.5278 | 1.55E-03 | 5.79E-03 |
| sp\|P39447\|ZO | Tjp1 | ZO1_MOUSE T | 3 | 26.0565 | 19.5952 | 32.7133 | 6.42332 | 7.08762 | 8.124 | 26.1217 | 6.5593 | 7.2116 | 0.8571 | 0.2761 | 7.75E-03 | 1.69E-02 |
| sp\|Q8BFY9\|TN | Tnpo1 | TNPO1_MOUS | 3 | 26.7764 | 24.9023 | 28.5138 | 8.14752 | 5.8764 | 5.7836 | 26.7308 | 1.8062 | 6.6025 | 1.3388 | 0.2470 | 1.01E-04 | 1.63E-03 |
| sp\|Q9CYG7\|TC | Tomm34 | TOM34_MOUS | 3 | 25.7789 | 20.6446 | 30.4782 | 6.55287 | 7.96062 | 8.58477 | 25.6339 | 4.9184 | 7.6994 | 1.0408 | 0.3004 | 3.49E-03 | 9.53E-03 |
| sp\|Q04750\|TC | Top1 | TOP1_MOUSE | 3 | 14.2626 | 18.1407 | 14.1566 | 17.8078 | 22.541 | 13.0911 | 15.5200 | 2.2702 | 17.8133 | 4.7250 | 1.1478 | 4.91E-01 | 5.39E-01 |
| sp\|Q9CRB6\|TF | Tppp3 | TPPP3_MOUSI | 3 | 27.7905 | 24.7088 | 24.9509 | 6.49662 | 7.3544 | 8.69877 | 25.8167 | 1.7136 | 7.5166 | 1.1100 | 0.2912 | 1.00E-04 | 1.63E-03 |
| sp\|Q9JJZ2\|TBA | Tuba8 | TBA8_MOUSE | 3 | 22.3916 | 29.8508 | 31.485 | 6.26085 | 4.30682 | 5.70496 | 27.9091 | 4.8477 | 5.4242 | 1.0068 | 0.1944 | 1.41E-03 | 5.48E-03 |
| sp\|P99024\|TB | Tubb5 | TBB5_MOUSE | 3 | 22.0192 | 32.3823 | 29.8823 | 7.06897 | 4.65163 | 3.99556 | 28.0946 | 5.4079 | 5.2387 | 1.6186 | 0.1865 | 2.18E-03 | 6.84E-03 |
| sp\|P61089\|UE | Ube2n | UBE2N_MOUS | 3 | 22.8131 | 20.2926 | 25.1407 | 14.0567 | 9.41029 | 8.28663 | 22.7488 | 2.4247 | 10.5845 | 3.0590 | 0.4653 | 5.70E-03 | 1.36E-02 |
| sp\|Q9QZM0\|U | Ubqln2 | UBQL2_MOUS | 3 | 34.9187 | 25.164 | 24.2666 | 5.20345 | 4.75145 | 5.69587 | 28.1164 | 5.9080 | 5.2169 | 0.4724 | 0.1855 | 2.59E-03 | 7.74E-03 |
| sp\|Q9JMA1\|U | Usp14 | UBP14_MOUS | 3 | 22.9322 | 18.7003 | 33.8632 | 8.94484 | 6.98961 | 8.56986 | 25.1652 | 7.8242 | 8.1681 | 1.0377 | 0.3246 | 2.03E-02 | 3.52E-02 |
| sp\|P62960\|YB | Ybx1 | YBOX1_MOUS | 3 | 26.4921 | 25.0711 | 25.5826 | 3.6091 | 9.64273 | 9.60232 | 25.7153 | 0.7197 | 7.6181 | 3.4719 | 0.2962 | 9.04E-04 | 4.52E-03 |
| sp\|Q9CQW1\|Y | Ykt6 | YKT6_MOUSE | 3 | 19.1239 | 14.8631 | 20.0624 | 18.4107 | 14.2079 | 13.332 | 18.0165 | 2.7709 | 15.3169 | 2.7149 | 0.8502 | 2.94E-01 | 3.54E-01 |

| | | | | | | | | | | | | | | | | | |
|---|---|---|---|---|---|---|---|---|---|---|---|---|---|---|---|---|---|
| sp\|P61982\|14 | Ywhag | 1433G_MOUSI | 3 | 23.7738 | 20.3194 | 25.5958 | 12.7646 | 8.63794 | 8.90849 | 23.2297 | 2.6800 | 10.1037 | 2.3084 | 0.4349 | 3.01E-03 | 8.57E-03 |
| sp\|P68254\|14 | Ywhaq | 1433T_MOUSE | 3 | 25.2662 | 22.1988 | 26.2306 | 8.10394 | 7.86833 | 10.3322 | 24.5652 | 2.1053 | 8.7682 | 1.3596 | 0.3569 | 4.00E-04 | 2.89E-03 |
| sp\|Q922Q1\|M | 2-Mar | MARC2_MOUS | 2 | 21.0216 | 22.1836 | 19.9778 | 12.0719 | 13.0532 | 11.6919 | 21.0610 | 1.1034 | 12.2723 | 0.7024 | 0.5827 | 3.12E-04 | 2.57E-03 |
| sp\|Q9Z2Q6\|SE | 5-Sep | SEPT5_MOUSE | 2 | 23.1491 | 26.44 | 22.4824 | 12.7114 | 7.16048 | 8.05664 | 24.0238 | 2.1188 | 9.3095 | 2.9800 | 0.3875 | 2.23E-03 | 6.98E-03 |
| tr\|Q99PA7\|Q\$4930550L24Rik | | Q99PA7_MOU | 2 | 32.5069 | 24.5571 | 36.7803 | 0 | 0.966364 | 5.18932 | 31.2814 | 6.2031 | 2.0519 | 2.7597 | 0.0656 | 1.73E-03 | 5.99E-03 |
| sp\|Q8CAY6\|TH | Acat2 | THIC_MOUSE | 2 | 23.0094 | 21.3933 | 27.9229 | 8.2392 | 8.66663 | 10.7686 | 24.1085 | 3.4007 | 9.2248 | 1.3539 | 0.3826 | 2.14E-03 | 6.84E-03 |
| sp\|Q32MW3\| | Acot10 | ACO10_MOUS | 2 | 23.8776 | 17.9444 | 23.8473 | 12.274 | 10.8172 | 11.2396 | 21.8898 | 3.4168 | 11.4436 | 0.7495 | 0.5228 | 6.64E-03 | 1.50E-02 |
| sp\|Q9CZW4\|A | Acsl3 | ACSL3_MOUSE | 2 | 28.5247 | 29.2636 | 27.2064 | 3.53246 | 3.53527 | 7.9376 | 28.3316 | 1.0421 | 5.0018 | 2.5425 | 0.1765 | 1.24E-04 | 1.79E-03 |
| sp\|P54822\|PU | Adsl | PUR8_MOUSE | 2 | 30.223 | 28.176 | 30.1329 | 3.22807 | 3.06734 | 5.17274 | 29.5106 | 1.1567 | 3.8227 | 1.1719 | 0.1295 | 1.12E-05 | 8.92E-04 |
| tr\|E9PYB0\|E9F | Ahnak2 | E9PYB0_MOUS | 2 | 2.35218 | 0.984889 | 0.916409 | 40.3096 | 27.2467 | 28.1902 | 1.4178 | 0.8099 | 31.9155 | 7.2848 | 22.5102 | 1.97E-03 | 6.49E-03 |
| sp\|Q8R010\|AI | Aimp2 | AIMP2_MOUS | 2 | 23.5704 | 31.5 | 22.6525 | 9.7582 | 6.14871 | 6.3701 | 25.9076 | 4.8648 | 7.4257 | 2.0231 | 0.2866 | 3.71E-03 | 9.86E-03 |
| sp\|Q9R0Y5\|KA | Ak1 | KAD1_MOUSE | 2 | 30.7913 | 19.9353 | 28.6336 | 3.69804 | 8.74993 | 8.19178 | 26.4534 | 5.7470 | 6.8799 | 2.7697 | 0.2601 | 6.03E-03 | 1.41E-02 |
| sp\|Q9DBT5\|AI | Ampd2 | AMPD2_MOUS | 2 | 26.9185 | 24.5211 | 30.0169 | 6.89445 | 5.19166 | 6.45735 | 27.1522 | 2.7553 | 6.1812 | 0.8844 | 0.2276 | 2.32E-04 | 2.27E-03 |
| sp\|Q9EST5\|AN | Anp32b | AN32B_MOUS | 2 | 30.6271 | 19.5671 | 27.3209 | 6.6537 | 5.25191 | 10.5793 | 25.8384 | 5.6771 | 7.4950 | 2.7615 | 0.2901 | 7.32E-03 | 1.62E-02 |
| sp\|Q07076\|AN | Anxa7 | ANXA7_MOUS | 2 | 14.5742 | 14.3436 | 18.2597 | 17.6903 | 15.3203 | 19.812 | 15.7258 | 2.1974 | 17.6075 | 2.2470 | 1.1197 | 3.58E-01 | 4.18E-01 |
| sp\|Q60875\|AF | Arhgef2 | ARHG2_MOUS | 2 | 17.6254 | 25.7762 | 13.9643 | 9.73957 | 7.69043 | 25.2042 | 19.1220 | 6.0465 | 14.2114 | 9.5750 | 0.7432 | 4.94E-01 | 5.42E-01 |
| sp\|P61211\|AR | Arl1 | ARL1_MOUSE | 2 | 26.0084 | 21.9665 | 24.4939 | 6.29345 | 11.2539 | 9.98388 | 24.1563 | 2.0420 | 9.1771 | 2.5768 | 0.3799 | 1.39E-03 | 5.48E-03 |
| sp\|Q9JM76\|AI | Arpc3 | ARPC3_MOUS | 2 | 16.8687 | 16.3538 | 19.894 | 19.7456 | 13.8282 | 13.3097 | 17.7055 | 1.9127 | 15.6278 | 3.5755 | 0.8827 | 4.25E-01 | 4.78E-01 |
| sp\|Q9CPW4\|A | Arpc5 | ARPC5_MOUS | 2 | 21.133 | 17.5623 | 22.1498 | 14.7752 | 12.4123 | 11.9675 | 20.2817 | 2.4093 | 13.0517 | 1.5091 | 0.6435 | 1.16E-02 | 2.25E-02 |
| sp\|O54984\|AS | Asna1 | ASNA_MOUSE | 2 | 26.4701 | 23.1057 | 30.0092 | 5.90918 | 7.74887 | 6.75698 | 26.5283 | 3.4521 | 6.8050 | 0.9208 | 0.2565 | 6.68E-04 | 3.87E-03 |
| sp\|Q9CQQ7\|A | Atp5f1 | AT5F1_MOUSI | 2 | 23.4166 | 24.1521 | 21.0065 | 13.0454 | 9.82087 | 8.55852 | 22.8584 | 1.6454 | 10.4749 | 2.3138 | 0.4583 | 1.65E-03 | 5.94E-03 |
| sp\|P57746\|VA | Atp6v1d | VATD_MOUSE | 2 | 19.1588 | 18.3281 | 23.1241 | 11.6372 | 13.7623 | 13.9894 | 20.2037 | 2.5630 | 13.1296 | 1.2975 | 0.6499 | 1.30E-02 | 2.46E-02 |
| sp\|Q8BVE3\|V | Atp6v1h | VATH_MOUSE | 2 | 23.8117 | 25.3486 | 29.5906 | 10.5531 | 5.86015 | 4.83585 | 26.2503 | 2.9931 | 7.0830 | 3.0485 | 0.2698 | 1.48E-03 | 5.61E-03 |
| sp\|Q06138\|CA | Cab39 | CAB39_MOUS | 2 | 25.3889 | 22.2223 | 27.017 | 13.1369 | 6.62592 | 5.60896 | 24.8761 | 2.4381 | 8.4573 | 4.0845 | 0.3400 | 3.93E-03 | 1.03E-02 |
| sp\|O35887\|CA | Calu | CALU_MOUSE | 2 | 24.4654 | 20.9799 | 16.0618 | 17.4393 | 11.3287 | 9.72492 | 20.5024 | 4.2221 | 12.8310 | 4.0707 | 0.6258 | 8.61E-02 | 1.19E-01 |
| sp\|Q6PHZ2\|KC | Camk2d | KCC2D_MOUS | 2 | 18.4081 | 18.9567 | 21.1613 | 17.3301 | 14.3121 | 9.83166 | 19.5087 | 1.4572 | 13.8246 | 3.7729 | 0.7086 | 7.17E-02 | 1.01E-01 |
| sp\|Q6ZQ73\|C | Cand2 | CAND2_MOUS | 2 | 24.2147 | 31.1764 | 31.6472 | 4.03674 | 4.38421 | 4.54077 | 29.0128 | 4.1619 | 4.3206 | 0.2580 | 0.1489 | 5.09E-04 | 3.35E-03 |
| sp\|O08529\|CA | Capn2 | CAN2_MOUSE | 2 | 14.643 | 13.4636 | 19.1202 | 23.6718 | 13.6445 | 15.4569 | 15.7423 | 2.9842 | 17.5911 | 5.3435 | 1.1174 | 6.28E-01 | 6.68E-01 |
| sp\|P47753\|CA | Capza1 | CAZA1_MOUSI | 2 | 25.4371 | 17.708 | 26.9324 | 12.7233 | 7.58289 | 9.61636 | 23.3592 | 4.9508 | 9.9742 | 2.5888 | 0.4270 | 1.43E-02 | 2.64E-02 |
| sp\|P30275\|KC | Ckmt1 | KCRU_MOUSE | 2 | 15.1959 | 13.1821 | 37.8961 | 10.6231 | 6.98296 | 16.1198 | 22.0914 | 13.7243 | 11.2420 | 4.5997 | 0.5089 | 2.64E-01 | 3.20E-01 |
| sp\|Q9QYB1\|CI | Clic4 | CLIC4_MOUSE | 2 | 23.1681 | 23.0552 | 32.299 | 8.71324 | 4.3821 | 8.38242 | 26.1741 | 5.3046 | 7.1593 | 2.4108 | 0.2735 | 4.83E-03 | 1.19E-02 |
| sp\|Q5SW19\|C | Cluh | CLU_MOUSE C | 2 | 21.148 | 19.8862 | 17.797 | 13.8605 | 15.644 | 11.6643 | 19.6104 | 1.6924 | 13.7229 | 1.9934 | 0.6998 | 1.75E-02 | 3.12E-02 |
| sp\|Q08093\|CN | Cnn2 | CNN2_MOUSE | 2 | 5.5061 | 5.27332 | 5.06344 | 36.5187 | 26.8737 | 20.7648 | 5.2810 | 0.2214 | 28.0524 | 7.9428 | 5.3120 | 7.69E-03 | 1.68E-02 |
| sp\|Q8K297\|GT | Colgalt1 | GT251_MOUSI | 2 | 19.219 | 21.5916 | 23.2572 | 11.6849 | 14.8801 | 9.36719 | 21.3559 | 2.0294 | 11.9774 | 2.7681 | 0.5608 | 9.09E-03 | 1.87E-02 |
| sp\|P61202\|CS | Cops2 | CSN2_MOUSE | 2 | 23.7054 | 25.2734 | 27.4122 | 8.38037 | 6.43568 | 8.79287 | 25.4637 | 1.8607 | 7.8696 | 1.2589 | 0.3091 | 1.71E-04 | 1.97E-03 |
| sp\|O88544\|CS | Cops4 | CSN4_MOUSE | 2 | 24.0695 | 20.4151 | 29.1556 | 8.3409 | 8.72757 | 9.29133 | 24.5467 | 4.3897 | 8.7866 | 0.4780 | 0.3580 | 3.48E-03 | 9.53E-03 |
| sp\|Q6NVF9\|CI | Cpsf6 | CPSF6_MOUSE | 2 | 18.4014 | 20.6397 | 17.0789 | 21.0611 | 11.5188 | 11.3002 | 18.7067 | 1.7999 | 14.6267 | 5.5734 | 0.7819 | 2.94E-01 | 3.53E-01 |
| sp\|P97742\|CP | Cpt1a | CPT1A_MOUSI | 2 | 19.4544 | 23.0602 | 20.1853 | 16.4362 | 11.6 | 9.26401 | 20.9000 | 1.9062 | 12.4334 | 3.6580 | 0.5949 | 2.37E-02 | 3.97E-02 |
| sp\|P22935\|RA | Crabp2 | RABP2_MOUS | 2 | 8.82398 | 7.17415 | 56.3785 | 6.63994 | 8.52896 | 12.4545 | 24.1255 | 27.9441 | 9.2078 | 2.9661 | 0.3817 | 4.10E-01 | 4.64E-01 |
| sp\|Q9DCT8\|CI | Crip2 | CRIP2_MOUSE | 2 | 26.2136 | 30.1556 | 23.7321 | 8.10067 | 5.68976 | 6.10819 | 26.7004 | 3.2393 | 6.6329 | 1.2883 | 0.2484 | 5.68E-04 | 3.52E-03 |
| sp\|Q60737\|CS | Csnk2a1 | CSK21_MOUSI | 2 | 26.356 | 24.3603 | 24.221 | 7.39238 | 9.37181 | 8.29863 | 24.9791 | 1.1945 | 8.3543 | 0.9909 | 0.3345 | 4.97E-05 | 1.33E-03 |
| sp\|P67871\|CS | Csnk2b | CSK2B_MOUSI | 2 | 21.4244 | 22.3446 | 16.8477 | 8.90305 | 12.8239 | 17.6563 | 20.2056 | 2.9442 | 13.1278 | 4.3845 | 0.6497 | 8.10E-02 | 1.13E-01 |
| sp\|Q62426\|CY | Cstb | CYTB_MOUSE | 2 | 27.1699 | 16.8615 | 21.7437 | 12.2805 | 8.28597 | 13.6584 | 21.9250 | 5.1566 | 11.4083 | 2.7904 | 0.5203 | 3.60E-02 | 5.54E-02 |
| sp\|Q8VCN5\|C | Cth | CGL_MOUSE C | 2 | 25.3433 | 23.9619 | 29.2816 | 6.90469 | 7.28226 | 7.22624 | 26.1956 | 2.7604 | 7.1377 | 0.2038 | 0.2725 | 2.83E-04 | 2.45E-03 |
| sp\|P30999\|CT | Ctnnd1 | CTND1_MOUS | 2 | 15.1878 | 16.6598 | 14.0947 | 20.8919 | 20.3149 | 12.851 | 15.3141 | 1.2872 | 18.0193 | 4.4851 | 1.1766 | 3.72E-01 | 4.31E-01 |
| sp\|P70698\|PY | Ctps1 | PYRG1_MOUS | 2 | 12.9773 | 39.3208 | 21.002 | 8.60563 | 10.955 | 7.13933 | 24.4334 | 13.5028 | 8.9000 | 1.9248 | 0.3643 | 1.20E-01 | 1.59E-01 |
| sp\|Q9JLV5\|CU | Cul3 | CUL3_MOUSE | 2 | 20.4207 | 17.2873 | 19.4444 | 13.454 | 14.0952 | 15.2985 | 19.0508 | 1.6034 | 14.2826 | 0.9364 | 0.7497 | 1.13E-02 | 2.22E-02 |
| sp\|P62897\|CY | Cycs | CYC_MOUSE C | 2 | 21.8489 | 26.2105 | 23.7522 | 7.92605 | 8.96904 | 11.2933 | 23.9372 | 2.1867 | 9.3961 | 1.7238 | 0.3925 | 8.28E-04 | 4.32E-03 |
| sp\|Q62418\|DE | Dbnl | DBNL_MOUSE | 2 | 33.4065 | 20.3115 | 28.8598 | 6.84256 | 3.8712 | 6.70843 | 27.5259 | 6.6486 | 5.8074 | 1.6781 | 0.2110 | 5.38E-03 | 1.30E-02 |
| sp\|Q61655\|DI | Ddx19a | DD19A_MOUS | 2 | 20.7057 | 19.5027 | 26.093 | 14.1551 | 9.3602 | 10.1833 | 22.1005 | 3.5096 | 11.2329 | 2.5640 | 0.5083 | 1.23E-02 | 2.35E-02 |
| sp\|Q9Z1N5\|D | Ddx39b | DX39B_MOUS | 2 | 25.5633 | 25.4011 | 34.7215 | 0 | 6.68031 | 7.63371 | 28.5620 | 5.3349 | 4.7713 | 4.1595 | 0.1671 | 3.67E-03 | 9.81E-03 |
| sp\|Q810A7\|DI | Ddx42 | DDX42_MOUS | 2 | 22.1712 | 21.8513 | 29.5317 | 9.0654 | 9.18555 | 8.19487 | 24.5181 | 4.3449 | 8.8153 | 0.5406 | 0.3595 | 3.42E-03 | 9.40E-03 |
| sp\|P54823\|DD | Ddx6 | DDX6_MOUSE | 2 | 18.0053 | 18.4781 | 26.8499 | 8.22513 | 12.0166 | 16.4249 | 21.1111 | 4.9756 | 12.2222 | 4.1037 | 0.5789 | 7.54E-02 | 1.05E-01 |
| sp\|P63037\|DN | Dnaja1 | DNJA1_MOUSI | 2 | 27.6707 | 29.1808 | 25.0035 | 6.75684 | 5.66476 | 5.72334 | 27.2850 | 2.1152 | 6.0483 | 0.6143 | 0.2217 | 7.53E-05 | 1.54E-03 |
| sp\|Q99KV1\|D | Dnajb11 | DJB11_MOUSE | 2 | 20.9545 | 19.2475 | 24.8124 | 12.3565 | 12.2665 | 10.3625 | 21.6715 | 2.8509 | 11.6618 | 1.1262 | 0.5381 | 4.82E-03 | 1.19E-02 |
| sp\|Q9R0P5\|DI | Dstn | DEST_MOUSE | 2 | 21.1321 | 18.5624 | 18.1028 | 11.4854 | 16.0638 | 14.6535 | 19.2658 | 1.6325 | 14.0676 | 2.3448 | 0.7302 | 3.45E-02 | 5.35E-02 |
| sp\|P63168\|DY | Dynll1 | DYL1_MOUSE | 2 | 19.2984 | 18.6427 | 24.2611 | 8.44975 | 7.33914 | 22.0089 | 20.7341 | 3.0720 | 12.5993 | 8.1679 | 0.6077 | 1.82E-01 | 2.28E-01 |
| sp\|Q3UJB9\|ED | Edc4 | EDC4_MOUSE | 2 | 23.9268 | 23.9392 | 23.1295 | 9.04943 | 11.356 | 8.59896 | 23.6652 | 0.4639 | 9.6681 | 1.4790 | 0.4085 | 9.76E-05 | 1.63E-03 |
| sp\|O70251\|EF | Eef1b | EF1B_MOUSE | 2 | 25.3984 | 29.8888 | 16.3909 | 13.5456 | 8.48233 | 6.29397 | 23.8927 | 6.8738 | 9.4406 | 3.7196 | 0.3951 | 3.28E-02 | 5.14E-02 |
| sp\|Q60872\|IF | Eif1a | IF1A_MOUSE E | 2 | 22.9339 | 22.0252 | 22.0491 | 15.6839 | 9.87278 | 7.43518 | 22.3361 | 0.5179 | 10.9973 | 4.2378 | 0.4924 | 1.00E-02 | 2.02E-02 |
| sp\|Q8BJW6\|EI | Eif2a | EIF2A_MOUSE | 2 | 23.3033 | 24.8834 | 24.1421 | 9.29295 | 8.65523 | 9.72306 | 24.1096 | 0.7906 | 9.2237 | 0.5373 | 0.3826 | 1.12E-05 | 8.92E-04 |
| sp\|Q9QZD9\|EI | Eif3i | EIF3I_MOUSE | 2 | 24.0529 | 27.0445 | 26.8631 | 7.73992 | 7.4922 | 6.80732 | 25.9868 | 1.6773 | 7.3465 | 0.4831 | 0.2827 | 5.03E-05 | 1.33E-03 |
| sp\|Q9WUK2\|I | Eif4h | IF4H_MOUSE E | 2 | 26.5104 | 17.0982 | 27.9077 | 6.12035 | 8.30874 | 14.0546 | 23.8388 | 5.8792 | 9.4946 | 4.0979 | 0.3983 | 2.57E-02 | 4.24E-02 |

| | | | | | | | | | | | | | | | | |
|---|---|---|---|---|---|---|---|---|---|---|---|---|---|---|---|---|
| sp\|P59325\|IF5 | Eif5 | IF5_MOUSE Eu | 2 | 23.7414 | 19.9882 | 25.0453 | 14.0649 | 10.3378 | 6.82242 | 22.9250 | 2.6255 | 10.4084 | 3.6218 | 0.4540 | 8.36E-03 | 1.78E-02 |
| sp\|Q05D44\|IF | Eif5b | IF2P_MOUSE E | 2 | 14.5998 | 19.127 | 13.2001 | 27.853 | 14.6553 | 10.5649 | 15.6423 | 3.0979 | 17.6911 | 9.0350 | 1.1310 | 7.29E-01 | 7.60E-01 |
| sp\|O55135\|IF6 | Eif6 | IF6_MOUSE Eu | 2 | 20.9493 | 18.1922 | 25.7379 | 11.6643 | 9.72181 | 13.7346 | 21.6265 | 3.8182 | 11.7069 | 2.0067 | 0.5413 | 1.64E-02 | 2.93E-02 |
| sp\|Q60899\|EL | Elavl2 | ELAV2_MOUSE | 2 | 27.7604 | 41.5415 | 23.3431 | 3.1053 | 2.08745 | 2.16225 | 30.8817 | 9.4922 | 2.4517 | 0.5673 | 0.0794 | 6.61E-03 | 1.50E-02 |
| sp\|Q3U7R1\|ES | Esyt1 | ESYT1_MOUSE | 2 | 20.9884 | 21.2652 | 21.6306 | 15.7474 | 10.799 | 9.56942 | 21.2947 | 0.3221 | 12.0386 | 3.2702 | 0.5653 | 8.17E-03 | 1.75E-02 |
| sp\|Q99LC5\|ET | Etfa | ETFA_MOUSE | 2 | 20.7839 | 22.8289 | 25.7846 | 11.007 | 11.352 | 8.24371 | 23.1325 | 2.5141 | 10.2009 | 1.7037 | 0.4410 | 1.80E-03 | 6.13E-03 |
| sp\|Q61545\|EV | Ewsr1 | EWS_MOUSE R | 2 | 23.6797 | 19.8732 | 15.7581 | 16.3194 | 16.0587 | 8.31085 | 19.7703 | 3.9618 | 13.5630 | 4.5503 | 0.6860 | 1.49E-01 | 1.93E-01 |
| sp\|P58462\|FO | Foxp1 | FOXP1_MOUS | 2 | 17.0938 | 38.6456 | 14.4765 | 11.6458 | 9.32236 | 8.81599 | 23.4053 | 13.2632 | 9.9281 | 1.5090 | 0.4242 | 1.55E-01 | 1.99E-01 |
| sp\|Q9CQM9\|G | Glrx3 | GLRX3_MOUSE | 2 | 27.9073 | 20.9094 | 29.366 | 7.45786 | 7.22284 | 7.13649 | 26.0609 | 4.5206 | 7.2724 | 0.1663 | 0.2791 | 1.98E-03 | 6.52E-03 |
| tr\|A2AFI6\|A2A | Gm364 | A2AFI6_MOUS | 2 | 29.1441 | 32.5065 | 25.4072 | 3.21766 | 4.37626 | 5.34838 | 29.0193 | 3.5513 | 4.3141 | 1.0667 | 0.1487 | 3.22E-04 | 2.60E-03 |
| sp\|P05201\|AA | Got1 | AATC_MOUSE | 2 | 27.6594 | 19.2524 | 32.1168 | 8.78563 | 6.07672 | 6.10907 | 26.3429 | 6.5325 | 6.9905 | 1.5547 | 0.2654 | 7.53E-03 | 1.65E-02 |
| sp\|Q8BUV3\|G | Gphn | GEPH_MOUSE | 2 | 22.96 | 31.8927 | 30.2301 | 7.25279 | 2.26916 | 5.39527 | 28.3609 | 4.7506 | 4.9724 | 2.5186 | 0.1753 | 1.66E-03 | 5.94E-03 |
| sp\|P47791\|GS | Gsr | GSHR_MOUSE | 2 | 28.2996 | 23.4143 | 31.6185 | 4.00245 | 2.9915 | 9.67369 | 27.7775 | 4.1269 | 5.5559 | 3.6018 | 0.2000 | 2.16E-03 | 6.84E-03 |
| sp\|P10922\|H1 | H1f0 | H10_MOUSE H | 2 | 12.9527 | 17.1769 | 9.57026 | 23.5506 | 21.8451 | 14.9044 | 13.2333 | 3.8111 | 20.1000 | 4.5796 | 1.5189 | 1.17E-01 | 1.55E-01 |
| sp\|Q9D8V0\|H | Hm13 | HM13_MOUSE | 2 | 26.3741 | 23.9824 | 27.235 | 8.35668 | 7.21115 | 6.84075 | 25.8638 | 1.6853 | 7.4695 | 0.7903 | 0.2888 | 6.83E-05 | 1.49E-03 |
| sp\|Q9CX86\|RO | Hnrnpa0 | ROA0_MOUSE | 2 | 18.3553 | 16.6369 | 13.7803 | 19.3098 | 18.3501 | 13.5676 | 16.2575 | 2.3110 | 17.0758 | 3.0759 | 1.0503 | 7.31E-01 | 7.62E-01 |
| sp\|Q9Z130\|HN | Hnrnpdl | HNRDL_MOUS | 2 | 18.0834 | 26.5567 | 17.1597 | 14.1704 | 14.0311 | 9.99876 | 20.5999 | 5.1793 | 12.7334 | 2.3693 | 0.6181 | 7.50E-02 | 1.05E-01 |
| sp\|Q8BUK6\|H | Hook3 | HOOK3_MOUS | 2 | 21.4091 | 22.6359 | 18.5991 | 16.2525 | 9.26646 | 11.837 | 20.8814 | 2.0695 | 12.4520 | 3.5334 | 0.5963 | 2.35E-02 | 3.95E-02 |
| sp\|P14602\|HS | Hspb1 | HSPB1_MOUSE | 2 | 7.14242 | 5.85153 | 27.3722 | 10.8706 | 7.05836 | 41.705 | 13.4554 | 12.0696 | 19.8780 | 18.9986 | 1.4773 | 6.47E-01 | 6.85E-01 |
| sp\|Q7TMY8\|H | Huwe1 | HUWE1_MOU | 2 | 22.3555 | 23.2995 | 23.2235 | 7.25345 | 7.86412 | 16.0039 | 22.9595 | 0.5245 | 10.3738 | 4.8853 | 0.4518 | 1.14E-02 | 2.23E-02 |
| tr\|Q91VA7\|Q9 | Idh3b | Q91VA7_MOU | 2 | 24.1207 | 25.6581 | 22.1123 | 12.116 | 8.14837 | 7.84893 | 23.9637 | 1.7781 | 9.3696 | 2.3793 | 0.3910 | 1.05E-03 | 4.76E-03 |
| sp\|P70404\|IDI | Idh3g | IDHG1_MOUSE | 2 | 18.1397 | 19.8681 | 18.894 | 17.0165 | 15.0673 | 11.0144 | 18.9673 | 0.8665 | 14.3661 | 3.0619 | 0.7574 | 6.64E-02 | 9.47E-02 |
| sp\|P24547\|IM | Impdh2 | IMDH2_MOUS | 2 | 21.3285 | 23.7345 | 23.4525 | 12.4727 | 9.50705 | 9.50478 | 22.8385 | 1.3153 | 10.4948 | 1.7129 | 0.4595 | 5.84E-04 | 3.56E-03 |
| sp\|Q3UQ44\|IG | Iqgap2 | IQGA2_MOUSE | 2 | 14.1572 | 13.1476 | 15.3585 | 24.4915 | 18.7327 | 14.1125 | 14.2211 | 1.1068 | 19.1122 | 5.1999 | 1.3439 | 1.86E-01 | 2.34E-01 |
| sp\|Q60749\|KH | Khdrbs1 | KHDR1_MOUS | 2 | 18.1557 | 21.0257 | 13.7332 | 18.7825 | 17.1387 | 11.1642 | 17.6382 | 3.6737 | 15.6951 | 4.0091 | 0.8898 | 5.69E-01 | 6.11E-01 |
| sp\|P28738\|KIF | Kif5c | KIF5C_MOUSE | 2 | 21.9403 | 23.5053 | 21.107 | 13.5727 | 10.9613 | 8.9134 | 22.1842 | 1.2176 | 11.1491 | 2.3353 | 0.5026 | 1.91E-03 | 6.41E-03 |
| sp\|Q9D646\|KR | Krt34 | KRT34_MOUSE | 2 | 1.97372 | 3.63782 | 1.17922 | 0 | 0.508444 | 92.7008 | 2.2636 | 1.2547 | 31.0697 | 53.3747 | 13.7259 | 4.03E-01 | 4.57E-01 |
| sp\|P11679\|K2 | Krt8 | K2C8_MOUSE | 2 | 11.9381 | 7.46525 | 9.38051 | 22.8193 | 31.3351 | 17.0617 | 9.5946 | 2.2441 | 23.7387 | 7.1810 | 2.4742 | 3.12E-02 | 4.95E-02 |
| sp\|Q0VBK2\|K2 | Krt80 | K2C80_MOUSE | 2 | 9.77877 | 4.70092 | 7.67861 | 19.6376 | 22.9165 | 35.2876 | 7.3861 | 2.5515 | 25.9472 | 8.2535 | 3.5130 | 2.04E-02 | 3.54E-02 |
| sp\|Q9Z2T6\|KR | Krt85 | KRT85_MOUSE | 2 | 2.04092 | 2.6738 | 1.102 | 1.62046 | 5.52348 | 87.0393 | 1.9389 | 0.7909 | 31.3944 | 48.2294 | 16.1918 | 3.50E-01 | 4.10E-01 |
| sp\|Q6ZQ58\|LA | Larp1 | LARP1_MOUSE | 2 | 19.8881 | 25.1205 | 19.0079 | 14.391 | 12.9997 | 8.59274 | 21.3388 | 3.3045 | 11.9945 | 3.0270 | 0.5621 | 2.25E-02 | 3.83E-02 |
| sp\|Q61233\|PL | Lcp1 | PLSL_MOUSE P | 2 | 3.91894 | 2.84145 | 6.59125 | 38.3335 | 24.5603 | 23.7546 | 4.4505 | 1.9306 | 28.8828 | 8.1945 | 6.4897 | 7.35E-03 | 1.62E-02 |
| sp\|Q99JW4\|LI | Lims1 | LIMS1_MOUSE | 2 | 17.937 | 17.7244 | 16.4653 | 20.6305 | 15.1848 | 12.058 | 17.3756 | 0.7954 | 15.9578 | 4.3382 | 0.9184 | 6.07E-01 | 6.48E-01 |
| sp\|Q9CRC8\|LR | Lrrc40 | LRC40_MOUSE | 2 | 27.3637 | 30.4453 | 27.462 | 6.47154 | 2.16391 | 6.09348 | 28.4237 | 1.7515 | 4.9096 | 2.3854 | 0.1727 | 1.62E-04 | 1.93E-03 |
| sp\|P06801\|MA | Me1 | MAOX_MOUSE | 2 | 15.8374 | 15.1419 | 22.6116 | 14.6652 | 12.3111 | 19.4328 | 17.8636 | 4.1265 | 15.4697 | 3.6284 | 0.8660 | 4.92E-01 | 5.40E-01 |
| sp\|Q7TPH6\|M | Mycbp2 | MYCB2_MOUS | 2 | 6.23439 | 4.46355 | 5.12493 | 24.6264 | 25.8755 | 33.6752 | 5.2743 | 0.8948 | 28.0590 | 4.9037 | 5.3200 | 1.38E-03 | 5.48E-03 |
| sp\|Q80UM3\|N | Naa15 | NAA15_MOUS | 2 | 23.6216 | 21.668 | 30.597 | 5.75734 | 7.99287 | 10.3632 | 25.2955 | 4.6940 | 8.0378 | 2.3033 | 0.3178 | 4.63E-03 | 1.16E-02 |
| sp\|Q99MD9\|N | Nasp | NASP_MOUSE | 2 | 26.4444 | 19.2832 | 23.1872 | 10.2022 | 11.2195 | 9.66346 | 22.9716 | 3.5855 | 10.3617 | 0.7902 | 0.4511 | 4.01E-03 | 1.04E-02 |
| sp\|P13595\|NC | Ncam1 | NCAM1_MOU | 2 | 28.7467 | 25.0254 | 28.428 | 7.77318 | 3.8038 | 6.22296 | 27.4000 | 2.0627 | 5.9333 | 2.0005 | 0.2165 | 2.06E-04 | 2.11E-03 |
| sp\|Q8K2Z4\|CN | Ncapd2 | CND1_MOUSE | 2 | 25.2011 | 34.7538 | 27.0289 | 4.03248 | 4.00355 | 4.98026 | 28.9946 | 5.0707 | 4.3388 | 0.5557 | 0.1496 | 1.11E-03 | 4.90E-03 |
| sp\|Q62425\|NI | Ndufa4 | NDUA4_MOUS | 2 | 26.9506 | 31.0761 | 25.4672 | 4.94546 | 2.56589 | 8.99475 | 27.8313 | 2.9063 | 5.5020 | 3.2504 | 0.1977 | 8.92E-04 | 4.50E-03 |
| sp\|Q91VD9\|N | Ndufs1 | NDUS1_MOUS | 2 | 20.1258 | 29.1159 | 25.0574 | 7.85504 | 8.96143 | 8.88439 | 24.7664 | 4.5021 | 8.5670 | 0.6177 | 0.3459 | 3.49E-03 | 9.54E-03 |
| sp\|O55125\|NI | Nipsnap1 | NIPS1_MOUSE | 2 | 19.3097 | 19.7416 | 24.1171 | 10.5007 | 10.4341 | 15.8968 | 21.0561 | 2.6597 | 12.2772 | 3.1348 | 0.5831 | 2.09E-02 | 3.59E-02 |
| sp\|O70310\|NI | Nmt1 | NMT1_MOUSE | 2 | 27.1485 | 26.1166 | 27.0549 | 7.48353 | 7.52506 | 4.67141 | 26.7733 | 0.5707 | 6.5600 | 1.6357 | 0.2450 | 3.54E-05 | 1.17E-03 |
| sp\|Q61941\|NI | Nnt | NNTM_MOUSE | 2 | 16.2634 | 15.4779 | 19.7927 | 23.6608 | 14.3156 | 10.4897 | 17.1780 | 2.2982 | 16.1554 | 6.7755 | 0.9405 | 8.17E-01 | 8.43E-01 |
| sp\|P54729\|NU | Nub1 | NUB1_MOUSE | 2 | 24.4725 | 25.19 | 26.4696 | 8.14286 | 7.75973 | 7.96524 | 25.3774 | 1.0116 | 7.9559 | 0.1917 | 0.3135 | 8.07E-06 | 8.92E-04 |
| sp\|Q9CQF3\|CI | Nudt21 | CPSF5_MOUSE | 2 | 20.8009 | 21.1769 | 21.2573 | 14.5838 | 12.0853 | 10.0957 | 21.0784 | 0.2436 | 12.2549 | 2.2489 | 0.5814 | 2.50E-03 | 7.57E-03 |
| sp\|Q9QY81\|PO | Nup210 | PO210_MOUS | 2 | 29.6431 | 34.5024 | 27.7246 | 0 | 0 | 8.12999 | 30.6234 | 3.4936 | 2.7100 | 4.6939 | 0.0885 | 1.17E-03 | 5.07E-03 |
| sp\|Q99PG2\|O | Ogfr | OGFR_MOUSE | 2 | 32.6639 | 27.5498 | 25.9816 | 6.29684 | 4.03143 | 3.47644 | 28.7318 | 3.4944 | 4.6016 | 1.4941 | 0.1602 | 3.89E-04 | 2.88E-03 |
| sp\|Q8CGY8\|O | Ogt | OGT1_MOUSE | 2 | 19.8631 | 20.613 | 16.7015 | 18.7244 | 11.3277 | 12.7703 | 19.0592 | 2.0760 | 14.2741 | 3.9210 | 0.7489 | 1.35E-01 | 1.77E-01 |
| sp\|Q8CIN4\|PA | Pak2 | PAK2_MOUSE | 2 | 10.2114 | 12.6679 | 12.4837 | 19.9436 | 24.9379 | 19.7554 | 11.7877 | 1.3682 | 21.5456 | 2.9393 | 1.8278 | 6.46E-03 | 1.47E-02 |
| sp\|Q4VA53\|PI | Pds5b | PDS5B_MOUSE | 2 | 31.2268 | 20.7886 | 26.2762 | 7.38473 | 4.52229 | 9.8014 | 26.0972 | 5.2214 | 7.2361 | 2.6427 | 0.2773 | 5.05E-03 | 1.24E-02 |
| sp\|P12382\|K6 | Pfkl | K6PL_MOUSE | 2 | 24.4372 | 29.5851 | 27.3141 | 7.6182 | 6.16691 | 4.87845 | 27.1121 | 2.5799 | 6.2212 | 1.3707 | 0.2295 | 2.44E-04 | 2.29E-03 |
| sp\|Q9DBG5\|P | Plin3 | PLIN3_MOUSE | 2 | 30.0348 | 22.2359 | 32.5145 | 7.14171 | 1.64408 | 6.42903 | 28.2617 | 5.3638 | 5.0716 | 2.9896 | 0.1795 | 2.82E-03 | 8.17E-03 |
| sp\|Q9R0E1\|PL | Plod3 | PLOD3_MOUS | 2 | 20.786 | 18.173 | 19.9815 | 11.8353 | 19.613 | 9.61125 | 19.6468 | 1.3383 | 13.6865 | 5.2516 | 0.6966 | 1.30E-01 | 1.71E-01 |
| sp\|Q9CXT8\|M | Pmpcb | MPPB_MOUSE | 2 | 24.7383 | 26.4623 | 26.101 | 5.66055 | 6.7951 | 10.2429 | 25.7672 | 0.9092 | 7.5662 | 2.3865 | 0.2936 | 2.47E-04 | 2.29E-03 |
| sp\|Q8K1R3\|PN | Pnpt1 | PNPT1_MOUS | 2 | 20.092 | 20.2598 | 20.4958 | 15.3326 | 12.9065 | 10.9132 | 20.2825 | 0.2029 | 13.0508 | 2.2132 | 0.6434 | 4.88E-03 | 1.20E-02 |
| sp\|Q9CR16\|PF | Ppid | PPID_MOUSE | 2 | 24.8786 | 22.9066 | 27.8616 | 11.3149 | 6.40341 | 6.63498 | 25.2156 | 2.4946 | 8.1178 | 2.7712 | 0.3219 | 1.36E-03 | 5.44E-03 |
| sp\|P63330\|PP | Ppp2ca | PP2AA_MOUS | 2 | 25.4726 | 22.5272 | 25.8892 | 10.6642 | 7.32561 | 8.12115 | 24.6297 | 1.8327 | 8.7037 | 1.7439 | 0.3534 | 4.02E-04 | 2.89E-03 |
| sp\|Q60676\|PF | Ppp5c | PPP5_MOUSE | 2 | 23.3518 | 23.7626 | 34.0391 | 7.46678 | 3.47839 | 7.90127 | 27.0512 | 6.0552 | 6.2821 | 2.4378 | 0.2322 | 5.29E-03 | 1.29E-02 |
| sp\|P20108\|PR | Prdx3 | PRDX3_MOUS | 2 | 30.3237 | 21.3721 | 26.1773 | 9.44926 | 7.5236 | 5.15406 | 25.9577 | 4.4798 | 7.3756 | 2.1514 | 0.2841 | 2.93E-03 | 8.38E-03 |
| sp\|P99029\|PR | Prdx5 | PRDX5_MOUS | 2 | 8.79222 | 11.4413 | 13.003 | 34.118 | 16.9693 | 15.6761 | 11.0788 | 2.1287 | 22.2545 | 10.2944 | 2.0087 | 1.39E-01 | 1.82E-01 |
| sp\|Q9D7G0\|PI | Prps1 | PRPS1_MOUSE | 2 | 26.7745 | 22.6654 | 27.5248 | 5.6457 | 4.88247 | 12.5072 | 25.6549 | 2.6160 | 7.6785 | 4.1992 | 0.2993 | 3.26E-03 | 9.09E-03 |

| | | | | | | | | | | | | | | | | | |
|---|---|---|---|---|---|---|---|---|---|---|---|---|---|---|---|---|---|
| sp\|Q9R1P1\|PS | Psmb3 | PSB3_MOUSE | 2 | 22.8132 | 23.1685 | 24.1736 | 7.68554 | 9.20177 | 12.9574 | 23.3851 | 0.7056 | 9.9482 | 2.7140 | 0.4254 | 1.15E-03 | 5.02E-03 |
| sp\|P99026\|PS | Psmb4 | PSB4_MOUSE | 2 | 17.5412 | 18.8814 | 20.1667 | 12.2194 | 10.5694 | 20.622 | 18.8631 | 1.3128 | 14.4703 | 5.3911 | 0.7671 | 2.42E-01 | 2.97E-01 |
| sp\|O35226\|PS | Psmd4 | PSMD4_MOUS | 2 | 24.8512 | 28.1597 | 29.775 | 6.10848 | 3.92231 | 7.18323 | 27.5953 | 2.5100 | 5.7380 | 1.6617 | 0.2079 | 2.30E-04 | 2.27E-03 |
| sp\|Q8BJY1\|PS | Psmd5 | PSMD5_MOUS | 2 | 22.4176 | 22.7797 | 24.6828 | 7.72501 | 9.67526 | 12.7196 | 23.2934 | 1.2168 | 10.0400 | 2.5172 | 0.4310 | 1.20E-03 | 5.09E-03 |
| sp\|Q9CX56\|PS | Psmd8 | PSMD8_MOUS | 2 | 23.1733 | 28.3361 | 21.4651 | 10.8512 | 8.49175 | 7.68244 | 24.3248 | 3.5773 | 9.0085 | 1.6464 | 0.3703 | 2.53E-03 | 7.61E-03 |
| sp\|P97372\|PS | Psme2 | PSME2_MOUS | 2 | 19.5924 | 25.4935 | 20.8347 | 10.6207 | 6.54152 | 16.9172 | 21.9735 | 3.1110 | 11.3598 | 5.2272 | 0.5170 | 3.91E-02 | 5.95E-02 |
| sp\|P26350\|PT | Ptma | PTMA_MOUSE | 2 | 38.2057 | 19.864 | 32.1697 | 3.21009 | 1.23248 | 5.31807 | 30.0798 | 9.3477 | 3.2535 | 2.0431 | 0.1082 | 8.30E-03 | 1.77E-02 |
| sp\|O54724\|PT | Ptrf | PTRF_MOUSE | 2 | 8.37489 | 9.80981 | 5.45122 | 34.8332 | 23.4565 | 18.0744 | 7.8786 | 2.2213 | 25.4547 | 8.5562 | 3.2308 | 2.62E-02 | 4.31E-02 |
| sp\|Q91V41\|RA | Rab14 | RAB14_MOUS | 2 | 14.5164 | 22.9736 | 23.313 | 17.2351 | 11.7095 | 10.2525 | 20.2677 | 4.9836 | 13.0657 | 3.6836 | 0.6447 | 1.14E-01 | 1.53E-01 |
| sp\|P35278\|RA | Rab5c | RAB5C_MOUS | 2 | 22.1377 | 18.3288 | 26.679 | 11.5532 | 9.11953 | 12.1818 | 22.3818 | 4.1804 | 10.9515 | 1.6174 | 0.4893 | 1.15E-02 | 2.24E-02 |
| sp\|P63001\|RA | Rac1 | RAC1_MOUSE | 2 | 19.566 | 19.1874 | 21.639 | 16.7012 | 11.6598 | 11.2466 | 20.1308 | 1.3198 | 13.2025 | 3.0370 | 0.6558 | 2.23E-02 | 3.79E-02 |
| sp\|P54728\|RD | Rad23b | RD23B_MOUS | 2 | 30.4858 | 16.2557 | 26.4234 | 9.09448 | 9.94418 | 7.79642 | 24.3883 | 7.3301 | 8.9450 | 1.0817 | 0.3668 | 2.26E-02 | 3.83E-02 |
| sp\|Q9ERU9\|RI | Ranbp2 | RBP2_MOUSE | 2 | 22.1084 | 20.8264 | 20.8371 | 10.5763 | 11.7088 | 13.943 | 21.2573 | 0.7371 | 12.0760 | 1.7131 | 0.5681 | 1.04E-03 | 4.76E-03 |
| sp\|Q60972\|RE | Rbbp4 | RBBP4_MOUS | 2 | 24.2969 | 23.5304 | 24.114 | 11.6518 | 8.88011 | 7.5269 | 23.9804 | 0.4003 | 9.3529 | 2.1027 | 0.3900 | 2.92E-04 | 2.46E-03 |
| sp\|Q91VI7\|RI | Rnh1 | RINI_MOUSE R | 2 | 19.902 | 20.2977 | 21.2321 | 15.6856 | 13.8719 | 9.01082 | 20.4773 | 0.6830 | 12.8561 | 3.4514 | 0.6278 | 1.99E-02 | 3.47E-02 |
| sp\|Q8VCT3\|AI | Rnpep | AMPB_MOUSE | 2 | 19.7673 | 15.5865 | 25.0261 | 12.5022 | 10.5551 | 16.5629 | 20.1266 | 4.7300 | 13.2067 | 3.0652 | 0.6562 | 1.01E-01 | 1.36E-01 |
| sp\|P70336\|RC | Rock2 | ROCK2_MOUS | 2 | 15.2736 | 23.6473 | 15.0584 | 18.6726 | 14.842 | 12.5061 | 17.9931 | 4.8979 | 15.3402 | 3.1133 | 0.8526 | 4.73E-01 | 5.23E-01 |
| sp\|Q8VEE4\|RF | Rpa1 | RFA1_MOUSE | 2 | 21.7955 | 27.6983 | 21.536 | 10.3497 | 8.19941 | 10.4212 | 23.6766 | 3.4853 | 9.6568 | 1.2626 | 0.4079 | 2.81E-03 | 8.14E-03 |
| sp\|Q9CXW4\|R | Rpl11 | RL11_MOUSE | 2 | 18.4018 | 24.7022 | 15.2303 | 14.2746 | 17.5069 | 9.88417 | 19.4448 | 4.8213 | 13.8886 | 3.8260 | 0.7143 | 1.93E-01 | 2.42E-01 |
| sp\|Q9CR57\|RL | Rpl14 | RL14_MOUSE | 2 | 16.5716 | 21.5562 | 14.1014 | 18.0435 | 19.3391 | 10.3882 | 17.4097 | 3.7974 | 15.9236 | 4.8374 | 0.9146 | 6.97E-01 | 7.32E-01 |
| sp\|Q6ZWV7\|R | Rpl35 | RL35_MOUSE | 2 | 16.9137 | 16.7638 | 11.1592 | 20.1928 | 22.6166 | 12.3539 | 14.9456 | 3.2799 | 18.3878 | 5.3642 | 1.2303 | 3.97E-01 | 4.53E-01 |
| sp\|P47964\|RL | Rpl36 | RL36_MOUSE | 2 | 19.0328 | 19.3287 | 10.7239 | 16.2852 | 21.2294 | 13.4 | 16.3618 | 4.8848 | 16.9715 | 3.9596 | 1.0373 | 8.75E-01 | 8.93E-01 |
| sp\|P83882\|RL | Rpl36a | RL36A_MOUSE | 2 | 17.3818 | 23.1551 | 13.0684 | 16.8896 | 18.9689 | 10.5362 | 17.8684 | 5.0609 | 15.4649 | 4.3932 | 0.8655 | 5.68E-01 | 6.10E-01 |
| sp\|Q9JJI8\|RL3 | Rpl38 | RL38_MOUSE | 2 | 21.1677 | 27.1614 | 17.726 | 14.0079 | 11.9783 | 7.95865 | 22.0184 | 4.7749 | 11.3150 | 3.0787 | 0.5139 | 3.10E-02 | 4.92E-02 |
| sp\|P99027\|RL | Rplp2 | RLA2_MOUSE | 2 | 17.5092 | 33.0606 | 17.7768 | 14.4242 | 9.1664 | 8.06279 | 22.7822 | 8.9024 | 10.5511 | 3.3993 | 0.4631 | 9.03E-02 | 1.24E-01 |
| sp\|P62855\|RS | Rps26 | RS26_MOUSE | 2 | 14.4223 | 24.3543 | 17.3757 | 17.3483 | 16.7754 | 9.72389 | 18.7174 | 5.1001 | 14.6159 | 4.2462 | 0.7809 | 3.45E-01 | 4.04E-01 |
| sp\|Q6ZWU9\|R | Rps27 | RS27_MOUSE | 2 | 22.2224 | 26.3203 | 20.3456 | 10.5174 | 12.2883 | 8.30601 | 22.9628 | 3.0554 | 10.3706 | 1.9952 | 0.4516 | 3.94E-03 | 1.03E-02 |
| sp\|P14069\|S1 | S100a6 | S10A6_MOUS | 2 | 12.4852 | 14.8167 | 14.533 | 26.8534 | 17.2338 | 14.0779 | 13.9450 | 1.2721 | 19.3884 | 6.6547 | 1.3903 | 2.36E-01 | 2.90E-01 |
| sp\|O35114\|SC | Scarb2 | SCRB2_MOUS | 2 | 32.0668 | 22.3034 | 25.4135 | 6.91707 | 5.7509 | 7.54839 | 26.5946 | 4.9877 | 6.7388 | 0.9119 | 0.2534 | 2.47E-03 | 7.50E-03 |
| sp\|Q9CQA3\|SI | Sdhb | SDHB_MOUSE | 2 | 22.9692 | 23.0904 | 23.5633 | 13.5854 | 8.49517 | 8.29648 | 23.2076 | 0.3139 | 10.1257 | 2.9978 | 0.4363 | 1.68E-03 | 5.94E-03 |
| tr\|A2AIX1\|A2 | Sec16a | A2AIX1_MOUS | 2 | 23.5204 | 34.2163 | 21.1183 | 4.01113 | 6.69794 | 10.4359 | 26.2850 | 6.9729 | 7.0483 | 3.2267 | 0.2682 | 1.23E-02 | 2.35E-02 |
| sp\|Q9D662\|SC | Sec23b | SC23B_MOUS | 2 | 26.6398 | 24.1676 | 26.1537 | 9.06709 | 6.02947 | 7.94228 | 25.6537 | 1.3097 | 7.6796 | 1.5358 | 0.2994 | 1.03E-04 | 1.63E-03 |
| tr\|G3X972\|G3 | Sec24c | G3X972_MOU | 2 | 20.4639 | 22.3118 | 25.0726 | 10.8633 | 13.3587 | 7.92972 | 22.6161 | 2.3194 | 10.7172 | 2.7174 | 0.4739 | 4.48E-03 | 1.14E-02 |
| tr\|Q6NXL1\|Q6 | Sec24d | Q6NXL1_MOU | 2 | 15.5966 | 12.5946 | 16.4544 | 11.6934 | 8.38337 | 35.2776 | 14.8819 | 2.0267 | 18.4515 | 14.6656 | 1.2399 | 6.98E-01 | 7.32E-01 |
| sp\|Q8VHE0\|SE | Sec63 | SEC63_MOUS | 2 | 26.0595 | 27.0252 | 30.0654 | 6.62791 | 3.71203 | 6.50997 | 27.7167 | 2.0906 | 5.6166 | 1.6505 | 0.2026 | 1.36E-04 | 1.82E-03 |
| sp\|Q60854\|SP | Serpinb6 | SPB6_MOUSE | 2 | 9.1712 | 9.13423 | 8.55391 | 26.8176 | 20.5069 | 25.8161 | 8.9531 | 0.3462 | 24.3802 | 3.3915 | 2.7231 | 1.43E-03 | 5.49E-03 |
| sp\|P50431\|GL | Shmt1 | GLYC_MOUSE | 2 | 25.4241 | 27.247 | 34.5213 | 4.86291 | 2.57736 | 5.36733 | 29.0641 | 4.8131 | 4.2692 | 1.4867 | 0.1469 | 1.04E-03 | 4.76E-03 |
| sp\|P32037\|GT | Slc2a3 | GTR3_MOUSE | 2 | 33.9725 | 33.4126 | 29.9928 | 0 | 0 | 2.62215 | 32.4593 | 2.1543 | 0.8741 | 1.5139 | 0.0269 | 3.17E-05 | 1.17E-03 |
| sp\|Q91VZ6\|SM | Smap1 | SMAP1_MOUS | 2 | 20.6406 | 19.4439 | 18.4284 | 21.9628 | 12.1461 | 7.37833 | 19.5043 | 1.1073 | 13.8291 | 7.4365 | 0.7090 | 2.61E-01 | 3.17E-01 |
| sp\|Q9CU62\|SM | Smc1a | SMC1A_MOUS | 2 | 17.2141 | 19.2452 | 14.8644 | 10.9609 | 15.8454 | 21.8701 | 17.1079 | 2.1923 | 16.2255 | 5.4645 | 0.9484 | 8.08E-01 | 8.36E-01 |
| sp\|Q62376\|RU | Snrnp70 | RU17_MOUSE | 2 | 29.7446 | 18.1462 | 25.8186 | 8.2472 | 8.97381 | 9.06959 | 24.5698 | 5.8992 | 8.7635 | 0.4497 | 0.3567 | 9.83E-03 | 1.99E-02 |
| sp\|Q9CQI7\|RU | Snrpb2 | RU2B_MOUSE | 2 | 24.9443 | 22.9638 | 21.0211 | 12.5612 | 10.2064 | 8.3031 | 22.9764 | 1.9616 | 10.3569 | 2.1330 | 0.4508 | 1.66E-03 | 5.94E-03 |
| sp\|Q9WV80\|S | Snx1 | SNX1_MOUSE | 2 | 24.4652 | 19.2682 | 25.1644 | 16.2171 | 7.07517 | 7.80989 | 22.9659 | 3.2214 | 10.3674 | 5.0793 | 0.4514 | 2.22E-02 | 3.78E-02 |
| sp\|Q9CWK8\|S | Snx2 | SNX2_MOUSE | 2 | 19.6916 | 20.1185 | 21.4855 | 8.2666 | 15.0676 | 15.3701 | 20.4319 | 0.9371 | 12.9014 | 4.0167 | 0.6314 | 3.41E-02 | 5.32E-02 |
| sp\|O70492\|SN | Snx3 | SNX3_MOUSE | 2 | 23.3078 | 18.4644 | 23.416 | 15.8829 | 9.3933 | 9.53558 | 21.7294 | 2.8281 | 11.6039 | 3.7064 | 0.5340 | 1.97E-02 | 3.45E-02 |
| sp\|Q64105\|SP | Spr | SPRE_MOUSE | 2 | 24.8667 | 19.3506 | 34.8527 | 9.4701 | 5.09887 | 6.36103 | 26.3567 | 7.8577 | 6.9767 | 2.2497 | 0.2647 | 1.48E-02 | 2.72E-02 |
| sp\|P14576\|SR | Srp54 | SRP54_MOUS | 2 | 24.5332 | 21.3193 | 28.3026 | 9.40608 | 6.57535 | 9.86345 | 24.7184 | 3.4953 | 8.6150 | 1.7811 | 0.3485 | 2.07E-03 | 6.73E-03 |
| sp\|P47758\|SR | Srprb | SRPRB_MOUS | 2 | 23.1571 | 25.7756 | 29.4719 | 6.75058 | 6.98668 | 7.85812 | 26.1349 | 3.1727 | 7.1985 | 0.5834 | 0.2754 | 5.27E-04 | 3.42E-03 |
| sp\|Q62093\|SR | Srsf2 | SRSF2_MOUSE | 2 | 28.7403 | 24.6896 | 16.5581 | 12.2593 | 10.564 | 7.18871 | 23.3293 | 6.2040 | 10.0040 | 2.5813 | 0.4288 | 2.64E-02 | 4.33E-02 |
| sp\|P84104\|SR | Srsf3 | SRSF3_MOUSE | 2 | 13.8644 | 21.1832 | 15.2289 | 18.7511 | 19.6235 | 11.3488 | 16.7588 | 3.8919 | 16.5745 | 4.5465 | 0.9890 | 9.60E-01 | 9.66E-01 |
| sp\|O35326\|SR | Srsf5 | SRSF5_MOUSE | 2 | 18.3001 | 20.7882 | 13.642 | 17.0326 | 15.812 | 14.4251 | 17.5768 | 3.6276 | 15.7566 | 1.3046 | 0.8964 | 4.59E-01 | 5.10E-01 |
| sp\|P46978\|ST | Stt3a | STT3A_MOUSE | 2 | 22.744 | 22.0299 | 21.2372 | 13.2838 | 11.0769 | 9.62826 | 22.0037 | 0.7537 | 11.3297 | 1.8408 | 0.5149 | 7.46E-04 | 4.08E-03 |
| sp\|Q9ER00\|ST | Stx12 | STX12_MOUSE | 2 | 21.1183 | 16.6604 | 21.6679 | 12.1211 | 11.5648 | 16.8675 | 19.8155 | 2.7462 | 13.5178 | 2.9142 | 0.6822 | 5.28E-02 | 7.71E-02 |
| sp\|Q9Z2I9\|SU | Sucla2 | SUCB1_MOUS | 2 | 20.0029 | 21.4067 | 22.7608 | 13.0624 | 10.6401 | 12.127 | 21.3901 | 1.3790 | 11.9432 | 1.2216 | 0.5583 | 8.88E-04 | 4.49E-03 |
| sp\|Q64332\|SY | Syn2 | SYN2_MOUSE | 2 | 21.4529 | 22.5142 | 23.9912 | 9.32044 | 10.4148 | 12.3064 | 22.6528 | 1.2748 | 10.6805 | 1.5106 | 0.4715 | 4.67E-04 | 3.17E-03 |
| sp\|P37804\|TA | Tagln | TAGL_MOUSE | 2 | 2.15618 | 1.28236 | 1.54996 | 45.5562 | 25.87 | 23.5854 | 1.6628 | 0.4477 | 31.6705 | 12.0795 | 19.0461 | 1.26E-02 | 2.40E-02 |
| sp\|Q921F2\|TA | Tardbp | TADBP_MOUS | 2 | 19.4557 | 25.4511 | 23.9235 | 9.55866 | 10.8794 | 10.7317 | 22.9434 | 3.1155 | 10.3899 | 0.7237 | 0.4528 | 2.45E-03 | 7.46E-03 |
| sp\|Q8BYA0\|TE | Tbcd | TBCD_MOUSE | 2 | 28.3298 | 24.3263 | 32.6553 | 6.32318 | 3.7836 | 4.58179 | 28.4371 | 4.1655 | 4.8962 | 1.2987 | 0.1722 | 7.30E-04 | 4.06E-03 |
| sp\|P10711\|TC | Tcea1 | TCEA1_MOUSE | 2 | 32.3059 | 23.2618 | 25.5654 | 7.25006 | 5.70209 | 5.91473 | 27.0444 | 4.6999 | 6.2890 | 0.8391 | 0.2325 | 1.67E-03 | 5.94E-03 |
| tr\|Q9Z1A1\|Q9 | Tfg | Q9Z1A1_MOU | 2 | 25.1154 | 24.5619 | 23.4887 | 10.4438 | 7.14321 | 9.24701 | 24.3887 | 0.8271 | 8.9447 | 1.6709 | 0.3668 | 1.37E-04 | 1.82E-03 |
| sp\|P21981\|TG | Tgm2 | TGM2_MOUSE | 2 | 4.49274 | 5.23754 | 1.6096 | 36.9978 | 32.9384 | 18.724 | 3.7800 | 1.9161 | 29.5534 | 9.5957 | 7.8184 | 1.03E-02 | 2.06E-02 |
| sp\|O35857\|TI | Timm44 | TIM44_MOUSE | 2 | 13.2919 | 12.5711 | 16.0174 | 18.2558 | 22.7255 | 17.1383 | 13.9601 | 1.8177 | 19.3732 | 2.9565 | 1.3878 | 5.40E-02 | 7.87E-02 |
| sp\|Q9D1D4\|TI | Tmed10 | TMEDA_MOUS | 2 | 18.1826 | 19.1125 | 19.4622 | 17.7578 | 14.096 | 11.3889 | 18.9191 | 0.6614 | 14.4142 | 3.1964 | 0.7619 | 7.51E-02 | 1.05E-01 |

| | | | | | | | | | | | | | | | |
|---|---|---|---|---|---|---|---|---|---|---|---|---|---|---|---|
| sp\|Q61033\|LA | Tmpo | LAP2A_MOUSI | 2 | 18.1407 | 19.1904 | 17.0514 | 8.45101 | 14.5359 | 22.6306 | 18.1275 | 1.0696 | 15.2058 | 7.1135 | 0.8388 | 5.21E-01 | 5.67E-01 |
| sp\|Q01320\|TC | Top2a | TOP2A_MOUS | 2 | 11.3701 | 13.2884 | 11.5385 | 20.3581 | 19.4538 | 23.9911 | 12.0657 | 1.0623 | 21.2677 | 2.4015 | 1.7627 | 3.72E-03 | 9.86E-03 |
| sp\|Q8K1J6\|TR | Trnt1 | TRNT1_MOUSI | 2 | 29.3434 | 22.0125 | 34.6368 | 4.96 | 4.091 | 4.95629 | 28.6642 | 6.3395 | 4.6691 | 0.5006 | 0.1629 | 2.83E-03 | 8.17E-03 |
| sp\|Q3UDE2\|T | Ttll12 | TTL12_MOUSE | 2 | 26.9784 | 20.4977 | 31.6759 | 3.38827 | 7.06757 | 10.3922 | 26.3840 | 5.6128 | 6.9493 | 3.5035 | 0.2634 | 7.04E-03 | 1.57E-02 |
| sp\|P83887\|TB | Tubg1 | TBG1_MOUSE | 2 | 18.9315 | 25.6939 | 18.1121 | 11.4075 | 13.2863 | 12.5687 | 20.9125 | 4.1610 | 12.4208 | 0.9481 | 0.5939 | 2.61E-02 | 4.30E-02 |
| sp\|Q91W90\|T | Txndc5 | TXND5_MOUS | 2 | 10.7351 | 11.1866 | 12.3481 | 29.6846 | 19.5418 | 16.5037 | 11.4233 | 0.8321 | 21.9100 | 6.9022 | 1.9180 | 5.93E-02 | 8.55E-02 |
| sp\|Q9D883\|U | U2af1 | U2AF1_MOUS | 2 | 17.3607 | 24.5527 | 15.884 | 17.9101 | 15.1498 | 9.14287 | 19.2658 | 4.6377 | 14.0676 | 4.4827 | 0.7302 | 2.35E-01 | 2.89E-01 |
| sp\|Q80X50\|UI | Ubap2l | UBP2L_MOUSI | 2 | 28.4305 | 22.9656 | 17.5779 | 12.6068 | 11.8616 | 6.55748 | 22.9913 | 5.4263 | 10.3420 | 3.2986 | 0.4498 | 2.61E-02 | 4.29E-02 |
| sp\|Q9ES00\|UE | Ube4b | UBE4B_MOUS | 2 | 26.9283 | 32.6438 | 29.7412 | 2.33711 | 3.50075 | 4.84881 | 29.7711 | 2.8579 | 3.5622 | 1.2570 | 0.1197 | 1.30E-04 | 1.82E-03 |
| sp\|Q91ZJ5\|UG | Ugp2 | UGPA_MOUSE | 2 | 17.3603 | 18.4108 | 17.7707 | 22.37 | 13.3372 | 10.7509 | 17.8473 | 0.5294 | 15.4860 | 6.1003 | 0.8677 | 5.41E-01 | 5.84E-01 |
| sp\|Q99K46\|UI | Usp11 | UBP11_MOUS | 2 | 22.6688 | 40.0119 | 25.1854 | 2.83971 | 4.63788 | 4.65633 | 29.2887 | 9.3714 | 4.0446 | 1.0435 | 0.1381 | 9.76E-03 | 1.98E-02 |
| tr\|E9Q6R7\|E9 | Utrn | E9Q6R7_MOU | 2 | 12.211 | 12.2683 | 13.0138 | 17.6898 | 29.2664 | 15.5507 | 12.4977 | 0.4479 | 20.8356 | 7.3792 | 1.6672 | 1.22E-01 | 1.62E-01 |
| sp\|P40336\|VP | Vps26a | VP26A_MOUS | 2 | 19.9624 | 18.6685 | 23.3952 | 14.8031 | 11.101 | 12.0698 | 20.6754 | 2.4427 | 12.6580 | 1.9199 | 0.6122 | 1.11E-02 | 2.18E-02 |
| sp\|P0C7L0\|WI | Wipf3 | WIPF3_MOUSI | 2 | 31.8564 | 36.6485 | 26.5979 | 1.3525 | 1.6471 | 1.89761 | 31.7009 | 5.0271 | 1.6324 | 0.2729 | 0.0515 | 4.93E-04 | 3.27E-03 |
| sp\|Q6P1B1\|XI | Xpnpep1 | XPP1_MOUSE | 2 | 23.0098 | 18.9659 | 27.3671 | 6.51416 | 4.70382 | 19.4392 | 23.1143 | 4.2016 | 10.2191 | 8.0360 | 0.4421 | 6.95E-02 | 9.86E-02 |
| sp\|P23475\|XR | Xrcc6 | XRCC6_MOUSI | 2 | 20.7712 | 25.3255 | 21.4173 | 11.9258 | 12.7143 | 7.84582 | 22.5047 | 2.4642 | 10.8286 | 2.6131 | 0.4812 | 4.89E-03 | 1.21E-02 |
| sp\|Q9CQV8\|1 | Ywhab | 1433B_MOUSI | 2 | 22.8959 | 19.4206 | 24.7318 | 11.3342 | 6.57202 | 15.0455 | 22.3494 | 2.6974 | 10.9839 | 4.2476 | 0.4915 | 1.74E-02 | 3.09E-02 |
| sp\|O88291\|ZN | Znf326 | ZN326_MOUSI | 2 | 15.7041 | 30.9803 | 23.6149 | 10.2728 | 7.39839 | 12.0295 | 23.4331 | 7.6397 | 9.9002 | 2.3379 | 0.4225 | 4.27E-02 | 6.43E-02 |
| sp\|Q9CQE8\|CN166_MOUSE | | CN166_MOUS | 2 | 14.43 | 18.0526 | 8.95937 | 22.0879 | 24.0671 | 12.4031 | 13.8140 | 4.5778 | 19.5194 | 6.2418 | 1.4130 | 2.71E-01 | 3.27E-01 |
| sp\|Q8C1B7\|SE | 11-Sep | SEP11_MOUSE | 1 | 18.4982 | 14.4991 | 14.6236 | 24.7598 | 13.7863 | 13.8331 | 15.8736 | 2.2738 | 17.4597 | 6.3221 | 1.0999 | 7.04E-01 | 7.37E-01 |
| tr\|Q9CPN9\|Q | 2210010C04Rik | Q9CPN9_MOU | 1 | 14.373 | 11.4908 | 6.89249 | 30.5422 | 14.5903 | 22.1112 | 10.9188 | 3.7729 | 22.4146 | 7.9803 | 2.0528 | 8.71E-02 | 1.20E-01 |
| sp\|P55096\|AB | Abcd3 | ABCD3_MOUS | 1 | 16.9529 | 19.6508 | 16.3863 | 11.4137 | 20.9139 | 14.6824 | 17.6633 | 1.7444 | 15.6700 | 4.8265 | 0.8871 | 5.38E-01 | 5.83E-01 |
| sp\|Q99LE6\|AB | Abcf2 | ABCF2_MOUSI | 1 | 24.906 | 27.4248 | 26.1022 | 5.45088 | 8.75659 | 7.35955 | 26.1443 | 1.2599 | 7.1890 | 1.6594 | 0.2750 | 9.48E-05 | 1.63E-03 |
| sp\|Q8K268\|AE | Abcf3 | ABCF3_MOUSI | 1 | 20.1029 | 28.49 | 25.7124 | 6.90807 | 7.96239 | 10.8243 | 24.7684 | 4.2725 | 8.5649 | 2.0264 | 0.3458 | 4.04E-03 | 1.04E-02 |
| sp\|Q9CQR4\|A | Acot13 | ACO13_MOUS | 1 | 19.7895 | 18.3134 | 18.5162 | 18.5851 | 14.2613 | 10.5344 | 18.8730 | 0.8001 | 14.4603 | 4.0290 | 0.7662 | 1.36E-01 | 1.78E-01 |
| sp\|P41216\|AC | Acsl1 | ACSL1_MOUSI | 1 | 26.0002 | 20.6814 | 32.8323 | 5.17697 | 4.35635 | 10.9527 | 26.5046 | 6.0911 | 6.8287 | 3.5950 | 0.2576 | 8.53E-03 | 1.80E-02 |
| sp\|Q9Z2N8\|A | Actl6a | ACL6A_MOUSI | 1 | 20.893 | 31.7472 | 35.5611 | 0 | 5.03444 | 6.76426 | 29.4004 | 7.6104 | 3.9329 | 3.5141 | 0.1338 | 6.25E-03 | 1.43E-02 |
| sp\|P54923\|AD | Adprh | ADPRH_MOUS | 1 | 25.6534 | 23.7585 | 27.1125 | 11.3853 | 6.51299 | 5.57737 | 25.5081 | 1.6817 | 7.8252 | 3.1184 | 0.3068 | 9.85E-04 | 4.67E-03 |
| sp\|Q9JKV1\|AD | Adrm1 | ADRM1_MOU | 1 | 24.2305 | 22.4125 | 18.9076 | 11.57 | 14.0825 | 8.79698 | 21.8502 | 2.7056 | 11.4832 | 2.6438 | 0.5255 | 8.99E-03 | 1.86E-02 |
| sp\|P46664\|PU | Adss | PURA2_MOUS | 1 | 22.6794 | 23.4458 | 36.4522 | 4.4414 | 5.86171 | 7.11954 | 27.5258 | 7.7400 | 5.8076 | 1.3399 | 0.2110 | 8.72E-03 | 1.83E-02 |
| sp\|Q8K2K6\|A | Agfg1 | AGFG1_MOUS | 1 | 32.1156 | 22.7543 | 25.9876 | 5.83855 | 5.91464 | 7.38932 | 26.9525 | 4.7547 | 6.3808 | 0.8742 | 0.2367 | 1.81E-03 | 6.13E-03 |
| sp\|Q80SW1\|S | Ahcyl1 | SAHH2_MOUS | 1 | 11.6685 | 18.565 | 10.1618 | 22.2326 | 19.1189 | 18.2532 | 13.4651 | 4.4804 | 19.8682 | 2.0929 | 1.4755 | 8.84E-02 | 1.22E-01 |
| sp\|Q9Z0X1\|AI | Aifm1 | AIFM1_MOUSI | 1 | 11.4186 | 15.0238 | 37.4968 | 5.62687 | 8.13313 | 22.3007 | 21.3131 | 14.1310 | 12.0202 | 8.9909 | 0.5640 | 3.91E-01 | 4.48E-01 |
| sp\|Q9JII6\|AK1 | Akr1a1 | AK1A1_MOUS | 1 | 19.3737 | 15.5592 | 19.4276 | 21.7132 | 14.1876 | 9.73874 | 18.1202 | 2.2180 | 15.2132 | 6.0528 | 0.8396 | 4.78E-01 | 5.28E-01 |
| sp\|P10518\|HE | Alad | HEM2_MOUSE | 1 | 24.5548 | 21.6687 | 28.0698 | 12.6727 | 4.4156 | 8.61842 | 24.7644 | 3.2057 | 8.5689 | 4.1288 | 0.3460 | 5.82E-03 | 1.38E-02 |
| sp\|O08583\|TH | Alyref | THOC4_MOUS | 1 | 29.4997 | 23.7227 | 39.0883 | 0 | 3.56162 | 4.12775 | 30.7702 | 7.7612 | 2.5631 | 2.2377 | 0.0833 | 3.77E-03 | 9.93E-03 |
| sp\|P97822\|AN | Anp32e | AN32E_MOUS | 1 | 37.0098 | 19.5925 | 35.0674 | 2.2924 | 1.98426 | 4.05351 | 30.5566 | 9.5447 | 2.7767 | 1.1164 | 0.0909 | 7.45E-03 | 1.64E-02 |
| sp\|P22892\|AP | Ap1g1 | AP1G1_MOUS | 1 | 21.7753 | 21.6684 | 29.405 | 6.79559 | 7.36336 | 12.9924 | 24.2829 | 4.4362 | 9.0505 | 3.4256 | 0.3727 | 9.26E-03 | 1.90E-02 |
| sp\|P12023\|A4 | App | A4_MOUSE An | 1 | 5.11357 | 9.85599 | 5.20356 | 46.9696 | 23.7379 | 9.11932 | 6.7244 | 2.7124 | 26.6089 | 19.0878 | 3.9571 | 1.49E-01 | 1.92E-01 |
| tr\|A2RSX9\|A2 | Arfip1 | A2RSX9_MOU | 1 | 23.9339 | 35.8588 | 10.5569 | 13.315 | 8.55776 | 7.77777 | 23.4499 | 12.6579 | 9.8835 | 2.9972 | 0.4215 | 1.45E-01 | 1.88E-01 |
| sp\|Q5FWK3\|R | Arhgap1 | RHG01_MOUS | 1 | 22.6725 | 16.9084 | 26.902 | 8.30801 | 12.4276 | 12.7816 | 22.1610 | 5.0164 | 11.1724 | 2.4869 | 0.5041 | 2.73E-02 | 4.43E-02 |
| sp\|Q61599\|GI | Arhgdib | GDIR2_MOUSI | 1 | 11.5259 | 28.5543 | 56.9273 | 0 | 0 | 2.9925 | 32.3358 | 22.9357 | 0.9975 | 1.7277 | 0.0308 | 7.77E-02 | 1.08E-01 |
| sp\|Q8VEH3\|AI | Arl8a | ARL8A_MOUSI | 1 | 21.3925 | 22.3678 | 28.7339 | 12.8999 | 7.2846 | 7.32125 | 24.1647 | 3.9869 | 9.1686 | 3.2315 | 0.3794 | 7.17E-03 | 1.59E-02 |
| sp\|Q8VD46\|A | Asz1 | ASZ1_MOUSE | 1 | 26.5922 | 29.7373 | 27.34 | 6.93785 | 2.6127 | 6.77993 | 27.8898 | 1.6431 | 5.4435 | 2.4528 | 0.1952 | 1.92E-04 | 2.08E-03 |
| sp\|Q8BH66\|A | Atl1 | ATLA1_MOUSI | 1 | 14.1675 | 11.7801 | 16.506 | 26.9781 | 16.4056 | 14.1628 | 14.1512 | 2.3630 | 19.1822 | 6.8440 | 1.3555 | 2.95E-01 | 3.54E-01 |
| sp\|Q06185\|AT | Atp5i | ATP5I_MOUSE | 1 | 29.617 | 32.5144 | 31.9087 | 0 | 0 | 5.95988 | 31.3467 | 1.5283 | 1.9866 | 3.4409 | 0.0634 | 1.74E-04 | 1.97E-03 |
| sp\|Q9Z1G4\|VI | Atp6v0a1 | VPP1_MOUSE | 1 | 24.8904 | 24.6798 | 27.8615 | 6.39386 | 6.12582 | 10.0487 | 25.8106 | 1.7793 | 7.5228 | 2.1916 | 0.2915 | 3.59E-04 | 2.73E-03 |
| sp\|P51863\|VA | Atp6v0d1 | VA0D1_MOUS | 1 | 23.6478 | 26.8333 | 22.8559 | 12.153 | 7.57289 | 6.93717 | 24.4457 | 2.1053 | 8.8877 | 2.8457 | 0.3636 | 1.60E-03 | 5.88E-03 |
| sp\|O70305\|AT | Atxn2 | ATX2_MOUSE | 1 | 19.8188 | 23.4421 | 14.2858 | 16.6175 | 15.8038 | 10.0319 | 19.1822 | 4.6112 | 14.1511 | 3.5904 | 0.7377 | 2.10E-01 | 2.60E-01 |
| sp\|Q9Z1R2\|B | Bag6 | BAG6_MOUSE | 1 | 23.2195 | 29.5445 | 24.3185 | 7.50815 | 5.35341 | 10.0559 | 25.6942 | 3.3795 | 7.6392 | 2.3540 | 0.2973 | 1.61E-03 | 5.92E-03 |
| sp\|Q61334\|B | Bcap29 | BAP29_MOUSI | 1 | 23.7965 | 27.0548 | 21.87 | 11.733 | 7.4794 | 8.06632 | 24.2404 | 2.6208 | 9.0929 | 2.3051 | 0.3751 | 1.68E-03 | 5.94E-03 |
| sp\|Q80XN0\|BI | Bdh1 | BDH_MOUSE I | 1 | 30.4299 | 33.8352 | 25.376 | 4.26534 | 2.05821 | 4.03535 | 29.8804 | 4.2563 | 3.4530 | 1.2134 | 0.1156 | 4.93E-04 | 3.27E-03 |
| sp\|Q923D2\|BI | Blvrb | BLVRB_MOUSI | 1 | 17.0248 | 17.6313 | 15.2145 | 20.9598 | 12.0054 | 17.1642 | 16.6235 | 1.2574 | 16.7098 | 4.4945 | 1.0052 | 9.76E-01 | 9.79E-01 |
| sp\|Q9Z0S1\|BP | Bpnt1 | BPNT1_MOUS | 1 | 27.2579 | 23.367 | 34.2549 | 3.51169 | 5.03767 | 6.57082 | 28.2933 | 5.5173 | 5.0401 | 1.5296 | 0.1781 | 2.15E-03 | 6.84E-03 |
| sp\|Q9WVA3\|E | Bub3 | BUB3_MOUSE | 1 | 20.3514 | 23.0929 | 20.4239 | 13.8406 | 13.1813 | 9.10997 | 21.2894 | 1.5623 | 12.0440 | 2.5622 | 0.5657 | 5.94E-03 | 1.39E-02 |
| sp\|P62204\|CA | Calm1 | CALM_MOUSE | 1 | 22.8334 | 13.8819 | 29.143 | 6.10323 | 9.1647 | 18.8737 | 21.9528 | 7.6686 | 11.3805 | 6.6674 | 0.5184 | 1.46E-01 | 1.89E-01 |
| sp\|Q8BW96\|K | Camk1d | KCC1D_MOUS | 1 | 28.7352 | 20.7872 | 35.7676 | 0 | 5.41773 | 9.29225 | 28.4300 | 7.4949 | 4.9033 | 4.6674 | 0.1725 | 9.92E-03 | 2.00E-02 |
| sp\|P70677\|CA | Casp3 | CASP3_MOUSI | 1 | 20.7292 | 18.6794 | 21.8113 | 14.9463 | 19.3109 | 4.52295 | 20.4066 | 1.5907 | 12.9267 | 7.5980 | 0.6335 | 1.70E-01 | 2.16E-01 |
| sp\|P51125\|IC | Cast | ICAL_MOUSE ( | 1 | 14.1495 | 7.07136 | 13.3119 | 40.0885 | 10.0543 | 15.3244 | 11.5109 | 3.8675 | 21.8224 | 16.0369 | 1.8958 | 3.40E-01 | 4.00E-01 |
| sp\|Q08024\|PE | Cbfb | PEBB_MOUSE | 1 | 27.6604 | 22.2239 | 23.4356 | 10.3982 | 7.02299 | 9.25891 | 24.4400 | 2.8540 | 8.8934 | 1.7170 | 0.3639 | 1.27E-03 | 5.27E-03 |
| sp\|Q9D024\|C | Ccdc47 | CCD47_MOUS | 1 | 21.3934 | 27.1787 | 22.2731 | 6.3622 | 8.77821 | 14.0143 | 23.6151 | 3.1174 | 9.7182 | 3.9117 | 0.4115 | 8.57E-03 | 1.81E-02 |
| sp\|P41731\|CD | Cd63 | CD63_MOUSE | 1 | 26.2058 | 14.3456 | 28.3189 | 13.5084 | 10.0148 | 7.6065 | 22.9568 | 7.5320 | 10.3766 | 2.9675 | 0.4520 | 5.46E-02 | 7.93E-02 |
| sp\|P60766\|CD | Cdc42 | CDC42_MOUS | 1 | 18.286 | 17.5657 | 22.5089 | 19.5526 | 11.6796 | 10.4072 | 19.4535 | 2.6704 | 13.8798 | 4.9538 | 0.7135 | 1.61E-01 | 2.06E-01 |

| | | | | | | | | | | | | | | | | |
|---|---|---|---|---|---|---|---|---|---|---|---|---|---|---|---|---|
| sp\|P24788\|CD | Cdk11b | CD11B_MOUS | 1 | 21.3521 | 22.3348 | 23.8268 | 9.89617 | 0 | 22.5902 | 22.5046 | 1.2461 | 10.8288 | 11.3239 | 0.4812 | 1.51E-01 | 1.94E-01 |
| sp\|Q99LM2\|C | Cdk5rap3 | CK5P3_MOUSI | 1 | 24.2551 | 27.7345 | 32.73 | 0 | 0 | 15.2804 | 28.2399 | 4.2600 | 5.0935 | 8.8221 | 0.1804 | 1.49E-02 | 2.74E-02 |
| sp\|Q99L43\|CD | Cds2 | CDS2_MOUSE | 1 | 28.8968 | 27.0781 | 29.319 | 0 | 6.56441 | 8.14167 | 28.4313 | 1.1908 | 4.9020 | 4.3179 | 0.1724 | 8.09E-04 | 4.27E-03 |
| sp\|Q4VAA2\|C | Cdv3 | CDV3_MOUSE | 1 | 23.5053 | 24.0885 | 22.9083 | 10.7617 | 4.21803 | 14.5181 | 23.5007 | 0.5901 | 9.8326 | 5.2125 | 0.4184 | 1.07E-02 | 2.13E-02 |
| tr\|E9Q3P4\|E9 | Cenpf | E9Q3P4_MOU | 1 | 6.82117 | 7.47055 | 17.0025 | 16.3757 | 11.1967 | 41.1334 | 10.4314 | 5.7000 | 22.9019 | 15.9999 | 2.1955 | 2.72E-01 | 3.29E-01 |
| sp\|Q9D6K9\|C | Cers5 | CERS5_MOUSI | 1 | 27.1426 | 18.3582 | 24.9783 | 9.34115 | 9.68796 | 10.4918 | 23.4930 | 4.5767 | 9.8403 | 0.5903 | 0.4189 | 6.86E-03 | 1.54E-02 |
| sp\|Q9CRB9\|C | Chchd3 | CHCH3_MOUS | 1 | 22.6636 | 31.3964 | 15.4785 | 13.9173 | 9.50714 | 7.03705 | 23.1795 | 7.9715 | 10.1538 | 3.4854 | 0.4381 | 6.05E-02 | 8.70E-02 |
| sp\|Q6PDQ2\|C | Chd4 | CHD4_MOUSE | 1 | 25.4389 | 23.8946 | 20.5761 | 12.6976 | 7.81383 | 9.57901 | 23.3032 | 2.4848 | 10.0301 | 2.4729 | 0.4304 | 2.80E-03 | 8.12E-03 |
| sp\|Q9D1P4\|C | Chordc1 | CHRD1_MOUS | 1 | 22.06 | 21.2453 | 32.072 | 9.64814 | 7.82884 | 7.1456 | 25.1258 | 6.0294 | 8.2075 | 1.2935 | 0.3267 | 8.96E-03 | 1.86E-02 |
| sp\|Q9JHS4\|CL | Clpx | CLPX_MOUSE | 1 | 28.4713 | 20.3637 | 30.2444 | 11.921 | 3.74838 | 5.25125 | 26.3598 | 5.2679 | 6.9735 | 4.3500 | 0.2646 | 7.96E-03 | 1.73E-02 |
| sp\|Q9DBP5\|K | Cmpk1 | KCY_MOUSE U | 1 | 26.0937 | 20.3423 | 21.0989 | 14.6139 | 9.5994 | 8.25173 | 22.5116 | 3.1251 | 10.8217 | 3.3526 | 0.4807 | 1.15E-02 | 2.24E-02 |
| sp\|Q9D1A2\|C | Cndp2 | CNDP2_MOUS | 1 | 16.6818 | 16.452 | 19.8914 | 19.2376 | 14.4308 | 13.3064 | 17.6751 | 1.9228 | 15.6583 | 3.1504 | 0.8859 | 3.98E-01 | 4.53E-01 |
| sp\|Q61245\|C | Col11a1 | COBA1_MOUS | 1 | 2.82642 | 1.01757 | 1.8937 | 32.2365 | 47.1772 | 14.8486 | 1.9126 | 0.9046 | 31.4208 | 16.1797 | 16.4286 | 3.44E-02 | 5.35E-02 |
| sp\|Q01149\|C | Col1a2 | CO1A2_MOUS | 1 | 3.30314 | 1.64893 | 2.26321 | 35.4358 | 41.0584 | 16.2904 | 2.4051 | 0.8362 | 30.9282 | 12.9847 | 12.8595 | 1.92E-02 | 3.37E-02 |
| sp\|P08121\|C | Col3a1 | CO3A1_MOUS | 1 | 2.00544 | 0.499149 | 0.80513 | 37.7583 | 40.2906 | 18.6413 | 1.1032 | 0.7962 | 32.2301 | 11.8361 | 29.2140 | 1.05E-02 | 2.08E-02 |
| sp\|O35864\|CS | Cops5 | CSN5_MOUSE | 1 | 26.1817 | 20.3245 | 27.669 | 7.39575 | 8.85368 | 9.57542 | 24.7251 | 3.8829 | 8.6083 | 1.1104 | 0.3482 | 2.30E-03 | 7.09E-03 |
| sp\|O88545\|CS | Cops6 | CSN6_MOUSE | 1 | 23.8875 | 23.5911 | 18.3792 | 14.4206 | 10.5923 | 9.1292 | 21.9526 | 3.0982 | 11.3807 | 2.7324 | 0.5184 | 1.14E-02 | 2.23E-02 |
| sp\|O89053\|C | Coro1a | COR1A_MOUS | 1 | 10.9324 | 12.3084 | 13.1855 | 27.4923 | 21.8169 | 14.2645 | 12.1421 | 1.1357 | 21.1912 | 6.6361 | 1.7453 | 8.04E-02 | 1.12E-01 |
| sp\|Q9WUM3\| | Coro1b | COR1B_MOUS | 1 | 13.1792 | 12.9631 | 15.9748 | 22.0743 | 20.2621 | 15.5465 | 14.0390 | 1.6799 | 19.2943 | 3.3698 | 1.3743 | 7.30E-02 | 1.03E-01 |
| sp\|P19783\|C | Cox4i1 | COX41_MOUS | 1 | 22.8111 | 26.0469 | 23.3699 | 11.6302 | 9.22777 | 6.9141 | 24.0760 | 1.7296 | 9.2574 | 2.3582 | 0.3845 | 9.29E-04 | 4.58E-03 |
| sp\|P59108\|CP | Cpne2 | CPNE2_MOUS | 1 | 13.0056 | 9.66753 | 18.1471 | 23.9575 | 15.0281 | 20.1941 | 13.6067 | 4.2716 | 19.7266 | 4.4830 | 1.4498 | 1.62E-01 | 2.07E-01 |
| sp\|P47934\|CA | Crat | CACP_MOUSE | 1 | 23.3528 | 27.8446 | 35.7926 | 0 | 7.10877 | 5.90124 | 28.9967 | 6.2994 | 4.3367 | 3.8039 | 0.1496 | 4.38E-03 | 1.12E-02 |
| sp\|P97315\|CS | Csrp1 | CSRP1_MOUSI | 1 | 11.1087 | 10.8144 | 11.9745 | 21.5557 | 26.8293 | 17.7175 | 11.2992 | 0.6031 | 22.0342 | 4.5747 | 1.9501 | 1.57E-02 | 2.85E-02 |
| sp\|Q9D1D6\|C | Cthrc1 | CTHR1_MOUS | 1 | 28.5299 | 35.3189 | 24.7606 | 2.8448 | 4.34287 | 4.2029 | 29.5365 | 5.3506 | 3.7969 | 0.8275 | 0.1285 | 1.19E-03 | 5.07E-03 |
| sp\|Q9CWL8\|C | Ctnnbl1 | CTBL1_MOUSE | 1 | 31.878 | 18.0202 | 30.0706 | 8.50721 | 3.96311 | 7.5609 | 26.6563 | 7.5335 | 6.6771 | 2.3975 | 0.2505 | 1.19E-02 | 2.29E-02 |
| sp\|Q9WUU7\| | Ctsz | CATZ_MOUSE | 1 | 13.1747 | 16.3165 | 10.0167 | 28.266 | 14.7216 | 17.5045 | 13.1693 | 3.1499 | 20.1640 | 7.1531 | 1.5311 | 1.96E-01 | 2.45E-01 |
| sp\|Q99LJ0\|CT | Cttnbp2nl | CT2NL_MOUSI | 1 | 28.5019 | 22.1742 | 23.4737 | 9.38329 | 6.94097 | 9.52594 | 24.7166 | 3.3419 | 8.6167 | 1.4530 | 0.3486 | 1.57E-03 | 5.82E-03 |
| sp\|Q9CQX2\|C | Cyb5b | CYB5B_MOUSI | 1 | 27.6972 | 30.8894 | 26.6013 | 5.1556 | 2.80869 | 6.84784 | 28.3960 | 2.2278 | 4.9374 | 2.0284 | 0.1739 | 1.75E-04 | 1.97E-03 |
| sp\|Q9D0M3\|C | Cyc1 | CY1_MOUSE C | 1 | 14.7716 | 20.5077 | 23.1073 | 15.0766 | 12.6564 | 13.8804 | 19.4622 | 4.2651 | 13.8711 | 1.2101 | 0.7127 | 9.43E-02 | 1.29E-01 |
| sp\|Q7TMB8\|C | Cyfip1 | CYFP1_MOUSE | 1 | 16.6622 | 21.4355 | 29.8487 | 14.5288 | 9.98255 | 7.54214 | 22.6488 | 6.6765 | 10.6845 | 3.5458 | 0.4717 | 5.18E-02 | 7.60E-02 |
| sp\|Q8K0C4\|C | Cyp51a1 | CP51A_MOUSI | 1 | 22.571 | 20.7518 | 30.3305 | 9.19856 | 3.80902 | 13.3392 | 24.5511 | 5.0871 | 8.7823 | 4.7787 | 0.3577 | 1.73E-02 | 3.09E-02 |
| sp\|O08967\|CY | Cyth3 | CYH3_MOUSE | 1 | 19.4197 | 28.7692 | 29.2962 | 7.42131 | 4.98158 | 10.1121 | 25.8284 | 5.5563 | 7.5050 | 2.5663 | 0.2906 | 6.58E-03 | 1.50E-02 |
| sp\|Q9D172\|ES | D10Jhu81e | ES1_MOUSE E | 1 | 19.6079 | 24.4538 | 15.3012 | 18.6418 | 9.88114 | 12.114 | 19.7876 | 4.5789 | 13.5456 | 4.5524 | 0.6846 | 1.69E-01 | 2.15E-01 |
| sp\|Q9CPT4\|CS | D17Wsu104e | CS010_MOUSE | 1 | 27.5086 | 22.5541 | 28.1868 | 0 | 7.50948 | 14.241 | 26.0832 | 3.0750 | 7.2502 | 7.1240 | 0.2780 | 1.37E-02 | 2.56E-02 |
| sp\|Q8VC30\|D | Dak | DHAK_MOUSE | 1 | 25.7033 | 26.8092 | 24.1334 | 9.84118 | 6.62012 | 6.89275 | 25.5486 | 1.3446 | 7.7847 | 1.7862 | 0.3047 | 1.62E-04 | 1.93E-03 |
| sp\|Q8BHC4\|D | Dcakd | DCAKD_MOUS | 1 | 26.4551 | 28.769 | 26.5053 | 3.01339 | 9.12592 | 6.13131 | 27.2431 | 1.3217 | 6.0902 | 3.0565 | 0.2236 | 3.88E-04 | 2.88E-03 |
| sp\|Q99LD8\|D | Ddah2 | DDAH2_MOUS | 1 | 13.0458 | 29.6674 | 46.6776 | 0 | 0 | 10.6091 | 29.7969 | 16.8163 | 3.5364 | 6.1252 | 0.1187 | 6.39E-02 | 9.12E-02 |
| sp\|Q8K363\|D | Ddx18 | DDX18_MOUS | 1 | 21.9844 | 23.5584 | 18.7672 | 11.889 | 14.2022 | 9.59868 | 21.4367 | 2.4421 | 11.8966 | 2.3018 | 0.5550 | 7.91E-03 | 1.72E-02 |
| sp\|Q7TNV0\|D | Dek | DEK_MOUSE P | 1 | 21.3436 | 12.8291 | 16.381 | 19.4774 | 17.3474 | 12.6215 | 16.8512 | 4.2767 | 16.4821 | 3.5089 | 0.9781 | 9.14E-01 | 9.28E-01 |
| sp\|P00375\|DY | Dhfr | DYR_MOUSE D | 1 | 14.9177 | 28.9752 | 53.6483 | 0 | 0 | 2.45882 | 32.5137 | 19.6063 | 0.8196 | 1.4196 | 0.0252 | 4.92E-02 | 7.28E-02 |
| sp\|Q99LB2\|D | Dhrs4 | DHRS4_MOUS | 1 | 10.8155 | 10.9679 | 4.38159 | 38.1212 | 23.48 | 12.2337 | 8.7217 | 3.7594 | 24.6116 | 12.9808 | 2.8219 | 1.11E-01 | 1.50E-01 |
| sp\|Q8VHK9\|D | Dhx36 | DHX36_MOUS | 1 | 22.3659 | 29.9746 | 37.4939 | 0 | 5.13018 | 5.03532 | 29.9448 | 7.5640 | 3.3885 | 2.9349 | 0.1132 | 4.77E-03 | 1.19E-02 |
| sp\|Q9CSH3\|R | Dis3 | RRP44_MOUSI | 1 | 20.903 | 29.0481 | 29.1747 | 5.12186 | 7.22633 | 8.52605 | 26.3753 | 4.7395 | 6.9581 | 1.7179 | 0.2638 | 2.62E-03 | 7.81E-03 |
| sp\|O54946\|D | Dnajb6 | DNJB6_MOUSI | 1 | 20.7301 | 11.944 | 14.7526 | 8.98483 | 11.5871 | 32.0015 | 15.8089 | 4.4873 | 17.5245 | 12.6048 | 1.1085 | 8.35E-01 | 8.60E-01 |
| tr\|G3X922\|G3 | Dnajc13 | G3X922_MOU | 1 | 4.22981 | 0 | 4.1832 | 13.5102 | 57.6975 | 20.3793 | 2.8043 | 2.4287 | 30.5290 | 23.7780 | 10.8864 | 1.15E-01 | 1.54E-01 |
| sp\|Q80VJ3\|D | Dnph1 | DNPH1_MOUS | 1 | 16.5246 | 16.3201 | 11.3719 | 30.7347 | 17.8157 | 7.233 | 14.7389 | 2.9177 | 18.5945 | 11.7702 | 1.2616 | 6.11E-01 | 6.51E-01 |
| sp\|Q8R1A4\|D | Dock7 | DOCK7_MOUS | 1 | 25.4017 | 24.0254 | 22.7698 | 14.9486 | 5.0148 | 7.83974 | 24.0656 | 1.3164 | 9.2677 | 5.1185 | 0.3851 | 8.34E-03 | 1.78E-02 |
| sp\|O70152\|D | Dpm1 | DPM1_MOUSE | 1 | 22.6221 | 19.0967 | 17.9819 | 8.27216 | 15.9667 | 16.0604 | 19.9002 | 2.4222 | 13.4331 | 4.4697 | 0.6750 | 9.23E-02 | 1.27E-01 |
| sp\|P32233\|DR | Drg1 | DRG1_MOUSE | 1 | 19.171 | 25.4155 | 18.7329 | 15.7033 | 12.333 | 8.64441 | 21.1065 | 3.7382 | 12.2269 | 3.5306 | 0.5793 | 4.03E-02 | 6.12E-02 |
| sp\|Q9QXB9\|D | Drg2 | DRG2_MOUSE | 1 | 25.1952 | 24.7679 | 28.8294 | 6.59814 | 7.58127 | 7.02811 | 26.2642 | 2.2318 | 7.0692 | 0.4928 | 0.2692 | 1.30E-04 | 1.82E-03 |
| sp\|Q6PDL0\|D | Dync1li2 | DC1L2_MOUSI | 1 | 17.7838 | 25.4313 | 16.4567 | 19.7635 | 11.1641 | 9.40062 | 19.8906 | 4.8440 | 13.4427 | 5.5445 | 0.6758 | 2.04E-01 | 2.53E-01 |
| sp\|Q9JMG1\|E | Edf1 | EDF1_MOUSE | 1 | 8.32821 | 4.82759 | 17.7408 | 3.64488 | 8.43349 | 57.025 | 10.2989 | 6.6784 | 23.0345 | 29.5339 | 2.2366 | 5.07E-01 | 5.55E-01 |
| sp\|Q8BL66\|EE | Eea1 | EEA1_MOUSE | 1 | 24.4369 | 21.7905 | 24.6518 | 7.3702 | 8.9602 | 12.7905 | 23.6264 | 1.5936 | 9.7070 | 2.7862 | 0.4109 | 1.68E-03 | 5.94E-03 |
| sp\|Q61749\|EI | Eif2b4 | EI2BD_MOUSE | 1 | 20.6521 | 20.5284 | 18.7031 | 0 | 23.6085 | 16.5079 | 19.9612 | 1.0913 | 13.3721 | 12.1126 | 0.6699 | 4.01E-01 | 4.56E-01 |
| sp\|Q3UGC7\|E | Eif3j1 | EI3JA_MOUSE | 1 | 32.1765 | 22.3179 | 23.4676 | 4.66181 | 4.58972 | 12.7864 | 25.9873 | 5.3907 | 7.3460 | 4.7117 | 0.2827 | 1.07E-02 | 2.13E-02 |
| sp\|Q91VC3\|IF | Eif4a3 | IF4A3_MOUSE | 1 | 19.9848 | 24.2249 | 18.9667 | 14.1787 | 13.2309 | 9.41402 | 21.0588 | 2.7888 | 12.2745 | 2.5222 | 0.5829 | 1.55E-02 | 2.82E-02 |
| sp\|P63073\|IF4 | Eif4e | IF4E_MOUSE E | 1 | 21.6806 | 21.0173 | 25.5001 | 12.2456 | 10.7263 | 8.83 | 22.7327 | 2.4195 | 10.6006 | 1.7113 | 0.4663 | 2.09E-03 | 6.77E-03 |
| sp\|Q80Y81\|R | Elac2 | RNZ2_MOUSE | 1 | 29.5235 | 23.0776 | 30.5458 | 6.18662 | 3.58627 | 7.08021 | 27.7156 | 4.0490 | 5.6177 | 1.8151 | 0.2027 | 9.93E-04 | 4.69E-03 |
| sp\|Q9EP72\|E | Emc7 | EMC7_MOUSE | 1 | 22.9964 | 23.2749 | 26.8773 | 9.56152 | 3.96638 | 13.3234 | 24.3829 | 2.1647 | 8.9504 | 4.7083 | 0.3671 | 6.71E-03 | 1.51E-02 |
| sp\|Q3UMY5\|E | Eml4 | EMAL4_MOUS | 1 | 18.5813 | 24.6322 | 21.1291 | 16.7356 | 9.86454 | 9.05729 | 21.4475 | 3.0380 | 11.8858 | 4.2194 | 0.5542 | 3.34E-02 | 5.22E-02 |
| sp\|Q03173\|E | Enah | ENAH_MOUSE | 1 | 19.5975 | 18.3253 | 20.4893 | 8.64711 | 11.9419 | 20.9989 | 19.4707 | 1.0876 | 13.8626 | 6.3960 | 0.7120 | 2.09E-01 | 2.59E-01 |
| sp\|Q8R0W0\|E | Eppk1 | EPIPL_MOUSE | 1 | 6.79001 | 3.51343 | 5.28771 | 23.2388 | 40.7355 | 20.4346 | 5.1971 | 1.6402 | 28.1363 | 11.0009 | 5.4139 | 2.33E-02 | 3.93E-02 |
| sp\|Q9DC16\|E | Ergic1 | ERGI1_MOUSE | 1 | 20.1888 | 25.1019 | 23.421 | 14.3276 | 6.14541 | 10.8152 | 22.9039 | 2.4970 | 10.4294 | 4.1047 | 0.4554 | 1.08E-02 | 2.14E-02 |

| | | | | | | | | | | | | | | | | |
|---|---|---|---|---|---|---|---|---|---|---|---|---|---|---|---|---|
| sp\|Q8BFZ9\|ER | Erlin2 | ERLN2_MOUSI | 1 | 20.1509 | 27.6745 | 18.4125 | 13.4557 | 10.3079 | 9.99853 | 22.0793 | 4.9229 | 11.2540 | 1.9130 | 0.5097 | 2.38E-02 | 3.98E-02 |
| sp\|Q3UVK0\|EI | Ermp1 | ERMP1_MOUS | 1 | 23.2213 | 22.2988 | 28.3488 | 7.55562 | 10.3428 | 8.23281 | 24.6230 | 3.2595 | 8.7104 | 1.4537 | 0.3538 | 1.51E-03 | 5.71E-03 |
| sp\|P57759\|ER | Erp29 | ERP29_MOUSI | 1 | 23.7147 | 18.1077 | 24.3057 | 17.8702 | 8.46857 | 7.53305 | 22.0427 | 3.4206 | 11.2906 | 5.7173 | 0.5122 | 4.90E-02 | 7.27E-02 |
| sp\|P54731\|FA | Faf1 | FAF1_MOUSE | 1 | 29.0809 | 24.6822 | 37.8818 | 0 | 4.34083 | 4.01425 | 30.5483 | 6.7210 | 2.7850 | 2.4174 | 0.0912 | 2.54E-03 | 7.61E-03 |
| sp\|Q3UW53\|N | Fam129a | NIBAN_MOUS | 1 | 17.6095 | 28.1882 | 27.7296 | 6.93218 | 4.83755 | 14.703 | 24.5091 | 5.9796 | 8.8242 | 5.1978 | 0.3600 | 2.66E-02 | 4.34E-02 |
| sp\|Q8R1F1\|NI | Fam129b | NIBL1_MOUSE | 1 | 12.1621 | 12.6525 | 16.9466 | 26.341 | 18.9902 | 12.9076 | 13.9204 | 2.6322 | 19.4129 | 6.7267 | 1.3946 | 2.58E-01 | 3.14E-01 |
| sp\|Q80VD1\|F/ | Fam98b | FA98B_MOUSI | 1 | 11.4757 | 24.1351 | 33.9587 | 11.1994 | 11.6606 | 7.57042 | 23.1898 | 11.2713 | 10.1435 | 2.2402 | 0.4374 | 1.21E-01 | 1.60E-01 |
| sp\|P62862\|RS | Fau | RS30_MOUSE | 1 | 18.2568 | 20.2899 | 11.844 | 19.6607 | 18.9734 | 10.9753 | 16.7969 | 4.4082 | 16.5365 | 4.8284 | 0.9845 | 9.48E-01 | 9.58E-01 |
| sp\|Q61578\|AI | Fdxr | ADRO_MOUSE | 1 | 28.3644 | 32.2152 | 34.0383 | 0 | 0 | 5.38215 | 31.5393 | 2.8967 | 1.7941 | 3.1074 | 0.0569 | 2.65E-04 | 2.40E-03 |
| sp\|P45878\|FK | Fkbp2 | FKBP2_MOUSI | 1 | 26.7125 | 35.0657 | 20.8091 | 6.41079 | 4.56813 | 6.43371 | 27.5291 | 7.1633 | 5.8042 | 1.0705 | 0.2108 | 6.54E-03 | 1.49E-02 |
| sp\|Q64378\|FK | Fkbp5 | FKBP5_MOUSI | 1 | 23.5645 | 26.2687 | 24.1443 | 11.1663 | 3.69294 | 11.1632 | 24.6592 | 1.4237 | 8.6741 | 4.3139 | 0.3518 | 3.67E-03 | 9.81E-03 |
| sp\|Q9Z247\|FK | Fkbp9 | FKBP9_MOUSI | 1 | 11.127 | 11.6582 | 13.5616 | 27.148 | 20.3188 | 16.1864 | 12.1156 | 1.2801 | 21.2177 | 5.5358 | 1.7513 | 5.01E-02 | 7.39E-02 |
| sp\|P42128\|FO | Foxk1 | FOXK1_MOUS | 1 | 22.8008 | 25.6452 | 26.9207 | 7.80553 | 5.66783 | 11.1599 | 25.1222 | 2.1092 | 8.2111 | 2.7684 | 0.3268 | 1.09E-03 | 4.88E-03 |
| sp\|P29391\|FR | Ftl1 | FRIL1_MOUSE | 1 | 17.8725 | 20.3943 | 15.1503 | 24.1553 | 15.14 | 7.28767 | 17.8057 | 2.6226 | 15.5277 | 8.4405 | 0.8721 | 6.78E-01 | 7.15E-01 |
| sp\|Q8BGW1\|F | Fto | FTO_MOUSE A | 1 | 17.3697 | 27.5566 | 14.3453 | 17.5255 | 13.6328 | 9.5701 | 19.7572 | 6.9217 | 13.5761 | 3.9780 | 0.6871 | 2.51E-01 | 3.06E-01 |
| tr\|A2AJ72\|A2/ | Fubp3 | A2AJ72_MOUS | 1 | 25.2386 | 24.7166 | 21.8855 | 15.273 | 3.70457 | 9.18173 | 23.9469 | 1.8042 | 9.3864 | 5.7869 | 0.3920 | 1.41E-02 | 2.63E-02 |
| sp\|P70699\|LY/ | Gaa | LYAG_MOUSE | 1 | 23.1509 | 24.4599 | 25.4489 | 10.3101 | 5.07118 | 11.559 | 24.3532 | 1.1527 | 8.9801 | 3.4423 | 0.3687 | 1.84E-03 | 6.20E-03 |
| sp\|Q8R059\|G/ | Gale | GALE_MOUSE | 1 | 32.1121 | 23.9026 | 36.1806 | 2.84733 | 2.0836 | 2.87368 | 30.7318 | 6.2543 | 2.6015 | 0.4487 | 0.0847 | 1.48E-03 | 5.61E-03 |
| sp\|Q9R0N0\|G. | Galk1 | GALK1_MOUSI | 1 | 25.0365 | 26.4853 | 24.9695 | 5.18245 | 9.38764 | 8.93865 | 25.4971 | 0.8565 | 7.8362 | 2.3092 | 0.3073 | 2.42E-04 | 2.29E-03 |
| sp\|Q8R2Q4\|RI | Gfm2 | RRF2M_MOUS | 1 | 31.0195 | 24.2029 | 41.0662 | 0 | 0 | 3.71139 | 32.0962 | 8.4831 | 1.2371 | 2.1428 | 0.0385 | 3.63E-03 | 9.78E-03 |
| sp\|P47856\|GF | Gfpt1 | GFPT1_MOUSI | 1 | 19.1142 | 24.5079 | 24.4987 | 18.3547 | 4.81409 | 8.71049 | 22.7069 | 3.1114 | 10.6264 | 6.9707 | 0.4680 | 5.18E-02 | 7.60E-02 |
| sp\|Q9Z0G0\|GI | Gipc1 | GIPC1_MOUSE | 1 | 24.6318 | 22.7869 | 27.2957 | 7.99912 | 0 | 17.2866 | 24.9048 | 2.2668 | 8.4286 | 8.6513 | 0.3384 | 3.32E-02 | 5.20E-02 |
| sp\|P15105\|GL | Glul | GLNA_MOUSE | 1 | 6.05435 | 11.2429 | 68.9071 | 3.84823 | 4.64309 | 5.3044 | 28.7348 | 34.8868 | 4.5986 | 0.7291 | 0.1600 | 2.97E-01 | 3.56E-01 |
| tr\|A2ADW8\|A | Gm15128 | A2ADW8_MOI | 1 | 32.2244 | 35.8827 | 21.2909 | 0 | 2.56442 | 8.03755 | 29.7993 | 7.5922 | 3.5340 | 4.1056 | 0.1186 | 6.21E-03 | 1.43E-02 |
| sp\|Q8CI11\|GN | Gnl3 | GNL3_MOUSE | 1 | 24.5806 | 22.0564 | 25.1947 | 10.8154 | 6.55832 | 10.7946 | 23.9439 | 1.6632 | 9.3894 | 2.4518 | 0.3921 | 1.05E-03 | 4.76E-03 |
| sp\|Q99JX3\|GC | Gorasp2 | GORS2_MOUS | 1 | 13.8415 | 11.4114 | 9.77112 | 15.5672 | 22.5362 | 26.8726 | 11.6747 | 2.0479 | 21.6587 | 5.7036 | 1.8552 | 4.62E-02 | 6.90E-02 |
| sp\|Q8BUV8\|G | Gpr107 | GP107_MOUSI | 1 | 24.4676 | 18.2149 | 25.722 | 12.1478 | 8.24136 | 11.2063 | 22.8015 | 4.0213 | 10.5318 | 2.0387 | 0.4619 | 9.22E-03 | 1.89E-02 |
| sp\|Q91Z53\|GF | Grhpr | GRHPR_MOUS | 1 | 21.4418 | 19.1297 | 28.5397 | 7.78212 | 7.77634 | 15.3304 | 23.0371 | 4.9036 | 10.2963 | 4.3597 | 0.4469 | 2.82E-02 | 4.55E-02 |
| sp\|Q9WV60\|G | Gsk3b | GSK3B_MOUSI | 1 | 20.6378 | 24.3711 | 41.5335 | 0 | 7.16606 | 6.29156 | 28.8475 | 11.1439 | 4.4859 | 3.9094 | 0.1555 | 2.33E-02 | 3.93E-02 |
| sp\|P10649\|GS | Gstm1 | GSTM1_MOUS | 1 | 18.8002 | 20.3452 | 17.9345 | 22.4878 | 12.3669 | 8.06547 | 19.0266 | 1.2212 | 14.3067 | 7.4043 | 0.7519 | 3.37E-01 | 3.98E-01 |
| tr\|Q80ZM5\|Q | H1fx | Q80ZM5_MOU | 1 | 25.6647 | 28.3817 | 20.5838 | 12.3121 | 7.55578 | 5.50197 | 24.8767 | 3.9582 | 8.4566 | 3.4933 | 0.3399 | 5.74E-03 | 1.36E-02 |
| sp\|Q9QZQ8\|H | H2afy | H2AY_MOUSE | 1 | 5.76518 | 6.55213 | 9.37411 | 26.7613 | 28.5699 | 22.9774 | 7.2305 | 1.8977 | 26.1029 | 2.8538 | 3.6101 | 6.75E-04 | 3.87E-03 |
| sp\|Q61425\|H( | Hadh | HCDH_MOUSE | 1 | 22.4603 | 23.6164 | 22.0577 | 11.5588 | 9.65593 | 10.6509 | 22.7115 | 0.8091 | 10.6219 | 0.9518 | 0.4677 | 7.42E-05 | 1.54E-03 |
| sp\|P35492\|HL | Hal | HUTH_MOUSE | 1 | 15.8962 | 22.4383 | 19.2142 | 8.12951 | 13.7112 | 20.6105 | 19.1829 | 3.2712 | 14.1504 | 6.2521 | 0.7377 | 2.84E-01 | 3.42E-01 |
| sp\|P70288\|HD | Hdac2 | HDAC2_MOUS | 1 | 25.2042 | 33.751 | 22.7914 | 6.99037 | 4.27339 | 6.98966 | 27.2489 | 5.7588 | 6.0845 | 1.5684 | 0.2233 | 3.56E-03 | 9.65E-03 |
| sp\|Q3UGR5\|H | Hdhd2 | HDHD2_MOUS | 1 | 23.8082 | 16.767 | 32.7529 | 12.979 | 3.96064 | 9.73226 | 24.4427 | 8.0118 | 8.8906 | 4.5677 | 0.3637 | 4.32E-02 | 6.49E-02 |
| sp\|Q9JLZ6\|HI | Hic2 | HIC2_MOUSE | 1 | 17.9695 | 22.074 | 22.0471 | 14.5855 | 14.3674 | 8.9565 | 20.6969 | 2.3620 | 12.6365 | 3.1888 | 0.6105 | 2.45E-02 | 4.08E-02 |
| sp\|P70349\|HI | Hint1 | HINT1_MOUSE | 1 | 27.962 | 25.1376 | 26.457 | 8.99645 | 6.23897 | 5.208 | 26.5189 | 1.4132 | 6.8145 | 1.9587 | 0.2570 | 1.46E-04 | 1.84E-03 |
| sp\|Q64524\|H | Hist2h2be | H2B2E_MOUSI | 1 | 7.45771 | 15.7763 | 6.06233 | 38.7065 | 17.9946 | 14.0026 | 9.7654 | 5.2521 | 23.5679 | 13.2615 | 2.4134 | 1.69E-01 | 2.15E-01 |
| sp\|P52927\|HN | Hmga2 | HMGA2_MOU | 1 | 3.87032 | 2.23717 | 3.31637 | 37.4434 | 32.7687 | 20.3641 | 3.1413 | 0.8305 | 30.1921 | 8.8264 | 9.6114 | 6.15E-03 | 1.42E-02 |
| sp\|Q921F4\|HN | Hnrnpll | HNRLL_MOUSI | 1 | 21.6287 | 22.3341 | 25.5278 | 11.3644 | 9.18208 | 9.96279 | 23.1635 | 2.0777 | 10.1698 | 1.1058 | 0.4390 | 6.68E-04 | 3.87E-03 |
| sp\|Q8VDM6\|H | Hnrnpul1 | HNRL1_MOUS | 1 | 9.16657 | 10.3631 | 9.63277 | 19.8998 | 24.4415 | 26.4964 | 9.7208 | 0.6031 | 23.6126 | 3.3755 | 2.4291 | 2.17E-03 | 6.84E-03 |
| sp\|Q00PI9\|HN | Hnrnpul2 | HNRL2_MOUS | 1 | 13.3219 | 17.8855 | 12.7835 | 17.571 | 21.0623 | 17.3758 | 14.6636 | 2.8032 | 18.6697 | 2.0743 | 1.2732 | 1.17E-01 | 1.56E-01 |
| sp\|Q3TEA8\|HI | Hp1bp3 | HP1B3_MOUS | 1 | 12.9257 | 20.331 | 11.3626 | 26.8731 | 16.2609 | 12.2467 | 14.8731 | 4.7909 | 18.4602 | 7.5572 | 1.2412 | 5.26E-01 | 5.71E-01 |
| sp\|P00493\|HP | Hprt1 | HPRT_MOUSE | 1 | 22.7343 | 21.1787 | 23.7922 | 13.4304 | 9.2813 | 9.58312 | 22.5684 | 1.3146 | 10.7649 | 2.3133 | 0.4770 | 1.54E-03 | 5.79E-03 |
| sp\|Q99M31\|H | Hspa14 | HSP7E_MOUSI | 1 | 20.7482 | 20.7158 | 20.1118 | 14.8955 | 14.0108 | 9.51784 | 20.5253 | 0.3584 | 12.8080 | 2.8835 | 0.6240 | 1.00E-02 | 2.02E-02 |
| sp\|Q9JIY5\|HTI | Htra2 | HTRA2_MOUS | 1 | 26.6931 | 25.5351 | 27.9278 | 6.87913 | 3.23409 | 9.73073 | 26.7187 | 1.1966 | 6.6147 | 3.2564 | 0.2476 | 5.54E-04 | 3.50E-03 |
| sp\|Q9CPN8\|IF | Igf2bp3 | IF2B3_MOUSE | 1 | 2.40382 | 0 | 2.39464 | 37.3956 | 35.6602 | 22.1458 | 1.5995 | 1.3852 | 31.7339 | 8.3487 | 19.8400 | 3.51E-03 | 9.56E-03 |
| sp\|O55222\|ILI | Ilk | ILK_MOUSE In | 1 | 12.5502 | 18.1646 | 8.87708 | 24.6305 | 19.6023 | 16.1753 | 13.1973 | 4.6775 | 20.1360 | 4.2528 | 1.5258 | 1.30E-01 | 1.71E-01 |
| sp\|Q8CDA1\|S/ | Inpp5f | SAC2_MOUSE | 1 | 25.7477 | 31.147 | 32.1945 | 0 | 7.84648 | 3.06434 | 29.6964 | 3.4596 | 3.6369 | 3.9545 | 0.1225 | 1.01E-03 | 4.73E-03 |
| sp\|Q9CX00\|IS | Ist1 | IST1_MOUSE I | 1 | 18.742 | 24.7044 | 26.7049 | 5.11155 | 8.97656 | 15.7606 | 23.3838 | 4.1425 | 9.9496 | 5.3908 | 0.4255 | 2.67E-02 | 4.35E-02 |
| sp\|Q8K2Q9\|SI | Kiaa1598 | SHOT1_MOUS | 1 | 1.10039 | 5.81421 | 0.0653631 | 0 | 0 | 93.02 | 2.3267 | 3.0643 | 31.0067 | 53.7051 | 13.3267 | 4.08E-01 | 4.63E-01 |
| sp\|O35071\|KI | Kif1c | KIF1C_MOUSE | 1 | 13.3827 | 15.9145 | 13.994 | 21.4546 | 21.9615 | 13.2926 | 14.4304 | 1.3211 | 18.9029 | 4.8653 | 1.3099 | 1.99E-01 | 2.48E-01 |
| sp\|P28740\|KII | Kif2a | KIF2A_MOUSE | 1 | 25.4412 | 25.7328 | 21.2217 | 5.4467 | 12.8682 | 9.2895 | 24.1319 | 2.5245 | 9.2015 | 3.7115 | 0.3813 | 4.50E-03 | 1.14E-02 |
| sp\|O35344\|IV | Kpna3 | IMA4_MOUSE | 1 | 28.9426 | 21.0079 | 26.6 | 7.29556 | 9.13565 | 7.01827 | 25.5168 | 4.0767 | 7.8165 | 1.1508 | 0.3063 | 1.93E-03 | 6.45E-03 |
| sp\|P08730\|K1 | Krt13 | K1C13_MOUSI | 1 | 4.77396 | 2.74231 | 5.27946 | 9.9001 | 18.3133 | 58.9909 | 4.2652 | 1.3429 | 29.0681 | 26.2531 | 6.8151 | 1.78E-01 | 2.24E-01 |
| sp\|Q8K0Y2\|KT | Krt33a | KT33A_MOUSI | 1 | 1.02904 | 2.7632 | 0.257509 | 0 | 0 | 95.9502 | 1.3499 | 1.2833 | 31.9834 | 55.3969 | 23.6929 | 3.93E-01 | 4.49E-01 |
| tr\|F8VQJ3\|F8 | Lamc1 | F8VQJ3_MOU | 1 | 23.4978 | 53.2322 | 15.1057 | 1.66203 | 3.5493 | 2.95296 | 30.6119 | 20.0341 | 2.7214 | 0.9647 | 0.0889 | 7.37E-02 | 1.04E-01 |
| sp\|P11438\|LA | Lamp1 | LAMP1_MOUS | 1 | 18.4952 | 14.9535 | 28.5211 | 16.0211 | 9.39476 | 12.6143 | 20.6566 | 7.0373 | 12.6767 | 3.3136 | 0.6137 | 1.50E-01 | 1.94E-01 |
| sp\|P17047\|LA | Lamp2 | LAMP2_MOUS | 1 | 22.0722 | 23.4664 | 29.1059 | 8.6843 | 10.685 | 5.9863 | 24.8815 | 3.7243 | 8.4519 | 2.3580 | 0.3397 | 2.96E-03 | 8.47E-03 |
| sp\|Q05CL8\|LA | Larp7 | LARP7_MOUSI | 1 | 25.2076 | 31.9822 | 24.7428 | 7.25238 | 0 | 10.815 | 27.3109 | 4.0522 | 6.0225 | 5.5114 | 0.2205 | 5.73E-03 | 1.36E-02 |
| sp\|P16110\|LE | Lgals3 | LEG3_MOUSE | 1 | 4.47796 | 7.64678 | 4.97947 | 38.6702 | 23.9774 | 20.2482 | 5.7014 | 1.7033 | 27.6319 | 9.7396 | 4.8465 | 1.84E-02 | 3.26E-02 |
| sp\|P37913\|DN | Lig1 | DNLI1_MOUSE | 1 | 27.3395 | 26.0478 | 29.5944 | 7.27711 | 4.95053 | 4.79069 | 27.6606 | 1.7950 | 5.6728 | 1.3917 | 0.2051 | 7.41E-05 | 1.54E-03 |

| | | | | | | | | | | | | | | | | |
|---|---|---|---|---|---|---|---|---|---|---|---|---|---|---|---|---|
| sp\|Q9DBH5\|LI | Lman2 | LMAN2_MOUS | 1 | 22.7296 | 19.3852 | 25.5717 | 13.3218 | 9.74371 | 9.24802 | 22.5622 | 3.0966 | 10.7712 | 2.2228 | 0.4774 | 5.86E-03 | 1.38E-02 |
| sp\|Q80X72\|LR | Lrrc15 | LRC15_MOUSE | 1 | 3.30218 | 1.90353 | 2.00952 | 0 | 0 | 92.7848 | 2.4051 | 0.7787 | 30.9283 | 53.5693 | 12.8596 | 4.09E-01 | 4.63E-01 |
| tr\|G5E8E1\|G5 | Lrrfip1 | G5E8E1_MOU | 1 | 7.38282 | 3.26405 | 9.37643 | 25.3877 | 28.3821 | 26.2069 | 6.6744 | 3.1172 | 26.6589 | 1.5475 | 3.9942 | 5.74E-04 | 3.52E-03 |
| sp\|P08905\|LY | Lyz2 | LYZ2_MOUSE I | 1 | 7.26677 | 14.0209 | 5.88799 | 35.8054 | 21.3732 | 15.6457 | 9.0586 | 4.3525 | 24.2748 | 10.3883 | 2.6798 | 7.94E-02 | 1.10E-01 |
| sp\|Q9CQY5\|M | Magt1 | MAGT1_MOUS | 1 | 17.7259 | 20.5892 | 18.3875 | 11.5179 | 19.1519 | 12.6275 | 18.9009 | 1.4991 | 14.4324 | 4.1247 | 0.7636 | 1.53E-01 | 1.97E-01 |
| sp\|P27046\|MA | Man2a1 | MA2A1_MOUS | 1 | 8.58642 | 5.0064 | 10.4458 | 32.0098 | 25.4934 | 18.4582 | 8.0129 | 2.7647 | 25.3205 | 6.7775 | 3.1600 | 1.49E-02 | 2.74E-02 |
| sp\|Q9ESN9\|JIF | Mapk8ip3 | JIP3_MOUSE C | 1 | 28.876 | 29.2961 | 26.8107 | 5.85978 | 3.90465 | 5.25278 | 28.3276 | 1.3304 | 5.0057 | 1.0007 | 0.1767 | 1.71E-05 | 1.03E-03 |
| sp\|Q61166\|M | Mapre1 | MARE1_MOUS | 1 | 16.3617 | 20.1425 | 35.4996 | 7.86319 | 7.59319 | 12.5398 | 24.0013 | 10.1357 | 9.3321 | 2.7813 | 0.3888 | 7.30E-02 | 1.03E-01 |
| sp\|P26645\|MA | Marcks | MARCS_MOUS | 1 | 22.9694 | 15.2833 | 15.5543 | 20.9225 | 13.6714 | 11.5991 | 17.9357 | 4.3614 | 15.3977 | 4.8956 | 0.8585 | 5.39E-01 | 5.83E-01 |
| sp\|Q99LB6\|MA | Mat2b | MAT2B_MOUS | 1 | 26.1007 | 22.3402 | 36.1458 | 0 | 8.15574 | 7.2576 | 28.1956 | 7.1372 | 5.1378 | 4.4721 | 0.1822 | 9.03E-03 | 1.87E-02 |
| sp\|Q8C181\|M | Mbnl2 | MBNL2_MOUS | 1 | 18.6881 | 24.602 | 16.4808 | 14.7578 | 15.9386 | 9.53264 | 19.9236 | 4.1992 | 13.4097 | 3.4091 | 0.6731 | 1.05E-01 | 1.42E-01 |
| sp\|Q80YQ2\|M | Med23 | MED23_MOUS | 1 | 10.5108 | 10.905 | 5.485 | 34.6245 | 22.0065 | 16.4682 | 8.9669 | 3.0219 | 24.3664 | 9.3054 | 2.7174 | 5.26E-02 | 7.71E-02 |
| sp\|O08663\|M | Metap2 | MAP2_MOUSE | 1 | 28.7175 | 14.0557 | 34.0543 | 0 | 13.6018 | 9.5706 | 25.6092 | 10.3553 | 7.7241 | 6.9864 | 0.3016 | 6.82E-02 | 9.70E-02 |
| sp\|P34884\|MI | Mif | MIF_MOUSE M | 1 | 18.2379 | 15.3772 | 14.3889 | 28.1732 | 12.5052 | 11.3175 | 16.0013 | 1.9990 | 17.3320 | 9.4075 | 1.0832 | 8.22E-01 | 8.47E-01 |
| sp\|Q99KX1\|M | Mlf2 | MLF2_MOUSE | 1 | 27.023 | 28.0618 | 21.3315 | 0 | 6.26802 | 17.3157 | 25.4721 | 3.6233 | 7.8612 | 8.7671 | 0.3086 | 3.24E-02 | 5.11E-02 |
| sp\|P23249\|MO | Mov10 | MOV10_MOUS | 1 | 12.7491 | 14.6491 | 11.6076 | 23.5589 | 21.5794 | 15.856 | 13.0019 | 1.5364 | 20.3314 | 4.0002 | 1.5637 | 4.14E-02 | 6.26E-02 |
| sp\|P97434\|MI | Mprip | MPRIP_MOUS | 1 | 9.6148 | 2.45337 | 11.9784 | 10.3498 | 38.4023 | 27.2013 | 8.0155 | 4.9598 | 25.3178 | 14.1208 | 3.1586 | 1.16E-01 | 1.55E-01 |
| sp\|Q91ZC4\|M | Mrgpra8 | MRGA8_MOU | 1 | 6.73688 | 6.27497 | 11.7675 | 16.9615 | 15.7189 | 42.5402 | 8.2598 | 3.0465 | 25.0735 | 15.1393 | 3.0356 | 1.32E-01 | 1.74E-01 |
| sp\|Q9JIK9\|RT3 | Mrps34 | RT34_MOUSE | 1 | 22.1364 | 27.7955 | 14.8952 | 9.6878 | 12.6514 | 12.8337 | 21.6090 | 6.4663 | 11.7243 | 1.7660 | 0.5426 | 6.30E-02 | 9.03E-02 |
| sp\|P43247\|MS | Msh2 | MSH2_MOUSE | 1 | 19.2513 | 16.5371 | 38.9743 | 5.04152 | 15.172 | 5.0237 | 24.9209 | 12.2460 | 8.4124 | 5.8540 | 0.3376 | 1.03E-01 | 1.39E-01 |
| sp\|Q8K4B0\|M | Mta1 | MTA1_MOUSE | 1 | 24.3069 | 29.0793 | 23.3616 | 9.6589 | 8.62895 | 4.96439 | 25.5826 | 3.0649 | 7.7507 | 2.4674 | 0.3030 | 1.42E-03 | 5.48E-03 |
| sp\|Q791V5\|M | Mtch2 | MTCH2_MOUS | 1 | 21.655 | 21.4764 | 25.0527 | 15.9249 | 7.40338 | 8.48772 | 22.7280 | 2.0152 | 10.6053 | 4.6387 | 0.4666 | 1.42E-02 | 2.64E-02 |
| sp\|P00405\|CC | Mtco2 | COX2_MOUSE | 1 | 20.1627 | 21.7528 | 24.5913 | 8.68756 | 14.8916 | 9.91399 | 22.1689 | 2.2434 | 11.1644 | 3.2856 | 0.5036 | 8.71E-03 | 1.83E-02 |
| sp\|Q80WJ7\|LY | Mtdh | LYRIC_MOUSE | 1 | 28.3827 | 22.4817 | 16.5063 | 14.1346 | 9.63628 | 8.854 | 22.4584 | 5.9405 | 10.8750 | 2.8499 | 0.4842 | 3.82E-02 | 5.85E-02 |
| sp\|P03911\|NU | Mtnd4 | NU4M_MOUSI | 1 | 17.7089 | 15.6037 | 17.7868 | 18.8147 | 17.6661 | 12.4198 | 17.0331 | 1.2385 | 16.3002 | 3.4092 | 0.9570 | 7.44E-01 | 7.74E-01 |
| sp\|P62774\|MT | Mtpn | MTPN_MOUSE | 1 | 23.1099 | 27.5938 | 24.8454 | 7.7629 | 7.11322 | 9.57477 | 25.1830 | 2.2609 | 8.1503 | 1.2757 | 0.3236 | 3.42E-04 | 2.69E-03 |
| sp\|Q8C854\|M | Myef2 | MYEF2_MOUS | 1 | 16.6158 | 21.1728 | 14.3047 | 18.5083 | 17.7749 | 11.6234 | 17.3644 | 3.4947 | 15.9689 | 3.7811 | 0.9196 | 6.63E-01 | 7.02E-01 |
| sp\|Q8BWZ3\|N | Naa25 | NAA25_MOUS | 1 | 21.6372 | 20.6315 | 23.5153 | 13.03 | 7.95005 | 13.236 | 21.9280 | 1.4637 | 11.4054 | 2.9941 | 0.5201 | 5.44E-03 | 1.31E-02 |
| sp\|Q8VBW6\|U | Nae1 | ULA1_MOUSE | 1 | 29.4542 | 19.0198 | 30.1711 | 6.64297 | 7.78533 | 6.92668 | 26.2150 | 6.2416 | 7.1183 | 0.5948 | 0.2715 | 6.19E-03 | 1.43E-02 |
| tr\|O88325\|O8 | Naglu | O88325_MOU | 1 | 22.4893 | 22.0716 | 22.8985 | 10.4753 | 7.17841 | 14.8868 | 22.4865 | 0.4135 | 10.8468 | 3.8676 | 0.4824 | 6.59E-03 | 1.50E-02 |
| sp\|Q99KQ4\|N | Nampt | NAMPT_MOU | 1 | 27.4321 | 20.738 | 31.5908 | 10.2758 | 3.0988 | 6.86453 | 26.5870 | 5.4755 | 6.7464 | 3.5900 | 0.2537 | 6.30E-03 | 1.44E-02 |
| sp\|P28660\|NC | Nckap1 | NCKP1_MOUS | 1 | 25.5469 | 18.9838 | 31.9124 | 4.09299 | 10.422 | 9.04191 | 25.4810 | 6.4646 | 7.8523 | 3.3280 | 0.3082 | 1.37E-02 | 2.56E-02 |
| sp\|Q9CQZ5\|N | Ndufa6 | NDUA6_MOUS | 1 | 23.1777 | 22.576 | 21.5149 | 14.2391 | 9.89827 | 8.59411 | 22.4229 | 0.8419 | 10.9105 | 2.9555 | 0.4866 | 2.91E-03 | 8.34E-03 |
| sp\|P19246\|NF | Nefh | NFH_MOUSE N | 1 | 10.0072 | 5.70015 | 6.64308 | 24.5035 | 37.5637 | 15.5824 | 7.4501 | 2.2641 | 25.8832 | 11.0554 | 3.4742 | 4.74E-02 | 7.06E-02 |
| sp\|Q91YP2\|NE | Nln | NEUL_MOUSE | 1 | 19.9414 | 29.4431 | 29.6824 | 7.06295 | 4.19118 | 9.67896 | 26.3556 | 5.5562 | 6.9777 | 2.7449 | 0.2648 | 5.63E-03 | 1.35E-02 |
| sp\|Q3TKR3\|NA | Nlrp4c | NAL4C_MOUS | 1 | 29.4155 | 33.9178 | 28.1858 | 0 | 2.25202 | 6.22886 | 30.5064 | 3.0177 | 2.8270 | 3.1540 | 0.0927 | 3.91E-04 | 2.88E-03 |
| sp\|Q922K7\|NO | Nop2 | NOP2_MOUSE | 1 | 15.2292 | 17.3078 | 29.0589 | 8.4584 | 17.8208 | 12.1249 | 20.5320 | 7.4573 | 12.8014 | 4.7177 | 0.6235 | 2.04E-01 | 2.53E-01 |
| sp\|P97300\|NP | Nptn | NPTN_MOUSE | 1 | 24.7543 | 25.0961 | 25.0098 | 6.52286 | 6.29633 | 12.3206 | 24.9534 | 0.1777 | 8.3799 | 3.4146 | 0.3358 | 1.10E-03 | 4.89E-03 |
| sp\|Q9CZ44\|NS | Nsfl1c | NSF1C_MOUSI | 1 | 34.6555 | 19.4588 | 27.4051 | 6.48049 | 4.94669 | 7.05349 | 27.1731 | 7.6010 | 6.1602 | 1.0893 | 0.2267 | 9.04E-03 | 1.87E-02 |
| sp\|Q80XU3\|N | Nucks1 | NUCKS_MOUS | 1 | 31.2983 | 11.8828 | 20.1404 | 17.3723 | 11.5597 | 7.74665 | 21.1072 | 9.7438 | 12.2262 | 4.8473 | 0.5792 | 2.30E-01 | 2.84E-01 |
| sp\|Q6PIP5\|NU | Nudcd1 | NUDC1_MOUS | 1 | 28.322 | 23.3792 | 29.4357 | 7.33153 | 5.3093 | 6.22227 | 27.0456 | 3.2237 | 6.2877 | 1.0127 | 0.2325 | 4.42E-04 | 3.07E-03 |
| sp\|Q9JKX6\|NU | Nudt5 | NUDT5_MOUS | 1 | 35.0169 | 31.6707 | 28.3743 | 0 | 0 | 4.9381 | 31.6873 | 3.3213 | 1.6460 | 2.8510 | 0.0519 | 2.87E-04 | 2.45E-03 |
| sp\|Q5F2E7\|NU | Nufip2 | NUFP2_MOUS | 1 | 13.8332 | 15.2312 | 13.3854 | 22.5688 | 18.8626 | 16.1188 | 14.1499 | 0.9628 | 19.1834 | 3.2369 | 1.3557 | 6.12E-02 | 8.79E-02 |
| sp\|Q8R0G9\|N | Nup133 | NU133_MOUS | 1 | 23.279 | 29.0814 | 23.6309 | 9.11442 | 5.89164 | 9.00254 | 25.3304 | 3.2532 | 8.0029 | 1.8292 | 0.3159 | 1.30E-03 | 5.31E-03 |
| sp\|Q99P88\|NU | Nup155 | NU155_MOUS | 1 | 21.0334 | 21.6071 | 23.2174 | 12.1771 | 5.42085 | 16.5442 | 21.9526 | 1.1323 | 11.3807 | 5.6043 | 0.5184 | 3.28E-02 | 5.14E-02 |
| tr\|Q4ZGD9\|Q4 | Nxf3 | Q4ZGD9_MOU | 1 | 25.8416 | 26.8957 | 22.9751 | 8.20926 | 7.06581 | 9.01261 | 25.2375 | 2.0289 | 8.0959 | 0.9783 | 0.3208 | 1.91E-04 | 2.08E-03 |
| tr\|D3YYU8\|D3 | Obsl1 | D3YYU8_MOU | 1 | 21.582 | 32.9383 | 20.1995 | 9.90311 | 8.3042 | 7.07286 | 24.9066 | 6.9899 | 8.4267 | 1.4191 | 0.3383 | 1.61E-02 | 2.90E-02 |
| tr\|E9Q7L0\|E9 | Ogdhl | E9Q7L0_MOU | 1 | 29.8948 | 41.0996 | 26.2327 | 0 | 0 | 2.77301 | 32.4090 | 7.7458 | 0.9243 | 1.6010 | 0.0285 | 2.32E-03 | 7.14E-03 |
| sp\|P58281\|OP | Opa1 | OPA1_MOUSE | 1 | 17.6758 | 29.0508 | 26.059 | 5.06029 | 10.4187 | 11.7353 | 24.2619 | 5.8966 | 9.0714 | 3.5356 | 0.3739 | 1.87E-02 | 3.30E-02 |
| sp\|Q6IE21\|OT | Otud6a | OTU6A_MOUS | 1 | 10.6626 | 11.011 | 9.5466 | 22.3011 | 23.6982 | 22.7805 | 10.4067 | 0.7650 | 22.9266 | 0.7099 | 2.2031 | 3.17E-05 | 1.17E-03 |
| tr\|A3KFU5\|A3 | Pabpc4 | A3KFU5_MOU | 1 | 20.6575 | 20.0135 | 19.1138 | 13.9111 | 17.2121 | 9.09204 | 19.9283 | 0.7754 | 13.4051 | 4.0836 | 0.6727 | 5.31E-02 | 7.75E-02 |
| sp\|Q8CCS6\|PA | Pabpn1 | PABP2_MOUSI | 1 | 18.3135 | 21.9693 | 16.1084 | 17.0517 | 16.5424 | 10.0148 | 18.7971 | 2.9602 | 14.5363 | 3.9240 | 0.7733 | 2.08E-01 | 2.58E-01 |
| sp\|Q8K212\|PA | Pacs1 | PACS1_MOUSI | 1 | 25.8534 | 23.758 | 29.8301 | 6.97835 | 4.8565 | 8.72361 | 26.4805 | 3.0842 | 6.8528 | 1.9366 | 0.2588 | 7.33E-04 | 4.06E-03 |
| sp\|Q61205\|PA | Pafah1b3 | PA1B3_MOUSI | 1 | 31.6592 | 24.9721 | 32.8319 | 0 | 5.9159 | 4.62096 | 29.8211 | 4.2401 | 3.5123 | 3.1099 | 0.1178 | 9.76E-04 | 4.64E-03 |
| sp\|Q9DCL9\|PU | Paics | PUR6_MOUSE | 1 | 24.3878 | 27.7836 | 23.5742 | 10.0395 | 6.11899 | 8.09592 | 25.2485 | 2.2328 | 8.0848 | 1.9603 | 0.3202 | 5.61E-04 | 3.52E-03 |
| sp\|Q05920\|PY | Pc | PYC_MOUSE P | 1 | 17.5984 | 20.3516 | 23.1845 | 6.92051 | 19.1818 | 12.7631 | 20.3782 | 2.7931 | 12.9551 | 6.1329 | 0.6357 | 1.29E-01 | 1.70E-01 |
| sp\|Q61990\|PC | Pcbp2 | PCBP2_MOUSI | 1 | 21.5538 | 24.4177 | 24.4075 | 7.6355 | 11.4037 | 10.5818 | 23.4597 | 1.6505 | 9.8737 | 1.9814 | 0.4209 | 8.00E-04 | 4.27E-03 |
| sp\|Q11136\|PE | Pepd | PEPD_MOUSE | 1 | 25.8176 | 24.9211 | 25.6944 | 6.0203 | 5.59582 | 11.9508 | 25.4777 | 0.4859 | 7.8556 | 3.5529 | 0.3083 | 1.05E-03 | 4.76E-03 |
| sp\|Q9EQ61\|PE | Pes1 | PESC_MOUSE | 1 | 19.83 | 24.843 | 23.7643 | 7.67808 | 12.9025 | 10.9821 | 22.8124 | 2.6386 | 10.5209 | 2.6426 | 0.4612 | 4.68E-03 | 1.17E-02 |
| sp\|Q9R0A0\|PE | Pex14 | PEX14_MOUSI | 1 | 32.9016 | 24.1027 | 25.1294 | 6.84315 | 4.86629 | 6.15686 | 27.3779 | 4.8111 | 5.9554 | 1.0037 | 0.2175 | 1.65E-03 | 5.94E-03 |
| sp\|Q9JJV2\|PR | Pfn2 | PROF2_MOUS | 1 | 25.6578 | 25.539 | 30.9093 | 7.30967 | 6.85525 | 3.72902 | 27.3687 | 3.0668 | 5.9646 | 1.9494 | 0.2179 | 5.20E-04 | 3.39E-03 |
| sp\|Q9CQ60\|6I | Pgls | 6PGL_MOUSE | 1 | 21.7775 | 19.8408 | 24.2624 | 16.6017 | 10.1786 | 7.33903 | 21.9602 | 2.2165 | 11.3731 | 4.7455 | 0.5179 | 2.49E-02 | 4.14E-02 |
| sp\|Q7M6Y3\|P | Picalm | PICAL_MOUSE | 1 | 16.5592 | 15.6334 | 20.6042 | 20.7155 | 13.2815 | 13.2063 | 17.5989 | 2.6435 | 15.7344 | 4.3139 | 0.8941 | 5.58E-01 | 6.00E-01 |

| | | | | | | | | | | | | | | | | | |
|---|---|---|---|---|---|---|---|---|---|---|---|---|---|---|---|---|---|
| sp\|Q6PD26\|PI | Pigs | PIGS_MOUSE ( | 1 | 26.0563 | 26.0417 | 26.0856 | 0 | 9.32636 | 12.4901 | 26.0612 | 0.0224 | 7.2722 | 6.4935 | 0.2790 | 7.43E-03 | 1.63E-02 |
| sp\|Q4KWH5\|F | Plch1 | PLCH1_MOUSI | 1 | 24.9162 | 21.3877 | 25.4146 | 10.7702 | 9.3681 | 8.14328 | 23.9062 | 2.1952 | 9.4272 | 1.3145 | 0.3943 | 6.07E-04 | 3.63E-02 |
| sp\|Q8BLJ3\|PL( | Plcxd3 | PLCX3_MOUSI | 1 | 27.4123 | 19.9554 | 50.1228 | 0 | 0 | 2.50951 | 32.4968 | 15.7133 | 0.8365 | 1.4489 | 0.0257 | 2.55E-02 | 4.22E-02 |
| sp\|Q922V4\|PL | Plrg1 | PLRG1_MOUSI | 1 | 29.7413 | 40.7221 | 21.0752 | 0 | 8.4615 | 0 | 30.5129 | 9.8461 | 2.8205 | 4.8852 | 0.0924 | 1.20E-02 | 2.30E-02 |
| sp\|Q9DC61\|M | Pmpca | MPPA_MOUSE | 1 | 20.3609 | 18.9805 | 25.0251 | 8.4427 | 0 | 27.1908 | 21.4555 | 3.1675 | 11.8778 | 13.9171 | 0.5536 | 3.10E-01 | 3.70E-01 |
| sp\|Q9D1G2\|PI | Pmvk | PMVK_MOUSE | 1 | 27.3195 | 29.525 | 33.2223 | 0 | 3.68257 | 6.25062 | 30.0223 | 2.9827 | 3.3111 | 3.1418 | 0.1103 | 4.35E-04 | 3.05E-03 |
| sp\|P23492\|PN | Pnp | PNPH_MOUSE | 1 | 14.7404 | 10.7999 | 28.9062 | 5.99018 | 6.10599 | 33.4574 | 18.1488 | 9.5222 | 15.1845 | 15.8249 | 0.8367 | 7.95E-01 | 8.23E-01 |
| sp\|O35134\|RF | Polr1a | RPA1_MOUSE | 1 | 19.8727 | 28.1135 | 23.4161 | 9.91449 | 8.25075 | 10.4326 | 23.8008 | 4.1338 | 9.5326 | 1.1400 | 0.4005 | 4.50E-03 | 1.14E-02 |
| sp\|Q8CFI7\|RPI | Polr2b | RPB2_MOUSE | 1 | 22.1775 | 32.5465 | 22.6707 | 12.7272 | 4.60054 | 5.27748 | 25.7982 | 5.8494 | 7.5351 | 4.5092 | 0.2921 | 1.28E-02 | 2.43E-02 |
| tr\|Q8K205\|Q8 | Pop1 | Q8K205_MOU | 1 | 19.169 | 19.9555 | 13.7758 | 18.2209 | 17.2056 | 11.6731 | 17.6334 | 3.3639 | 15.6999 | 3.5240 | 0.8903 | 5.30E-01 | 5.75E-01 |
| tr\|Q8CIH9\|Q8 | Ppat | Q8CIH9_MOU! | 1 | 25.8835 | 27.1807 | 31.8855 | 0 | 7.34366 | 7.70666 | 28.3166 | 3.1581 | 5.0168 | 4.3484 | 0.1772 | 1.68E-03 | 5.94E-03 |
| sp\|Q9DBR7\|M | Ppp1r12a | MYPT1_MOUS | 1 | 20.5245 | 11.1326 | 21.6325 | 19.5766 | 16.0974 | 11.0363 | 17.7632 | 5.7689 | 15.5701 | 4.2945 | 0.8765 | 6.25E-01 | 6.65E-01 |
| sp\|Q6PD03\|2/ | Ppp2r5a | 2A5A_MOUSE | 1 | 25.6397 | 30.1691 | 33.6508 | 0 | 2.79279 | 7.74757 | 29.8199 | 4.0170 | 3.5135 | 3.9237 | 0.1178 | 1.25E-03 | 5.21E-03 |
| sp\|Q5EG47\|A/ | Prkaa1 | AAPK1_MOUS | 1 | 13.3717 | 16.5805 | 5.85112 | 37.2729 | 14.1992 | 12.7247 | 11.9344 | 5.5072 | 21.3989 | 13.7670 | 1.7930 | 3.31E-01 | 3.93E-01 |
| sp\|O54950\|A/ | Prkag1 | AAKG1_MOUS | 1 | 8.622 | 15.2677 | 13.8678 | 9.63216 | 16.1815 | 36.4288 | 12.5858 | 3.5034 | 20.7475 | 13.9697 | 1.6485 | 3.82E-01 | 4.41E-01 |
| sp\|Q922H1\|AI | Prmt3 | ANM3_MOUSI | 1 | 22.0322 | 22.3386 | 36.1522 | 8.17774 | 6.7121 | 4.58708 | 26.8410 | 8.0652 | 6.4923 | 1.8054 | 0.2419 | 1.30E-02 | 2.46E-02 |
| sp\|Q922U1\|PI | Prpf3 | PRPF3_MOUSI | 1 | 17.3695 | 18.0439 | 18.412 | 11.1234 | 13.5602 | 21.4909 | 17.9418 | 0.5287 | 15.3915 | 5.4209 | 0.8579 | 4.63E-01 | 5.14E-01 |
| sp\|Q3UPH1\|P | Prrc1 | PRRC1_MOUSI | 1 | 19.317 | 24.0535 | 37.1023 | 8.97245 | 5.55348 | 5.00137 | 26.8243 | 9.2107 | 6.5091 | 2.1511 | 0.2427 | 2.05E-02 | 3.54E-02 |
| sp\|Q7TSC1\|PR | Prrc2a | PRC2A_MOUS | 1 | 18.7295 | 21.511 | 16.2698 | 15.8275 | 17.3236 | 10.3386 | 18.8368 | 2.6222 | 14.4966 | 3.6778 | 0.7696 | 1.71E-01 | 2.17E-01 |
| sp\|Q9Z2X2\|PS | Psmd10 | PSD10_MOUSI | 1 | 18.6264 | 19.1366 | 19.0477 | 16.2108 | 14.9905 | 11.988 | 18.9369 | 0.2725 | 14.3964 | 2.1732 | 0.7602 | 2.29E-02 | 3.89E-02 |
| sp\|O35593\|PS | Psmd14 | PSDE_MOUSE | 1 | 17.0665 | 20.4288 | 14.5992 | 21.7991 | 15.3419 | 10.7646 | 17.3648 | 2.9262 | 15.9685 | 5.5439 | 0.9196 | 7.19E-01 | 7.52E-01 |
| sp\|P26516\|PS | Psmd7 | PSMD7_MOUS | 1 | 22.6486 | 23.3625 | 32.4135 | 5.58108 | 7.33726 | 8.65706 | 26.1415 | 5.4434 | 7.1918 | 1.5431 | 0.2751 | 4.39E-03 | 1.12E-02 |
| sp\|P61290\|PS | Psme3 | PSME3_MOUS | 1 | 26.2936 | 21.2628 | 31.4295 | 5.90798 | 8.03648 | 7.06956 | 26.3286 | 5.0834 | 7.0047 | 1.0657 | 0.2660 | 2.98E-03 | 8.51E-03 |
| sp\|Q9JK23\|PS | Psmg1 | PSMG1_MOUS | 1 | 22.1206 | 21.6965 | 28.6893 | 9.09979 | 4.48635 | 13.9075 | 24.1688 | 3.9206 | 9.1645 | 4.7109 | 0.3792 | 1.33E-02 | 2.50E-02 |
| sp\|Q14C51\|PT | Ptcd3 | PTCD3_MOUS | 1 | 9.66476 | 24.7071 | 24.9977 | 12.5815 | 8.93633 | 19.1126 | 19.7899 | 8.7698 | 13.5435 | 5.1559 | 0.6844 | 3.47E-01 | 4.07E-01 |
| sp\|Q9R0Q7\|TI | Ptges3 | TEBP_MOUSE | 1 | 26.3203 | 19.7169 | 22.324 | 16.005 | 8.21519 | 7.41849 | 22.7871 | 3.3260 | 10.5462 | 4.7442 | 0.4628 | 2.16E-02 | 3.69E-02 |
| sp\|Q91VU8\|P | Ptov1 | PTOV1_MOUS | 1 | 22.1563 | 45.9604 | 17.9396 | 8.62117 | 0 | 5.32255 | 28.6854 | 15.1084 | 4.6479 | 4.3500 | 0.1620 | 5.71E-02 | 8.25E-02 |
| sp\|Q6PB44\|PT | Ptpn23 | PTN23_MOUSI | 1 | 15.8919 | 23.1965 | 20.7032 | 17.5883 | 11.8714 | 10.7487 | 19.9305 | 3.7131 | 13.4028 | 3.6680 | 0.6725 | 9.62E-02 | 1.31E-01 |
| sp\|Q80U58\|PI | Pum2 | PUM2_MOUSE | 1 | 24.1592 | 26.7499 | 23.2554 | 7.77203 | 7.20626 | 10.8573 | 24.7215 | 1.8138 | 8.6119 | 1.9651 | 0.3484 | 4.77E-04 | 3.22E-03 |
| sp\|P42669\|PU | Pura | PURA_MOUSE | 1 | 12.4242 | 13.2418 | 10.7418 | 22.0124 | 23.7796 | 17.8003 | 12.1359 | 1.2747 | 21.1974 | 3.0718 | 1.7467 | 9.18E-03 | 1.88E-02 |
| tr\|F7AC41\|F7/ | Pus7 | F7AC41_MOU! | 1 | 26.6009 | 28.555 | 29.1899 | 0 | 4.7063 | 10.948 | 28.1153 | 1.3494 | 5.2181 | 5.4919 | 0.1856 | 2.18E-03 | 6.84E-03 |
| sp\|Q9DCC4\|P! | Pycrl | P5CR3_MOUSI | 1 | 26.2835 | 28.104 | 39.7104 | 0 | 0 | 5.90211 | 31.3660 | 7.2836 | 1.9674 | 3.4076 | 0.0627 | 3.18E-03 | 8.94E-03 |
| tr\|D3Z158\|D3 | Qars | D3Z158_MOU! | 1 | 22.3506 | 25.3974 | 24.7887 | 5.85696 | 7.82904 | 13.7772 | 24.1789 | 1.6123 | 9.1544 | 4.1231 | 0.3786 | 4.19E-03 | 1.08E-02 |
| sp\|P53994\|RA | Rab2a | RAB2A_MOUS | 1 | 20.8106 | 19.5413 | 19.4412 | 17.1125 | 10.1557 | 12.9387 | 19.9310 | 0.7634 | 13.4023 | 3.5015 | 0.6724 | 3.43E-02 | 5.34E-02 |
| sp\|Q9CQD1\|R | Rab5a | RAB5A_MOUS | 1 | 20.08 | 18.9503 | 23.1092 | 14.7915 | 11.3386 | 11.7305 | 20.7132 | 2.1505 | 12.6202 | 1.8906 | 0.6093 | 8.07E-03 | 1.74E-02 |
| sp\|Q9R0M6\|R | Rab9a | RAB9A_MOUS | 1 | 28.0259 | 26.4829 | 26.3078 | 9.79083 | 2.71954 | 6.67302 | 26.9389 | 0.9455 | 6.3945 | 3.5439 | 0.2374 | 6.32E-04 | 3.71E-03 |
| sp\|Q9CT10\|R/ | Ranbp3 | RANB3_MOUS | 1 | 34.9348 | 20.9686 | 27.1918 | 6.51819 | 2.91561 | 7.47098 | 27.6984 | 6.9969 | 5.6349 | 2.4027 | 0.2034 | 6.67E-03 | 1.50E-02 |
| sp\|Q6PFQ7\|R/ | Rasa4 | RASL2_MOUSI | 1 | 18.9957 | 8.16708 | 22.3078 | 13.3917 | 26.2364 | 10.9014 | 16.4902 | 7.3958 | 16.8432 | 8.2295 | 1.0214 | 9.59E-01 | 9.66E-01 |
| sp\|Q8R4X3\|RI | Rbm12 | RBM12_MOUS | 1 | 23.9444 | 28.5135 | 36.5964 | 0 | 4.04239 | 6.90338 | 29.6848 | 6.4068 | 3.6486 | 3.4685 | 0.1229 | 3.46E-03 | 9.51E-03 |
| sp\|Q9QYF1\|RI | Rdh11 | RDH11_MOUS | 1 | 26.5922 | 24.3037 | 29.5659 | 10.019 | 5.19114 | 4.32804 | 26.8206 | 2.6385 | 6.5127 | 3.0670 | 0.2428 | 9.64E-04 | 4.62E-03 |
| sp\|Q921J2\|RH | Rheb | RHEB_MOUSE | 1 | 19.7796 | 20.5686 | 21.5357 | 6.9201 | 9.37837 | 21.8177 | 20.6280 | 0.8796 | 12.7054 | 7.9866 | 0.6159 | 1.63E-01 | 2.07E-01 |
| sp\|Q8BG51\|M | Rhot1 | MIRO1_MOUS | 1 | 26.1991 | 30.5635 | 26.1316 | 5.95417 | 4.36331 | 6.78832 | 27.6314 | 2.5395 | 5.7019 | 1.2320 | 0.2064 | 1.76E-04 | 1.97E-03 |
| sp\|Q99M28\|R | Rnps1 | RNPS1_MOUS | 1 | 15.9337 | 18.4834 | 11.9489 | 18.4809 | 21.3608 | 13.7924 | 15.4553 | 3.2934 | 17.8780 | 3.8200 | 1.1568 | 4.52E-01 | 5.03E-01 |
| sp\|P17932\|RI | Rpl32-ps | RL32P_MOUSE | 1 | 9.26873 | 14.437 | 4.556 | 37.6783 | 22.1209 | 11.9391 | 9.4206 | 4.9422 | 23.9128 | 12.9628 | 2.5384 | 1.45E-01 | 1.88E-01 |
| sp\|O55142\|RL | Rpl35a | RL35A_MOUSI | 1 | 19.3452 | 14.3092 | 16.7381 | 12.5078 | 19.7046 | 17.395 | 16.7975 | 2.5185 | 16.5358 | 3.6745 | 0.9844 | 9.24E-01 | 9.36E-01 |
| sp\|P61514\|RI | Rpl37a | RL37A_MOUSI | 1 | 17.6838 | 22.424 | 13.7422 | 20.3414 | 15.9257 | 9.88272 | 17.9500 | 4.3470 | 15.3833 | 5.2504 | 0.8570 | 5.50E-01 | 5.92E-01 |
| sp\|P62843\|RS | Rps15 | RS15_MOUSE | 1 | 17.3003 | 15.7439 | 13.8037 | 18.4481 | 20.5951 | 14.1089 | 15.6160 | 1.7518 | 17.7174 | 3.3043 | 1.1346 | 3.86E-01 | 4.44E-01 |
| sp\|P62849\|RS | Rps24 | RS24_MOUSE | 1 | 18.848 | 20.8568 | 13.9816 | 17.5279 | 19.3112 | 9.47442 | 17.8955 | 3.5352 | 15.4378 | 5.2409 | 0.8627 | 5.38E-01 | 5.83E-01 |
| sp\|P62274\|RS | Rps29 | RS29_MOUSE | 1 | 15.5412 | 25.7812 | 10.7031 | 23.6743 | 15.2907 | 9.0094 | 17.3418 | 7.6986 | 15.9915 | 7.3575 | 0.9221 | 8.37E-01 | 8.61E-01 |
| sp\|Q7TT45\|RI | Rragd | RRAGD_MOUS | 1 | 24.6845 | 21.0053 | 27.8904 | 5.92716 | 7.76802 | 12.7247 | 24.5267 | 3.4453 | 8.8066 | 3.5158 | 0.3591 | 5.22E-03 | 1.28E-02 |
| sp\|Q01730\|RS | Rsu1 | RSU1_MOUSE | 1 | 17.9132 | 12.7071 | 24.3365 | 8.27895 | 10.232 | 26.5322 | 18.3189 | 5.8253 | 15.0144 | 10.0224 | 0.8196 | 6.47E-01 | 6.85E-01 |
| sp\|Q9D7H3\|R | RtcA | RTCA_MOUSE | 1 | 26.9687 | 25.2617 | 25.6187 | 7.54628 | 6.53435 | 8.07034 | 25.9497 | 0.9004 | 7.3837 | 0.7808 | 0.2845 | 1.12E-05 | 8.92E-04 |
| sp\|Q8K0T0\|RT | Rtn1 | RTN1_MOUSE | 1 | 26.8956 | 19.4139 | 26.7734 | 8.70143 | 5.17191 | 13.0439 | 24.3610 | 4.2847 | 8.9724 | 3.9430 | 0.3683 | 1.02E-02 | 2.05E-02 |
| sp\|P60122\|RU | Ruvbl1 | RUVB1_MOUS | 1 | 23.8855 | 25.7777 | 26.043 | 9.12109 | 7.84813 | 7.32453 | 25.2354 | 1.1765 | 8.0979 | 0.9240 | 0.3209 | 3.81E-05 | 1.17E-03 |
| sp\|Q9D2Q8\|S: | S100a14 | S10AE_MOUSI | 1 | 7.6378 | 5.25736 | 17.0173 | 15.2237 | 13.1549 | 41.709 | 9.9708 | 6.2174 | 23.3625 | 15.9221 | 2.3431 | 2.46E-01 | 3.01E-01 |
| sp\|P07091\|S1 | S100a4 | S10A4_MOUSI | 1 | 9.69411 | 19.3801 | 9.80259 | 30.3866 | 21.4388 | 9.29788 | 12.9589 | 5.5612 | 20.3744 | 10.5846 | 1.5722 | 3.43E-01 | 4.03E-01 |
| sp\|Q9EP69\|SA | Sacm1l | SAC1_MOUSE | 1 | 11.7208 | 29.4783 | 9.51194 | 24.2947 | 13.5696 | 11.4246 | 16.9037 | 10.9458 | 16.4296 | 6.8953 | 0.9720 | 9.52E-01 | 9.61E-01 |
| sp\|P36536\|SA | Sar1a | SAR1A_MOUS | 1 | 25.4991 | 19.2327 | 27.9064 | 11.5318 | 8.29877 | 7.5312 | 24.2127 | 4.4776 | 9.1206 | 2.1231 | 0.3767 | 6.19E-03 | 1.43E-02 |
| sp\|Q9D1J3\|SA | Sarnp | SARNP_MOUS | 1 | 33.8856 | 25.7979 | 30.4559 | 3.75554 | 0 | 6.10502 | 30.0465 | 4.0594 | 3.2869 | 3.0794 | 0.1094 | 8.10E-04 | 4.27E-03 |
| sp\|Q9JLI8\|SAI | Sart3 | SART3_MOUSI | 1 | 16.7596 | 16.6967 | 15.8681 | 16.4727 | 24.1355 | 10.0675 | 16.4415 | 0.4975 | 16.8919 | 7.0434 | 1.0274 | 9.17E-01 | 9.31E-01 |
| sp\|Q8R127\|SC | Sccpdh | SCPDL_MOUS | 1 | 27.8263 | 26.5417 | 28.2017 | 5.9821 | 3.25404 | 8.19413 | 27.5232 | 0.8705 | 5.8101 | 2.4745 | 0.2111 | 1.38E-04 | 1.82E-03 |
| sp\|Q8BRF7\|SC | Scfd1 | SCFD1_MOUSI | 1 | 8.79787 | 14.2565 | 2.89859 | 34.0997 | 16.7781 | 23.1692 | 8.6510 | 5.6804 | 24.6823 | 8.7594 | 2.8531 | 5.64E-02 | 8.17E-02 |
| sp\|Q6NZC7\|S2 | Sec23ip | S23IP_MOUSE | 1 | 24.8026 | 19.2947 | 26.6376 | 9.31928 | 7.6693 | 12.2765 | 23.5783 | 3.8215 | 9.7550 | 2.3343 | 0.4137 | 5.90E-03 | 1.38E-02 |

| | | | | | | | | | | | | | | | | |
|---|---|---|---|---|---|---|---|---|---|---|---|---|---|---|---|---|
| tr\|F6VJC5\|F6V | Sec24b | F6VJC5_MOUS | 1 | 23.9282 | 23.155 | 29.6865 | 9.40153 | 6.11448 | 7.7143 | 25.5899 | 3.5688 | 7.7434 | 1.6437 | 0.3026 | 1.41E-03 | 5.48E-03 |
| sp\|P70124\|SP | Serpinb5 | SPB5_MOUSE | 1 | 6.06989 | 0 | 20.397 | 6.05312 | 4.11013 | 63.3699 | 8.8223 | 10.4734 | 24.5111 | 33.6668 | 2.7783 | 4.84E-01 | 5.33E-01 |
| sp\|Q9D554\|SF | Sf3a3 | SF3A3_MOUSE | 1 | 24.3429 | 25.1791 | 23.0549 | 11.8202 | 8.80072 | 6.80207 | 24.1923 | 1.0701 | 9.1410 | 2.5263 | 0.3778 | 6.85E-04 | 3.89E-03 |
| sp\|Q99JR1\|SF | Sfxn1 | SFXN1_MOUSE | 1 | 17.0707 | 22.9176 | 17.5604 | 12.0392 | 19.4573 | 10.9548 | 19.1829 | 3.2436 | 14.1504 | 4.6278 | 0.7377 | 1.98E-01 | 2.47E-01 |
| sp\|Q8R0X7\|SG | Sgpl1 | SGPL1_MOUSE | 1 | 15.0939 | 16.148 | 13.5212 | 20.0487 | 20.1898 | 14.9984 | 14.9210 | 1.3219 | 18.4123 | 2.9574 | 1.2340 | 1.35E-01 | 1.77E-01 |
| sp\|Q8R550\|SH | Sh3kbp1 | SH3K1_MOUSE | 1 | 35.206 | 19.1436 | 32.7197 | 0 | 0 | 12.9308 | 29.0231 | 8.6457 | 4.3103 | 7.4656 | 0.1485 | 2.00E-02 | 3.48E-02 |
| sp\|Q9CR62\|M | Slc25a11 | M2OM_MOUS | 1 | 21.9707 | 22.5616 | 21.6358 | 13.9063 | 9.63745 | 10.288 | 22.0560 | 0.4688 | 11.2773 | 2.2999 | 0.5113 | 1.35E-03 | 5.43E-03 |
| sp\|Q9D6M3\|G | Slc25a22 | GHC1_MOUSE | 1 | 18.2052 | 28.1684 | 19.1185 | 11.3473 | 12.4717 | 10.6889 | 21.8307 | 5.5076 | 11.5026 | 0.9015 | 0.5269 | 3.27E-02 | 5.14E-02 |
| sp\|P17809\|GT | Slc2a1 | GTR1_MOUSE | 1 | 21.3945 | 23.9729 | 19.9843 | 8.97119 | 8.04449 | 17.6326 | 21.7839 | 2.0226 | 11.5494 | 5.2885 | 0.5302 | 3.52E-02 | 5.45E-02 |
| sp\|Q31125\|S3 | Slc39a7 | S39A7_MOUSE | 1 | 21.6704 | 22.6564 | 24.8285 | 9.6364 | 10.3379 | 10.8704 | 23.0518 | 1.6157 | 10.2816 | 0.6189 | 0.4460 | 2.16E-04 | 2.16E-03 |
| sp\|Q9Z127\|LA | Slc7a5 | LAT1_MOUSE | 1 | 28.279 | 31.9432 | 31.0078 | 3.76859 | 1.96958 | 3.03188 | 30.4100 | 1.9038 | 2.9234 | 0.9044 | 0.0961 | 2.28E-05 | 1.11E-03 |
| sp\|O54988\|SL | Slk | SLK_MOUSE S | 1 | 3.18874 | 2.25745 | 17.7133 | 2.18161 | 9.12152 | 65.5373 | 7.7198 | 8.6671 | 25.6135 | 34.7487 | 3.3179 | 4.36E-01 | 4.87E-01 |
| sp\|P70340\|SM | Smad1 | SMAD1_MOUS | 1 | 25.6259 | 28.4398 | 33.4587 | 0 | 5.24446 | 7.23119 | 29.1748 | 3.9678 | 4.1586 | 3.7359 | 0.1425 | 1.36E-03 | 5.43E-03 |
| sp\|Q62432\|SM | Smad2 | SMAD2_MOUS | 1 | 18.6723 | 23.0424 | 20.796 | 15.0861 | 9.73043 | 12.6728 | 20.8369 | 2.1853 | 12.4964 | 2.6822 | 0.5997 | 1.40E-02 | 2.61E-02 |
| sp\|Q6P2K6\|P4 | Smek1 | P4R3A_MOUSE | 1 | 18.1518 | 33.9381 | 29.4713 | 7.92275 | 4.90745 | 5.60859 | 27.1871 | 8.1373 | 6.1463 | 1.5779 | 0.2261 | 1.17E-02 | 2.26E-02 |
| sp\|Q3TYX3\|SM | Smyd5 | SMYD5_MOUS | 1 | 28.0707 | 20.9895 | 33.292 | 4.98347 | 6.05768 | 6.60664 | 27.4507 | 6.1746 | 5.8826 | 0.8256 | 0.2143 | 3.89E-03 | 1.02E-02 |
| sp\|P57784\|RU | Snrpa1 | RU2A_MOUSE | 1 | 27.7688 | 24.2908 | 21.7402 | 10.135 | 8.4736 | 7.59156 | 24.5999 | 3.0262 | 8.7334 | 1.2915 | 0.3550 | 1.12E-03 | 4.93E-03 |
| sp\|P62315\|SM | Snrpd1 | SMD1_MOUSE | 1 | 23.0762 | 21.0455 | 22.9739 | 11.2176 | 6.6202 | 15.0665 | 22.3652 | 1.1440 | 10.9681 | 4.2287 | 0.4904 | 1.08E-02 | 2.13E-02 |
| sp\|P62317\|SM | Snrpd2 | SMD2_MOUSE | 1 | 17.0557 | 16.5647 | 16.2732 | 18.8325 | 17.8339 | 13.4399 | 16.6312 | 0.3955 | 16.7021 | 2.8689 | 1.0043 | 9.68E-01 | 9.74E-01 |
| sp\|Q9CSN1\|SN | Snw1 | SNW1_MOUSE | 1 | 28.4152 | 29.3872 | 16.6495 | 10.5153 | 6.30853 | 8.72421 | 24.8173 | 7.0902 | 8.5160 | 2.1111 | 0.3431 | 1.88E-02 | 3.32E-02 |
| sp\|O70493\|SN | Snx12 | SNX12_MOUSE | 1 | 21.0839 | 15.3906 | 22.303 | 17.1028 | 11.157 | 12.9626 | 19.5925 | 3.6897 | 13.7408 | 3.0483 | 0.7013 | 1.02E-01 | 1.38E-01 |
| sp\|Q91VH2\|SN | Snx9 | SNX9_MOUSE | 1 | 10.7209 | 12.3891 | 13.1003 | 24.3816 | 24.3834 | 15.0247 | 12.0701 | 1.2214 | 21.2632 | 5.4027 | 1.7616 | 4.53E-02 | 6.78E-02 |
| sp\|Q58A65\|JIF | Spag9 | JIP4_MOUSE C | 1 | 26.1856 | 28.3987 | 25.0983 | 9.92841 | 4.67118 | 5.71789 | 26.5609 | 1.6819 | 6.7725 | 2.7828 | 0.2550 | 4.58E-04 | 3.14E-03 |
| sp\|Q62267\|SP | Sprr1b | SPR1B_MOUSE | 1 | 10.8857 | 4.51436 | 19.6461 | 25.1159 | 17.3521 | 22.4859 | 11.6821 | 7.5972 | 21.6513 | 3.9486 | 1.8534 | 1.14E-01 | 1.53E-01 |
| sp\|Q8BMA6\|S | Srp68 | SRP68_MOUSE | 1 | 27.3142 | 26.3462 | 22.9996 | 13.889 | 4.72237 | 4.72852 | 25.5533 | 2.2639 | 7.7800 | 5.2906 | 0.3045 | 5.89E-03 | 1.38E-02 |
| tr\|F8VQC1\|F8 | Srp72 | F8VQC1_MOU | 1 | 15.3664 | 23.2491 | 31.8312 | 18.6081 | 4.35199 | 6.59326 | 23.4822 | 8.2349 | 9.8511 | 7.6661 | 0.4195 | 1.04E-01 | 1.40E-01 |
| sp\|Q6PDM2\|S | Srsf1 | SRSF1_MOUSE | 1 | 11.289 | 15.4159 | 10.5078 | 25.0277 | 26.417 | 11.3426 | 12.4042 | 2.6373 | 20.9291 | 8.3312 | 1.6873 | 1.66E-01 | 2.11E-01 |
| tr\|D3Z4B0\|D3 | Srsf11 | D3Z4B0_MOU | 1 | 23.0382 | 25.1319 | 17.5483 | 12.5138 | 12.9438 | 8.82403 | 21.9061 | 3.9165 | 11.4272 | 2.2646 | 0.5216 | 1.60E-02 | 2.88E-02 |
| sp\|Q3TWW8\| | Srsf6 | SRSF6_MOUSE | 1 | 16.0589 | 17.218 | 12.7317 | 18.0024 | 23.4385 | 12.5505 | 15.3362 | 2.3288 | 17.9971 | 5.4440 | 1.1735 | 4.80E-01 | 5.29E-01 |
| sp\|Q9CY50\|SS | Ssr1 | SSRA_MOUSE | 1 | 22.5509 | 21.5579 | 17.2143 | 17.0423 | 10.1235 | 11.511 | 20.4410 | 2.8382 | 12.8923 | 3.6604 | 0.6307 | 4.77E-02 | 7.09E-02 |
| sp\|Q62186\|SS | Ssr4 | SSRD_MOUSE | 1 | 20.193 | 28.0254 | 27.5879 | 11.0477 | 0 | 13.146 | 25.2688 | 4.4012 | 8.0646 | 7.0625 | 0.3192 | 2.31E-02 | 3.91E-02 |
| sp\|P70297\|ST | Stam | STAM1_MOUS | 1 | 17.5075 | 19.4092 | 15.9277 | 13.802 | 18.5269 | 14.8268 | 17.6148 | 1.7432 | 15.7186 | 2.4855 | 0.8923 | 3.40E-01 | 4.00E-01 |
| sp\|P42227\|ST | Stat3 | STAT3_MOUSE | 1 | 16.1899 | 22.7298 | 40.3229 | 8.7005 | 6.69108 | 5.36578 | 26.4142 | 12.4812 | 6.9191 | 1.6790 | 0.2619 | 5.52E-02 | 8.00E-02 |
| sp\|P54116\|ST | Stom | STOM_MOUSE | 1 | 24.2753 | 25.8964 | 28.242 | 5.96507 | 4.89961 | 10.7215 | 26.1379 | 1.9943 | 7.1954 | 3.0998 | 0.2753 | 8.80E-04 | 4.49E-03 |
| sp\|Q3TDQ1\|S | Stt3b | STT3B_MOUSE | 1 | 16.2869 | 16.7491 | 17.1704 | 19.1682 | 15.8558 | 14.7696 | 16.7355 | 0.4419 | 16.5979 | 2.2913 | 0.9918 | 9.24E-01 | 9.36E-01 |
| sp\|O08599\|ST | Stxbp1 | STXB1_MOUSE | 1 | 23.4883 | 23.5192 | 29.4342 | 8.72208 | 7.08975 | 7.74644 | 25.4806 | 3.4240 | 7.8528 | 0.8213 | 0.3082 | 9.73E-04 | 4.64E-03 |
| sp\|P11031\|TC | Sub1 | TCP4_MOUSE | 1 | 32.0816 | 22.0737 | 20.1006 | 9.00501 | 5.91145 | 10.8276 | 24.7520 | 6.4239 | 8.5814 | 2.4853 | 0.3467 | 1.53E-02 | 2.78E-02 |
| sp\|P61957\|SU | Sumo2 | SUMO2_MOU | 1 | 18.7722 | 24.5553 | 22.3139 | 14.5603 | 11.0278 | 8.77053 | 21.8805 | 2.9158 | 11.4529 | 2.9182 | 0.5234 | 1.19E-02 | 2.29E-02 |
| sp\|O55201\|SP | Supt5h | SPT5H_MOUSE | 1 | 12.6672 | 14.532 | 14.1749 | 23.759 | 16.4543 | 18.4126 | 13.7914 | 0.9898 | 19.5420 | 3.7810 | 1.4170 | 6.34E-02 | 9.08E-02 |
| sp\|Q6ZWQ0\|S | Syne2 | SYNE2_MOUSE | 1 | 0.953571 | 0.203403 | 0.220857 | 37.8344 | 49.0678 | 11.72 | 0.4593 | 0.4282 | 32.8741 | 19.1616 | 71.5779 | 4.28E-02 | 6.45E-02 |
| sp\|Q9CXF4\|TB | Tbc1d15 | TBC15_MOUSE | 1 | 22.9008 | 21.6229 | 25.5968 | 14.2037 | 8.26662 | 7.40912 | 23.3735 | 2.0287 | 9.9598 | 3.7002 | 0.4261 | 5.31E-03 | 1.29E-02 |
| sp\|Q8CGF7\|TC | Tcerg1 | TCRG1_MOUS | 1 | 22.1945 | 25.9377 | 23.4414 | 11.3587 | 10.3358 | 6.73175 | 23.8579 | 1.9060 | 9.4754 | 2.4305 | 0.3972 | 1.28E-03 | 5.27E-03 |
| sp\|P35441\|TS | Thbs1 | TSP1_MOUSE | 1 | 1.523 | 0 | 0.977845 | 40.2548 | 33.5319 | 23.7124 | 0.8336 | 0.7717 | 32.4997 | 8.3194 | 38.9865 | 2.79E-03 | 8.11E-03 |
| sp\|Q9WTQ8\|T | Timm23 | TIM23_MOUSE | 1 | 17.5374 | 40.2075 | 29.2802 | 5.82621 | 3.43405 | 3.71465 | 29.0084 | 11.3375 | 4.3250 | 1.3077 | 0.1491 | 2.00E-02 | 3.48E-02 |
| sp\|Q9D880\|TI | Timm50 | TIM50_MOUSE | 1 | 24.541 | 32.0944 | 29.7144 | 7.22132 | 2.76772 | 3.66119 | 28.7833 | 3.8618 | 4.5501 | 2.3561 | 0.1581 | 7.51E-04 | 4.09E-03 |
| sp\|P58021\|TM | Tm9sf2 | TM9S2_MOUS | 1 | 19.6806 | 22.0229 | 24.4265 | 9.19116 | 13.6435 | 11.0355 | 22.0433 | 2.3730 | 11.2901 | 2.2371 | 0.5122 | 4.65E-03 | 1.16E-02 |
| sp\|Q8BXZ1\|TM | Tmx3 | TMX3_MOUSE | 1 | 28.9989 | 21.3546 | 28.5021 | 7.1739 | 3.54079 | 10.4297 | 26.2852 | 4.2772 | 7.0481 | 3.4462 | 0.2681 | 3.73E-03 | 9.86E-03 |
| sp\|Q8C0L0\|TM | Tmx4 | TMX4_MOUSE | 1 | 35.4833 | 20.8979 | 33.0875 | 3.55683 | 1.93087 | 5.04357 | 29.8229 | 7.8216 | 3.5104 | 1.5569 | 0.1177 | 4.64E-03 | 1.16E-02 |
| sp\|Q921T2\|TC | Tor1aip1 | TOIP1_MOUSE | 1 | 19.1604 | 18.2015 | 23.3926 | 16.9891 | 9.9743 | 12.282 | 20.2515 | 2.7622 | 13.0818 | 3.5751 | 0.6460 | 5.14E-02 | 7.55E-02 |
| sp\|P58774-2\|T | Tpm2 | TPM2_MOUSE | 1 | 2.8893 | 1.13041 | 1.77318 | 35.3648 | 41.6182 | 17.2242 | 1.9310 | 0.8900 | 31.4024 | 12.6705 | 16.2626 | 1.59E-02 | 2.88E-02 |
| sp\|P62996\|TR | Tra2b | TRA2B_MOUSE | 1 | 15.6054 | 17.2863 | 12.6499 | 21.085 | 21.2573 | 12.1161 | 15.1805 | 2.3472 | 18.1528 | 5.2286 | 1.1958 | 4.20E-01 | 4.73E-01 |
| sp\|Q99PP9\|TR | Trim16 | TRI16_MOUSE | 1 | 13.8503 | 19.9365 | 5.06582 | 25.1473 | 18.1462 | 17.8539 | 12.9509 | 7.4760 | 20.3825 | 4.1291 | 1.5738 | 2.06E-01 | 2.56E-01 |
| tr\|Q3U7K7\|Q3 | Trim21 | Q3U7K7_MOU | 1 | 23.9095 | 47.2988 | 17.0032 | 5.25534 | 3.33771 | 3.19543 | 29.4038 | 15.8776 | 3.9295 | 1.1504 | 0.1336 | 5.02E-02 | 7.39E-02 |
| sp\|Q4QQM4\|I | Trp53i11 | P5I11_MOUSE | 1 | 26.1794 | 32.6385 | 30.2801 | 4.49271 | 0 | 6.40921 | 29.6993 | 3.2685 | 3.6340 | 3.2898 | 0.1224 | 6.23E-04 | 3.69E-03 |
| sp\|Q9CZR8\|EF | Tsfm | EFTS_MOUSE I | 1 | 34.8564 | 25.1117 | 31.8712 | 0 | 5.50255 | 2.65809 | 30.6131 | 4.9927 | 2.7202 | 2.7518 | 0.0889 | 1.06E-03 | 4.81E-03 |
| sp\|Q62348\|TS | Tsn | TSN_MOUSE T | 1 | 24.2378 | 21.8532 | 30.0104 | 8.99366 | 7.7282 | 7.17675 | 25.3671 | 4.1942 | 7.9662 | 0.9315 | 0.3140 | 2.17E-03 | 6.84E-03 |
| sp\|P68368\|TB | Tuba4a | TBA4A_MOUS | 1 | 9.32564 | 7.75536 | 39.8533 | 7.89583 | 9.89393 | 25.276 | 18.9781 | 18.0955 | 14.3553 | 9.5103 | 0.7564 | 7.15E-01 | 7.49E-01 |
| sp\|Q9CWF2\|T | Tubb2b | TBB2B_MOUSE | 1 | 27.4196 | 31.5548 | 28.2074 | 5.4127 | 2.30434 | 5.10119 | 29.0606 | 2.1957 | 4.2727 | 1.7118 | 0.1470 | 1.03E-04 | 1.63E-03 |
| sp\|Q8CDN6\|T | Txnl1 | TXNL1_MOUSE | 1 | 22.822 | 24.8014 | 26.4189 | 11.8831 | 6.29991 | 7.7748 | 24.6808 | 1.8015 | 8.6526 | 2.8933 | 0.3506 | 1.24E-03 | 5.17E-03 |
| sp\|P26369\|U2 | U2af2 | U2AF2_MOUS | 1 | 14.3365 | 8.94241 | 17.0165 | 14.6728 | 7.97641 | 37.0555 | 13.4318 | 4.1124 | 19.9016 | 15.2284 | 1.4817 | 5.17E-01 | 5.63E-01 |
| sp\|Q8C7R4\|UI | Uba6 | UBA6_MOUSE | 1 | 15.7379 | 15.2942 | 17.9746 | 17.5627 | 10.0935 | 23.3372 | 16.3356 | 1.4367 | 16.9978 | 6.6399 | 1.0405 | 8.74E-01 | 8.93E-01 |
| sp\|P25976\|UB | Ubtf | UBF1_MOUSE | 1 | 12.9424 | 15.1369 | 18.7151 | 14.3033 | 18.5463 | 20.356 | 15.5981 | 2.9139 | 17.7352 | 3.1068 | 1.1370 | 4.34E-01 | 4.86E-01 |
| sp\|Q9WUP7\|U | Uchl5 | UCHL5_MOUS | 1 | 32.0739 | 32.6513 | 31.8308 | 0 | 0 | 3.44402 | 32.1853 | 0.4214 | 1.1480 | 1.9884 | 0.0357 | 1.21E-05 | 9.12E-04 |

| | | | | | | | | | | | | | | | | |
|---|---|---|---|---|---|---|---|---|---|---|---|---|---|---|---|---|
| sp\|P13439\|UM | Umps | UMPS_MOUSE | 1 | 29.1558 | 24.624 | 32.7058 | 6.39686 | 0 | 7.11758 | 28.8285 | 4.0508 | 4.5048 | 3.9179 | 0.1563 | 1.71E-03 | 5.98E-03 |
| sp\|Q99KD5\|UI | Unc45a | UN45A_MOUS | 1 | 22.5691 | 35.2815 | 33.7403 | 0 | 4.18598 | 4.22316 | 30.5303 | 6.9375 | 2.8030 | 2.4276 | 0.0918 | 2.83E-03 | 8.17E-03 |
| sp\|Q9CR68\|U( | Uqcrfs1 | UCRI_MOUSE | 1 | 25.1453 | 27.9413 | 24.5365 | 7.48353 | 7.45547 | 7.43788 | 25.8744 | 1.8157 | 7.4590 | 0.0230 | 0.2883 | 6.17E-05 | 1.41E-03 |
| sp\|Q8BY87\|UI | Usp47 | UBP47_MOUS | 1 | 29.4149 | 23.5093 | 33.3533 | 0 | 3.93333 | 9.78917 | 28.7592 | 4.9547 | 4.5742 | 4.9259 | 0.1591 | 3.89E-03 | 1.02E-02 |
| tr\|Q0VGU4\|Q( | Vgf | Q0VGU4_MOU | 1 | 14.8523 | 19.2768 | 6.33077 | 27.2912 | 20.1566 | 12.0923 | 13.4866 | 6.5802 | 19.8467 | 7.6042 | 1.4716 | 3.35E-01 | 3.96E-01 |
| sp\|Q9QZ88\|VI | Vps29 | VPS29_MOUSI | 1 | 20.3432 | 19.0075 | 26.4928 | 12.6966 | 10.7065 | 10.7533 | 21.9478 | 3.9923 | 11.3855 | 1.1357 | 0.5188 | 1.16E-02 | 2.25E-02 |
| sp\|Q8CCB4\|VI | Vps53 | VPS53_MOUSI | 1 | 21.2517 | 21.2762 | 39.5947 | 5.07639 | 5.34579 | 7.45521 | 27.3742 | 10.5833 | 5.9591 | 1.3026 | 0.2177 | 2.54E-02 | 4.21E-02 |
| sp\|Q8R5H6\|W | Wasf1 | WASF1_MOUS | 1 | 5.05282 | 11.7972 | 5.41172 | 34.848 | 21.7852 | 21.1051 | 7.4206 | 3.7945 | 25.9128 | 7.7456 | 3.4920 | 2.06E-02 | 3.55E-02 |
| sp\|Q8BH43\|W | Wasf2 | WASF2_MOUS | 1 | 14.889 | 14.6436 | 11.5545 | 21.0876 | 18.7297 | 19.0957 | 13.6957 | 1.8584 | 19.6377 | 1.2689 | 1.4339 | 1.02E-02 | 2.05E-02 |
| sp\|Q8C6G8\|W | Wdr26 | WDR26_MOU! | 1 | 29.6512 | 26.5429 | 25.4754 | 0 | 7.38536 | 10.9452 | 27.2232 | 2.1694 | 6.1102 | 5.5829 | 0.2244 | 3.64E-03 | 9.78E-03 |
| sp\|Q924C1\|XF | Xpo5 | XPO5_MOUSE | 1 | 24.9317 | 17.8649 | 23.5557 | 10.0768 | 16.6669 | 6.90405 | 22.1174 | 3.7465 | 11.2159 | 4.9801 | 0.5071 | 3.88E-02 | 5.92E-02 |
| sp\|Q9CRT8\|XF | Xpot | XPOT_MOUSE | 1 | 26.4218 | 20.243 | 24.466 | 9.50638 | 7.42578 | 11.937 | 23.7103 | 3.1580 | 9.6231 | 2.2579 | 0.4059 | 3.27E-03 | 9.10E-03 |
| sp\|Q9DBR1\|XI | Xrn2 | XRN2_MOUSE | 1 | 19.3929 | 17.6913 | 21.8482 | 11.3979 | 16.7547 | 12.915 | 19.6441 | 2.0898 | 13.6892 | 2.7610 | 0.6969 | 4.08E-02 | 6.18E-02 |
| sp\|Q91YT7\|YT | Ythdf2 | YTHD2_MOUS | 1 | 26.3413 | 33.6051 | 19.5175 | 8.75362 | 6.6097 | 5.17282 | 26.4880 | 7.0449 | 6.8454 | 1.8020 | 0.2584 | 9.46E-03 | 1.93E-02 |
| sp\|Q9Z2U2\|ZI | Zfp292 | ZN292_MOUSI | 1 | 27.2131 | 15.4589 | 34.687 | 6.92663 | 4.82901 | 10.8854 | 25.7863 | 9.6931 | 7.5470 | 3.0755 | 0.2927 | 3.60E-02 | 5.54E-02 |
| sp\|Q9JMD0\|ZI | Znf207 | ZN207_MOUSI | 1 | 22.9821 | 18.2834 | 22.5744 | 9.57313 | 15.956 | 10.631 | 21.2800 | 2.6031 | 12.0534 | 3.4209 | 0.5664 | 2.05E-02 | 3.54E-02 |
| sp\|Q62384\|ZP | Zpr1 | ZPR1_MOUSE | 1 | 19.5109 | 24.9239 | 42.5461 | 0 | 9.05047 | 3.96866 | 28.9936 | 12.0448 | 4.3397 | 4.5366 | 0.1497 | 2.94E-02 | 4.72E-02 |
| sp\|O54692\|ZV | Zw10 | ZW10_MOUSE | 1 | 19.4907 | 27.584 | 24.2968 | 8.94339 | 12.0675 | 7.61758 | 23.7905 | 4.0703 | 9.5428 | 2.2847 | 0.4011 | 6.14E-03 | 1.42E-02 |
| sp\|Q8BWQ6\|CP062_MOUSE | CP062_MOUSI | | 1 | 20.4991 | 39.7789 | 18.5266 | 7.26676 | 6.20418 | 7.72449 | 26.2682 | 11.7421 | 7.0651 | 0.7800 | 0.2690 | 4.75E-02 | 7.07E-02 |
