# Supplementary figures and images for: PEG3 Interacts with KAP1 through KRAB-A
Source: PLoS One. 2016 Nov 29;11(11):e0167541. doi: 10.1371/journal.pone.0167541 (PMC5127583; doi:10.1371/journal.pone.0167541)

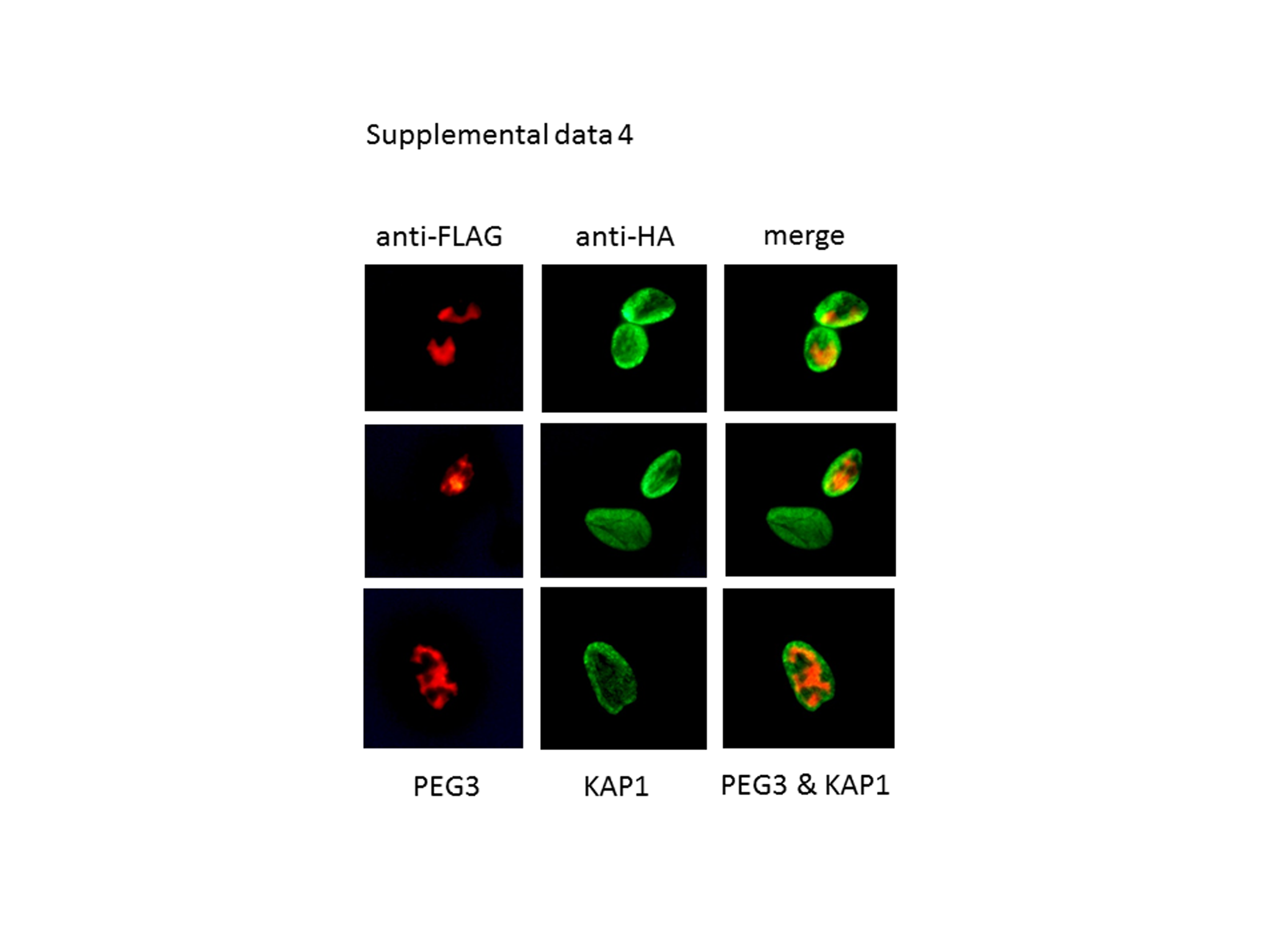

Supplement: S4 File — (TIF) [file pone.0167541.s004.tif]
